# Tracking sectoral allocation of official development assistance: a comparative study of the 29 Development Assistance Committee countries, 2011–2018

**DOI:** 10.1080/16549716.2021.1903222

**Published:** 2021-04-06

**Authors:** Shuhei Nomura, Haruka Sakamoto, Aya Ishizuka, Kazuki Shimizu, Kenji Shibuya

**Affiliations:** a Department of Health Policy and Management, School of Medicine, Keio University, Tokyo, Japan; b Department of Global Health Policy, Graduate School of Medicine, the University of Tokyo, Tokyo, Japan; c Department of Health Policy, London School of Economics and Political Science, London, UK; d Faculty of Public Health and Policy, London School of Hygiene and Tropical Medicine, London, UK; e Institute for Population Health, King’s College London, London, UK

**Keywords:** Official development assistance, OECD, Development Assistance Committee

## Abstract

**Background**: Official development assistance (ODA) is one of the most important means for donor countries to foster diplomatic relations with low- and middle-income countries and contribute to the welfare of the international community.

**Objective**: This study estimated the sectoral allocation of gross disbursements of ODA of the 29 Development Assistance Committee (DAC) member countries of the Organisation for Economic Co-operation and Development (OECD) for the duration of 2011 to 2018, by aid type (bilateral, multilateral, and both aids).

**Methods**: Data from the OECD iLibrary were used. The sector definition was based on the OECD sector classification. For core funding to multilateral agencies that do not specialize in each aid sector, we estimated ODA and its flows based on the OECD methodology for calculating imputed multilateral ODA.

**Results**: For all 29 countries, during the period of 2014–2018 where data were available for all the countries, the sector with the highest average annual ODA contribution was health at 20.34 billion USD (13.21%), followed by humanitarian aid at 18.04 billion (11.72%). Humanitarian aid has increased in the sectoral share rankings in both bilateral and multilateral aid, and the sectoral share for refugees in donor countries has increased in bilateral aid. While the 29 countries show relatively similar trends for sectoral shares, some countries and sectors display unique trends. For instance, infrastructure and energy sectors in bilateral aid of Japan are particularly high accounts for 48.48% of the total bilateral ODA of the country in 2018.

**Conclusions**: This paper evaluated ODA trends by major donors of DAC countries in the pre-COVID-19 pandemic periods. We hope that our estimates will contribute to the review of the strategic decision-making and the effective implementation of future ODA policy discussions in the DAC countries while ensuring transparency.

## Background

Development Assistance for Health (DAH) is one of the important means of achieving the Sustainable Development Goals (SDGs). While domestic finances are by far the largest source of funding for accelerating sustainable development in many low- and middle-income countries (LMICs), more robust official development assistance (ODA) policy and its efficient use are concurrently pivotal to achieve the SDGs [1]. According to figures compiled by the Organisation for Economic Co-operation and Development (OECD) Development Assistance Committee (DAC), which includes the majority of donor countries, donor spending is less than half of the 1970 pledge agreed upon at 0.7% of the Gross National Income (GNI) spending target, and in 2019, total ODA spending by DAC member countries was only 0.3% of GNI [2].

There has been a repeated emphasis on aid coordination, such as the Paris Declaration on Aid Effectiveness in 2005, that all OECD countries signed and many donor recipient countries endorsed [3]. Although OECD member countries are often high-income, donor countries that provide significant amounts of ODA, the actual scale and scope of ODA flows from each donor country – i.e. how much is allocated to which sectors and through what delivery channels, such as bilateral and multilateral channels – has not been well documented in a cross-sector, cross-country comparative manner.

The objective of this paper is to estimate the sectoral allocation of ODA in OECD DAC member countries by aid type (bilateral, multilateral, and both aids) from 2011 to 2018, for which data are available. Faced with the coronavirus disease 2019 (COVID-19) pandemic and the accompanying economic recession, a more synergistic ODA strategy is to be expected [4]. This paper will provide a foundation to consider the changing paradigm of ODA in the post-COVID-19 era.

## Methods

### Data

We employed data on ODA projects from 2011 to 2018, administered by the governments of all 29 DAC countries. The data were downloaded from the OECD iLibrary [5]. This data included, for each project and year, gross disbursements of ODA, aid type, and target aid sector. Aid type included both bilateral aid (i.e. bilateral grant including technical assistance, bilateral loan, and earmarked funding to multilaterals – often called as ‘bi-multi’ and reported as bilateral ODA) and multilateral aid (i.e. core funding to multilateral agencies including assessed contributions and non-earmarked funding). Aid sectors were based on purpose codes (also known as Creditor Reporting System [CRS] codes) for sector classification defined by OECD [6]. According to OECD, aid activities were grouped into broad three-digit sector categories, each of which is further classified into five-digit purpose codes.

Sector categories of the purpose codes used for this study were as follows based on previous studies [7–12]: 110s for education; 120s and 130s for health; 140s for water and sanitation; 151s for government and civil society; 152s for conflict, peace and security; 160s for other social services; 210s and 220s for infrastructure; 230s for energy; 240s and 250s for financial services and business support; 310s (including forestry and fishing) and 43,040 (rural development) for agriculture; 321s, 322s and 323s for industry, construction and mining; 331s for trade policy; 332s for tourism; 410s for environmental protection; 430s, excluding 43,040 (rural development) for multisector; 510s for general budget support; 520s and 530s for food aid and commodity assistance; 600s for debt relief; 720s, 730s and 740s for humanitarian aid; 910s for donor administration costs; 930s for refugees in donor country; and 998s for unspecified.

Note that the countries eligible to receive ODA are based on World Bank calculations of GNI per capita. These countries can be found on the DAC list of ODA recipients, which is revised by the DAC every three years [13]. The list includes all the Least Developed Countries, which are defined by the United Nations as the countries with the lowest levels of per capita income and socio-economic development [14]. The list also includes all the LMICs, which are defined by the World Bank in accordance with the GNI per capita [15], except for those that are members of the European Union.

The gross domestic product (GDP) deflator from the OECD national accounts was used to convert current prices of 2011 through 2017 to constant prices at 2018 [16]. For Hungary, Poland, and the Slovak Republic, the data for 2011 to 2013, 2011 to 2012, and 2011 to 2012, respectively, were not included in the OECD iLibrary, partly because they were accessioned to the DAC after these dates.

### Imputed multilateral aid to each sector

For core funding to multilateral agencies that do not specialize in each aid sector, it was not possible to directly identify the sector-specific ODA. We, therefore, estimated the sector-specific ODA based on the OECD methodology for calculating imputed multilateral ODA as follows [17]. Step 1: based on reports from multilateral agencies to the OECD [5], ODA flows to each aid sector of each agency were calculated as a percentage of total ODA disbursements (α: sectoral share of the total ODA of the agency). Step 2: The total ODA of each DAC member country was multiplied by α that was obtained for each multilateral agency (in Steps 1) to estimate flows of countries’ sector-specific ODA through the agency. For example, Japan’s multilateral health sector ODA through the World Bank was estimated by multiplying the total ODA from Japan to the World Bank by α. SN downloaded the data from the OECD iLibrary and developed an analysis algorithm for the estimation. The validity of this methodology was confirmed in our previous work [18] and elsewhere [12].

### Trend analyses of the sectoral ODA disbursements across the countries

In order to identify trends in ODA disbursements by sector in each country, rankings of the sectoral share percentages were plotted on a heat map by year. They were separately shown for the total (bi- and multilateral), bilateral, and multilateral ODA. To identify their trends that are particularly different by country, we compared the sectoral share percentages among the OECD DAC countries and determined outliers – referred to as low or high priority sector. Low or high priority sectors were defined as sectors whose sectoral share was greater than 1.5 times of interquartile range away from 25th percentile or 75th percentile of the sectoral share percentages among the countries for the sector. A high priority sector means that a country has a disproportionate preference for allocating ODA to the sector, compared to other countries’ ODA allocation. A lower priority sector means a less prioritized allocation of ODA to the sector in comparison to other countries in terms of the share percentage.

## Results

For all 29 countries, during the period of 2014 to 2018 where data were available for all countries, the sector with the highest average annual ODA contribution was health at 20.34 billion USD (13.21%), followed by humanitarian aid at 18.04 billion (11.72%), government and civil society at 13.06 billion (8.48%), and education at 10.89 billion (7.07%). The lowest sector was tourism at 0.15 billion (0.10%), followed by trade policy at 1.06 billion (0.69%), food aid and commodity assistance at 1.69 billion (1.09%), and industry, construction and mining at 2.06 billion (1.34%) (Table 1). The largest ratios on bilateral to multilateral funding were observed in refugees in donor country (99.97% for bilateral), donor administration costs (88.38%), food aid and commodity assistance (86.37%), and humanitarian aid (82.79%). The lowest ratios were observed in general budget support (52.54%), other social services (57.54%), infrastructure (63.66%), and financial services and business support (65.91%).

**Table 1.** Estimated average annual total ODA disbursements by sector and sectoral shares, and share of the bilateral and multilateral in the 29 DAC member countries, 2014–2018. Data were adjusted at constant prices of 2018. The sum of the percentages of bilateral and multilateral breakdown may not necessarily total to 100, given that data on core funding to multilateral agencies were not considered if the sectoral share data were not available in the OECD iLibrary. ODA: official development assistance; DAC: Development Assistance Committee

Sector	Total USD (% ODA share in column)	Breakdown bilateral (%), multilateral (%)
Health	20,338.02 (13.21)	68.64,31.36
Humanitarian aid	18,040.43 (11.72)	82.79,17.21
Government and civil society	13,058.56 (8.48)	75.85,24.15
Education	10,889.42 (7.07)	80.47,19.53
Infrastructure	9921.42 (6.44)	63.66,36.34
Agriculture	8546.80 (5.55)	68.12,31.88
Refugees in donor country	12,493.70 (8.11)	99.97,0.03
Donor administration costs	7956.38 (5.17)	88.38,11.62
Energy	8247.69 (5.36)	67.97,32.03
Debt relief	1549.16 (1.01)	78.98,21.02
Multisector	7437.57 (4.83)	80.38,19.62
Water and sanitation	6088.45 (3.95)	71.95,28.05
Financial services and business support	6114.81 (3.97)	65.91,34.09
Environmental protection	5024.19 (3.26)	73.58,26.42
Other social services	3731.70 (2.42)	57.54,42.46
Conflict, peace and security	3448.04 (2.24)	82.61,17.39
General budget support	2398.28 (1.56)	52.54,47.46
Unspecified	2693.51 (1.75)	74.62,25.38
Industry, construction and mining	2056.71 (1.34)	67.96,32.04
Food aid and commodity assistance	1685.13 (1.09)	86.37,13.63
Trade policy	1060.89 (0.69)	73.10,26.90
Tourism	151.34 (0.10)	71.63,28.37

**Figure 1.**
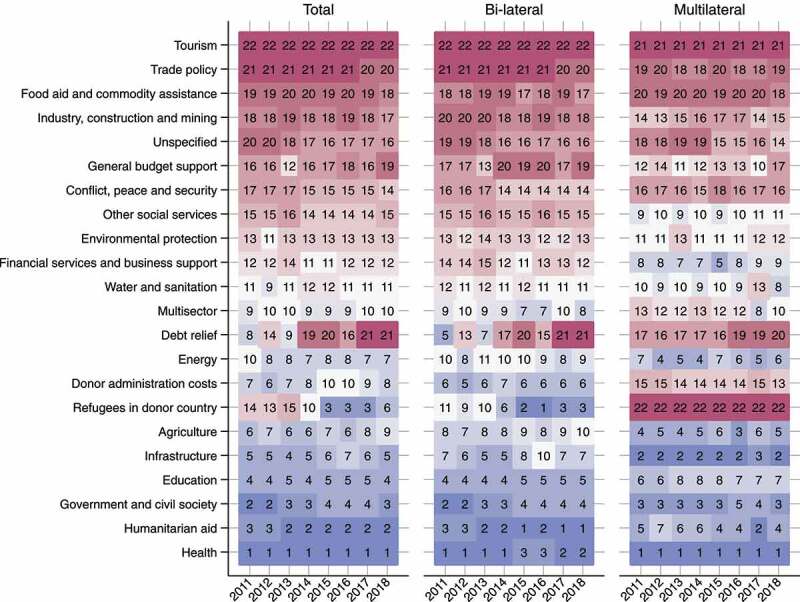
Ranking of estimated total, bilateral, and multilateral ODA by sector in the 29 DAC member countries-combined, 2011–2018. Blue and red blocks represent high and low sectoral shares, respectively. The orders of sectors and countries on axes are in descending orders of the total ODA disbursements during the study period (2011–2018). ODA: official development assistance; DAC: Development Assistance Committee

The overall rankings on sectoral share for all 29 countries during 2011 to 2018, respectively, are shown in Figure 1. Based on a visual judgment, humanitarian aid has risen in the rankings in both bilateral and multilateral funding, and refugees in donor countries has risen its rankings in bilateral funding.

**Figure 2.**
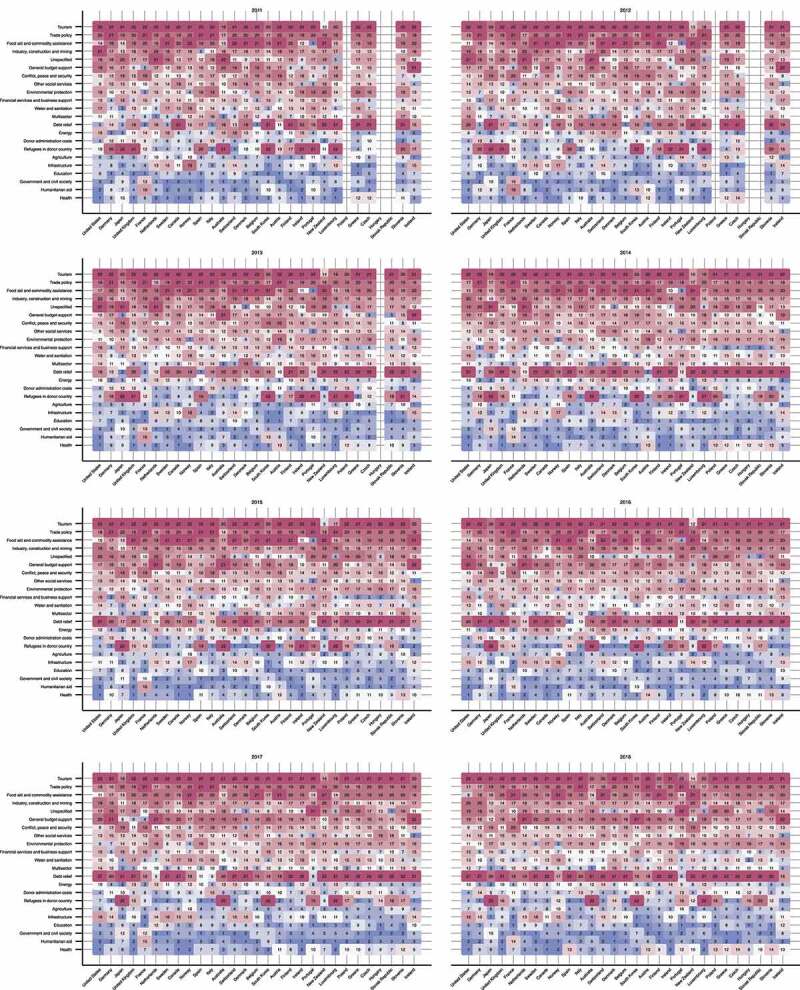
Ranking of estimated total (bi- and multilateral) ODA by sector in the 29 DAC member countries, 2011–2018. Blue and red blocks represent high and low sectoral shares, respectively. The orders of sectors and countries on axes are in descending orders of the total ODA disbursements during the study period (2011–2018). ODA: official development assistance; DAC: Development Assistance Committee

The estimated sectoral share rankings for 29 countries since 2011 are shown in Figure 2. ODA disbursements by year, country, and sector are included in the online supplementary table s1. Visual judgments show that 29 countries reveal relatively similar trends in sectoral shares while some countries and specific sectors may show unique trends. While humanitarian aid and refugees in donor countries have risen their rankings across the countries, the share ranking of health has not changed much over the years.

**Figure 3.**
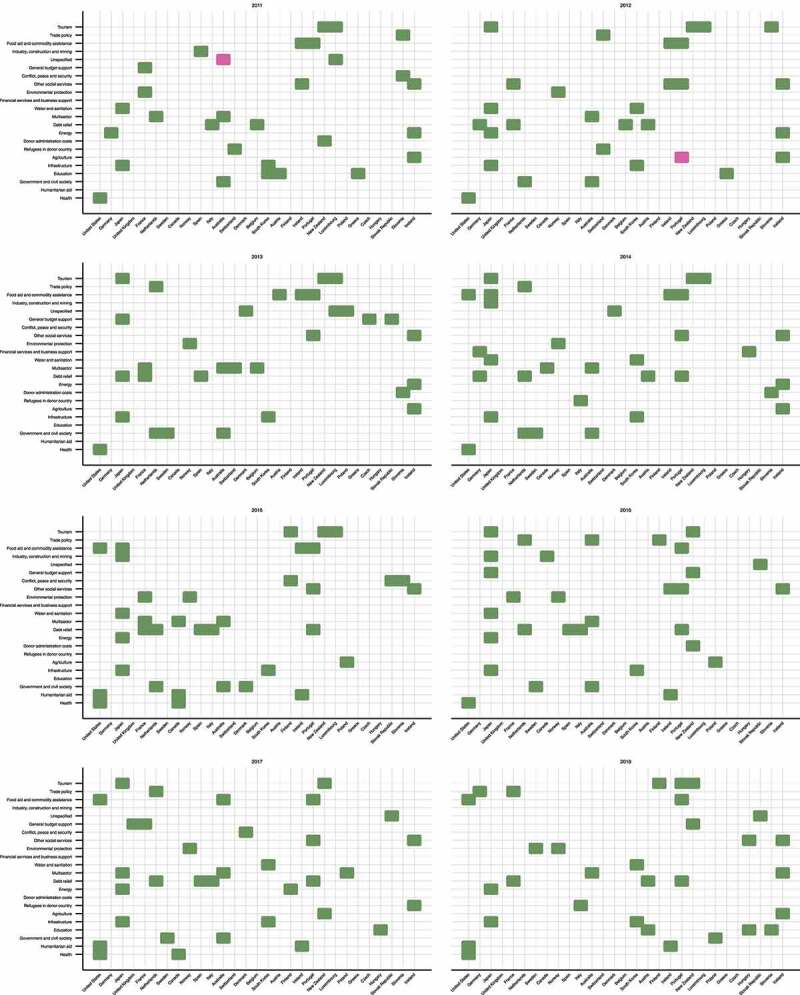
Outliers of estimated total (bi- and multilateral) ODA ranking by sector of the 29 DAC member countries, 2011–2018. Green blocks indicate higher sectoral shares compared to other countries, while pink blocks indicate lower shares. The orders of sectors and countries on axes are in descending orders of the total ODA disbursements during the study period (2011–2018). ODA: official development assistance; DAC: Development Assistance Committee

High and low priority sectors based on the sectoral share are displayed in Figure 3. The orders of sectors and countries on axes are in descending orders of the total ODA disbursements during the study period (2011–2018). The large number of high priority sectors and the small number of low priority sectors indicate that in some sectors, there are countries that give a greater priority to the ODA allocation than other countries. It also indicates that a few countries in most sectors have significantly lower ODA allocations than others. From 2012 to 2017, Japan had the largest number of high priority sectors among countries, with over four different sectors indicated to be of high priority sectors. Of these, infrastructure consistently represented a particularly higher share compared to other countries throughout all years over the study period (18.17–29.16%). Energy was also a high priority sector for five out of the eight years studied (11.73–12.71%). Portugal and Australia had relatively many high priority sectors as well; Portugal prioritized food aid and commodity assistance and other social services for all eight years and six years respectively over the study period (3.62–39.96% and 6.91–15.70%, respectively). For Australia, multisector was a high priority sector during the entire period of the study (11.46–17.13%), and government and civil society was also prioritized from 2011 to 2017 (14.68–17.52%).

The rankings of estimated sectoral shares of bilateral ODA for each country are shown in online supplementary figure s1. Disbursements by year, country, and sector are included in online supplementary table s2. The bilateral disbursement results are similar to the total results, with Japan having the largest number of the high priority sectors (online supplementary figure s2), followed by Portugal and France. In Japan, infrastructure was a high priority sector every year (19.98–34.51%), and energy was a high priority sector for five out of the eight years (9.58–14.69%). In Portugal, food aid and commodity assistance was a high priority sector (6.73–57.28%) every year. In France, infrastructure was a high priority sector (7.68–12.33%) for seven out of the eight years. In addition, a low priority sector was only identified with Poland in 2013–2014 and with Greece in 2018, both in donor administrative costs (0.26–0.53% and 0.06%, respectively).

The results of sectoral share rankings for multilateral ODA are shown in online supplementary figure s3. Disbursements by year, country, and sector are included in online supplementary table s3. The results for multilateral ODA are similar to the total results, with high priority sectors being prominently indicated for Japan (online supplementary figure s4), followed by South Korea. Japan’s contributions to energy in 2014 to 2015, followed by water and sanitation in 2011 and 2015 to 2017 were identified as high priority sectors (8.12–9.52% and 4.71–6.21%, respectively), mainly as contributions to the World Bank and other regional development banks (data not shown). South Korea also had a high priority sector for water and sanitation in 2012, 2014–2015, and 2018 (5.48–12.21%), mainly as contributions to the World Bank and other regional development banks (data not shown). On the other hand, a number of low priority sectors were observed in Norway and New Zealand, with agricultural sector being a low priority sector in particular from 2015 to 2017 for Norway and 2014 and 2017 for New Zealand (2.58–2.95% and 2.72–3.12%, respectively). Water and sanitation was also a low priority sector in Norway in 2015 (2.38%), and in New Zealand in 2014 and 2017 (2.56% and 1.43%, respectively).

## Discussion

The study provides the first comparable estimates of sectoral spending on ODA from 29 OECD DAC member countries, based on the structure of OECD CRS purpose codes. In the contemporary globalized world, world leaders are simultaneously responding to human security challenges such as epidemics of infectious diseases, threats of terrorism, refugee crisis, and climate change [19]. Health was a dominant sector that comprised most of the total ODA of all 29 countries during the study period. In the recent years, global health agendas have received extensive attention while also being recognized as national security concerns following some recent human security challenges and reoccurrences of epidemics [20,21]. Health was also at the heart of the 2000–2015 Millennium Development Goals (MDGs: a predecessor of SDGs), recognizing that it was an important measure of human well-being and central to the global agenda of poverty reduction [22]. Health was represented in three of the eight goals of the MDGs, and was acknowledged by political leaders to play a decisive role in the achievement of all the other MDG goals, especially those related to eradicating extreme poverty, hunger, and education [22]. Many LMICs experience a shortfall of domestic public resources needed to fully meet their health-care needs, making ODA for health a critical resource within these countries’ contexts. In fact, a previous study has shown that more than one-third of total health spending in low-income countries in 2014 was financed by ODA [23].

Humanitarian aid has also recorded higher sectoral share rankings. Humanitarian aid, often defined as ‘the impartial, independent and neutral provision of aid to those in immediate danger’ [24], has recently been highlighted as an increasingly important policy agenda as the number and intensity of global humanitarian crises increase with more people than ever being affected by armed conflicts, natural disasters, unplanned urbanization, climate change, food insecurity, and gender inequality [25]. Similarly, the investment in refugees has accounted for relatively higher sectoral shares, which may be indicative of the context of the 2014–2015 European migrant crisis [26]. At the country level, the prioritization for humanitarian aid and refugees has generally strengthened in most donor countries. On the contrary, scant change has observed in the rankings of health and environmental protection.

The USA (US) has contributed a substantial amount of ODA to health every year, accounting for 40–50% of the total amount of DAC’s 29 countries’ contributions. The US indicated in mid 2020 that it would suspend its contribution to the World Health Organization (WHO) over its COVID-19 response [27] though the new US president Joe Biden announced on January 2021 to resume funding for WHO [28]. This example has clearly presented that sectors relying on a large amount of contributions from a particular country would run the risk of threatening their financial sustainability due to political, economic, and societal reasons.

Donor countries engage in development assistance for many reasons, and generally there is an explicit or implicit appreciation of the value of such assistance to countries’ foreign-policy objectives [29]. The determinants of sectoral allocation of ODA are therefore influenced by a number of diplomatic, political, security, and economic considerations, including historical and traditional diplomatic relations, geographic proximity, strategic reciprocity, and trade-related aspects [30,31]. Indeed, while the 29 countries have shown relatively similar sectoral shares of trends, some countries and sectors may have unique trends. For example, there is a relatively large number of cases in Japan that diverges from the trends in other countries. Results suggest that infrastructure and energy sectors are of particular importance to Japan (especially in bilateral aid, these sectors account for 48.48% of the total bilateral ODA in 2018). It is an extremely challenging task to have measurable common indices among countries that quantitatively assess the diplomatic, political, security, and economic factors. One of the limitations of the present study, therefore, is that we provided only estimates of sectoral ODA allocations of the countries and were not able to conduct a statistical analysis to assess their determinants.

Bilateral aid refers to the flow of ODA from official government sources to the recipient country while multilateral aid is a core contribution from official government sources to a multilateral agency, which is then used to fund the agency’s own programs. In other words, while the trend of ODA allocation in bilateral aid is influenced only by the circumstances of each donor country, the trend of ODA allocation in multilateral aid is also influenced by the mandates and missions of the multilateral agencies that allocate it. In our study, with the exception of refugees in donor country and donor administration costs, the sectoral allocation of ODA tends to be roughly similar between bilateral and multilateral aids, suggesting that countries’ conditions and circumstances naturally play a dominant role in countries’ decision on which multilateral agencies they should contribute ODA to, taking into account of their mandates and missions. It is worth noting that donors may choose bilateral aid if they are primarily motivated by the need to make their ODA controllable, manageable, accountable, or visible while multilateral aid is chosen if they have the motivation promote harmonious global advancement [32].

The COVID-19 pandemic demonstrated that the mishandling of the early response and insufficient countermeasures not only negatively impacted the population health but also had put a significant burden on the extensive socio-economic activities that posed multi-sectoral challenges [33–36]. Countries are now remarkably investing in health, and for the foreseeable future, the amount of ODA to the health sector will increase. However, as we have noted earlier, health is not the only challenge the international community faces. Political leaders must simultaneously tackle climate change, migration, refugees, and many other social issues [33–37]. Crosscutting issues such as gender and human rights, embraced in the very goals of SDGs, must also be considered. Therefore, it remains to be seen whether the current momentum for health will continue or whether it will actually manifest itself in increased contributions.

Other limitations of this study arise from the very nature of ODA. While the ODA system is well known, there are many complexities in its use. This study used gross disbursements (i.e. actual distributions of committed aid funds) rather than commitments (i.e. amount the donor agreed to make available to). Disbursements are more volatile than commitments, and may be contingent on events occurring in a particular country (e.g. political or economic instability) or on the absorptive capacity of recipient countries [38]. As for the accuracy and reliability of our estimates, we cross-examined the numbers and ensured consistency with the findings of a previous study where country-specific ODA for 2016 were estimated for several sectors (agriculture, education, and health) [39].

## Conclusions

Although the emphasis on aid coordination was proclaimed in the Paris Declaration and its related documents [3], the overall picture of the actual amount of ODA provided by major donors of DAC has remained to be unclear. This study provided the first opportunity to shed light on the situation and to clarify it. In the past few years, the world has witnessed a series of milestones unfold in the aid world, including the agreement on the SDGs. At the same time, the world faces emerging challenges such as migration and refugee crisis and unprecedented natural disasters. Reflecting these, sectors related to migrants and refugees as well as humanitarian aid have relatively increased their share of ODA budged of major donors. Conversely, there has been no remarkable increase in investments to the health sector in most DAC countries during and after the Ebola outbreak in 2013–2016. There was no particular change in environmental protection in recent years either. This paper, which evaluated ODA trends prior to the COVID-19 pandemic, should invigorate policy discussions and contribute to the assessment of past decision-making processes for ODA funding, effective implementation of the ODA policies, and heightened transparency of ODA among the DAC countries.

## Supplementary Material

Supplemental Material

## Paper context

Stronger Official Development Assistance (ODA) policies and their efficient use are critical for achieving the Sustainable Development Goals. We estimated the gross disbursement of ODA of the 29 Development Assistance Committee member countries of the Organisation for Economic Co-operation and Development from 2011 to 2018 by sectors. Our estimates will contribute to the review of the strategic decision-making of future ODA policy discussions.
