# Supplementary material for: Tracking sectoral allocation of official development assistance: a comparative study of the 29 Development Assistance Committee countries, 2011–2018
Source: Glob Health Action. 2021 Apr 6;14(1):1903222. doi: 10.1080/16549716.2021.1903222 (PMC8032342; doi:10.1080/16549716.2021.1903222)
Supplement: Supplemental Material [file ZGHA_A_1903222_SM5868.docx]

**Supplementary figure s1: Raking of estimated bi-lateral ODA by sector in the 29 DAC member countries, 2011–2018.** Blue and red blocks represent high and low sectoral shares, respectively. The orders of sectors and countries on the axes are in descending orders of the total ODA disbursements during the study period (2011–2018). ODA: official development assistance; DAC: Development Assistance Committee.

**Supplementary figure s2: Outliers of estimated bi-lateral ODA ranking by sector in the 29 DAC member countries, 2011–2018.** Green blocks indicate higher sectoral shares compared with other countries, while pink blocks indicate lower shares. The orders of sectors and countries on axes are in descending orders of the total ODA disbursements during the study period (2011–2018). ODA: official development assistance; DAC: Development Assistance Committee.

**Supplementary figure s3: Raking of estimated multilateral ODA by sector in the 29 DAC member countries, 2011–2018.** Blue and red blocks represent high and low sectoral shares, respectively. The orders of sectors and countries on axes are in descending orders of the total ODA disbursements during the study period (2011–2018). ODA: official development assistance; DAC: Development Assistance Committee.

**Supplementary figure s4: Outliers of estimated multilateral ODA ranking by sector in the 29 DAC member countries, 2011–2018.** Green blocks indicate higher sectoral shares compared with other countries, while pink blocks indicate lower shares. The orders of sectors and countries on axes are in descending orders of the total ODA disbursements during the study period (2011–2018). ODA: official development assistance; DAC: Development Assistance Committee.

**Supplementary table s1: Estimated total (bi- and multilateral) ODA by sector in million USD at constant price of 2018 and sectoral shares (%) in the 29 DAC member countries, 2011–2018.** The sum of the percentages may not necessarily total to 100 given that data on core funding to multilateral agencies were not considered if the sectoral share data were not available in the OECD iLibrary. ODA: official development assistance; DAC: Development Assistance Committee.

| Country | Sector | 2011 | 2012 | 2013 | 2014 | 2015 | 2016 | 2017 | 2018 |
| --- | --- | --- | --- | --- | --- | --- | --- | --- | --- |
| United States | Health | 9733.36 (26.79) | 9716.87 (28.04) | 10046.28 (28.97) | 10280.01 (28.50) | 9006.73 (26.91) | 10912.16 (29.75) | 10842.03 (29.86) | 10081.95 (29.21) |
|  | Humanitarian aid | 4890.17 (13.46) | 4386.07 (12.66) | 5354.13 (15.44) | 6463.18 (17.92) | 6565.53 (19.62) | 6619.76 (18.05) | 7234.81 (19.92) | 7189.78 (20.83) |
|  | Government and civil society | 4518.62 (12.44) | 4704.48 (13.57) | 3578.46 (10.32) | 4283.20 (11.87) | 3076.38 (9.19) | 3392.79 (9.25) | 3149.99 (8.67) | 3493.38 (10.12) |
|  | Education | 1225.95 (3.37) | 1280.69 (3.70) | 1107.99 (3.19) | 1362.01 (3.78) | 1221.94 (3.65) | 1771.78 (4.83) | 1735.19 (4.78) | 1727.94 (5.01) |
|  | Infrastructure | 1487.47 (4.09) | 1247.36 (3.60) | 1129.52 (3.26) | 1010.88 (2.80) | 725.46 (2.17) | 506.69 (1.38) | 333.64 (0.92) | 288.07 (0.83) |
|  | Agriculture | 1827.86 (5.03) | 1750.57 (5.05) | 1658.41 (4.78) | 1771.34 (4.91) | 1724.76 (5.15) | 1752.17 (4.78) | 1501.09 (4.13) | 1298.81 (3.76) |
|  | Refugees in donor country | 940.24 (2.59) | 1067.37 (3.08) | 1103.87 (3.18) | 1362.37 (3.78) | 1453.17 (4.34) | 2001.58 (5.46) | 1491.73 (4.11) | 1621.20 (4.70) |
|  | Donor administration costs | 1966.54 (5.41) | 2516.01 (7.26) | 2476.09 (7.14) | 2197.58 (6.09) | 2330.78 (6.97) | 2338.06 (6.38) | 2951.17 (8.13) | 2682.07 (7.77) |
|  | Energy | 695.61 (1.91) | 810.67 (2.34) | 873.94 (2.52) | 773.51 (2.14) | 714.33 (2.13) | 703.24 (1.92) | 726.25 (2.00) | 670.53 (1.94) |
|  | Debt relief | 1845.88 (5.08) | 164.69 (0.48) | 405.47 (1.17) | 83.37 (0.23) | 105.47 (0.32) | 43.14 (0.12) | 46.35 (0.13) | 30.54 (0.09) |
|  | Multisector | 914.30 (2.52) | 572.54 (1.65) | 653.34 (1.88) | 770.47 (2.14) | 870.28 (2.60) | 882.96 (2.41) | 435.14 (1.20) | 557.78 (1.62) |
|  | Water and sanitation | 566.85 (1.56) | 656.36 (1.89) | 660.84 (1.91) | 508.50 (1.41) | 579.75 (1.73) | 581.34 (1.59) | 646.65 (1.78) | 535.04 (1.55) |
|  | Financial services and business support | 764.25 (2.10) | 800.11 (2.31) | 630.57 (1.82) | 628.62 (1.74) | 488.17 (1.46) | 422.70 (1.15) | 420.18 (1.16) | 418.46 (1.21) |
|  | Environmental protection | 633.19 (1.74) | 704.39 (2.03) | 748.90 (2.16) | 849.62 (2.36) | 890.20 (2.66) | 957.47 (2.61) | 840.77 (2.32) | 664.32 (1.92) |
|  | Other social services | 1008.14 (2.78) | 696.34 (2.01) | 1006.45 (2.90) | 717.09 (1.99) | 571.27 (1.71) | 618.91 (1.69) | 536.35 (1.48) | 420.48 (1.22) |
|  | Conflict, peace and security | 813.21 (2.24) | 712.67 (2.06) | 631.79 (1.82) | 614.17 (1.70) | 693.18 (2.07) | 749.29 (2.04) | 763.69 (2.10) | 806.87 (2.34) |
|  | General budget support | 251.11 (0.69) | 455.71 (1.31) | 667.17 (1.92) | 213.28 (0.59) | 311.07 (0.93) | 21.83 (0.06) | 111.23 (0.31) | 139.13 (0.40) |
|  | Unspecified | 349.24 (0.96) | 72.54 (0.21) | 74.32 (0.21) | 137.19 (0.38) | 110.60 (0.33) | 91.85 (0.25) | 306.38 (0.84) | 121.27 (0.35) |
|  | Industry, construction and mining | 214.31 (0.59) | 195.54 (0.56) | 153.76 (0.44) | 127.65 (0.35) | 128.92 (0.39) | 147.26 (0.40) | 125.48 (0.35) | 86.23 (0.25) |
|  | Food aid and commodity assistance | 757.11 (2.08) | 715.91 (2.07) | 498.15 (1.44) | 577.83 (1.60) | 777.55 (2.32) | 579.62 (1.58) | 690.74 (1.90) | 965.56 (2.80) |
|  | Trade policy | 240.36 (0.66) | 318.39 (0.92) | 304.23 (0.88) | 264.38 (0.73) | 221.64 (0.66) | 163.85 (0.45) | 232.90 (0.64) | 169.69 (0.49) |
|  | Tourism | 8.00 (0.02) | 12.53 (0.04) | 20.14 (0.06) | 41.02 (0.11) | 6.98 (0.02) | 2.54 (0.01) | 2.41 (0.01) | 4.13 (0.01) |
| Germany | Health | 948.82 (6.44) | 913.60 (6.23) | 975.22 (6.29) | 1022.79 (5.63) | 1037.78 (4.76) | 1215.72 (4.14) | 1366.85 (4.62) | 1411.45 (4.93) |
|  | Humanitarian aid | 747.25 (5.07) | 667.97 (4.55) | 876.02 (5.65) | 1206.92 (6.64) | 1237.88 (5.68) | 2668.45 (9.09) | 3328.10 (11.25) | 3235.77 (11.30) |
|  | Government and civil society | 1342.72 (9.11) | 1450.38 (9.89) | 1475.31 (9.51) | 1573.90 (8.66) | 1474.91 (6.76) | 2024.88 (6.90) | 1844.64 (6.23) | 2322.73 (8.11) |
|  | Education | 2038.24 (13.83) | 1964.52 (13.39) | 1885.64 (12.16) | 1917.44 (10.55) | 2212.78 (10.14) | 2438.80 (8.31) | 2477.65 (8.37) | 2821.12 (9.85) |
|  | Infrastructure | 794.66 (5.39) | 768.31 (5.24) | 966.67 (6.23) | 683.89 (3.76) | 946.85 (4.34) | 959.35 (3.27) | 804.15 (2.72) | 1010.18 (3.53) |
|  | Agriculture | 985.53 (6.69) | 768.94 (5.24) | 982.03 (6.33) | 898.18 (4.94) | 1063.91 (4.88) | 1309.68 (4.46) | 1343.57 (4.54) | 1341.21 (4.68) |
|  | Refugees in donor country | 81.33 (0.55) | 76.48 (0.52) | 135.38 (0.87) | 165.06 (0.91) | 3333.37 (15.28) | 7209.45 (24.55) | 6438.79 (21.76) | 3871.52 (13.52) |
|  | Donor administration costs | 526.31 (3.57) | 608.36 (4.15) | 648.89 (4.18) | 697.72 (3.84) | 709.77 (3.25) | 669.37 (2.28) | 950.71 (3.21) | 1002.64 (3.50) |
|  | Energy | 1543.79 (10.48) | 1136.92 (7.75) | 1498.75 (9.66) | 1860.60 (10.24) | 2208.56 (10.13) | 2400.97 (8.18) | 2191.50 (7.41) | 2280.59 (7.96) |
|  | Debt relief | 439.52 (2.98) | 917.97 (6.26) | 592.74 (3.82) | 1043.03 (5.74) | 89.46 (0.41) | 70.82 (0.24) | 127.27 (0.43) | 59.35 (0.21) |
|  | Multisector | 573.50 (3.89) | 702.30 (4.79) | 683.07 (4.40) | 908.57 (5.00) | 1050.43 (4.82) | 1148.87 (3.91) | 912.22 (3.08) | 1570.53 (5.48) |
|  | Water and sanitation | 857.90 (5.82) | 773.23 (5.27) | 812.05 (5.24) | 1073.62 (5.91) | 1087.59 (4.99) | 1101.00 (3.75) | 1131.76 (3.83) | 1292.22 (4.51) |
|  | Financial services and business support | 1225.61 (8.32) | 1202.60 (8.20) | 1374.50 (8.86) | 2288.86 (12.60) | 2457.54 (11.27) | 1827.26 (6.22) | 1819.61 (6.15) | 1724.38 (6.02) |
|  | Environmental protection | 622.67 (4.23) | 808.23 (5.51) | 800.10 (5.16) | 884.83 (4.87) | 837.68 (3.84) | 1287.08 (4.38) | 1391.37 (4.70) | 1132.89 (3.96) |
|  | Other social services | 355.71 (2.41) | 347.76 (2.37) | 346.54 (2.23) | 360.07 (1.98) | 316.95 (1.45) | 438.68 (1.49) | 797.20 (2.69) | 509.32 (1.78) |
|  | Conflict, peace and security | 466.43 (3.17) | 398.36 (2.72) | 453.50 (2.92) | 498.62 (2.74) | 470.03 (2.15) | 636.15 (2.17) | 899.57 (3.04) | 1094.34 (3.82) |
|  | General budget support | 331.98 (2.25) | 231.72 (1.58) | 215.68 (1.39) | 239.53 (1.32) | 162.99 (0.75) | 122.65 (0.42) | 121.04 (0.41) | 364.55 (1.27) |
|  | Unspecified | 141.13 (0.96) | 122.35 (0.83) | 123.36 (0.80) | 123.22 (0.68) | 159.75 (0.73) | 224.16 (0.76) | 200.01 (0.68) | 245.50 (0.86) |
|  | Industry, construction and mining | 285.89 (1.94) | 346.52 (2.36) | 243.68 (1.57) | 263.77 (1.45) | 239.40 (1.10) | 187.15 (0.64) | 221.52 (0.75) | 219.99 (0.77) |
|  | Food aid and commodity assistance | 111.52 (0.76) | 166.97 (1.14) | 93.15 (0.60) | 136.39 (0.75) | 201.33 (0.92) | 451.27 (1.54) | 281.50 (0.95) | 350.56 (1.22) |
|  | Trade policy | 71.67 (0.49) | 65.84 (0.45) | 76.79 (0.50) | 82.89 (0.46) | 74.70 (0.34) | 104.60 (0.36) | 347.67 (1.18) | 363.89 (1.27) |
|  | Tourism | 10.48 (0.07) | 5.31 (0.04) | 8.44 (0.05) | 5.89 (0.03) | 2.95 (0.01) | 6.26 (0.02) | 10.16 (0.03) | 8.71 (0.03) |
| Japan | Health | 684.08 (4.56) | 840.11 (6.02) | 704.00 (3.42) | 839.91 (5.39) | 850.35 (5.17) | 872.59 (5.29) | 1076.19 (5.74) | 931.26 (5.40) |
|  | Humanitarian aid | 850.11 (5.66) | 542.73 (3.89) | 808.38 (3.93) | 837.99 (5.38) | 1278.42 (7.77) | 872.17 (5.28) | 898.63 (4.80) | 749.21 (4.34) |
|  | Government and civil society | 728.89 (4.85) | 410.51 (2.94) | 679.42 (3.30) | 499.57 (3.21) | 612.15 (3.72) | 579.34 (3.51) | 582.78 (3.11) | 575.57 (3.34) |
|  | Education | 1188.28 (7.91) | 854.06 (6.12) | 802.22 (3.90) | 694.66 (4.46) | 673.66 (4.09) | 787.30 (4.77) | 757.59 (4.04) | 820.72 (4.76) |
|  | Infrastructure | 2727.23 (18.17) | 3171.40 (22.71) | 4177.49 (20.30) | 4045.98 (25.97) | 3776.29 (22.95) | 3662.59 (22.19) | 5130.49 (27.38) | 5030.40 (29.16) |
|  | Agriculture | 1073.31 (7.15) | 875.51 (6.27) | 996.79 (4.84) | 877.93 (5.63) | 891.47 (5.42) | 1121.31 (6.79) | 1107.31 (5.91) | 963.00 (5.58) |
|  | Refugees in donor country | 0.53 (0.00) | 0.56 (0.00) | 0.58 (0.00) | 0.56 (0.00) | 0.24 (0.00) | 0.24 (0.00) | 0.30 (0.00) | 0.32 (0.00) |
|  | Donor administration costs | 601.70 (4.01) | 617.63 (4.42) | 626.51 (3.05) | 638.89 (4.10) | 682.12 (4.14) | 702.38 (4.26) | 676.48 (3.61) | 739.49 (4.29) |
|  | Energy | 1295.91 (8.63) | 1775.15 (12.71) | 1264.40 (6.15) | 1535.41 (9.85) | 1954.43 (11.88) | 1936.63 (11.73) | 2367.96 (12.64) | 2190.55 (12.70) |
|  | Debt relief | 1094.82 (7.29) | 50.10 (0.36) | 3723.14 (18.10) | 35.96 (0.23) | 132.50 (0.81) | 42.26 (0.26) | 36.52 (0.19) | 44.11 (0.26) |
|  | Multisector | 731.65 (4.87) | 797.80 (5.71) | 669.61 (3.25) | 819.52 (5.26) | 1130.46 (6.87) | 1258.10 (7.62) | 1431.15 (7.64) | 1416.89 (8.21) |
|  | Water and sanitation | 1510.69 (10.06) | 1323.59 (9.48) | 1327.88 (6.45) | 1374.90 (8.82) | 1448.60 (8.80) | 1464.93 (8.87) | 1316.03 (7.02) | 1329.62 (7.71) |
|  | Financial services and business support | 147.80 (0.98) | 112.13 (0.80) | 151.05 (0.73) | 157.42 (1.01) | 210.11 (1.28) | 125.37 (0.76) | 151.72 (0.81) | 176.42 (1.02) |
|  | Environmental protection | 593.81 (3.96) | 339.96 (2.43) | 450.04 (2.19) | 430.00 (2.76) | 481.79 (2.93) | 395.16 (2.39) | 368.32 (1.97) | 119.08 (0.69) |
|  | Other social services | 310.32 (2.07) | 286.34 (2.05) | 262.02 (1.27) | 516.03 (3.31) | 260.29 (1.58) | 319.04 (1.93) | 376.67 (2.01) | 403.27 (2.34) |
|  | Conflict, peace and security | 86.31 (0.57) | 77.61 (0.56) | 86.81 (0.42) | 87.87 (0.56) | 83.75 (0.51) | 80.54 (0.49) | 74.72 (0.40) | 109.39 (0.63) |
|  | General budget support | 90.19 (0.60) | 140.51 (1.01) | 2451.39 (11.91) | 553.01 (3.55) | 223.24 (1.36) | 750.64 (4.55) | 679.72 (3.63) | 333.85 (1.94) |
|  | Unspecified | 28.48 (0.19) | 258.13 (1.85) | 49.18 (0.24) | 35.50 (0.23) | 99.17 (0.60) | 217.71 (1.32) | 139.34 (0.74) | 160.69 (0.93) |
|  | Industry, construction and mining | 528.51 (3.52) | 351.99 (2.52) | 565.21 (2.75) | 593.32 (3.81) | 633.86 (3.85) | 397.97 (2.41) | 613.84 (3.28) | 313.24 (1.82) |
|  | Food aid and commodity assistance | 333.52 (2.22) | 259.38 (1.86) | 267.56 (1.30) | 284.41 (1.83) | 353.18 (2.15) | 126.97 (0.77) | 192.86 (1.03) | 158.14 (0.92) |
|  | Trade policy | 79.94 (0.53) | 105.47 (0.76) | 111.72 (0.54) | 116.76 (0.75) | 70.57 (0.43) | 117.44 (0.71) | 53.75 (0.29) | 69.29 (0.40) |
|  | Tourism | 21.90 (0.15) | 74.32 (0.53) | 52.76 (0.26) | 74.17 (0.48) | 51.47 (0.31) | 80.20 (0.49) | 84.26 (0.45) | 24.30 (0.14) |
| United Kingdom | Health | 2105.55 (15.87) | 2235.44 (16.82) | 3354.29 (19.83) | 2888.06 (16.82) | 2277.68 (13.17) | 2247.01 (12.03) | 2874.57 (14.94) | 2939.93 (14.96) |
|  | Humanitarian aid | 978.57 (7.38) | 857.08 (6.45) | 1491.43 (8.82) | 1952.56 (11.37) | 2167.58 (12.53) | 2229.33 (11.93) | 2385.52 (12.40) | 2335.37 (11.88) |
|  | Government and civil society | 1280.47 (9.65) | 1235.28 (9.30) | 1383.94 (8.18) | 1455.78 (8.48) | 1501.49 (8.68) | 1398.19 (7.48) | 1435.31 (7.46) | 1582.52 (8.05) |
|  | Education | 1339.01 (10.09) | 1208.18 (9.09) | 1628.68 (9.63) | 1579.51 (9.20) | 1257.04 (7.27) | 1703.11 (9.12) | 1301.93 (6.77) | 1329.33 (6.76) |
|  | Infrastructure | 722.68 (5.45) | 801.12 (6.03) | 957.80 (5.66) | 857.39 (4.99) | 740.55 (4.28) | 745.64 (3.99) | 774.30 (4.02) | 917.76 (4.67) |
|  | Agriculture | 766.58 (5.78) | 683.66 (5.14) | 842.22 (4.98) | 1024.37 (5.96) | 1044.59 (6.04) | 1029.20 (5.51) | 1020.13 (5.30) | 986.51 (5.02) |
|  | Refugees in donor country | 29.30 (0.22) | 41.88 (0.32) | 48.71 (0.29) | 194.25 (1.13) | 356.32 (2.06) | 567.94 (3.04) | 513.87 (2.67) | 493.10 (2.51) |
|  | Donor administration costs | 557.38 (4.20) | 620.58 (4.67) | 486.66 (2.88) | 509.77 (2.97) | 698.94 (4.04) | 774.03 (4.14) | 861.98 (4.48) | 1000.17 (5.09) |
|  | Energy | 559.69 (4.22) | 627.13 (4.72) | 609.31 (3.60) | 658.41 (3.83) | 523.37 (3.03) | 648.16 (3.47) | 580.30 (3.02) | 788.53 (4.01) |
|  | Debt relief | 185.15 (1.40) | 185.65 (1.40) | 91.98 (0.54) | 13.67 (0.08) | 91.14 (0.53) | 12.15 (0.07) | 12.37 (0.06) | 14.32 (0.07) |
|  | Multisector | 700.17 (5.28) | 690.66 (5.20) | 878.75 (5.19) | 899.76 (5.24) | 1071.79 (6.20) | 1346.99 (7.21) | 1290.07 (6.70) | 1493.62 (7.60) |
|  | Water and sanitation | 363.02 (2.74) | 359.74 (2.71) | 438.90 (2.59) | 557.11 (3.24) | 502.23 (2.90) | 498.17 (2.67) | 449.28 (2.33) | 665.28 (3.38) |
|  | Financial services and business support | 632.08 (4.76) | 505.25 (3.80) | 613.04 (3.62) | 817.42 (4.76) | 1280.92 (7.40) | 956.52 (5.12) | 835.59 (4.34) | 1349.40 (6.86) |
|  | Environmental protection | 361.13 (2.72) | 541.25 (4.07) | 733.04 (4.33) | 637.54 (3.71) | 559.70 (3.24) | 642.85 (3.44) | 606.04 (3.15) | 625.72 (3.18) |
|  | Other social services | 567.47 (4.28) | 481.41 (3.62) | 692.56 (4.09) | 541.96 (3.16) | 658.08 (3.80) | 844.31 (4.52) | 565.50 (2.94) | 571.32 (2.91) |
|  | Conflict, peace and security | 357.19 (2.69) | 332.12 (2.50) | 331.90 (1.96) | 344.11 (2.00) | 508.76 (2.94) | 673.92 (3.61) | 693.34 (3.60) | 710.88 (3.62) |
|  | General budget support | 578.81 (4.36) | 464.54 (3.50) | 392.15 (2.32) | 219.61 (1.28) | 336.68 (1.95) | 795.90 (4.26) | 1062.06 (5.52) | 166.97 (0.85) |
|  | Unspecified | 208.86 (1.57) | 68.39 (0.51) | 320.87 (1.90) | 368.59 (2.15) | 191.30 (1.11) | 194.90 (1.04) | 422.04 (2.19) | 187.17 (0.95) |
|  | Industry, construction and mining | 320.17 (2.41) | 236.96 (1.78) | 182.77 (1.08) | 210.86 (1.23) | 227.15 (1.31) | 234.90 (1.26) | 459.35 (2.39) | 586.16 (2.98) |
|  | Food aid and commodity assistance | 177.31 (1.34) | 127.62 (0.96) | 167.59 (0.99) | 57.86 (0.34) | 69.20 (0.40) | 79.10 (0.42) | 81.36 (0.42) | 88.05 (0.45) |
|  | Trade policy | 108.57 (0.82) | 118.96 (0.90) | 159.73 (0.94) | 127.82 (0.74) | 117.21 (0.68) | 131.25 (0.70) | 139.94 (0.73) | 142.67 (0.73) |
|  | Tourism | 5.11 (0.04) | 4.94 (0.04) | 6.72 (0.04) | 4.36 (0.03) | 8.17 (0.05) | 6.85 (0.04) | 9.15 (0.05) | 12.75 (0.06) |
| France | Health | 790.24 (6.11) | 784.88 (6.04) | 876.01 (7.39) | 1116.19 (9.72) | 774.22 (6.53) | 882.63 (6.96) | 856.23 (5.94) | 909.59 (5.91) |
|  | Humanitarian aid | 338.23 (2.62) | 270.29 (2.08) | 291.23 (2.46) | 342.85 (2.99) | 361.68 (3.05) | 579.64 (4.57) | 445.21 (3.09) | 504.32 (3.28) |
|  | Government and civil society | 454.71 (3.52) | 367.48 (2.83) | 406.48 (3.43) | 385.60 (3.36) | 463.85 (3.91) | 713.69 (5.62) | 604.31 (4.19) | 1164.46 (7.57) |
|  | Education | 1582.21 (12.23) | 1566.83 (12.06) | 1515.96 (12.79) | 1480.88 (12.90) | 1423.45 (12.01) | 1444.56 (11.38) | 1549.72 (10.76) | 1577.00 (10.25) |
|  | Infrastructure | 1071.95 (8.29) | 1282.15 (9.87) | 1183.17 (9.98) | 1304.64 (11.36) | 1027.65 (8.67) | 1045.80 (8.24) | 1295.27 (8.99) | 1334.20 (8.67) |
|  | Agriculture | 634.54 (4.91) | 566.18 (4.36) | 584.68 (4.93) | 719.63 (6.27) | 671.69 (5.67) | 750.54 (5.92) | 928.68 (6.45) | 1035.85 (6.73) |
|  | Refugees in donor country | 488.70 (3.78) | 485.66 (3.74) | 418.98 (3.53) | 447.48 (3.90) | 393.46 (3.32) | 504.20 (3.97) | 657.05 (4.56) | 801.66 (5.21) |
|  | Donor administration costs | 525.66 (4.06) | 567.66 (4.37) | 580.16 (4.89) | 609.29 (5.31) | 611.81 (5.16) | 654.27 (5.16) | 695.95 (4.83) | 743.61 (4.83) |
|  | Energy | 423.85 (3.28) | 675.53 (5.20) | 815.03 (6.87) | 809.04 (7.05) | 832.44 (7.03) | 977.31 (7.70) | 1199.29 (8.32) | 1077.33 (7.00) |
|  | Debt relief | 1250.69 (9.67) | 1579.80 (12.16) | 1095.66 (9.24) | 115.47 (1.01) | 269.15 (2.27) | 166.33 (1.31) | 171.69 (1.19) | 189.65 (1.23) |
|  | Multisector | 575.40 (4.45) | 413.20 (3.18) | 979.58 (8.26) | 726.37 (6.33) | 1039.32 (8.77) | 639.56 (5.04) | 610.79 (4.24) | 630.54 (4.10) |
|  | Water and sanitation | 505.78 (3.91) | 609.68 (4.69) | 463.92 (3.91) | 592.69 (5.16) | 625.04 (5.28) | 812.45 (6.40) | 868.15 (6.03) | 952.91 (6.20) |
|  | Financial services and business support | 384.56 (2.97) | 289.57 (2.23) | 309.56 (2.61) | 337.23 (2.94) | 427.52 (3.61) | 538.86 (4.25) | 386.97 (2.69) | 756.58 (4.92) |
|  | Environmental protection | 1123.26 (8.68) | 666.38 (5.13) | 476.75 (4.02) | 577.68 (5.03) | 734.47 (6.20) | 667.31 (5.26) | 457.06 (3.17) | 580.29 (3.77) |
|  | Other social services | 525.14 (4.06) | 844.94 (6.51) | 302.99 (2.56) | 317.75 (2.77) | 382.47 (3.23) | 305.53 (2.41) | 465.09 (3.23) | 493.58 (3.21) |
|  | Conflict, peace and security | 142.63 (1.10) | 80.61 (0.62) | 127.00 (1.07) | 133.44 (1.16) | 128.35 (1.08) | 125.43 (0.99) | 96.95 (0.67) | 130.21 (0.85) |
|  | General budget support | 986.94 (7.63) | 758.57 (5.84) | 697.21 (5.88) | 355.75 (3.10) | 367.26 (3.10) | 293.23 (2.31) | 969.10 (6.73) | 491.38 (3.19) |
|  | Unspecified | 322.19 (2.49) | 301.93 (2.32) | 98.07 (0.83) | 547.72 (4.77) | 568.10 (4.79) | 615.46 (4.85) | 623.37 (4.33) | 748.83 (4.87) |
|  | Industry, construction and mining | 291.95 (2.26) | 405.34 (3.12) | 97.90 (0.83) | 88.46 (0.77) | 136.91 (1.16) | 171.51 (1.35) | 303.16 (2.10) | 275.34 (1.79) |
|  | Food aid and commodity assistance | 82.92 (0.64) | 81.73 (0.63) | 75.58 (0.64) | 62.19 (0.54) | 82.41 (0.70) | 77.33 (0.61) | 76.34 (0.53) | 95.07 (0.62) |
|  | Trade policy | 41.46 (0.32) | 28.49 (0.22) | 38.13 (0.32) | 39.23 (0.34) | 40.65 (0.34) | 44.19 (0.35) | 35.54 (0.25) | 250.84 (1.63) |
|  | Tourism | 30.46 (0.24) | 7.96 (0.06) | 18.98 (0.16) | 5.99 (0.05) | 6.01 (0.05) | 8.95 (0.07) | 18.30 (0.13) | 12.29 (0.08) |
| Netherlands | Health | 570.65 (9.50) | 500.66 (9.10) | 548.74 (10.47) | 517.30 (9.69) | 501.52 (7.79) | 583.97 (10.28) | 537.63 (9.92) | 627.20 (11.00) |
|  | Humanitarian aid | 327.59 (5.45) | 281.73 (5.12) | 289.30 (5.52) | 424.14 (7.95) | 681.84 (10.59) | 534.26 (9.40) | 579.76 (10.70) | 491.55 (8.62) |
|  | Government and civil society | 494.11 (8.22) | 1042.77 (18.96) | 997.47 (19.03) | 923.25 (17.30) | 919.13 (14.28) | 703.05 (12.37) | 642.39 (11.86) | 801.87 (14.06) |
|  | Education | 474.00 (7.89) | 336.33 (6.12) | 282.63 (5.39) | 210.56 (3.94) | 201.10 (3.12) | 244.66 (4.31) | 159.14 (2.94) | 279.58 (4.90) |
|  | Infrastructure | 147.63 (2.46) | 159.64 (2.90) | 154.33 (2.94) | 100.60 (1.88) | 132.40 (2.06) | 148.53 (2.61) | 93.70 (1.73) | 160.52 (2.81) |
|  | Agriculture | 249.35 (4.15) | 306.99 (5.58) | 364.54 (6.95) | 293.96 (5.51) | 309.82 (4.81) | 459.74 (8.09) | 316.53 (5.84) | 403.11 (7.07) |
|  | Refugees in donor country | 441.14 (7.34) | 331.33 (6.02) | 348.73 (6.65) | 872.91 (16.35) | 1466.71 (22.78) | 479.17 (8.43) | 894.03 (16.50) | 629.32 (11.03) |
|  | Donor administration costs | 322.16 (5.36) | 450.04 (8.18) | 308.31 (5.88) | 277.15 (5.19) | 420.32 (6.53) | 337.60 (5.94) | 432.59 (7.98) | 412.73 (7.24) |
|  | Energy | 229.54 (3.82) | 179.54 (3.26) | 164.05 (3.13) | 108.84 (2.04) | 121.72 (1.89) | 169.52 (2.98) | 118.76 (2.19) | 197.30 (3.46) |
|  | Debt relief | 119.77 (1.99) | 125.80 (2.29) | 198.70 (3.79) | 204.36 (3.83) | 194.00 (3.01) | 152.81 (2.69) | 209.17 (3.86) | 50.34 (0.88) |
|  | Multisector | 981.42 (16.33) | 229.93 (4.18) | 242.11 (4.62) | 123.28 (2.31) | 134.06 (2.08) | 335.95 (5.91) | 203.10 (3.75) | 166.73 (2.92) |
|  | Water and sanitation | 275.22 (4.58) | 213.18 (3.88) | 214.80 (4.10) | 216.07 (4.05) | 223.31 (3.47) | 279.87 (4.92) | 231.55 (4.27) | 293.22 (5.14) |
|  | Financial services and business support | 264.39 (4.40) | 299.62 (5.45) | 284.83 (5.43) | 322.12 (6.03) | 349.48 (5.43) | 298.46 (5.25) | 224.36 (4.14) | 334.47 (5.86) |
|  | Environmental protection | 137.82 (2.29) | 154.88 (2.82) | 114.90 (2.19) | 70.79 (1.33) | 70.55 (1.10) | 93.41 (1.64) | 71.83 (1.33) | 89.86 (1.58) |
|  | Other social services | 145.89 (2.43) | 105.93 (1.93) | 112.80 (2.15) | 91.46 (1.71) | 126.63 (1.97) | 170.66 (3.00) | 85.55 (1.58) | 141.23 (2.48) |
|  | Conflict, peace and security | 176.08 (2.93) | 186.54 (3.39) | 218.26 (4.16) | 182.34 (3.42) | 149.03 (2.32) | 203.48 (3.58) | 182.60 (3.37) | 199.73 (3.50) |
|  | General budget support | 119.73 (1.99) | 70.47 (1.28) | 55.16 (1.05) | 39.83 (0.75) | 35.97 (0.56) | 27.71 (0.49) | 28.16 (0.52) | 25.69 (0.45) |
|  | Unspecified | 32.02 (0.53) | 24.74 (0.45) | 24.03 (0.46) | 19.56 (0.37) | 53.73 (0.83) | 63.78 (1.12) | 85.56 (1.58) | 64.90 (1.14) |
|  | Industry, construction and mining | 55.11 (0.92) | 62.36 (1.13) | 29.18 (0.56) | 33.41 (0.63) | 68.10 (1.06) | 58.04 (1.02) | 66.52 (1.23) | 79.40 (1.39) |
|  | Food aid and commodity assistance | 95.46 (1.59) | 74.75 (1.36) | 51.55 (0.98) | 61.63 (1.15) | 63.04 (0.98) | 40.68 (0.72) | 53.09 (0.98) | 42.84 (0.75) |
|  | Trade policy | 77.45 (1.29) | 87.86 (1.60) | 120.06 (2.29) | 95.96 (1.80) | 80.42 (1.25) | 120.22 (2.12) | 74.00 (1.37) | 68.54 (1.20) |
|  | Tourism | 2.40 (0.04) | 1.36 (0.02) | 1.28 (0.02) | 0.64 (0.01) | 1.67 (0.03) | 1.41 (0.02) | 1.27 (0.02) | 2.10 (0.04) |
| Sweden | Health | 464.17 (9.79) | 493.98 (10.76) | 506.69 (10.41) | 457.95 (8.49) | 485.96 (6.59) | 491.67 (9.54) | 538.92 (9.51) | 540.09 (8.83) |
|  | Humanitarian aid | 581.34 (12.26) | 539.21 (11.74) | 521.92 (10.72) | 670.33 (12.42) | 670.67 (9.10) | 616.33 (11.96) | 790.56 (13.95) | 940.59 (15.38) |
|  | Government and civil society | 709.94 (14.97) | 734.85 (16.00) | 778.40 (15.99) | 828.66 (15.35) | 800.67 (10.86) | 783.38 (15.21) | 881.55 (15.56) | 948.71 (15.51) |
|  | Education | 220.32 (4.65) | 150.17 (3.27) | 186.08 (3.82) | 233.82 (4.33) | 206.58 (2.80) | 204.83 (3.98) | 217.07 (3.83) | 277.18 (4.53) |
|  | Infrastructure | 110.59 (2.33) | 120.91 (2.63) | 146.48 (3.01) | 141.88 (2.63) | 136.39 (1.85) | 115.87 (2.25) | 132.27 (2.33) | 160.33 (2.62) |
|  | Agriculture | 187.81 (3.96) | 209.19 (4.56) | 201.43 (4.14) | 222.12 (4.12) | 175.96 (2.39) | 224.88 (4.37) | 253.09 (4.47) | 280.89 (4.59) |
|  | Refugees in donor country | 410.20 (8.65) | 494.79 (10.77) | 582.94 (11.98) | 937.57 (17.37) | 2463.75 (33.42) | 844.25 (16.39) | 832.49 (14.69) | 524.75 (8.58) |
|  | Donor administration costs | 280.12 (5.91) | 263.28 (5.73) | 267.35 (5.49) | 275.22 (5.10) | 292.88 (3.97) | 275.70 (5.35) | 260.80 (4.60) | 277.68 (4.54) |
|  | Energy | 109.64 (2.31) | 122.21 (2.66) | 148.97 (3.06) | 136.68 (2.53) | 174.32 (2.36) | 112.64 (2.19) | 172.94 (3.05) | 216.80 (3.54) |
|  | Debt relief | 312.90 (6.60) | 91.03 (1.98) | 9.29 (0.19) | 6.21 (0.11) | 20.36 (0.28) | 7.84 (0.15) | 6.00 (0.11) | 6.07 (0.10) |
|  | Multisector | 193.62 (4.08) | 150.82 (3.28) | 184.63 (3.79) | 203.07 (3.76) | 228.90 (3.11) | 285.63 (5.54) | 286.00 (5.05) | 285.51 (4.67) |
|  | Water and sanitation | 99.46 (2.10) | 146.14 (3.18) | 160.23 (3.29) | 160.45 (2.97) | 154.32 (2.09) | 154.06 (2.99) | 153.53 (2.71) | 220.32 (3.60) |
|  | Financial services and business support | 138.14 (2.91) | 152.11 (3.31) | 166.30 (3.42) | 171.66 (3.18) | 128.43 (1.74) | 131.95 (2.56) | 166.94 (2.95) | 196.61 (3.21) |
|  | Environmental protection | 164.18 (3.46) | 124.36 (2.71) | 133.39 (2.74) | 245.13 (4.54) | 132.74 (1.80) | 145.68 (2.83) | 200.67 (3.54) | 356.20 (5.82) |
|  | Other social services | 112.77 (2.38) | 110.87 (2.41) | 102.21 (2.10) | 114.72 (2.13) | 135.88 (1.84) | 127.05 (2.47) | 156.49 (2.76) | 186.39 (3.05) |
|  | Conflict, peace and security | 158.97 (3.35) | 171.12 (3.73) | 178.18 (3.66) | 160.38 (2.97) | 155.23 (2.11) | 155.69 (3.02) | 190.26 (3.36) | 228.96 (3.74) |
|  | General budget support | 141.37 (2.98) | 125.07 (2.72) | 113.53 (2.33) | 72.59 (1.35) | 97.11 (1.32) | 17.20 (0.33) | 19.96 (0.35) | 15.24 (0.25) |
|  | Unspecified | 61.20 (1.29) | 95.16 (2.07) | 106.16 (2.18) | 119.50 (2.21) | 89.06 (1.21) | 201.93 (3.92) | 138.34 (2.44) | 160.80 (2.63) |
|  | Industry, construction and mining | 66.29 (1.40) | 69.02 (1.50) | 75.55 (1.55) | 62.90 (1.17) | 75.53 (1.02) | 56.04 (1.09) | 81.97 (1.45) | 93.90 (1.54) |
|  | Food aid and commodity assistance | 11.38 (0.24) | 14.83 (0.32) | 7.28 (0.15) | 8.33 (0.15) | 11.57 (0.16) | 13.16 (0.26) | 7.50 (0.13) | 16.55 (0.27) |
|  | Trade policy | 48.31 (1.02) | 50.88 (1.11) | 54.09 (1.11) | 47.54 (0.88) | 56.52 (0.77) | 44.95 (0.87) | 48.38 (0.85) | 46.74 (0.76) |
|  | Tourism | 0.57 (0.01) | 0.53 (0.01) | 1.32 (0.03) | 1.02 (0.02) | 1.89 (0.03) | 1.57 (0.03) | 2.15 (0.04) | 1.65 (0.03) |
| Canada | Health | 814.83 (17.79) | 807.84 (17.01) | 869.88 (20.66) | 610.03 (16.04) | 867.27 (19.37) | 787.62 (18.58) | 880.96 (19.87) | 899.28 (19.18) |
|  | Humanitarian aid | 405.87 (8.86) | 446.87 (9.41) | 530.62 (12.60) | 615.20 (16.17) | 746.40 (16.67) | 572.24 (13.50) | 733.10 (16.54) | 742.21 (15.83) |
|  | Government and civil society | 415.52 (9.07) | 325.54 (6.86) | 303.15 (7.20) | 273.52 (7.19) | 298.02 (6.65) | 279.92 (6.61) | 272.77 (6.15) | 306.04 (6.53) |
|  | Education | 342.96 (7.49) | 324.21 (6.83) | 281.34 (6.68) | 273.87 (7.20) | 310.77 (6.94) | 289.55 (6.83) | 263.43 (5.94) | 282.04 (6.01) |
|  | Infrastructure | 116.70 (2.55) | 133.52 (2.81) | 125.68 (2.98) | 119.59 (3.14) | 118.81 (2.65) | 87.74 (2.07) | 88.89 (2.00) | 108.74 (2.32) |
|  | Agriculture | 373.62 (8.16) | 308.59 (6.50) | 326.74 (7.76) | 261.27 (6.87) | 253.65 (5.66) | 275.90 (6.51) | 231.04 (5.21) | 263.45 (5.62) |
|  | Refugees in donor country | 282.24 (6.16) | 221.95 (4.67) | 178.19 (4.23) | 192.12 (5.05) | 220.78 (4.93) | 416.36 (9.82) | 475.66 (10.73) | 505.93 (10.79) |
|  | Donor administration costs | 250.37 (5.47) | 254.11 (5.35) | 228.55 (5.43) | 217.71 (5.72) | 218.10 (4.87) | 217.23 (5.13) | 195.28 (4.40) | 260.90 (5.56) |
|  | Energy | 281.29 (6.14) | 206.43 (4.35) | 148.09 (3.52) | 88.18 (2.32) | 92.64 (2.07) | 67.26 (1.59) | 202.18 (4.56) | 259.39 (5.53) |
|  | Debt relief | 24.46 (0.53) | 228.78 (4.82) | 26.26 (0.62) | 0.44 (0.01) | 41.52 (0.93) | 3.23 (0.08) | 7.08 (0.16) | 0.70 (0.01) |
|  | Multisector | 302.38 (6.60) | 267.72 (5.64) | 233.27 (5.54) | 419.44 (11.03) | 481.06 (10.74) | 235.90 (5.57) | 201.86 (4.55) | 258.57 (5.51) |
|  | Water and sanitation | 74.60 (1.63) | 130.22 (2.74) | 100.86 (2.39) | 94.51 (2.48) | 116.44 (2.60) | 87.91 (2.07) | 80.28 (1.81) | 100.14 (2.14) |
|  | Financial services and business support | 93.35 (2.04) | 91.20 (1.92) | 119.58 (2.84) | 96.55 (2.54) | 98.21 (2.19) | 90.49 (2.14) | 112.98 (2.55) | 129.28 (2.76) |
|  | Environmental protection | 99.31 (2.17) | 192.27 (4.05) | 84.82 (2.01) | 46.36 (1.22) | 85.07 (1.90) | 80.26 (1.89) | 98.96 (2.23) | 47.79 (1.02) |
|  | Other social services | 71.13 (1.55) | 92.22 (1.94) | 86.41 (2.05) | 67.13 (1.76) | 71.84 (1.60) | 81.84 (1.93) | 85.83 (1.94) | 105.64 (2.25) |
|  | Conflict, peace and security | 134.79 (2.94) | 90.25 (1.90) | 60.16 (1.43) | 39.43 (1.04) | 105.11 (2.35) | 54.31 (1.28) | 94.45 (2.13) | 96.47 (2.06) |
|  | General budget support | 73.73 (1.61) | 49.81 (1.05) | 47.24 (1.12) | 26.09 (0.69) | 40.65 (0.91) | 14.47 (0.34) | 8.97 (0.20) | 11.75 (0.25) |
|  | Unspecified | 98.36 (2.15) | 81.74 (1.72) | 98.70 (2.34) | 83.61 (2.20) | 64.16 (1.43) | 88.98 (2.10) | 124.87 (2.82) | 90.95 (1.94) |
|  | Industry, construction and mining | 73.53 (1.61) | 58.69 (1.24) | 85.69 (2.03) | 51.76 (1.36) | 65.02 (1.45) | 107.08 (2.53) | 91.28 (2.06) | 75.28 (1.61) |
|  | Food aid and commodity assistance | 26.48 (0.58) | 39.45 (0.83) | 41.04 (0.97) | 20.00 (0.53) | 16.99 (0.38) | 46.08 (1.09) | 27.26 (0.61) | 16.10 (0.34) |
|  | Trade policy | 33.78 (0.74) | 43.78 (0.92) | 13.48 (0.32) | 22.80 (0.60) | 23.07 (0.52) | 27.97 (0.66) | 17.86 (0.40) | 14.62 (0.31) |
|  | Tourism | 1.09 (0.02) | 1.05 (0.02) | 4.74 (0.11) | 5.27 (0.14) | 3.71 (0.08) | 1.94 (0.05) | 1.56 (0.04) | 2.93 (0.06) |
| Norway | Health | 412.59 (11.26) | 426.01 (11.35) | 516.81 (11.95) | 551.11 (13.23) | 568.42 (12.30) | 553.39 (11.07) | 586.19 (13.14) | 560.42 (13.02) |
|  | Humanitarian aid | 354.79 (9.68) | 315.75 (8.41) | 383.17 (8.86) | 427.27 (10.25) | 507.13 (10.97) | 600.57 (12.01) | 737.44 (16.53) | 681.91 (15.85) |
|  | Government and civil society | 442.79 (12.08) | 411.72 (10.97) | 472.61 (10.92) | 449.47 (10.79) | 451.57 (9.77) | 402.69 (8.05) | 400.78 (8.98) | 436.89 (10.15) |
|  | Education | 249.01 (6.79) | 251.19 (6.69) | 259.59 (6.00) | 273.64 (6.57) | 371.13 (8.03) | 468.21 (9.37) | 442.77 (9.92) | 407.05 (9.46) |
|  | Infrastructure | 24.34 (0.66) | 46.03 (1.23) | 43.44 (1.00) | 49.17 (1.18) | 48.05 (1.04) | 42.37 (0.85) | 40.38 (0.91) | 54.62 (1.27) |
|  | Agriculture | 147.52 (4.02) | 167.14 (4.45) | 201.72 (4.66) | 170.03 (4.08) | 163.27 (3.53) | 128.37 (2.57) | 151.77 (3.40) | 136.35 (3.17) |
|  | Refugees in donor country | 202.85 (5.53) | 175.50 (4.68) | 205.88 (4.76) | 227.26 (5.45) | 497.15 (10.75) | 908.31 (18.17) | 161.20 (3.61) | 101.71 (2.36) |
|  | Donor administration costs | 242.46 (6.62) | 245.72 (6.55) | 267.15 (6.18) | 266.40 (6.39) | 293.07 (6.34) | 296.57 (5.93) | 299.93 (6.72) | 288.68 (6.71) |
|  | Energy | 225.33 (6.15) | 303.79 (8.10) | 209.83 (4.85) | 193.28 (4.64) | 160.90 (3.48) | 114.49 (2.29) | 104.39 (2.34) | 110.17 (2.56) |
|  | Debt relief | 84.16 (2.30) | 35.80 (0.95) | 23.34 (0.54) | 19.30 (0.46) | 29.41 (0.64) | 26.55 (0.53) | 31.16 (0.70) | 30.36 (0.71) |
|  | Multisector | 132.47 (3.61) | 146.27 (3.90) | 138.03 (3.19) | 131.69 (3.16) | 117.58 (2.54) | 124.74 (2.50) | 145.08 (3.25) | 182.81 (4.25) |
|  | Water and sanitation | 31.70 (0.86) | 42.84 (1.14) | 46.96 (1.09) | 56.93 (1.37) | 52.45 (1.13) | 55.57 (1.11) | 36.08 (0.81) | 47.28 (1.10) |
|  | Financial services and business support | 141.55 (3.86) | 99.82 (2.66) | 112.87 (2.61) | 190.17 (4.56) | 225.68 (4.88) | 217.30 (4.35) | 234.44 (5.25) | 241.24 (5.61) |
|  | Environmental protection | 262.05 (7.15) | 377.10 (10.05) | 756.49 (17.49) | 522.64 (12.54) | 471.53 (10.20) | 429.28 (8.59) | 438.21 (9.82) | 457.69 (10.64) |
|  | Other social services | 91.28 (2.49) | 80.22 (2.14) | 98.34 (2.27) | 79.68 (1.91) | 89.34 (1.93) | 87.92 (1.76) | 101.72 (2.28) | 70.34 (1.63) |
|  | Conflict, peace and security | 185.00 (5.05) | 181.93 (4.85) | 180.57 (4.17) | 180.64 (4.34) | 176.52 (3.82) | 154.34 (3.09) | 178.89 (4.01) | 204.13 (4.74) |
|  | General budget support | 120.03 (3.27) | 95.97 (2.56) | 75.96 (1.76) | 62.73 (1.51) | 57.88 (1.25) | 38.57 (0.77) | 35.45 (0.79) | 20.59 (0.48) |
|  | Unspecified | 50.52 (1.38) | 50.01 (1.33) | 46.15 (1.07) | 54.04 (1.30) | 77.50 (1.68) | 66.89 (1.34) | 93.42 (2.09) | 65.21 (1.52) |
|  | Industry, construction and mining | 51.67 (1.41) | 52.88 (1.41) | 48.95 (1.13) | 45.37 (1.09) | 45.92 (0.99) | 29.39 (0.59) | 31.13 (0.70) | 29.22 (0.68) |
|  | Food aid and commodity assistance | 1.56 (0.04) | 5.77 (0.15) | 4.28 (0.10) | 1.74 (0.04) | 1.70 (0.04) | 2.48 (0.05) | 1.33 (0.03) | 2.40 (0.06) |
|  | Trade policy | 15.53 (0.42) | 15.61 (0.42) | 13.46 (0.31) | 13.23 (0.32) | 13.83 (0.30) | 14.77 (0.30) | 11.34 (0.25) | 14.20 (0.33) |
|  | Tourism | 10.10 (0.28) | 2.84 (0.08) | 2.70 (0.06) | 0.86 (0.02) | 0.82 (0.02) | 0.46 (0.01) | 0.50 (0.01) | 0.83 (0.02) |
| Spain | Health | 244.82 (6.22) | 110.75 (5.49) | 130.40 (5.52) | 108.41 (5.56) | 91.84 (4.67) | 147.01 (2.96) | 164.46 (5.24) | 129.82 (4.36) |
|  | Humanitarian aid | 351.03 (8.91) | 168.82 (8.36) | 155.42 (6.58) | 186.88 (9.59) | 168.76 (8.58) | 234.14 (4.72) | 216.06 (6.89) | 215.89 (7.25) |
|  | Government and civil society | 373.85 (9.49) | 185.29 (9.18) | 186.94 (7.91) | 174.33 (8.95) | 187.19 (9.51) | 248.35 (5.01) | 291.77 (9.30) | 339.34 (11.40) |
|  | Education | 336.13 (8.53) | 146.02 (7.23) | 124.72 (5.28) | 96.20 (4.94) | 94.06 (4.78) | 169.19 (3.41) | 162.58 (5.18) | 175.84 (5.91) |
|  | Infrastructure | 214.85 (5.45) | 131.01 (6.49) | 162.34 (6.87) | 145.89 (7.49) | 113.68 (5.78) | 210.53 (4.24) | 190.46 (6.07) | 248.18 (8.34) |
|  | Agriculture | 291.55 (7.40) | 131.85 (6.53) | 162.10 (6.86) | 150.32 (7.72) | 131.49 (6.68) | 238.65 (4.81) | 205.15 (6.54) | 205.21 (6.89) |
|  | Refugees in donor country | 29.25 (0.74) | 21.61 (1.07) | 23.50 (0.99) | 18.39 (0.94) | 35.33 (1.80) | 96.86 (1.95) | 229.59 (7.32) | 275.54 (9.25) |
|  | Donor administration costs | 214.13 (5.44) | 200.00 (9.91) | 172.36 (7.29) | 161.78 (8.30) | 157.65 (8.01) | 173.71 (3.50) | 160.54 (5.12) | 164.26 (5.52) |
|  | Energy | 208.54 (5.29) | 85.36 (4.23) | 110.72 (4.68) | 90.07 (4.62) | 70.91 (3.60) | 159.16 (3.21) | 158.23 (5.05) | 148.15 (4.98) |
|  | Debt relief | 71.07 (1.80) | 75.27 (3.73) | 223.97 (9.48) | 0.15 (0.01) | 134.18 (6.82) | 2310.53 (46.57) | 130.19 (4.15) | 5.48 (0.18) |
|  | Multisector | 146.71 (3.72) | 79.65 (3.95) | 85.40 (3.61) | 75.35 (3.87) | 77.15 (3.92) | 169.04 (3.41) | 196.42 (6.26) | 150.61 (5.06) |
|  | Water and sanitation | 182.42 (4.63) | 59.45 (2.94) | 116.84 (4.94) | 96.61 (4.96) | 69.26 (3.52) | 112.74 (2.27) | 81.67 (2.60) | 149.23 (5.01) |
|  | Financial services and business support | 153.22 (3.89) | 142.64 (7.06) | 122.61 (5.19) | 127.30 (6.53) | 143.07 (7.27) | 164.72 (3.32) | 126.79 (4.04) | 133.71 (4.49) |
|  | Environmental protection | 68.09 (1.73) | 34.80 (1.72) | 29.45 (1.25) | 40.62 (2.08) | 36.01 (1.83) | 57.62 (1.16) | 54.92 (1.75) | 50.29 (1.69) |
|  | Other social services | 197.97 (5.03) | 82.38 (4.08) | 96.07 (4.07) | 73.75 (3.79) | 94.29 (4.79) | 112.05 (2.26) | 102.10 (3.26) | 89.01 (2.99) |
|  | Conflict, peace and security | 106.33 (2.70) | 49.75 (2.46) | 60.28 (2.55) | 54.12 (2.78) | 53.35 (2.71) | 58.37 (1.18) | 64.01 (2.04) | 78.18 (2.63) |
|  | General budget support | 83.98 (2.13) | 57.85 (2.87) | 60.32 (2.55) | 73.07 (3.75) | 54.53 (2.77) | 49.96 (1.01) | 43.81 (1.40) | 37.37 (1.25) |
|  | Unspecified | 154.88 (3.93) | 66.97 (3.32) | 57.07 (2.41) | 46.09 (2.37) | 114.64 (5.83) | 73.80 (1.49) | 108.40 (3.46) | 133.46 (4.48) |
|  | Industry, construction and mining | 252.44 (6.41) | 80.04 (3.96) | 29.33 (1.24) | 32.26 (1.66) | 35.42 (1.80) | 34.01 (0.69) | 66.62 (2.12) | 58.09 (1.95) |
|  | Food aid and commodity assistance | 45.15 (1.15) | 30.02 (1.49) | 22.69 (0.96) | 15.62 (0.80) | 22.42 (1.14) | 21.34 (0.43) | 19.25 (0.61) | 26.24 (0.88) |
|  | Trade policy | 18.90 (0.48) | 8.86 (0.44) | 13.49 (0.57) | 14.49 (0.74) | 12.95 (0.66) | 21.82 (0.44) | 16.35 (0.52) | 16.58 (0.56) |
|  | Tourism | 4.75 (0.12) | 2.04 (0.10) | 1.88 (0.08) | 3.09 (0.16) | 1.82 (0.09) | 6.86 (0.14) | 6.60 (0.21) | 6.25 (0.21) |
| Italy | Health | 179.23 (4.26) | 137.84 (5.02) | 166.56 (5.05) | 204.90 (5.42) | 228.50 (5.15) | 250.75 (4.48) | 288.95 (4.49) | 310.24 (5.96) |
|  | Humanitarian aid | 267.58 (6.37) | 236.29 (8.60) | 247.09 (7.49) | 277.33 (7.34) | 315.42 (7.11) | 463.25 (8.28) | 531.49 (8.26) | 480.03 (9.22) |
|  | Government and civil society | 223.56 (5.32) | 181.75 (6.61) | 233.47 (7.08) | 270.24 (7.15) | 327.90 (7.40) | 271.76 (4.86) | 432.56 (6.73) | 494.80 (9.50) |
|  | Education | 206.70 (4.92) | 161.17 (5.86) | 176.56 (5.35) | 190.70 (5.05) | 225.48 (5.09) | 258.11 (4.62) | 265.30 (4.13) | 288.33 (5.54) |
|  | Infrastructure | 329.76 (7.85) | 285.73 (10.40) | 366.62 (11.12) | 274.68 (7.27) | 272.45 (6.15) | 291.20 (5.21) | 304.32 (4.73) | 387.15 (7.44) |
|  | Agriculture | 210.81 (5.02) | 139.59 (5.08) | 210.95 (6.40) | 228.23 (6.04) | 265.76 (5.99) | 324.79 (5.81) | 309.24 (4.81) | 318.75 (6.12) |
|  | Refugees in donor country | 478.48 (11.38) | 239.72 (8.72) | 376.99 (11.43) | 777.02 (20.57) | 1074.45 (24.24) | 1805.24 (32.28) | 1905.29 (29.63) | 1124.71 (21.60) |
|  | Donor administration costs | 127.62 (3.04) | 100.50 (3.66) | 109.50 (3.32) | 112.55 (2.98) | 111.48 (2.51) | 98.63 (1.76) | 121.67 (1.89) | 129.08 (2.48) |
|  | Energy | 212.74 (5.06) | 177.43 (6.46) | 190.11 (5.77) | 199.49 (5.28) | 173.40 (3.91) | 212.27 (3.80) | 379.51 (5.90) | 225.05 (4.32) |
|  | Debt relief | 745.50 (17.74) | 32.17 (1.17) | 49.70 (1.51) | 36.76 (0.97) | 111.37 (2.51) | 167.47 (2.99) | 227.92 (3.54) | 24.86 (0.48) |
|  | Multisector | 94.08 (2.24) | 88.38 (3.22) | 107.75 (3.27) | 102.27 (2.71) | 100.77 (2.27) | 125.45 (2.24) | 183.46 (2.85) | 234.56 (4.50) |
|  | Water and sanitation | 114.76 (2.73) | 94.68 (3.44) | 110.02 (3.34) | 140.69 (3.72) | 117.88 (2.66) | 121.59 (2.17) | 112.01 (1.74) | 166.09 (3.19) |
|  | Financial services and business support | 163.60 (3.89) | 169.42 (6.16) | 193.31 (5.86) | 218.82 (5.79) | 253.85 (5.73) | 234.81 (4.20) | 182.19 (2.83) | 195.48 (3.75) |
|  | Environmental protection | 59.14 (1.41) | 60.02 (2.18) | 80.31 (2.44) | 93.83 (2.48) | 90.28 (2.04) | 114.23 (2.04) | 139.83 (2.17) | 86.31 (1.66) |
|  | Other social services | 106.29 (2.53) | 111.75 (4.07) | 136.08 (4.13) | 148.35 (3.93) | 157.53 (3.55) | 155.27 (2.78) | 146.81 (2.28) | 133.94 (2.57) |
|  | Conflict, peace and security | 75.70 (1.80) | 34.07 (1.24) | 50.46 (1.53) | 61.82 (1.64) | 76.96 (1.74) | 90.25 (1.61) | 98.04 (1.52) | 100.70 (1.93) |
|  | General budget support | 131.39 (3.13) | 95.74 (3.48) | 112.63 (3.42) | 117.62 (3.11) | 90.13 (2.03) | 72.58 (1.30) | 70.72 (1.10) | 56.62 (1.09) |
|  | Unspecified | 72.10 (1.72) | 47.20 (1.72) | 49.68 (1.51) | 35.92 (0.95) | 93.02 (2.10) | 46.15 (0.83) | 127.55 (1.98) | 59.92 (1.15) |
|  | Industry, construction and mining | 147.69 (3.51) | 138.48 (5.04) | 76.71 (2.33) | 54.09 (1.43) | 77.95 (1.76) | 53.11 (0.95) | 109.95 (1.71) | 99.45 (1.91) |
|  | Food aid and commodity assistance | 46.07 (1.10) | 31.57 (1.15) | 40.96 (1.24) | 25.07 (0.66) | 41.52 (0.94) | 60.04 (1.07) | 48.31 (0.75) | 46.12 (0.89) |
|  | Trade policy | 28.33 (0.67) | 17.01 (0.62) | 25.51 (0.77) | 25.68 (0.68) | 22.60 (0.51) | 27.90 (0.50) | 23.25 (0.36) | 23.24 (0.45) |
|  | Tourism | 4.32 (0.10) | 3.06 (0.11) | 3.64 (0.11) | 2.38 (0.06) | 4.61 (0.10) | 4.01 (0.07) | 6.63 (0.10) | 7.46 (0.14) |
| Australia | Health | 500.26 (13.27) | 581.66 (13.56) | 480.69 (11.84) | 458.69 (11.84) | 382.76 (10.29) | 359.14 (10.30) | 259.16 (8.49) | 338.75 (10.75) |
|  | Humanitarian aid | 363.52 (9.64) | 315.01 (7.34) | 251.11 (6.18) | 292.37 (7.55) | 308.56 (8.30) | 210.38 (6.03) | 244.77 (8.02) | 224.56 (7.12) |
|  | Government and civil society | 646.90 (17.16) | 751.63 (17.52) | 662.21 (16.31) | 568.77 (14.68) | 548.13 (14.74) | 529.66 (15.19) | 508.95 (16.67) | 463.06 (14.69) |
|  | Education | 379.69 (10.07) | 484.73 (11.30) | 381.77 (9.40) | 499.66 (12.90) | 388.86 (10.45) | 279.54 (8.01) | 221.30 (7.25) | 245.64 (7.79) |
|  | Infrastructure | 225.88 (5.99) | 192.74 (4.49) | 182.63 (4.50) | 197.01 (5.08) | 215.78 (5.80) | 189.99 (5.45) | 157.27 (5.15) | 267.01 (8.47) |
|  | Agriculture | 250.53 (6.65) | 331.36 (7.72) | 217.51 (5.36) | 251.08 (6.48) | 185.29 (4.98) | 180.01 (5.16) | 195.28 (6.39) | 199.26 (6.32) |
|  | Refugees in donor country | 0.01 (0.00) | 119.65 (2.79) | 282.48 (6.96) | 0.00 (0.00) | 0.00 (0.00) | 0.00 (0.00) | 0.00 (0.00) | 0.00 (0.00) |
|  | Donor administration costs | 234.79 (6.23) | 280.80 (6.54) | 270.37 (6.66) | 261.93 (6.76) | 198.88 (5.35) | 213.38 (6.12) | 200.78 (6.57) | 199.86 (6.34) |
|  | Energy | 25.88 (0.69) | 64.27 (1.50) | 38.94 (0.96) | 64.06 (1.65) | 54.63 (1.47) | 52.67 (1.51) | 51.06 (1.67) | 64.81 (2.06) |
|  | Debt relief | 9.93 (0.26) | 9.30 (0.22) | 10.40 (0.26) | 6.69 (0.17) | 16.26 (0.44) | 8.37 (0.24) | 3.07 (0.10) | 0.29 (0.01) |
|  | Multisector | 526.04 (13.96) | 491.52 (11.46) | 633.50 (15.60) | 603.52 (15.58) | 637.32 (17.13) | 478.43 (13.72) | 506.14 (16.57) | 470.84 (14.94) |
|  | Water and sanitation | 197.84 (5.25) | 155.75 (3.63) | 166.59 (4.10) | 135.80 (3.51) | 125.39 (3.37) | 102.12 (2.93) | 81.52 (2.67) | 144.97 (4.60) |
|  | Financial services and business support | 39.35 (1.04) | 60.96 (1.42) | 44.48 (1.10) | 59.71 (1.54) | 87.07 (2.34) | 107.90 (3.09) | 116.37 (3.81) | 83.59 (2.65) |
|  | Environmental protection | 90.53 (2.40) | 116.95 (2.73) | 106.97 (2.63) | 105.46 (2.72) | 52.55 (1.41) | 35.17 (1.01) | 45.80 (1.50) | 38.80 (1.23) |
|  | Other social services | 94.91 (2.52) | 85.73 (2.00) | 88.94 (2.19) | 98.43 (2.54) | 79.18 (2.13) | 79.23 (2.27) | 63.29 (2.07) | 73.10 (2.32) |
|  | Conflict, peace and security | 86.26 (2.29) | 56.81 (1.32) | 45.29 (1.12) | 47.11 (1.22) | 38.52 (1.04) | 45.33 (1.30) | 25.32 (0.83) | 63.04 (2.00) |
|  | General budget support | 0.08 (0.00) | 9.00 (0.21) | 5.41 (0.13) | 32.12 (0.83) | 0.15 (0.00) | 3.27 (0.09) | 5.53 (0.18) | 3.03 (0.10) |
|  | Unspecified | -73.07 (-1.94) | 6.43 (0.15) | 34.12 (0.84) | 24.59 (0.63) | 22.03 (0.59) | 103.38 (2.96) | 8.63 (0.28) | 30.15 (0.96) |
|  | Industry, construction and mining | 22.27 (0.59) | 22.25 (0.52) | 36.06 (0.89) | 19.55 (0.50) | 36.52 (0.98) | 28.38 (0.81) | 42.84 (1.40) | 55.34 (1.76) |
|  | Food aid and commodity assistance | 45.17 (1.20) | 35.47 (0.83) | 37.88 (0.93) | 24.85 (0.64) | 27.01 (0.73) | 45.34 (1.30) | 57.40 (1.88) | 57.42 (1.82) |
|  | Trade policy | 19.26 (0.51) | 20.56 (0.48) | 22.64 (0.56) | 18.99 (0.49) | 40.67 (1.09) | 38.78 (1.11) | 30.74 (1.01) | 30.81 (0.98) |
|  | Tourism | 0.33 (0.01) | 0.32 (0.01) | 0.69 (0.02) | 0.89 (0.02) | 0.84 (0.02) | 0.56 (0.02) | 0.97 (0.03) | 2.64 (0.08) |
| Switzerland | Health | 143.74 (5.23) | 145.79 (5.01) | 148.99 (4.96) | 207.17 (6.24) | 200.44 (5.70) | 186.71 (5.08) | 216.05 (6.74) | 204.90 (6.53) |
|  | Humanitarian aid | 298.96 (10.87) | 312.95 (10.75) | 348.56 (11.59) | 395.15 (11.90) | 455.83 (12.96) | 406.70 (11.06) | 395.18 (12.33) | 386.68 (12.33) |
|  | Government and civil society | 218.18 (7.94) | 215.64 (7.41) | 261.86 (8.71) | 276.04 (8.32) | 320.80 (9.12) | 323.20 (8.79) | 362.72 (11.31) | 392.13 (12.50) |
|  | Education | 128.32 (4.67) | 107.46 (3.69) | 120.95 (4.02) | 149.32 (4.50) | 161.35 (4.59) | 174.83 (4.75) | 176.01 (5.49) | 181.47 (5.79) |
|  | Infrastructure | 66.01 (2.40) | 66.98 (2.30) | 81.30 (2.70) | 82.92 (2.50) | 84.68 (2.41) | 84.18 (2.29) | 78.04 (2.43) | 87.50 (2.79) |
|  | Agriculture | 181.31 (6.59) | 161.54 (5.55) | 185.93 (6.18) | 202.37 (6.10) | 232.18 (6.60) | 238.83 (6.49) | 227.67 (7.10) | 221.22 (7.05) |
|  | Refugees in donor country | 476.23 (17.32) | 613.99 (21.09) | 417.45 (13.88) | 445.37 (13.42) | 485.10 (13.79) | 693.99 (18.87) | 333.09 (10.39) | 281.76 (8.98) |
|  | Donor administration costs | 217.60 (7.91) | 206.61 (7.10) | 229.47 (7.63) | 159.25 (4.80) | 166.78 (4.74) | 167.92 (4.57) | 219.04 (6.83) | 239.36 (7.63) |
|  | Energy | 44.06 (1.60) | 59.90 (2.06) | 66.80 (2.22) | 67.58 (2.04) | 64.84 (1.84) | 55.58 (1.51) | 78.66 (2.45) | 77.71 (2.48) |
|  | Debt relief | 76.34 (2.78) | 29.79 (1.02) | 7.85 (0.26) | 21.58 (0.65) | 18.47 (0.53) | 15.22 (0.41) | 5.39 (0.17) | 5.21 (0.17) |
|  | Multisector | 129.77 (4.72) | 184.08 (6.32) | 205.91 (6.85) | 228.90 (6.90) | 243.27 (6.92) | 241.50 (6.57) | 111.02 (3.46) | 135.65 (4.33) |
|  | Water and sanitation | 148.26 (5.39) | 169.45 (5.82) | 180.61 (6.01) | 232.67 (7.01) | 232.02 (6.60) | 182.76 (4.97) | 154.65 (4.82) | 148.28 (4.73) |
|  | Financial services and business support | 65.70 (2.39) | 73.65 (2.53) | 103.85 (3.45) | 130.64 (3.94) | 141.54 (4.02) | 138.55 (3.77) | 78.52 (2.45) | 116.74 (3.72) |
|  | Environmental protection | 119.10 (4.33) | 100.16 (3.44) | 108.14 (3.60) | 123.45 (3.72) | 111.14 (3.16) | 128.07 (3.48) | 102.90 (3.21) | 87.89 (2.80) |
|  | Other social services | 44.97 (1.64) | 48.81 (1.68) | 50.07 (1.67) | 49.64 (1.50) | 67.50 (1.92) | 81.12 (2.21) | 88.62 (2.76) | 68.98 (2.20) |
|  | Conflict, peace and security | 96.13 (3.50) | 94.63 (3.25) | 119.14 (3.96) | 123.14 (3.71) | 92.19 (2.62) | 82.07 (2.23) | 75.40 (2.35) | 77.44 (2.47) |
|  | General budget support | 38.41 (1.40) | 33.89 (1.16) | 49.76 (1.66) | 54.69 (1.65) | 35.29 (1.00) | 37.63 (1.02) | 28.24 (0.88) | 22.45 (0.72) |
|  | Unspecified | 98.86 (3.60) | 112.96 (3.88) | 143.30 (4.77) | 148.92 (4.49) | 178.44 (5.07) | 192.46 (5.23) | 203.79 (6.36) | 188.08 (6.00) |
|  | Industry, construction and mining | 44.13 (1.61) | 23.31 (0.80) | 22.74 (0.76) | 64.01 (1.93) | 47.96 (1.36) | 35.18 (0.96) | 62.39 (1.95) | 59.25 (1.89) |
|  | Food aid and commodity assistance | 0.91 (0.03) | 0.83 (0.03) | 7.77 (0.26) | 5.15 (0.16) | 8.86 (0.25) | 0.85 (0.02) | 7.23 (0.23) | 5.31 (0.17) |
|  | Trade policy | 22.87 (0.83) | 50.91 (1.75) | 38.08 (1.27) | 36.51 (1.10) | 35.88 (1.02) | 39.38 (1.07) | 34.73 (1.08) | 34.78 (1.11) |
|  | Tourism | 4.12 (0.15) | 0.24 (0.01) | 4.10 (0.14) | 4.60 (0.14) | 5.28 (0.15) | 4.90 (0.13) | 4.34 (0.14) | 8.03 (0.26) |
| Denmark | Health | 246.36 (9.02) | 219.61 (8.11) | 195.27 (6.82) | 229.67 (7.97) | 168.53 (5.80) | 151.91 (5.54) | 183.91 (6.91) | 208.34 (7.91) |
|  | Humanitarian aid | 243.49 (8.92) | 234.62 (8.67) | 295.05 (10.31) | 351.63 (12.21) | 288.78 (9.95) | 388.97 (14.19) | 509.99 (19.15) | 489.85 (18.60) |
|  | Government and civil society | 401.72 (14.71) | 402.77 (14.88) | 377.48 (13.19) | 338.75 (11.76) | 468.78 (16.15) | 307.58 (11.22) | 331.63 (12.45) | 305.61 (11.61) |
|  | Education | 217.09 (7.95) | 217.76 (8.05) | 163.80 (5.72) | 141.36 (4.91) | 125.82 (4.33) | 106.45 (3.88) | 126.77 (4.76) | 165.84 (6.30) |
|  | Infrastructure | 144.46 (5.29) | 163.41 (6.04) | 105.93 (3.70) | 74.54 (2.59) | 66.78 (2.30) | 78.25 (2.85) | 61.04 (2.29) | 85.60 (3.25) |
|  | Agriculture | 196.77 (7.21) | 189.46 (7.00) | 160.85 (5.62) | 155.99 (5.42) | 144.62 (4.98) | 170.29 (6.21) | 152.24 (5.72) | 152.35 (5.79) |
|  | Refugees in donor country | 110.29 (4.04) | 137.63 (5.09) | 149.72 (5.23) | 234.57 (8.14) | 432.38 (14.89) | 447.49 (16.33) | 123.51 (4.64) | 67.17 (2.55) |
|  | Donor administration costs | 185.32 (6.79) | 185.07 (6.84) | 178.01 (6.22) | 194.68 (6.76) | 174.40 (6.01) | 167.07 (6.10) | 168.25 (6.32) | 168.84 (6.41) |
|  | Energy | 96.44 (3.53) | 62.83 (2.32) | 113.34 (3.96) | 86.87 (3.02) | 71.83 (2.47) | 69.10 (2.52) | 94.42 (3.55) | 128.22 (4.87) |
|  | Debt relief | 15.14 (0.55) | 24.09 (0.89) | 64.97 (2.27) | 0.41 (0.01) | 7.20 (0.25) | 4.86 (0.18) | 2.70 (0.10) | 3.22 (0.12) |
|  | Multisector | 55.46 (2.03) | 46.76 (1.73) | 44.56 (1.56) | 67.41 (2.34) | 63.71 (2.19) | 50.05 (1.83) | 75.67 (2.84) | 64.39 (2.45) |
|  | Water and sanitation | 108.65 (3.98) | 83.33 (3.08) | 99.41 (3.47) | 134.20 (4.66) | 103.83 (3.58) | 105.84 (3.86) | 62.74 (2.36) | 65.82 (2.50) |
|  | Financial services and business support | 100.76 (3.69) | 101.28 (3.74) | 98.25 (3.43) | 92.45 (3.21) | 130.27 (4.49) | 130.42 (4.76) | 86.96 (3.27) | 96.60 (3.67) |
|  | Environmental protection | 162.96 (5.97) | 130.49 (4.82) | 157.45 (5.50) | 146.50 (5.09) | 114.91 (3.96) | 121.77 (4.44) | 91.82 (3.45) | 106.62 (4.05) |
|  | Other social services | 53.89 (1.97) | 74.28 (2.74) | 67.13 (2.35) | 60.05 (2.09) | 64.32 (2.22) | 57.07 (2.08) | 38.43 (1.44) | 78.81 (2.99) |
|  | Conflict, peace and security | 61.91 (2.27) | 62.85 (2.32) | 60.11 (2.10) | 73.71 (2.56) | 64.10 (2.21) | 63.69 (2.32) | 156.96 (5.89) | 123.17 (4.68) |
|  | General budget support | 80.79 (2.96) | 84.94 (3.14) | 81.58 (2.85) | 71.44 (2.48) | 40.22 (1.39) | 26.35 (0.96) | 23.68 (0.89) | 23.10 (0.88) |
|  | Unspecified | 116.40 (4.26) | 114.11 (4.22) | 316.83 (11.07) | 258.82 (8.99) | 215.42 (7.42) | 160.30 (5.85) | 221.53 (8.32) | 170.17 (6.46) |
|  | Industry, construction and mining | 60.77 (2.23) | 75.71 (2.80) | 46.51 (1.62) | 61.75 (2.14) | 48.50 (1.67) | 25.55 (0.93) | 38.26 (1.44) | 39.20 (1.49) |
|  | Food aid and commodity assistance | 6.14 (0.22) | 10.39 (0.38) | 5.04 (0.18) | 10.99 (0.38) | 8.85 (0.30) | 5.89 (0.22) | 12.29 (0.46) | 28.75 (1.09) |
|  | Trade policy | 7.81 (0.29) | 15.90 (0.59) | 25.67 (0.90) | 10.29 (0.36) | 9.53 (0.33) | 12.35 (0.45) | 18.35 (0.69) | 10.94 (0.42) |
|  | Tourism | 1.12 (0.04) | 2.52 (0.09) | 0.59 (0.02) | 1.00 (0.03) | 7.03 (0.24) | 3.07 (0.11) | 0.77 (0.03) | 0.64 (0.02) |
| Belgium | Health | 254.87 (8.84) | 236.28 (10.02) | 233.43 (10.37) | 250.15 (10.59) | 218.00 (9.87) | 211.28 (8.16) | 219.51 (9.24) | 192.14 (8.13) |
|  | Humanitarian aid | 226.32 (7.85) | 132.55 (5.62) | 200.07 (8.88) | 160.30 (6.79) | 228.08 (10.33) | 361.82 (13.98) | 286.82 (12.08) | 287.15 (12.15) |
|  | Government and civil society | 219.26 (7.61) | 169.04 (7.17) | 149.23 (6.63) | 180.96 (7.66) | 149.86 (6.78) | 162.64 (6.28) | 165.46 (6.97) | 192.78 (8.16) |
|  | Education | 262.03 (9.09) | 251.11 (10.65) | 153.58 (6.82) | 148.28 (6.28) | 125.02 (5.66) | 142.26 (5.50) | 167.54 (7.06) | 175.70 (7.44) |
|  | Infrastructure | 147.06 (5.10) | 123.29 (5.23) | 156.97 (6.97) | 123.03 (5.21) | 106.47 (4.82) | 107.62 (4.16) | 103.79 (4.37) | 113.44 (4.80) |
|  | Agriculture | 216.38 (7.51) | 189.04 (8.01) | 213.83 (9.50) | 210.15 (8.90) | 184.08 (8.33) | 205.62 (7.95) | 180.66 (7.61) | 189.66 (8.03) |
|  | Refugees in donor country | 119.37 (4.14) | 125.74 (5.33) | 149.68 (6.65) | 177.60 (7.52) | 255.03 (11.55) | 414.48 (16.02) | 335.99 (14.15) | 244.50 (10.35) |
|  | Donor administration costs | 124.67 (4.33) | 122.12 (5.18) | 117.75 (5.23) | 121.05 (5.13) | 110.31 (4.99) | 108.10 (4.18) | 115.39 (4.86) | 124.44 (5.27) |
|  | Energy | 116.10 (4.03) | 82.65 (3.50) | 86.50 (3.84) | 79.34 (3.36) | 56.86 (2.57) | 76.90 (2.97) | 93.48 (3.94) | 101.28 (4.29) |
|  | Debt relief | 293.87 (10.20) | 282.89 (11.99) | 22.04 (0.98) | 12.06 (0.51) | 8.64 (0.39) | 8.56 (0.33) | 19.04 (0.80) | 9.55 (0.40) |
|  | Multisector | 101.28 (3.51) | 89.83 (3.81) | 198.73 (8.83) | 187.03 (7.92) | 164.29 (7.44) | 184.43 (7.13) | 167.96 (7.07) | 157.41 (6.66) |
|  | Water and sanitation | 94.45 (3.28) | 79.42 (3.37) | 69.73 (3.10) | 91.11 (3.86) | 66.19 (3.00) | 86.75 (3.35) | 68.06 (2.87) | 82.31 (3.48) |
|  | Financial services and business support | 223.11 (7.74) | 74.03 (3.14) | 69.80 (3.10) | 142.80 (6.05) | 127.59 (5.78) | 121.83 (4.71) | 105.59 (4.45) | 118.86 (5.03) |
|  | Environmental protection | 58.15 (2.02) | 55.60 (2.36) | 67.88 (3.01) | 57.32 (2.43) | 51.17 (2.32) | 77.01 (2.98) | 81.81 (3.45) | 90.06 (3.81) |
|  | Other social services | 69.43 (2.41) | 74.10 (3.14) | 79.93 (3.55) | 71.50 (3.03) | 58.65 (2.66) | 55.83 (2.16) | 49.98 (2.10) | 59.29 (2.51) |
|  | Conflict, peace and security | 50.48 (1.75) | 30.75 (1.30) | 25.52 (1.13) | 24.86 (1.05) | 24.78 (1.12) | 32.79 (1.27) | 30.36 (1.28) | 37.71 (1.60) |
|  | General budget support | 48.19 (1.67) | 26.41 (1.12) | 33.75 (1.50) | 51.56 (2.18) | 65.77 (2.98) | 24.87 (0.96) | 23.55 (0.99) | 18.38 (0.78) |
|  | Unspecified | 82.12 (2.85) | 78.00 (3.31) | 99.33 (4.41) | 88.43 (3.74) | 94.71 (4.29) | 81.46 (3.15) | 54.26 (2.28) | 64.98 (2.75) |
|  | Industry, construction and mining | 55.84 (1.94) | 45.36 (1.92) | 22.54 (1.00) | 16.96 (0.72) | 22.78 (1.03) | 17.72 (0.68) | 35.85 (1.51) | 28.65 (1.21) |
|  | Food aid and commodity assistance | 18.37 (0.64) | 7.80 (0.33) | 7.24 (0.32) | 5.52 (0.23) | 8.30 (0.38) | 8.22 (0.32) | 8.37 (0.35) | 11.12 (0.47) |
|  | Trade policy | 20.77 (0.72) | 14.01 (0.59) | 17.13 (0.76) | 15.60 (0.66) | 8.72 (0.39) | 13.56 (0.52) | 8.56 (0.36) | 11.95 (0.51) |
|  | Tourism | 1.05 (0.04) | 0.99 (0.04) | 1.39 (0.06) | 1.00 (0.04) | 0.50 (0.02) | 1.06 (0.04) | 1.43 (0.06) | 1.15 (0.05) |
| South Korea | Health | 148.68 (9.67) | 192.61 (10.38) | 213.65 (10.81) | 210.40 (10.50) | 223.32 (10.39) | 262.80 (10.45) | 258.28 (10.93) | 243.58 (9.61) |
|  | Humanitarian aid | 40.72 (2.65) | 26.62 (1.43) | 36.46 (1.85) | 84.57 (4.22) | 67.97 (3.16) | 84.20 (3.35) | 118.91 (5.03) | 154.79 (6.11) |
|  | Government and civil society | 90.92 (5.91) | 131.82 (7.10) | 169.24 (8.57) | 120.95 (6.03) | 134.36 (6.25) | 134.58 (5.35) | 124.05 (5.25) | 141.10 (5.57) |
|  | Education | 235.83 (15.34) | 268.59 (14.47) | 260.15 (13.17) | 266.53 (13.29) | 281.21 (13.08) | 294.78 (11.73) | 253.63 (10.73) | 253.78 (10.02) |
|  | Infrastructure | 365.62 (23.78) | 376.05 (20.26) | 385.21 (19.50) | 348.58 (17.39) | 339.03 (15.77) | 388.10 (15.44) | 345.21 (14.61) | 558.49 (22.04) |
|  | Agriculture | 111.43 (7.25) | 136.34 (7.34) | 176.24 (8.92) | 178.50 (8.90) | 181.09 (8.43) | 179.36 (7.14) | 173.94 (7.36) | 202.91 (8.01) |
|  | Refugees in donor country | 0.00 (0.00) | 0.00 (0.00) | 0.00 (0.00) | 0.00 (0.00) | 0.00 (0.00) | 0.00 (0.00) | 0.00 (0.00) | 1.83 (0.07) |
|  | Donor administration costs | 65.80 (4.28) | 78.01 (4.20) | 68.27 (3.46) | 64.35 (3.21) | 76.47 (3.56) | 98.58 (3.92) | 86.26 (3.65) | 87.38 (3.45) |
|  | Energy | 61.77 (4.02) | 90.72 (4.89) | 150.73 (7.63) | 152.64 (7.61) | 227.82 (10.60) | 140.67 (5.60) | 161.92 (6.85) | 112.30 (4.43) |
|  | Debt relief | 0.04 (0.00) | 7.66 (0.41) | 9.79 (0.50) | 6.86 (0.34) | 15.57 (0.72) | 8.12 (0.32) | 9.31 (0.39) | 8.88 (0.35) |
|  | Multisector | 57.96 (3.77) | 79.96 (4.31) | 44.38 (2.25) | 46.19 (2.30) | 47.25 (2.20) | 68.86 (2.74) | 60.83 (2.57) | 66.52 (2.63) |
|  | Water and sanitation | 105.56 (6.87) | 169.56 (9.13) | 141.82 (7.18) | 189.14 (9.43) | 174.35 (8.11) | 145.17 (5.78) | 191.84 (8.12) | 236.61 (9.34) |
|  | Financial services and business support | 14.73 (0.96) | 15.70 (0.85) | 22.02 (1.11) | 24.92 (1.24) | 25.01 (1.16) | 21.67 (0.86) | 25.95 (1.10) | 26.73 (1.05) |
|  | Environmental protection | 24.82 (1.61) | 52.86 (2.85) | 38.28 (1.94) | 25.63 (1.28) | 39.85 (1.85) | 43.33 (1.72) | 37.26 (1.58) | 69.46 (2.74) |
|  | Other social services | 34.76 (2.26) | 45.85 (2.47) | 58.01 (2.94) | 61.05 (3.05) | 62.28 (2.90) | 68.53 (2.73) | 72.06 (3.05) | 84.15 (3.32) |
|  | Conflict, peace and security | 2.97 (0.19) | 2.98 (0.16) | 2.92 (0.15) | 8.30 (0.41) | 22.28 (1.04) | 27.68 (1.10) | 39.16 (1.66) | 48.57 (1.92) |
|  | General budget support | 10.24 (0.67) | 9.08 (0.49) | 13.21 (0.67) | 7.13 (0.36) | 5.16 (0.24) | 5.63 (0.22) | 4.89 (0.21) | 3.25 (0.13) |
|  | Unspecified | 39.63 (2.58) | 29.96 (1.61) | 53.82 (2.72) | 64.12 (3.20) | 84.53 (3.93) | 103.21 (4.11) | 107.81 (4.56) | 104.54 (4.13) |
|  | Industry, construction and mining | 23.30 (1.52) | 25.37 (1.37) | 27.62 (1.40) | 28.81 (1.44) | 38.35 (1.78) | 37.78 (1.50) | 44.82 (1.90) | 28.58 (1.13) |
|  | Food aid and commodity assistance | 2.55 (0.17) | 2.53 (0.14) | 0.64 (0.03) | 1.63 (0.08) | 2.40 (0.11) | 14.67 (0.58) | 14.13 (0.60) | 14.52 (0.57) |
|  | Trade policy | 9.52 (0.62) | 11.54 (0.62) | 11.69 (0.59) | 10.75 (0.54) | 9.61 (0.45) | 12.69 (0.50) | 8.15 (0.34) | 5.90 (0.23) |
|  | Tourism | 0.81 (0.05) | 1.85 (0.10) | 1.84 (0.09) | 3.32 (0.17) | 2.54 (0.12) | 1.73 (0.07) | 2.97 (0.13) | 4.23 (0.17) |
| Austria | Health | 48.45 (4.52) | 40.56 (3.57) | 40.01 (3.50) | 43.77 (3.71) | 40.08 (2.71) | 46.38 (2.57) | 49.65 (3.71) | 39.43 (3.35) |
|  | Humanitarian aid | 53.26 (4.97) | 48.82 (4.30) | 52.25 (4.57) | 61.54 (5.22) | 64.01 (4.33) | 86.50 (4.80) | 112.05 (8.38) | 76.00 (6.46) |
|  | Government and civil society | 84.75 (7.90) | 72.27 (6.36) | 79.60 (6.97) | 67.91 (5.76) | 76.64 (5.18) | 78.23 (4.34) | 89.73 (6.71) | 104.21 (8.86) |
|  | Education | 169.15 (15.77) | 193.44 (17.03) | 173.19 (15.16) | 175.66 (14.90) | 185.00 (12.50) | 201.47 (11.18) | 211.56 (15.83) | 203.60 (17.30) |
|  | Infrastructure | 72.74 (6.78) | 87.69 (7.72) | 89.37 (7.82) | 75.49 (6.40) | 71.55 (4.84) | 79.13 (4.39) | 78.28 (5.86) | 96.12 (8.17) |
|  | Agriculture | 76.84 (7.17) | 59.00 (5.19) | 86.93 (7.61) | 72.02 (6.11) | 71.26 (4.82) | 84.63 (4.70) | 87.85 (6.57) | 80.86 (6.87) |
|  | Refugees in donor country | 40.41 (3.77) | 58.73 (5.17) | 61.75 (5.40) | 104.60 (8.87) | 488.38 (33.01) | 654.32 (36.31) | 163.20 (12.21) | 61.77 (5.25) |
|  | Donor administration costs | 49.58 (4.62) | 44.86 (3.95) | 46.98 (4.11) | 49.51 (4.20) | 48.58 (3.28) | 48.45 (2.69) | 49.92 (3.73) | 49.89 (4.24) |
|  | Energy | 65.62 (6.12) | 63.88 (5.62) | 65.91 (5.77) | 66.51 (5.64) | 64.60 (4.37) | 69.93 (3.88) | 88.18 (6.60) | 77.33 (6.57) |
|  | Debt relief | 45.00 (4.20) | 122.15 (10.76) | 47.20 (4.13) | 105.71 (8.97) | 9.52 (0.64) | 11.06 (0.61) | 12.55 (0.94) | 14.63 (1.24) |
|  | Multisector | 33.69 (3.14) | 27.20 (2.39) | 44.49 (3.89) | 45.16 (3.83) | 39.08 (2.64) | 55.73 (3.09) | 53.28 (3.99) | 58.65 (4.99) |
|  | Water and sanitation | 45.62 (4.25) | 52.38 (4.61) | 49.40 (4.32) | 55.13 (4.68) | 51.95 (3.51) | 52.43 (2.91) | 41.42 (3.10) | 59.91 (5.09) |
|  | Financial services and business support | 53.05 (4.95) | 57.04 (5.02) | 52.62 (4.61) | 63.92 (5.42) | 61.71 (4.17) | 59.19 (3.28) | 45.47 (3.40) | 50.62 (4.30) |
|  | Environmental protection | 36.96 (3.45) | 24.03 (2.12) | 25.44 (2.23) | 17.82 (1.51) | 37.96 (2.57) | 31.77 (1.76) | 31.22 (2.34) | 14.89 (1.27) |
|  | Other social services | 32.79 (3.06) | 34.23 (3.01) | 37.78 (3.31) | 41.16 (3.49) | 40.05 (2.71) | 49.32 (2.74) | 57.66 (4.31) | 50.88 (4.32) |
|  | Conflict, peace and security | 21.97 (2.05) | 17.01 (1.50) | 22.87 (2.00) | 20.22 (1.72) | 14.69 (0.99) | 16.17 (0.90) | 20.90 (1.56) | 20.02 (1.70) |
|  | General budget support | 31.45 (2.93) | 26.24 (2.31) | 27.10 (2.37) | 26.91 (2.28) | 19.06 (1.29) | 15.84 (0.88) | 14.63 (1.09) | 11.08 (0.94) |
|  | Unspecified | 42.89 (4.00) | 38.50 (3.39) | 26.73 (2.34) | 36.57 (3.10) | 42.27 (2.86) | 59.46 (3.30) | 33.90 (2.54) | 32.99 (2.80) |
|  | Industry, construction and mining | 28.16 (2.63) | 29.59 (2.61) | 33.23 (2.91) | 18.90 (1.60) | 18.41 (1.24) | 15.42 (0.86) | 36.62 (2.74) | 33.95 (2.89) |
|  | Food aid and commodity assistance | 5.72 (0.53) | 5.19 (0.46) | 28.76 (2.52) | 3.79 (0.32) | 5.47 (0.37) | 4.65 (0.26) | 4.45 (0.33) | 7.47 (0.64) |
|  | Trade policy | 5.95 (0.55) | 4.79 (0.42) | 6.61 (0.58) | 5.77 (0.49) | 5.48 (0.37) | 8.52 (0.47) | 5.30 (0.40) | 5.97 (0.51) |
|  | Tourism | 2.48 (0.23) | 1.86 (0.16) | 2.59 (0.23) | 1.51 (0.13) | 1.53 (0.10) | 2.08 (0.12) | 2.69 (0.20) | 2.28 (0.19) |
| Finland | Health | 109.70 (8.04) | 109.71 (8.24) | 109.13 (8.03) | 145.75 (9.56) | 95.86 (6.68) | 54.40 (4.67) | 57.89 (4.95) | 51.18 (5.11) |
|  | Humanitarian aid | 153.69 (11.27) | 137.00 (10.29) | 145.12 (10.67) | 161.93 (10.62) | 151.72 (10.57) | 143.46 (12.32) | 138.62 (11.85) | 121.30 (12.10) |
|  | Government and civil society | 127.89 (9.38) | 105.39 (7.91) | 119.29 (8.77) | 136.49 (8.96) | 131.49 (9.16) | 89.73 (7.71) | 110.72 (9.46) | 104.58 (10.43) |
|  | Education | 80.51 (5.90) | 80.12 (6.02) | 76.19 (5.60) | 97.88 (6.42) | 101.40 (7.06) | 76.96 (6.61) | 75.83 (6.48) | 75.88 (7.57) |
|  | Infrastructure | 61.51 (4.51) | 74.03 (5.56) | 65.89 (4.85) | 71.63 (4.70) | 60.26 (4.20) | 48.31 (4.15) | 56.87 (4.86) | 74.12 (7.39) |
|  | Agriculture | 125.16 (9.18) | 113.33 (8.51) | 114.28 (8.40) | 138.73 (9.10) | 123.43 (8.60) | 103.83 (8.92) | 88.21 (7.54) | 61.32 (6.12) |
|  | Refugees in donor country | 33.61 (2.46) | 22.92 (1.72) | 19.96 (1.47) | 15.27 (1.00) | 42.83 (2.98) | 142.98 (12.28) | 82.80 (7.08) | 56.96 (5.68) |
|  | Donor administration costs | 106.03 (7.78) | 99.55 (7.48) | 105.11 (7.73) | 95.03 (6.24) | 76.81 (5.35) | 66.56 (5.72) | 62.60 (5.35) | 61.46 (6.13) |
|  | Energy | 55.28 (4.05) | 60.75 (4.56) | 59.57 (4.38) | 95.41 (6.26) | 69.37 (4.83) | 47.59 (4.09) | 132.30 (11.31) | 54.46 (5.43) |
|  | Debt relief | 3.38 (0.25) | 12.97 (0.97) | 3.19 (0.23) | 2.27 (0.15) | 7.87 (0.55) | 2.23 (0.19) | 1.93 (0.17) | 1.82 (0.18) |
|  | Multisector | 64.81 (4.75) | 63.82 (4.79) | 58.32 (4.29) | 58.74 (3.85) | 48.41 (3.37) | 43.92 (3.77) | 45.28 (3.87) | 38.34 (3.82) |
|  | Water and sanitation | 51.22 (3.76) | 75.01 (5.63) | 77.04 (5.67) | 77.40 (5.08) | 71.60 (4.99) | 54.83 (4.71) | 42.62 (3.64) | 39.88 (3.98) |
|  | Financial services and business support | 51.06 (3.74) | 47.73 (3.58) | 52.28 (3.84) | 53.78 (3.53) | 61.35 (4.27) | 47.60 (4.09) | 41.67 (3.56) | 38.90 (3.88) |
|  | Environmental protection | 57.00 (4.18) | 68.95 (5.18) | 59.98 (4.41) | 56.79 (3.73) | 56.14 (3.91) | 25.18 (2.16) | 19.26 (1.65) | 20.81 (2.08) |
|  | Other social services | 46.32 (3.40) | 44.50 (3.34) | 52.91 (3.89) | 45.07 (2.96) | 56.91 (3.96) | 37.72 (3.24) | 40.66 (3.47) | 37.51 (3.74) |
|  | Conflict, peace and security | 52.21 (3.83) | 43.64 (3.28) | 49.81 (3.66) | 54.87 (3.60) | 57.63 (4.01) | 41.76 (3.59) | 38.40 (3.28) | 35.89 (3.58) |
|  | General budget support | 63.03 (4.62) | 39.40 (2.96) | 49.16 (3.62) | 48.05 (3.15) | 18.58 (1.29) | 10.19 (0.88) | 10.97 (0.94) | 7.08 (0.71) |
|  | Unspecified | 37.12 (2.72) | 64.41 (4.84) | 57.77 (4.25) | 63.52 (4.17) | 69.15 (4.82) | 43.22 (3.71) | 45.89 (3.92) | 48.10 (4.80) |
|  | Industry, construction and mining | 20.98 (1.54) | 23.58 (1.77) | 18.87 (1.39) | 13.84 (0.91) | 20.36 (1.42) | 17.60 (1.51) | 18.36 (1.57) | 16.25 (1.62) |
|  | Food aid and commodity assistance | 3.81 (0.28) | 3.95 (0.30) | 3.22 (0.24) | 4.99 (0.33) | 7.41 (0.52) | 3.59 (0.31) | 3.28 (0.28) | 4.79 (0.48) |
|  | Trade policy | 13.46 (0.99) | 7.19 (0.54) | 9.93 (0.73) | 16.77 (1.10) | 12.92 (0.90) | 17.80 (1.53) | 7.96 (0.68) | 10.61 (1.06) |
|  | Tourism | 2.84 (0.21) | 2.27 (0.17) | 0.72 (0.05) | 1.10 (0.07) | 7.12 (0.50) | 3.20 (0.27) | 2.64 (0.23) | 6.35 (0.63) |
| Ireland | Health | 131.36 (14.96) | 125.93 (15.31) | 136.46 (16.57) | 134.05 (16.85) | 112.03 (14.42) | 113.18 (12.96) | 113.44 (12.83) | 102.08 (10.93) |
|  | Humanitarian aid | 117.27 (13.35) | 135.53 (16.48) | 129.18 (15.68) | 132.43 (16.65) | 140.26 (18.06) | 194.22 (22.25) | 184.99 (20.92) | 197.38 (21.13) |
|  | Government and civil society | 100.10 (11.40) | 85.30 (10.37) | 94.54 (11.48) | 90.01 (11.32) | 82.90 (10.67) | 87.84 (10.06) | 98.41 (11.13) | 101.02 (10.81) |
|  | Education | 78.21 (8.91) | 59.11 (7.19) | 62.69 (7.61) | 58.95 (7.41) | 59.76 (7.69) | 60.12 (6.89) | 59.59 (6.74) | 65.71 (7.03) |
|  | Infrastructure | 24.48 (2.79) | 23.41 (2.85) | 27.82 (3.38) | 20.84 (2.62) | 22.23 (2.86) | 30.88 (3.54) | 27.73 (3.14) | 34.51 (3.69) |
|  | Agriculture | 74.86 (8.52) | 74.41 (9.05) | 77.08 (9.36) | 72.87 (9.16) | 58.68 (7.56) | 76.62 (8.78) | 68.87 (7.79) | 60.30 (6.45) |
|  | Refugees in donor country | 0.28 (0.03) | 0.13 (0.02) | 0.14 (0.02) | 0.54 (0.07) | 0.68 (0.09) | 1.16 (0.13) | 42.94 (4.86) | 56.37 (6.03) |
|  | Donor administration costs | 50.80 (5.78) | 47.64 (5.79) | 45.06 (5.47) | 44.20 (5.56) | 42.76 (5.51) | 44.57 (5.11) | 44.74 (5.06) | 46.53 (4.98) |
|  | Energy | 17.15 (1.95) | 16.08 (1.96) | 15.06 (1.83) | 16.12 (2.03) | 13.72 (1.77) | 22.62 (2.59) | 23.09 (2.61) | 22.73 (2.43) |
|  | Debt relief | 0.63 (0.07) | 0.46 (0.06) | 2.64 (0.32) | 0.18 (0.02) | 1.39 (0.18) | 0.21 (0.02) | 0.02 (0.00) | 0.06 (0.01) |
|  | Multisector | 24.73 (2.82) | 15.84 (1.93) | 16.80 (2.04) | 13.07 (1.64) | 12.57 (1.62) | 16.86 (1.93) | 24.12 (2.73) | 25.48 (2.73) |
|  | Water and sanitation | 19.48 (2.22) | 14.29 (1.74) | 14.22 (1.73) | 15.36 (1.93) | 16.70 (2.15) | 17.53 (2.01) | 16.00 (1.81) | 17.54 (1.88) |
|  | Financial services and business support | 20.02 (2.28) | 21.91 (2.66) | 20.22 (2.45) | 23.40 (2.94) | 27.53 (3.54) | 29.31 (3.36) | 21.07 (2.38) | 23.52 (2.52) |
|  | Environmental protection | 7.84 (0.89) | 9.66 (1.17) | 9.54 (1.16) | 9.34 (1.17) | 10.67 (1.37) | 14.95 (1.71) | 16.19 (1.83) | 15.14 (1.62) |
|  | Other social services | 60.48 (6.89) | 56.06 (6.82) | 40.12 (4.87) | 34.44 (4.33) | 31.34 (4.04) | 43.86 (5.02) | 39.85 (4.51) | 35.19 (3.77) |
|  | Conflict, peace and security | 15.31 (1.74) | 11.98 (1.46) | 13.50 (1.64) | 14.75 (1.85) | 16.11 (2.07) | 17.09 (1.96) | 17.36 (1.96) | 25.23 (2.70) |
|  | General budget support | 36.51 (4.16) | 31.01 (3.77) | 30.16 (3.66) | 20.86 (2.62) | 25.43 (3.27) | 7.98 (0.91) | 7.48 (0.85) | 7.01 (0.75) |
|  | Unspecified | 25.09 (2.86) | 25.65 (3.12) | 26.04 (3.16) | 34.91 (4.39) | 35.01 (4.51) | 37.15 (4.25) | 35.07 (3.97) | 48.69 (5.21) |
|  | Industry, construction and mining | 10.55 (1.20) | 11.34 (1.38) | 4.51 (0.55) | 3.62 (0.46) | 6.33 (0.81) | 6.17 (0.71) | 11.66 (1.32) | 10.13 (1.08) |
|  | Food aid and commodity assistance | 23.99 (2.73) | 21.15 (2.57) | 23.89 (2.90) | 21.20 (2.67) | 22.69 (2.92) | 7.43 (0.85) | 3.47 (0.39) | 5.23 (0.56) |
|  | Trade policy | 3.02 (0.34) | 2.80 (0.34) | 3.59 (0.44) | 3.11 (0.39) | 2.87 (0.37) | 3.72 (0.43) | 3.27 (0.37) | 4.73 (0.51) |
|  | Tourism | 0.21 (0.02) | 0.14 (0.02) | 0.25 (0.03) | 0.20 (0.03) | 0.22 (0.03) | 0.41 (0.05) | 0.48 (0.05) | 0.29 (0.03) |
| Portugal | Health | 27.92 (4.02) | 20.07 (3.21) | 26.67 (5.27) | 24.43 (5.41) | 33.50 (8.48) | 23.78 (5.51) | 20.17 (4.38) | 17.37 (3.92) |
|  | Humanitarian aid | 18.18 (2.62) | 14.69 (2.35) | 16.69 (3.30) | 22.44 (4.97) | 19.46 (4.93) | 35.27 (8.17) | 36.66 (7.96) | 28.81 (6.50) |
|  | Government and civil society | 26.88 (3.87) | 19.43 (3.11) | 19.36 (3.83) | 18.24 (4.04) | 19.12 (4.84) | 21.44 (4.97) | 32.30 (7.02) | 37.95 (8.56) |
|  | Education | 68.51 (9.86) | 61.71 (9.86) | 58.50 (11.56) | 60.69 (13.44) | 54.73 (13.86) | 64.26 (14.88) | 68.67 (14.92) | 73.03 (16.47) |
|  | Infrastructure | 53.54 (7.71) | 58.38 (9.33) | 33.15 (6.55) | 52.52 (11.63) | 23.50 (5.95) | 28.03 (6.49) | 33.42 (7.26) | 35.05 (7.90) |
|  | Agriculture | 17.70 (2.55) | 9.30 (1.49) | 11.64 (2.30) | 13.07 (2.89) | 13.47 (3.41) | 20.79 (4.81) | 23.91 (5.19) | 20.32 (4.58) |
|  | Refugees in donor country | 0.51 (0.07) | 0.83 (0.13) | 1.66 (0.33) | 1.12 (0.25) | 3.13 (0.79) | 4.77 (1.10) | 6.11 (1.33) | 9.54 (2.15) |
|  | Donor administration costs | 20.70 (2.98) | 18.27 (2.92) | 18.23 (3.60) | 14.93 (3.31) | 14.67 (3.71) | 16.10 (3.73) | 17.49 (3.80) | 17.24 (3.89) |
|  | Energy | 37.59 (5.41) | 33.74 (5.39) | 31.09 (6.14) | 20.40 (4.52) | 26.06 (6.60) | 20.67 (4.79) | 26.27 (5.71) | 20.81 (4.69) |
|  | Debt relief | 6.07 (0.87) | 8.93 (1.43) | 9.83 (1.94) | 12.00 (2.66) | 17.05 (4.32) | 20.22 (4.68) | 23.06 (5.01) | 25.68 (5.79) |
|  | Multisector | 12.26 (1.76) | 9.03 (1.44) | 9.90 (1.96) | 10.40 (2.30) | 10.74 (2.72) | 15.45 (3.58) | 22.44 (4.87) | 22.28 (5.02) |
|  | Water and sanitation | 8.57 (1.23) | 6.63 (1.06) | 5.51 (1.09) | 8.62 (1.91) | 7.26 (1.84) | 12.78 (2.96) | 19.53 (4.24) | 20.96 (4.73) |
|  | Financial services and business support | 16.06 (2.31) | 18.03 (2.88) | 17.50 (3.46) | 21.24 (4.70) | 23.88 (6.04) | 24.33 (5.63) | 18.30 (3.98) | 28.67 (6.47) |
|  | Environmental protection | 3.57 (0.51) | 3.97 (0.63) | 4.11 (0.81) | 4.04 (0.89) | 7.03 (1.78) | 7.11 (1.65) | 6.97 (1.51) | 4.73 (1.07) |
|  | Other social services | 32.01 (4.61) | 44.37 (7.09) | 70.80 (13.99) | 70.88 (15.70) | 45.85 (11.61) | 36.25 (8.40) | 31.79 (6.91) | 17.79 (4.01) |
|  | Conflict, peace and security | 18.36 (2.64) | 13.40 (2.14) | 7.70 (1.52) | 9.17 (2.03) | 10.05 (2.54) | 11.28 (2.61) | 12.82 (2.79) | 14.93 (3.37) |
|  | General budget support | 14.45 (2.08) | 12.61 (2.01) | 10.97 (2.17) | 11.65 (2.58) | 9.64 (2.44) | 8.45 (1.96) | 8.63 (1.87) | 6.52 (1.47) |
|  | Unspecified | 6.83 (0.98) | 3.22 (0.51) | 2.57 (0.51) | 1.90 (0.42) | 2.80 (0.71) | 3.60 (0.83) | 2.48 (0.54) | 1.74 (0.39) |
|  | Industry, construction and mining | 10.47 (1.51) | 12.05 (1.93) | 3.59 (0.71) | 3.29 (0.73) | 5.91 (1.50) | 3.92 (0.91) | 9.41 (2.05) | 8.03 (1.81) |
|  | Food aid and commodity assistance | 277.64 (39.96) | 239.59 (38.28) | 129.73 (25.64) | 57.63 (12.76) | 34.33 (8.69) | 34.59 (8.01) | 16.92 (3.68) | 16.05 (3.62) |
|  | Trade policy | 2.70 (0.39) | 1.57 (0.25) | 2.02 (0.40) | 2.30 (0.51) | 2.04 (0.52) | 2.07 (0.48) | 2.48 (0.54) | 2.26 (0.51) |
|  | Tourism | 0.23 (0.03) | 0.16 (0.03) | 0.20 (0.04) | 0.12 (0.03) | 0.67 (0.17) | 0.66 (0.15) | 0.95 (0.21) | 3.81 (0.86) |
| New Zealand | Health | 37.69 (9.03) | 36.41 (8.34) | 33.51 (7.92) | 29.53 (6.54) | 22.82 (4.88) | 20.26 (4.35) | 21.02 (4.72) | 25.83 (4.65) |
|  | Humanitarian aid | 34.71 (8.32) | 28.92 (6.62) | 30.02 (7.10) | 35.43 (7.85) | 31.88 (6.81) | 36.34 (7.80) | 48.05 (10.80) | 52.29 (9.40) |
|  | Government and civil society | 34.86 (8.36) | 35.18 (8.06) | 31.07 (7.34) | 31.61 (7.00) | 33.41 (7.14) | 37.80 (8.11) | 30.28 (6.81) | 43.14 (7.76) |
|  | Education | 59.87 (14.35) | 71.08 (16.28) | 75.32 (17.80) | 68.94 (15.26) | 81.71 (17.46) | 73.41 (15.76) | 74.49 (16.74) | 74.50 (13.40) |
|  | Infrastructure | 25.03 (6.00) | 32.92 (7.54) | 35.08 (8.29) | 30.04 (6.65) | 16.25 (3.47) | 11.07 (2.38) | 14.67 (3.30) | 35.15 (6.32) |
|  | Agriculture | 29.54 (7.08) | 24.51 (5.61) | 29.05 (6.87) | 34.73 (7.69) | 44.24 (9.45) | 46.49 (9.98) | 45.76 (10.28) | 52.78 (9.49) |
|  | Refugees in donor country | 13.59 (3.26) | 18.53 (4.24) | 17.94 (4.24) | 17.63 (3.90) | 17.60 (3.76) | 17.44 (3.74) | 17.13 (3.85) | 16.95 (3.05) |
|  | Donor administration costs | 46.89 (11.24) | 44.28 (10.14) | 43.26 (10.22) | 42.38 (9.38) | 44.15 (9.43) | 44.29 (9.51) | 44.81 (10.07) | 48.66 (8.75) |
|  | Energy | 13.77 (3.30) | 20.86 (4.78) | 16.50 (3.90) | 38.77 (8.58) | 29.80 (6.37) | 24.94 (5.36) | 23.65 (5.31) | 33.09 (5.95) |
|  | Debt relief | 0.01 (0.00) | 0.00 (0.00) | 0.25 (0.06) | 0.03 (0.01) | 0.67 (0.14) | 0.05 (0.01) | 0.03 (0.01) | 0.03 (0.01) |
|  | Multisector | 12.71 (3.05) | 16.34 (3.74) | 11.15 (2.64) | 11.31 (2.50) | 14.08 (3.01) | 16.46 (3.53) | 19.22 (4.32) | 34.11 (6.13) |
|  | Water and sanitation | 8.45 (2.03) | 8.58 (1.96) | 10.08 (2.38) | 10.39 (2.30) | 11.50 (2.46) | 10.76 (2.31) | 9.29 (2.09) | 14.23 (2.56) |
|  | Financial services and business support | 7.94 (1.90) | 8.52 (1.95) | 12.71 (3.00) | 11.78 (2.61) | 9.27 (1.98) | 14.60 (3.14) | 9.20 (2.07) | 25.35 (4.56) |
|  | Environmental protection | 3.27 (0.78) | 2.20 (0.50) | 5.10 (1.21) | 3.64 (0.81) | 11.37 (2.43) | 4.08 (0.87) | 5.09 (1.14) | 5.84 (1.05) |
|  | Other social services | 7.80 (1.87) | 4.73 (1.08) | 6.99 (1.65) | 7.57 (1.68) | 9.81 (2.10) | 9.15 (1.97) | 7.95 (1.79) | 7.98 (1.44) |
|  | Conflict, peace and security | 11.79 (2.83) | 11.23 (2.57) | 6.98 (1.65) | 10.25 (2.27) | 10.28 (2.20) | 11.21 (2.41) | 11.44 (2.57) | 2.71 (0.49) |
|  | General budget support | 15.86 (3.80) | 17.23 (3.95) | 14.32 (3.38) | 14.77 (3.27) | 14.93 (3.19) | 27.66 (5.94) | 15.31 (3.44) | 28.04 (5.04) |
|  | Unspecified | 3.96 (0.95) | 1.07 (0.24) | 1.32 (0.31) | 1.52 (0.34) | 3.01 (0.64) | 4.53 (0.97) | 4.14 (0.93) | 3.87 (0.70) |
|  | Industry, construction and mining | 4.62 (1.11) | 2.37 (0.54) | 1.93 (0.46) | 3.15 (0.70) | 3.53 (0.75) | 1.23 (0.26) | 0.42 (0.10) | 0.98 (0.18) |
|  | Food aid and commodity assistance | 0.72 (0.17) | 0.98 (0.22) | 0.23 (0.05) | 0.33 (0.07) | 0.29 (0.06) | 0.33 (0.07) | 0.15 (0.03) | 0.27 (0.05) |
|  | Trade policy | 3.43 (0.82) | 4.32 (0.99) | 3.31 (0.78) | 5.08 (1.12) | 4.66 (0.99) | 2.76 (0.59) | 2.65 (0.60) | 3.84 (0.69) |
|  | Tourism | 13.69 (3.28) | 9.77 (2.24) | 7.66 (1.81) | 6.33 (1.40) | 17.39 (3.72) | 13.88 (2.98) | 7.59 (1.71) | 8.43 (1.52) |
| Luxembourg | Health | 64.63 (16.33) | 64.52 (15.90) | 73.04 (17.56) | 68.78 (17.19) | 61.89 (15.25) | 65.04 (14.91) | 63.00 (13.76) | 63.12 (13.11) |
|  | Humanitarian aid | 58.59 (14.81) | 53.29 (13.13) | 55.60 (13.37) | 53.64 (13.41) | 56.21 (13.85) | 65.09 (14.92) | 69.90 (15.26) | 72.11 (14.98) |
|  | Government and civil society | 21.70 (5.48) | 23.47 (5.78) | 28.45 (6.84) | 25.38 (6.34) | 33.38 (8.23) | 37.22 (8.53) | 39.14 (8.55) | 38.49 (8.00) |
|  | Education | 36.15 (9.14) | 47.51 (11.71) | 53.51 (12.87) | 56.44 (14.11) | 54.11 (13.33) | 62.76 (14.38) | 49.43 (10.79) | 57.38 (11.92) |
|  | Infrastructure | 8.18 (2.07) | 10.01 (2.47) | 11.36 (2.73) | 10.81 (2.70) | 9.80 (2.41) | 9.18 (2.10) | 19.96 (4.36) | 14.36 (2.98) |
|  | Agriculture | 27.38 (6.92) | 28.97 (7.14) | 29.53 (7.10) | 26.54 (6.63) | 29.09 (7.17) | 34.45 (7.90) | 42.61 (9.30) | 45.14 (9.38) |
|  | Refugees in donor country | 0.00 (0.00) | 0.03 (0.01) | 0.37 (0.09) | 0.05 (0.01) | 0.00 (0.00) | 0.00 (0.00) | 0.00 (0.00) | 0.00 (0.00) |
|  | Donor administration costs | 23.16 (5.85) | 25.02 (6.16) | 25.89 (6.22) | 26.72 (6.68) | 24.59 (6.06) | 23.17 (5.31) | 24.93 (5.44) | 25.15 (5.22) |
|  | Energy | 9.84 (2.49) | 13.10 (3.23) | 9.50 (2.29) | 9.45 (2.36) | 5.43 (1.34) | 6.76 (1.55) | 8.37 (1.83) | 8.96 (1.86) |
|  | Debt relief | 0.15 (0.04) | 0.74 (0.18) | 0.22 (0.05) | 0.02 (0.00) | 2.09 (0.51) | 1.39 (0.32) | 0.03 (0.01) | 0.08 (0.02) |
|  | Multisector | 16.61 (4.20) | 20.34 (5.01) | 14.99 (3.61) | 15.91 (3.98) | 8.32 (2.05) | 11.49 (2.63) | 12.22 (2.67) | 13.63 (2.83) |
|  | Water and sanitation | 23.97 (6.06) | 19.41 (4.78) | 13.66 (3.28) | 15.78 (3.94) | 16.07 (3.96) | 10.09 (2.31) | 20.31 (4.43) | 27.36 (5.68) |
|  | Financial services and business support | 21.54 (5.44) | 21.88 (5.39) | 25.73 (6.19) | 22.56 (5.64) | 21.09 (5.20) | 27.50 (6.30) | 22.51 (4.92) | 27.61 (5.73) |
|  | Environmental protection | 4.66 (1.18) | 4.56 (1.12) | 4.91 (1.18) | 4.73 (1.18) | 4.94 (1.22) | 4.48 (1.03) | 4.57 (1.00) | 2.98 (0.62) |
|  | Other social services | 16.96 (4.29) | 15.48 (3.82) | 14.46 (3.48) | 12.40 (3.10) | 15.77 (3.89) | 16.33 (3.74) | 21.26 (4.64) | 18.74 (3.89) |
|  | Conflict, peace and security | 3.23 (0.82) | 2.45 (0.60) | 3.88 (0.93) | 3.24 (0.81) | 4.20 (1.04) | 3.74 (0.86) | 4.72 (1.03) | 6.23 (1.29) |
|  | General budget support | 3.66 (0.93) | 2.45 (0.60) | 2.00 (0.48) | 2.44 (0.61) | 1.75 (0.43) | 1.74 (0.40) | 1.73 (0.38) | 1.44 (0.30) |
|  | Unspecified | 26.93 (6.80) | 27.28 (6.72) | 28.23 (6.79) | 22.20 (5.55) | 34.31 (8.46) | 30.06 (6.89) | 30.96 (6.76) | 40.15 (8.34) |
|  | Industry, construction and mining | 3.50 (0.88) | 3.43 (0.85) | 1.69 (0.41) | 3.55 (0.89) | 1.99 (0.49) | 1.56 (0.36) | 3.74 (0.82) | 4.11 (0.85) |
|  | Food aid and commodity assistance | 3.74 (0.95) | 1.68 (0.41) | 4.31 (1.04) | 3.17 (0.79) | 4.47 (1.10) | 4.68 (1.07) | 5.23 (1.14) | 1.72 (0.36) |
|  | Trade policy | 2.35 (0.60) | 0.65 (0.16) | 0.90 (0.22) | 0.61 (0.15) | 0.58 (0.14) | 3.54 (0.81) | 0.69 (0.15) | 2.50 (0.52) |
|  | Tourism | 2.00 (0.51) | 2.16 (0.53) | 1.42 (0.34) | 1.84 (0.46) | 3.30 (0.81) | 0.34 (0.08) | 0.71 (0.16) | 0.66 (0.14) |
| Poland | Health | NA | NA | 15.73 (3.40) | 14.39 (3.35) | 18.34 (3.65) | 25.21 (3.25) | 25.39 (3.40) | 19.02 (2.42) |
|  | Humanitarian aid | NA | NA | 37.68 (8.13) | 43.17 (10.03) | 43.42 (8.65) | 94.14 (12.15) | 94.94 (12.72) | 83.69 (10.65) |
|  | Government and civil society | NA | NA | 47.05 (10.15) | 40.75 (9.47) | 52.52 (10.46) | 58.31 (7.53) | 68.23 (9.14) | 142.13 (18.08) |
|  | Education | NA | NA | 79.34 (17.12) | 55.03 (12.79) | 71.37 (14.21) | 71.50 (9.23) | 115.39 (15.45) | 121.46 (15.45) |
|  | Infrastructure | NA | NA | 46.26 (9.98) | 34.73 (8.07) | 37.54 (7.48) | 56.72 (7.32) | 45.79 (6.13) | 73.15 (9.31) |
|  | Agriculture | NA | NA | 19.90 (4.29) | 44.41 (10.32) | 54.68 (10.89) | 127.64 (16.48) | 46.13 (6.18) | 71.81 (9.14) |
|  | Refugees in donor country | NA | NA | 0.32 (0.07) | 0.47 (0.11) | 10.02 (2.00) | 6.50 (0.84) | 6.29 (0.84) | 6.25 (0.80) |
|  | Donor administration costs | NA | NA | 13.92 (3.00) | 14.41 (3.35) | 15.16 (3.02) | 18.07 (2.33) | 19.37 (2.59) | 20.92 (2.66) |
|  | Energy | NA | NA | 23.82 (5.14) | 22.86 (5.31) | 22.64 (4.51) | 41.15 (5.31) | 36.75 (4.92) | 36.54 (4.65) |
|  | Debt relief | NA | NA | 0.07 (0.01) | 0.02 (0.01) | 0.98 (0.19) | 1.10 (0.14) | 0.00 (0.00) | 0.00 (0.00) |
|  | Multisector | NA | NA | 16.04 (3.46) | 15.48 (3.60) | 14.16 (2.82) | 22.86 (2.95) | 87.27 (11.69) | 29.42 (3.74) |
|  | Water and sanitation | NA | NA | 11.63 (2.51) | 14.45 (3.36) | 15.24 (3.04) | 17.73 (2.29) | 12.87 (1.72) | 24.70 (3.14) |
|  | Financial services and business support | NA | NA | 33.80 (7.29) | 39.11 (9.09) | 46.91 (9.34) | 52.49 (6.78) | 33.11 (4.43) | 40.84 (5.20) |
|  | Environmental protection | NA | NA | 10.80 (2.33) | 5.55 (1.29) | 8.16 (1.63) | 14.25 (1.84) | 11.59 (1.55) | 9.51 (1.21) |
|  | Other social services | NA | NA | 16.30 (3.52) | 18.14 (4.22) | 22.53 (4.49) | 24.31 (3.14) | 27.28 (3.65) | 19.58 (2.49) |
|  | Conflict, peace and security | NA | NA | 11.32 (2.44) | 12.03 (2.80) | 12.98 (2.58) | 16.41 (2.12) | 18.83 (2.52) | 21.23 (2.70) |
|  | General budget support | NA | NA | 16.44 (3.55) | 20.27 (4.71) | 16.75 (3.33) | 15.95 (2.06) | 14.11 (1.89) | 13.48 (1.71) |
|  | Unspecified | NA | NA | 32.42 (7.00) | 3.54 (0.82) | 4.54 (0.90) | 5.14 (0.66) | 4.52 (0.61) | 3.90 (0.50) |
|  | Industry, construction and mining | NA | NA | 7.23 (1.56) | 6.71 (1.56) | 10.51 (2.09) | 10.09 (1.30) | 17.70 (2.37) | 17.13 (2.18) |
|  | Food aid and commodity assistance | NA | NA | 4.24 (0.92) | 2.47 (0.57) | 5.12 (1.02) | 5.25 (0.68) | 4.32 (0.58) | 7.72 (0.98) |
|  | Trade policy | NA | NA | 4.06 (0.88) | 4.46 (1.04) | 4.22 (0.84) | 5.41 (0.70) | 4.74 (0.64) | 4.53 (0.58) |
|  | Tourism | NA | NA | 0.40 (0.09) | 0.38 (0.09) | 0.48 (0.10) | 1.19 (0.15) | 1.37 (0.18) | 1.47 (0.19) |
| Greece | Health | 11.75 (3.41) | 8.19 (2.84) | 9.54 (4.55) | 7.83 (3.54) | 8.61 (3.36) | 10.41 (2.62) | 13.50 (4.09) | 8.67 (2.99) |
|  | Humanitarian aid | 21.28 (6.17) | 16.60 (5.75) | 17.29 (8.25) | 21.14 (9.55) | 19.06 (7.44) | 34.98 (8.81) | 36.50 (11.07) | 29.15 (10.04) |
|  | Government and civil society | 16.30 (4.72) | 15.38 (5.32) | 13.87 (6.62) | 14.47 (6.54) | 15.81 (6.17) | 18.35 (4.62) | 25.64 (7.78) | 32.28 (11.11) |
|  | Education | 71.88 (20.83) | 68.33 (23.66) | 17.33 (8.27) | 16.55 (7.48) | 18.19 (7.10) | 12.32 (3.10) | 15.63 (4.74) | 16.31 (5.61) |
|  | Infrastructure | 41.29 (11.97) | 25.32 (8.77) | 23.86 (11.39) | 17.87 (8.08) | 18.42 (7.19) | 24.30 (6.12) | 23.64 (7.17) | 25.83 (8.89) |
|  | Agriculture | 13.05 (3.78) | 7.04 (2.44) | 9.51 (4.54) | 11.29 (5.10) | 11.50 (4.49) | 18.65 (4.70) | 17.06 (5.17) | 18.26 (6.29) |
|  | Refugees in donor country | 20.63 (5.98) | 17.65 (6.11) | 18.89 (9.01) | 19.29 (8.72) | 63.77 (24.90) | 157.99 (39.78) | 71.58 (21.70) | 27.92 (9.61) |
|  | Donor administration costs | 19.38 (5.62) | 16.35 (5.66) | 13.12 (6.26) | 13.65 (6.17) | 7.07 (2.76) | 8.79 (2.21) | 8.17 (2.48) | 8.73 (3.01) |
|  | Energy | 19.65 (5.69) | 16.52 (5.72) | 11.70 (5.59) | 11.47 (5.19) | 9.48 (3.70) | 17.24 (4.34) | 19.15 (5.81) | 18.12 (6.24) |
|  | Debt relief | 0.88 (0.26) | 0.63 (0.22) | 0.02 (0.01) | 0.01 (0.01) | 0.00 (0.00) | 0.00 (0.00) | 0.01 (0.00) | 0.01 (0.00) |
|  | Multisector | 14.95 (4.33) | 12.26 (4.24) | 9.03 (4.31) | 10.33 (4.67) | 8.65 (3.38) | 12.04 (3.03) | 15.85 (4.81) | 14.60 (5.03) |
|  | Water and sanitation | 7.61 (2.21) | 6.50 (2.25) | 5.23 (2.50) | 6.92 (3.13) | 6.76 (2.64) | 6.66 (1.68) | 7.06 (2.14) | 8.36 (2.88) |
|  | Financial services and business support | 17.37 (5.03) | 18.82 (6.52) | 16.87 (8.05) | 20.00 (9.04) | 23.50 (9.17) | 22.91 (5.77) | 16.11 (4.88) | 18.78 (6.47) |
|  | Environmental protection | 2.93 (0.85) | 4.39 (1.52) | 3.47 (1.66) | 2.88 (1.30) | 3.27 (1.28) | 6.91 (1.74) | 6.10 (1.85) | 4.46 (1.54) |
|  | Other social services | 11.34 (3.29) | 6.17 (2.14) | 6.18 (2.95) | 6.86 (3.10) | 7.98 (3.12) | 6.95 (1.75) | 7.13 (2.16) | 7.61 (2.62) |
|  | Conflict, peace and security | 8.21 (2.38) | 4.29 (1.49) | 5.35 (2.55) | 5.94 (2.69) | 6.72 (2.62) | 7.07 (1.78) | 7.16 (2.17) | 8.89 (3.06) |
|  | General budget support | 11.97 (3.47) | 10.33 (3.58) | 8.55 (4.08) | 10.65 (4.81) | 8.89 (3.47) | 7.28 (1.83) | 6.83 (2.07) | 6.20 (2.13) |
|  | Unspecified | 6.46 (1.87) | 2.61 (0.90) | 2.91 (1.39) | 1.17 (0.53) | 1.43 (0.56) | 1.06 (0.27) | 0.76 (0.23) | 0.98 (0.34) |
|  | Industry, construction and mining | 11.70 (3.39) | 13.46 (4.66) | 3.58 (1.71) | 3.24 (1.46) | 5.14 (2.01) | 3.82 (0.96) | 8.93 (2.71) | 8.00 (2.76) |
|  | Food aid and commodity assistance | 3.33 (0.97) | 2.87 (0.99) | 2.20 (1.05) | 1.29 (0.58) | 2.70 (1.06) | 2.38 (0.60) | 2.13 (0.65) | 3.58 (1.23) |
|  | Trade policy | 3.13 (0.91) | 1.74 (0.60) | 2.09 (1.00) | 2.33 (1.05) | 2.11 (0.83) | 2.03 (0.51) | 2.37 (0.72) | 2.23 (0.77) |
|  | Tourism | 0.19 (0.05) | 0.14 (0.05) | 0.20 (0.10) | 0.12 (0.05) | 0.11 (0.04) | 0.34 (0.09) | 0.64 (0.19) | 0.63 (0.22) |
| Czech | Health | 10.22 (4.46) | 8.51 (3.89) | 10.03 (4.84) | 8.69 (4.02) | 10.02 (4.22) | 12.96 (4.26) | 17.14 (5.11) | 8.48 (2.78) |
|  | Humanitarian aid | 16.86 (7.36) | 15.95 (7.29) | 17.50 (8.44) | 23.48 (10.87) | 24.55 (10.35) | 39.06 (12.83) | 30.27 (9.02) | 38.30 (12.54) |
|  | Government and civil society | 20.17 (8.81) | 15.61 (7.14) | 17.02 (8.21) | 17.56 (8.13) | 20.25 (8.54) | 24.08 (7.91) | 32.80 (9.77) | 39.94 (13.08) |
|  | Education | 15.45 (6.75) | 15.64 (7.15) | 15.11 (7.29) | 16.73 (7.75) | 18.60 (7.84) | 20.38 (6.69) | 21.08 (6.28) | 20.14 (6.60) |
|  | Infrastructure | 18.15 (7.93) | 19.21 (8.78) | 18.74 (9.04) | 15.35 (7.11) | 14.85 (6.26) | 23.49 (7.72) | 21.36 (6.36) | 21.69 (7.10) |
|  | Agriculture | 15.11 (6.60) | 11.41 (5.22) | 11.45 (5.53) | 15.30 (7.09) | 15.47 (6.52) | 23.47 (7.71) | 21.78 (6.49) | 21.99 (7.20) |
|  | Refugees in donor country | 10.71 (4.68) | 9.49 (4.34) | 8.91 (4.30) | 11.96 (5.54) | 16.82 (7.09) | 21.07 (6.92) | 23.98 (7.15) | 24.99 (8.18) |
|  | Donor administration costs | 11.45 (5.00) | 9.83 (4.49) | 9.73 (4.70) | 10.36 (4.80) | 10.22 (4.31) | 11.87 (3.90) | 11.36 (3.39) | 12.43 (4.07) |
|  | Energy | 16.84 (7.35) | 16.23 (7.42) | 12.49 (6.03) | 11.89 (5.51) | 13.54 (5.71) | 18.46 (6.06) | 18.93 (5.64) | 16.35 (5.35) |
|  | Debt relief | 0.55 (0.24) | 0.62 (0.28) | 0.08 (0.04) | 0.01 (0.01) | 0.33 (0.14) | 0.00 (0.00) | 0.00 (0.00) | 0.00 (0.00) |
|  | Multisector | 7.16 (3.13) | 5.85 (2.67) | 5.95 (2.87) | 7.61 (3.52) | 10.59 (4.46) | 14.08 (4.62) | 13.61 (4.05) | 11.51 (3.77) |
|  | Water and sanitation | 12.98 (5.67) | 12.69 (5.80) | 9.42 (4.54) | 10.21 (4.73) | 9.74 (4.11) | 10.85 (3.56) | 9.11 (2.71) | 10.60 (3.47) |
|  | Financial services and business support | 12.12 (5.30) | 13.49 (6.17) | 13.49 (6.51) | 16.69 (7.73) | 19.03 (8.02) | 21.99 (7.22) | 15.56 (4.64) | 17.53 (5.74) |
|  | Environmental protection | 2.49 (1.09) | 3.86 (1.76) | 3.57 (1.72) | 4.67 (2.16) | 5.32 (2.24) | 8.32 (2.73) | 6.90 (2.05) | 5.05 (1.66) |
|  | Other social services | 8.45 (3.69) | 7.97 (3.64) | 8.03 (3.88) | 8.15 (3.77) | 9.85 (4.15) | 10.22 (3.36) | 14.42 (4.30) | 7.53 (2.47) |
|  | Conflict, peace and security | 11.77 (5.14) | 12.15 (5.56) | 5.56 (2.68) | 7.67 (3.55) | 7.71 (3.25) | 9.13 (3.00) | 7.88 (2.35) | 8.80 (2.88) |
|  | General budget support | 7.52 (3.29) | 11.12 (5.09) | 17.24 (8.32) | 8.53 (3.95) | 6.68 (2.81) | 6.75 (2.22) | 6.46 (1.93) | 5.32 (1.74) |
|  | Unspecified | 5.76 (2.52) | 4.28 (1.96) | 4.08 (1.97) | 3.19 (1.47) | 4.37 (1.84) | 4.17 (1.37) | 6.83 (2.03) | 13.25 (4.34) |
|  | Industry, construction and mining | 8.49 (3.71) | 10.00 (4.57) | 4.13 (1.99) | 3.44 (1.59) | 4.39 (1.85) | 3.76 (1.23) | 8.50 (2.53) | 6.86 (2.25) |
|  | Food aid and commodity assistance | 2.14 (0.93) | 1.93 (0.88) | 1.65 (0.80) | 1.05 (0.49) | 2.05 (0.86) | 2.40 (0.79) | 2.21 (0.66) | 3.13 (1.03) |
|  | Trade policy | 2.08 (0.91) | 1.46 (0.67) | 1.69 (0.82) | 1.93 (0.89) | 1.72 (0.73) | 2.16 (0.71) | 2.19 (0.65) | 1.86 (0.61) |
|  | Tourism | 0.19 (0.08) | 0.14 (0.07) | 0.20 (0.10) | 0.15 (0.07) | 0.13 (0.06) | 0.51 (0.17) | 0.40 (0.12) | 0.36 (0.12) |
| Hungary | Health | NA | NA | NA | 5.54 (3.99) | 7.56 (4.31) | 7.69 (3.43) | 6.39 (4.05) | 14.09 (4.95) |
|  | Humanitarian aid | NA | NA | NA | 11.95 (8.60) | 12.97 (7.38) | 17.60 (7.85) | 10.49 (6.65) | 21.98 (7.71) |
|  | Government and civil society | NA | NA | NA | 9.61 (6.92) | 11.90 (6.77) | 13.60 (6.06) | 11.54 (7.31) | 20.27 (7.11) |
|  | Education | NA | NA | NA | 12.50 (9.00) | 27.55 (15.69) | 35.65 (15.89) | 32.87 (20.83) | 71.10 (24.95) |
|  | Infrastructure | NA | NA | NA | 11.53 (8.30) | 12.52 (7.13) | 16.77 (7.48) | 10.32 (6.54) | 17.32 (6.08) |
|  | Agriculture | NA | NA | NA | 8.43 (6.07) | 9.02 (5.14) | 17.89 (7.98) | 9.64 (6.11) | 22.01 (7.72) |
|  | Refugees in donor country | NA | NA | NA | 10.18 (7.33) | 10.94 (6.23) | 11.06 (4.93) | 3.54 (2.25) | 3.65 (1.28) |
|  | Donor administration costs | NA | NA | NA | 5.92 (4.26) | 5.48 (3.12) | 5.13 (2.29) | 4.28 (2.71) | 5.81 (2.04) |
|  | Energy | NA | NA | NA | 8.12 (5.84) | 7.24 (4.12) | 12.13 (5.41) | 8.33 (5.28) | 11.40 (4.00) |
|  | Debt relief | NA | NA | NA | 0.02 (0.01) | 0.74 (0.42) | 0.00 (0.00) | 0.00 (0.00) | 0.00 (0.00) |
|  | Multisector | NA | NA | NA | 4.33 (3.12) | 5.84 (3.33) | 7.33 (3.27) | 6.50 (4.12) | 7.88 (2.76) |
|  | Water and sanitation | NA | NA | NA | 6.00 (4.32) | 10.50 (5.98) | 8.47 (3.78) | 5.29 (3.35) | 22.99 (8.07) |
|  | Financial services and business support | NA | NA | NA | 17.73 (12.77) | 14.48 (8.24) | 14.96 (6.67) | 7.24 (4.59) | 10.77 (3.78) |
|  | Environmental protection | NA | NA | NA | 1.73 (1.24) | 2.58 (1.47) | 4.09 (1.82) | 2.79 (1.77) | 2.91 (1.02) |
|  | Other social services | NA | NA | NA | 4.95 (3.56) | 6.17 (3.51) | 6.13 (2.73) | 2.77 (1.75) | 16.51 (5.79) |
|  | Conflict, peace and security | NA | NA | NA | 3.19 (2.30) | 3.37 (1.92) | 4.57 (2.04) | 3.46 (2.20) | 5.14 (1.81) |
|  | General budget support | NA | NA | NA | 5.95 (4.28) | 5.00 (2.85) | 4.64 (2.07) | 3.18 (2.02) | 3.58 (1.26) |
|  | Unspecified | NA | NA | NA | 0.62 (0.44) | 10.02 (5.70) | 15.34 (6.84) | 3.88 (2.46) | 4.11 (1.44) |
|  | Industry, construction and mining | NA | NA | NA | 2.02 (1.45) | 3.36 (1.91) | 2.65 (1.18) | 3.92 (2.48) | 4.59 (1.61) |
|  | Food aid and commodity assistance | NA | NA | NA | 0.74 (0.54) | 1.56 (0.89) | 1.51 (0.67) | 2.14 (1.36) | 2.07 (0.72) |
|  | Trade policy | NA | NA | NA | 1.39 (1.00) | 1.37 (0.78) | 1.69 (0.75) | 1.05 (0.66) | 1.28 (0.45) |
|  | Tourism | NA | NA | NA | 0.08 (0.06) | 0.11 (0.06) | 0.37 (0.16) | 0.32 (0.20) | 0.11 (0.04) |
| Slovak Republic | Health | NA | NA | 3.24 (4.13) | 3.00 (3.95) | 3.98 (4.29) | 4.52 (3.86) | 6.76 (5.30) | 5.08 (3.69) |
|  | Humanitarian aid | NA | NA | 6.34 (8.11) | 7.80 (10.28) | 9.73 (10.48) | 12.72 (10.89) | 10.47 (8.22) | 9.15 (6.64) |
|  | Government and civil society | NA | NA | 6.39 (8.16) | 6.56 (8.64) | 9.49 (10.22) | 8.85 (7.57) | 12.87 (10.10) | 16.52 (12.00) |
|  | Education | NA | NA | 7.26 (9.27) | 6.51 (8.57) | 8.19 (8.81) | 9.49 (8.12) | 9.29 (7.30) | 9.45 (6.86) |
|  | Infrastructure | NA | NA | 8.33 (10.65) | 6.33 (8.35) | 7.55 (8.13) | 9.15 (7.83) | 8.53 (6.69) | 9.91 (7.19) |
|  | Agriculture | NA | NA | 5.32 (6.80) | 4.61 (6.08) | 5.82 (6.27) | 7.69 (6.58) | 6.24 (4.90) | 7.24 (5.25) |
|  | Refugees in donor country | NA | NA | 0.93 (1.19) | 0.98 (1.30) | 1.90 (2.04) | 1.74 (1.49) | 1.42 (1.11) | 0.79 (0.58) |
|  | Donor administration costs | NA | NA | 4.54 (5.80) | 4.52 (5.96) | 4.35 (4.69) | 4.84 (4.14) | 6.43 (5.05) | 5.52 (4.01) |
|  | Energy | NA | NA | 4.26 (5.44) | 4.15 (5.46) | 4.42 (4.76) | 6.78 (5.80) | 6.96 (5.46) | 7.12 (5.17) |
|  | Debt relief | NA | NA | 0.02 (0.02) | 0.00 (0.01) | 0.42 (0.45) | 0.00 (0.00) | 0.00 (0.00) | 0.00 (0.00) |
|  | Multisector | NA | NA | 2.80 (3.57) | 2.59 (3.41) | 3.87 (4.16) | 5.27 (4.51) | 5.06 (3.97) | 4.83 (3.51) |
|  | Water and sanitation | NA | NA | 1.93 (2.47) | 2.53 (3.33) | 3.59 (3.87) | 3.04 (2.60) | 3.87 (3.04) | 4.19 (3.04) |
|  | Financial services and business support | NA | NA | 5.98 (7.63) | 6.99 (9.21) | 8.76 (9.44) | 8.72 (7.46) | 6.00 (4.71) | 7.39 (5.37) |
|  | Environmental protection | NA | NA | 1.37 (1.75) | 1.05 (1.38) | 1.60 (1.72) | 2.33 (1.99) | 2.20 (1.73) | 1.75 (1.27) |
|  | Other social services | NA | NA | 2.68 (3.43) | 2.71 (3.57) | 3.94 (4.25) | 3.01 (2.57) | 2.46 (1.93) | 3.23 (2.34) |
|  | Conflict, peace and security | NA | NA | 3.21 (4.10) | 3.18 (4.19) | 3.85 (4.14) | 4.16 (3.56) | 4.49 (3.53) | 4.68 (3.40) |
|  | General budget support | NA | NA | 6.80 (8.69) | 3.66 (4.83) | 3.04 (3.27) | 2.74 (2.34) | 2.68 (2.10) | 2.39 (1.73) |
|  | Unspecified | NA | NA | 1.22 (1.56) | 3.32 (4.37) | 1.25 (1.35) | 11.76 (10.06) | 18.59 (14.59) | 17.39 (12.63) |
|  | Industry, construction and mining | NA | NA | 1.25 (1.60) | 1.14 (1.50) | 2.09 (2.25) | 1.54 (1.32) | 3.31 (2.60) | 3.16 (2.29) |
|  | Food aid and commodity assistance | NA | NA | 0.76 (0.97) | 0.47 (0.61) | 0.96 (1.03) | 0.92 (0.79) | 0.88 (0.69) | 1.49 (1.08) |
|  | Trade policy | NA | NA | 0.74 (0.95) | 0.81 (1.07) | 0.83 (0.90) | 0.77 (0.66) | 0.90 (0.70) | 0.87 (0.63) |
|  | Tourism | NA | NA | 0.07 (0.09) | 0.04 (0.05) | 0.07 (0.07) | 0.13 (0.11) | 0.17 (0.13) | 0.08 (0.06) |
| Slovenia | Health | 2.32 (4.03) | 3.24 (5.59) | 2.71 (4.66) | 1.71 (2.96) | 2.16 (3.06) | 2.78 (3.08) | 3.10 (3.82) | 1.96 (2.35) |
|  | Humanitarian aid | 4.09 (7.09) | 3.58 (6.19) | 3.91 (6.73) | 5.05 (8.73) | 6.05 (8.58) | 8.00 (8.88) | 7.29 (8.98) | 7.26 (8.69) |
|  | Government and civil society | 5.86 (10.15) | 5.48 (9.47) | 5.38 (9.25) | 5.21 (9.00) | 5.78 (8.20) | 6.32 (7.01) | 7.59 (9.36) | 8.99 (10.77) |
|  | Education | 4.57 (7.93) | 5.83 (10.07) | 6.53 (11.22) | 7.80 (13.48) | 8.81 (12.51) | 10.81 (11.99) | 13.79 (17.00) | 14.63 (17.52) |
|  | Infrastructure | 4.60 (7.98) | 5.33 (9.21) | 5.42 (9.31) | 3.88 (6.71) | 4.19 (5.94) | 5.89 (6.54) | 5.26 (6.49) | 5.60 (6.70) |
|  | Agriculture | 2.82 (4.89) | 2.01 (3.48) | 2.48 (4.26) | 2.72 (4.71) | 2.86 (4.06) | 5.33 (5.91) | 3.73 (4.59) | 4.11 (4.92) |
|  | Refugees in donor country | 0.46 (0.79) | 0.26 (0.45) | 0.12 (0.21) | 0.12 (0.21) | 7.86 (11.16) | 8.02 (8.90) | 1.00 (1.23) | 2.45 (2.93) |
|  | Donor administration costs | 4.97 (8.63) | 5.78 (9.98) | 7.37 (12.66) | 6.84 (11.82) | 5.24 (7.44) | 6.53 (7.25) | 6.03 (7.44) | 6.35 (7.60) |
|  | Energy | 4.12 (7.15) | 3.55 (6.13) | 3.13 (5.37) | 2.94 (5.08) | 2.38 (3.38) | 4.44 (4.92) | 4.67 (5.76) | 6.62 (7.93) |
|  | Debt relief | 0.14 (0.25) | 0.11 (0.19) | 0.03 (0.06) | 0.00 (0.01) | 0.13 (0.19) | 0.00 (0.00) | 0.00 (0.00) | 0.00 (0.00) |
|  | Multisector | 1.51 (2.63) | 1.72 (2.98) | 1.86 (3.20) | 1.77 (3.06) | 1.70 (2.41) | 2.45 (2.72) | 3.06 (3.77) | 2.81 (3.37) |
|  | Water and sanitation | 2.24 (3.88) | 3.18 (5.49) | 2.05 (3.53) | 2.89 (5.00) | 2.92 (4.14) | 4.03 (4.47) | 3.96 (4.88) | 3.36 (4.02) |
|  | Financial services and business support | 3.16 (5.47) | 3.54 (6.12) | 3.79 (6.51) | 4.24 (7.33) | 5.16 (7.33) | 6.06 (6.73) | 3.99 (4.92) | 4.41 (5.28) |
|  | Environmental protection | 1.45 (2.52) | 2.00 (3.46) | 2.35 (4.05) | 1.33 (2.31) | 2.31 (3.28) | 2.76 (3.06) | 2.19 (2.70) | 1.77 (2.12) |
|  | Other social services | 1.82 (3.15) | 1.69 (2.92) | 1.79 (3.07) | 1.70 (2.94) | 2.01 (2.86) | 3.42 (3.80) | 3.12 (3.85) | 2.43 (2.91) |
|  | Conflict, peace and security | 3.38 (5.86) | 2.42 (4.18) | 2.79 (4.79) | 2.67 (4.61) | 3.30 (4.69) | 3.17 (3.51) | 3.31 (4.08) | 3.18 (3.80) |
|  | General budget support | 1.93 (3.35) | 1.78 (3.07) | 1.70 (2.92) | 2.12 (3.67) | 1.82 (2.59) | 1.63 (1.81) | 1.52 (1.88) | 1.34 (1.61) |
|  | Unspecified | 1.65 (2.86) | 1.12 (1.93) | 1.04 (1.79) | 0.71 (1.23) | 1.21 (1.72) | 0.80 (0.89) | 0.91 (1.12) | 0.72 (0.86) |
|  | Industry, construction and mining | 2.44 (4.24) | 2.49 (4.30) | 0.91 (1.57) | 0.84 (1.46) | 1.52 (2.16) | 1.38 (1.54) | 2.28 (2.81) | 1.78 (2.13) |
|  | Food aid and commodity assistance | 0.55 (0.95) | 0.50 (0.86) | 0.47 (0.81) | 0.26 (0.45) | 0.56 (0.79) | 0.54 (0.60) | 0.47 (0.58) | 0.77 (0.93) |
|  | Trade policy | 1.70 (2.95) | 0.39 (0.67) | 0.47 (0.81) | 0.48 (0.84) | 0.47 (0.66) | 0.57 (0.64) | 0.53 (0.65) | 0.48 (0.58) |
|  | Tourism | 0.06 (0.11) | 0.17 (0.29) | 0.14 (0.24) | 0.03 (0.05) | 0.13 (0.18) | 0.08 (0.09) | 0.09 (0.11) | 0.04 (0.05) |
| Iceland | Health | 3.06 (9.18) | 3.18 (8.96) | 4.61 (10.17) | 3.47 (7.77) | 2.51 (4.92) | 2.90 (4.29) | 1.82 (2.63) | 3.43 (4.63) |
|  | Humanitarian aid | 1.95 (5.87) | 1.29 (3.65) | 2.28 (5.04) | 2.45 (5.49) | 4.98 (9.77) | 7.66 (11.34) | 6.44 (9.32) | 8.83 (11.90) |
|  | Government and civil society | 2.82 (8.48) | 3.07 (8.67) | 4.17 (9.21) | 3.95 (8.85) | 3.75 (7.35) | 2.87 (4.26) | 3.52 (5.09) | 5.13 (6.91) |
|  | Education | 2.30 (6.89) | 2.42 (6.83) | 4.58 (10.10) | 3.08 (6.91) | 2.72 (5.33) | 2.47 (3.65) | 1.73 (2.51) | 1.17 (1.58) |
|  | Infrastructure | 0.31 (0.92) | 0.38 (1.07) | 0.38 (0.83) | 0.53 (1.19) | 0.81 (1.59) | 0.77 (1.14) | 0.69 (1.00) | 1.28 (1.72) |
|  | Agriculture | 6.69 (20.10) | 6.72 (18.96) | 8.89 (19.64) | 6.65 (14.91) | 5.23 (10.25) | 5.17 (7.66) | 5.38 (7.78) | 7.56 (10.18) |
|  | Refugees in donor country | 0.22 (0.67) | 0.27 (0.76) | 0.40 (0.89) | 3.04 (6.82) | 6.04 (11.85) | 18.05 (26.72) | 22.43 (32.46) | 11.20 (15.09) |
|  | Donor administration costs | 2.72 (8.16) | 2.62 (7.40) | 3.14 (6.93) | 3.17 (7.11) | 3.31 (6.49) | 3.70 (5.48) | 3.50 (5.07) | 3.81 (5.13) |
|  | Energy | 3.84 (11.53) | 5.10 (14.39) | 5.26 (11.61) | 5.67 (12.72) | 4.71 (9.24) | 4.99 (7.39) | 4.74 (6.86) | 5.52 (7.44) |
|  | Debt relief | 0.24 (0.71) | 0.01 (0.04) | 0.03 (0.07) | 0.01 (0.02) | 0.26 (0.51) | 0.00 (0.00) | 0.00 (0.01) | 0.42 (0.56) |
|  | Multisector | 0.78 (2.35) | 0.99 (2.79) | 1.49 (3.29) | 1.00 (2.24) | 0.63 (1.24) | 0.70 (1.04) | 1.09 (1.58) | 7.48 (10.08) |
|  | Water and sanitation | 0.73 (2.19) | 1.10 (3.09) | 1.48 (3.27) | 2.29 (5.14) | 3.99 (7.83) | 3.52 (5.21) | 2.14 (3.09) | 5.17 (6.96) |
|  | Financial services and business support | 0.31 (0.93) | 0.13 (0.36) | 0.45 (0.98) | 0.29 (0.65) | 0.60 (1.17) | 0.32 (0.48) | 0.33 (0.48) | 0.43 (0.57) |
|  | Environmental protection | 0.49 (1.47) | 0.53 (1.49) | 0.32 (0.70) | 0.22 (0.49) | 0.40 (0.78) | 0.24 (0.36) | 0.30 (0.44) | 0.10 (0.14) |
|  | Other social services | 2.91 (8.74) | 3.81 (10.74) | 3.91 (8.64) | 4.38 (9.83) | 6.59 (12.93) | 7.50 (11.10) | 8.74 (12.64) | 8.30 (11.18) |
|  | Conflict, peace and security | 1.21 (3.64) | 1.21 (3.41) | 0.99 (2.20) | 1.44 (3.23) | 1.07 (2.09) | 0.95 (1.41) | 1.01 (1.46) | 1.11 (1.49) |
|  | General budget support | 0.00 (0.00) | 0.00 (0.00) | 0.00 (0.00) | 0.01 (0.02) | 0.01 (0.01) | 0.01 (0.02) | 0.00 (0.00) | 0.00 (0.01) |
|  | Unspecified | 0.56 (1.67) | 0.49 (1.37) | 0.56 (1.23) | 0.56 (1.26) | 0.72 (1.41) | 1.07 (1.59) | 1.37 (1.98) | 0.89 (1.20) |
|  | Industry, construction and mining | 0.09 (0.27) | 0.31 (0.89) | 0.06 (0.13) | 0.07 (0.15) | 0.17 (0.34) | 0.11 (0.16) | 0.17 (0.25) | 0.15 (0.21) |
|  | Food aid and commodity assistance | 0.01 (0.02) | 0.00 (0.01) | 0.01 (0.02) | 0.01 (0.02) | 0.01 (0.02) | 0.01 (0.01) | 0.04 (0.06) | 0.04 (0.06) |
|  | Trade policy | 0.02 (0.06) | 0.03 (0.08) | 0.04 (0.08) | 0.03 (0.06) | 0.07 (0.13) | 0.18 (0.27) | 0.04 (0.05) | 0.08 (0.10) |
|  | Tourism | 0.00 (0.00) | 0.00 (0.00) | 0.00 (0.01) | 0.00 (0.01) | 0.02 (0.04) | 0.00 (0.01) | 0.01 (0.01) | 0.02 (0.03) |

**Supplementary table s2: Estimated bi-lateral ODA by sector in million USD at constant price of 2018 and sectoral shares (%) in the 29 DAC member countries, 2011–2018.** ODA: official development assistance; DAC: Development Assistance Committee.

| Country | Sector | 2011 | 2012 | 2013 | 2014 | 2015 | 2016 | 2017 | 2018 |
| --- | --- | --- | --- | --- | --- | --- | --- | --- | --- |
| United States | Health | 8218.56 (25.53) | 7882.08 (27.29) | 8215.98 (27.99) | 8056.43 (26.75) | 7907.30 (27.37) | 8511.18 (27.87) | 9242.74 (29.37) | 8594.96 (28.03) |
|  | Humanitarian aid | 4804.56 (14.93) | 4334.59 (15.01) | 5303.51 (18.07) | 6394.05 (21.23) | 6476.99 (22.42) | 6536.27 (21.41) | 7145.51 (22.70) | 7087.25 (23.11) |
|  | Government and civil society | 4225.08 (13.13) | 4427.83 (15.33) | 3259.78 (11.11) | 3983.85 (13.23) | 2801.62 (9.70) | 3157.71 (10.34) | 2956.89 (9.40) | 3314.04 (10.81) |
|  | Education | 996.38 (3.10) | 1028.43 (3.56) | 916.02 (3.12) | 1117.56 (3.71) | 1020.28 (3.53) | 1563.38 (5.12) | 1594.14 (5.07) | 1608.52 (5.25) |
|  | Infrastructure | 1252.26 (3.89) | 782.06 (2.71) | 765.13 (2.61) | 634.11 (2.11) | 420.21 (1.45) | 262.25 (0.86) | 104.93 (0.33) | 98.31 (0.32) |
|  | Agriculture | 1595.61 (4.96) | 1419.81 (4.92) | 1317.18 (4.49) | 1470.70 (4.88) | 1482.86 (5.13) | 1473.78 (4.83) | 1286.43 (4.09) | 1082.41 (3.53) |
|  | Refugees in donor country | 940.24 (2.92) | 1067.37 (3.70) | 1103.87 (3.76) | 1362.37 (4.52) | 1453.17 (5.03) | 2001.58 (6.55) | 1491.73 (4.74) | 1621.20 (5.29) |
|  | Donor administration costs | 1898.13 (5.90) | 2398.69 (8.31) | 2363.14 (8.05) | 2103.68 (6.99) | 2235.20 (7.74) | 2235.50 (7.32) | 2896.13 (9.20) | 2619.22 (8.54) |
|  | Energy | 569.92 (1.77) | 547.63 (1.90) | 634.66 (2.16) | 425.00 (1.41) | 452.23 (1.57) | 518.40 (1.70) | 517.51 (1.64) | 499.94 (1.63) |
|  | Debt relief | 1845.74 (5.73) | 70.01 (0.24) | 360.19 (1.23) | 50.29 (0.17) | 11.78 (0.04) | 9.75 (0.03) | 18.18 (0.06) | 4.30 (0.01) |
|  | Multisector | 816.34 (2.54) | 444.69 (1.54) | 539.28 (1.84) | 673.85 (2.24) | 765.08 (2.65) | 771.59 (2.53) | 330.59 (1.05) | 463.10 (1.51) |
|  | Water and sanitation | 447.57 (1.39) | 425.21 (1.47) | 480.70 (1.64) | 302.06 (1.00) | 408.65 (1.41) | 391.11 (1.28) | 525.38 (1.67) | 402.00 (1.31) |
|  | Financial services and business support | 682.95 (2.12) | 708.88 (2.45) | 506.16 (1.72) | 503.71 (1.67) | 359.64 (1.24) | 362.74 (1.19) | 340.97 (1.08) | 341.46 (1.11) |
|  | Environmental protection | 496.13 (1.54) | 532.34 (1.84) | 584.90 (1.99) | 669.19 (2.22) | 685.60 (2.37) | 755.36 (2.47) | 696.43 (2.21) | 548.21 (1.79) |
|  | Other social services | 859.18 (2.67) | 478.42 (1.66) | 796.34 (2.71) | 509.78 (1.69) | 359.98 (1.25) | 417.60 (1.37) | 374.30 (1.19) | 269.65 (0.88) |
|  | Conflict, peace and security | 803.54 (2.50) | 703.66 (2.44) | 623.85 (2.13) | 604.07 (2.01) | 685.00 (2.37) | 743.74 (2.44) | 757.52 (2.41) | 801.56 (2.61) |
|  | General budget support | 242.00 (0.75) | 417.69 (1.45) | 632.55 (2.16) | 188.61 (0.63) | 304.10 (1.05) | 7.49 (0.02) | 98.82 (0.31) | 136.78 (0.45) |
|  | Unspecified | 349.20 (1.08) | 52.22 (0.18) | 55.26 (0.19) | 117.03 (0.39) | 0.00 (0.00) | 0.00 (0.00) | 95.37 (0.30) | 0.00 (0.00) |
|  | Industry, construction and mining | 155.03 (0.48) | 138.55 (0.48) | 107.44 (0.37) | 86.98 (0.29) | 83.00 (0.29) | 116.99 (0.38) | 85.90 (0.27) | 56.71 (0.18) |
|  | Food aid and commodity assistance | 753.07 (2.34) | 712.97 (2.47) | 492.55 (1.68) | 573.34 (1.90) | 775.65 (2.68) | 577.70 (1.89) | 686.92 (2.18) | 962.70 (3.14) |
|  | Trade policy | 228.87 (0.71) | 296.44 (1.03) | 277.98 (0.95) | 249.42 (0.83) | 205.05 (0.71) | 120.45 (0.39) | 224.81 (0.71) | 155.29 (0.51) |
|  | Tourism | 6.38 (0.02) | 9.24 (0.03) | 15.98 (0.05) | 36.88 (0.12) | 1.23 (0.00) | 0.60 (0.00) | 0.48 (0.00) | 0.02 (0.00) |
| Germany | Health | 383.55 (3.96) | 428.27 (4.16) | 479.16 (4.38) | 486.75 (3.61) | 570.92 (3.25) | 668.04 (2.81) | 756.12 (3.14) | 827.25 (3.69) |
|  | Humanitarian aid | 401.78 (4.15) | 397.38 (3.86) | 556.03 (5.08) | 821.26 (6.08) | 889.73 (5.06) | 2217.53 (9.33) | 2798.13 (11.62) | 2646.29 (11.80) |
|  | Government and civil society | 897.10 (9.27) | 1123.71 (10.92) | 1118.28 (10.22) | 1220.70 (9.04) | 1177.80 (6.70) | 1650.25 (6.94) | 1358.73 (5.64) | 1684.82 (7.51) |
|  | Education | 1705.03 (17.62) | 1730.84 (16.83) | 1656.03 (15.13) | 1697.86 (12.58) | 2049.71 (11.66) | 2174.32 (9.14) | 2196.21 (9.12) | 2498.33 (11.14) |
|  | Infrastructure | 256.74 (2.65) | 218.89 (2.13) | 379.54 (3.47) | 203.15 (1.51) | 560.79 (3.19) | 474.93 (2.00) | 323.32 (1.34) | 333.86 (1.49) |
|  | Agriculture | 589.55 (6.09) | 517.66 (5.03) | 631.70 (5.77) | 542.20 (4.02) | 813.18 (4.62) | 869.81 (3.66) | 962.07 (4.00) | 893.23 (3.98) |
|  | Refugees in donor country | 81.33 (0.84) | 76.48 (0.74) | 132.71 (1.21) | 160.95 (1.19) | 3333.37 (18.96) | 7209.45 (30.32) | 6438.79 (26.75) | 3871.52 (17.26) |
|  | Donor administration costs | 409.09 (4.23) | 495.47 (4.82) | 527.12 (4.82) | 564.30 (4.18) | 583.09 (3.32) | 546.48 (2.30) | 810.53 (3.37) | 844.69 (3.77) |
|  | Energy | 1176.87 (12.16) | 795.23 (7.73) | 1173.25 (10.72) | 1522.70 (11.28) | 1986.79 (11.30) | 2056.66 (8.65) | 1788.36 (7.43) | 1853.63 (8.27) |
|  | Debt relief | 428.63 (4.43) | 855.45 (8.32) | 585.36 (5.35) | 1042.17 (7.72) | 87.98 (0.50) | 46.56 (0.20) | 110.88 (0.46) | 25.41 (0.11) |
|  | Multisector | 434.03 (4.49) | 556.95 (5.41) | 516.66 (4.72) | 742.53 (5.50) | 911.06 (5.18) | 940.74 (3.96) | 648.86 (2.70) | 1294.42 (5.77) |
|  | Water and sanitation | 650.42 (6.72) | 583.08 (5.67) | 622.62 (5.69) | 856.05 (6.34) | 905.82 (5.15) | 900.19 (3.79) | 955.80 (3.97) | 988.56 (4.41) |
|  | Financial services and business support | 942.67 (9.74) | 912.17 (8.87) | 1045.20 (9.55) | 1911.60 (14.16) | 2068.49 (11.76) | 1468.50 (6.18) | 1540.94 (6.40) | 1372.61 (6.12) |
|  | Environmental protection | 399.53 (4.13) | 619.96 (6.03) | 605.09 (5.53) | 678.29 (5.03) | 654.88 (3.72) | 1058.73 (4.45) | 1191.15 (4.95) | 946.70 (4.22) |
|  | Other social services | 161.64 (1.67) | 188.39 (1.83) | 167.09 (1.53) | 183.63 (1.36) | 164.26 (0.93) | 258.01 (1.09) | 638.65 (2.65) | 311.35 (1.39) |
|  | Conflict, peace and security | 363.78 (3.76) | 342.32 (3.33) | 369.36 (3.38) | 397.19 (2.94) | 367.87 (2.09) | 520.93 (2.19) | 772.48 (3.21) | 919.42 (4.10) |
|  | General budget support | 142.55 (1.47) | 58.43 (0.57) | 35.11 (0.32) | 36.36 (0.27) | 13.05 (0.07) | 3.14 (0.01) | 2.28 (0.01) | 266.70 (1.19) |
|  | Unspecified | 61.85 (0.64) | 85.12 (0.83) | 88.21 (0.81) | 98.91 (0.73) | 108.08 (0.61) | 133.91 (0.56) | 155.52 (0.65) | 170.13 (0.76) |
|  | Industry, construction and mining | 92.35 (0.95) | 140.90 (1.37) | 164.54 (1.50) | 192.75 (1.43) | 153.51 (0.87) | 118.15 (0.50) | 68.08 (0.28) | 74.57 (0.33) |
|  | Food aid and commodity assistance | 65.55 (0.68) | 123.74 (1.20) | 54.56 (0.50) | 97.04 (0.72) | 143.81 (0.82) | 405.54 (1.71) | 245.91 (1.02) | 280.18 (1.25) |
|  | Trade policy | 22.80 (0.24) | 33.19 (0.32) | 30.92 (0.28) | 38.16 (0.28) | 40.24 (0.23) | 56.27 (0.24) | 307.91 (1.28) | 319.18 (1.42) |
|  | Tourism | 7.42 (0.08) | 2.61 (0.03) | 3.46 (0.03) | 2.55 (0.02) | 0.76 (0.00) | 0.35 (0.00) | 2.56 (0.01) | 1.95 (0.01) |
| Japan | Health | 323.41 (2.74) | 380.39 (3.52) | 388.47 (2.18) | 374.59 (3.05) | 425.43 (3.24) | 458.12 (3.47) | 530.96 (3.47) | 378.90 (2.85) |
|  | Humanitarian aid | 729.42 (6.19) | 493.33 (4.56) | 756.95 (4.24) | 779.17 (6.33) | 1190.67 (9.07) | 757.84 (5.74) | 764.03 (4.99) | 589.32 (4.44) |
|  | Government and civil society | 389.42 (3.30) | 222.82 (2.06) | 378.30 (2.12) | 296.17 (2.41) | 347.46 (2.65) | 330.11 (2.50) | 338.24 (2.21) | 293.18 (2.21) |
|  | Education | 886.30 (7.52) | 655.36 (6.06) | 643.77 (3.61) | 503.58 (4.09) | 469.38 (3.57) | 549.42 (4.16) | 567.74 (3.71) | 577.18 (4.34) |
|  | Infrastructure | 2354.80 (19.98) | 2781.25 (25.71) | 3843.98 (21.54) | 3709.61 (30.16) | 3466.26 (26.39) | 3385.29 (25.65) | 4834.27 (31.59) | 4584.11 (34.51) |
|  | Agriculture | 739.53 (6.27) | 625.81 (5.78) | 762.21 (4.27) | 641.57 (5.22) | 649.99 (4.95) | 823.52 (6.24) | 825.60 (5.39) | 628.53 (4.73) |
|  | Refugees in donor country | 0.53 (0.00) | 0.56 (0.01) | 0.58 (0.00) | 0.56 (0.00) | 0.24 (0.00) | 0.24 (0.00) | 0.30 (0.00) | 0.32 (0.00) |
|  | Donor administration costs | 580.59 (4.93) | 594.08 (5.49) | 599.15 (3.36) | 616.50 (5.01) | 660.63 (5.03) | 690.94 (5.23) | 668.77 (4.37) | 731.35 (5.51) |
|  | Energy | 1129.35 (9.58) | 1588.93 (14.69) | 1068.85 (5.99) | 1222.78 (9.94) | 1684.51 (12.83) | 1717.11 (13.01) | 2102.42 (13.74) | 1856.05 (13.97) |
|  | Debt relief | 1072.29 (9.10) | 3.51 (0.03) | 3690.88 (20.68) | 0.00 (0.00) | 42.60 (0.32) | 15.04 (0.11) | 19.39 (0.13) | 23.72 (0.18) |
|  | Multisector | 648.33 (5.50) | 708.59 (6.55) | 590.31 (3.31) | 759.96 (6.18) | 1046.25 (7.97) | 1167.46 (8.85) | 1349.80 (8.82) | 1224.58 (9.22) |
|  | Water and sanitation | 1333.25 (11.31) | 1155.25 (10.68) | 1171.35 (6.56) | 1192.41 (9.69) | 1276.14 (9.72) | 1259.63 (9.54) | 1154.34 (7.54) | 1050.87 (7.91) |
|  | Financial services and business support | 39.93 (0.34) | 41.64 (0.38) | 48.85 (0.27) | 39.17 (0.32) | 76.31 (0.58) | 41.11 (0.31) | 38.38 (0.25) | 35.90 (0.27) |
|  | Environmental protection | 378.99 (3.22) | 222.09 (2.05) | 341.80 (1.91) | 296.53 (2.41) | 311.75 (2.37) | 244.44 (1.85) | 219.55 (1.43) | 88.49 (0.67) |
|  | Other social services | 162.03 (1.37) | 168.40 (1.56) | 146.61 (0.82) | 378.71 (3.08) | 113.39 (0.86) | 129.00 (0.98) | 188.68 (1.23) | 211.46 (1.59) |
|  | Conflict, peace and security | 77.39 (0.66) | 72.25 (0.67) | 81.14 (0.45) | 81.05 (0.66) | 77.24 (0.59) | 76.81 (0.58) | 68.07 (0.44) | 101.94 (0.77) |
|  | General budget support | 49.68 (0.42) | 123.51 (1.14) | 2358.24 (13.21) | 360.54 (2.93) | 216.99 (1.65) | 739.87 (5.61) | 669.88 (4.38) | 333.09 (2.51) |
|  | Unspecified | 28.45 (0.24) | 255.02 (2.36) | 42.91 (0.24) | 28.61 (0.23) | 38.47 (0.29) | 174.20 (1.32) | 95.08 (0.62) | 98.87 (0.74) |
|  | Industry, construction and mining | 447.88 (3.80) | 308.17 (2.85) | 527.92 (2.96) | 560.81 (4.56) | 587.48 (4.47) | 365.59 (2.77) | 557.52 (3.64) | 262.25 (1.97) |
|  | Food aid and commodity assistance | 328.62 (2.79) | 256.92 (2.37) | 263.61 (1.48) | 281.00 (2.28) | 351.07 (2.67) | 124.55 (0.94) | 187.69 (1.23) | 152.79 (1.15) |
|  | Trade policy | 66.09 (0.56) | 89.21 (0.82) | 94.27 (0.53) | 106.58 (0.87) | 54.21 (0.41) | 70.17 (0.53) | 42.10 (0.28) | 45.22 (0.34) |
|  | Tourism | 19.19 (0.16) | 72.06 (0.67) | 48.45 (0.27) | 69.94 (0.57) | 45.81 (0.35) | 78.16 (0.59) | 81.19 (0.53) | 16.50 (0.12) |
| United Kingdom | Health | 1423.16 (17.33) | 1593.75 (19.34) | 1865.38 (18.41) | 1782.87 (17.43) | 1443.01 (13.15) | 1461.55 (12.21) | 1811.21 (14.97) | 1808.70 (14.44) |
|  | Humanitarian aid | 635.57 (7.74) | 629.23 (7.64) | 1196.27 (11.81) | 1592.01 (15.57) | 1798.70 (16.40) | 1799.53 (15.03) | 1913.52 (15.82) | 1738.75 (13.88) |
|  | Government and civil society | 817.64 (9.96) | 883.34 (10.72) | 954.85 (9.43) | 962.94 (9.41) | 1040.13 (9.48) | 972.86 (8.12) | 977.60 (8.08) | 1003.64 (8.01) |
|  | Education | 977.55 (11.90) | 933.17 (11.32) | 1329.76 (13.13) | 1172.23 (11.46) | 927.40 (8.45) | 1340.12 (11.19) | 967.38 (8.00) | 933.03 (7.45) |
|  | Infrastructure | 221.58 (2.70) | 283.45 (3.44) | 328.85 (3.25) | 243.96 (2.39) | 219.28 (2.00) | 234.94 (1.96) | 299.71 (2.48) | 195.53 (1.56) |
|  | Agriculture | 376.12 (4.58) | 407.45 (4.94) | 445.61 (4.40) | 546.49 (5.34) | 679.11 (6.19) | 547.04 (4.57) | 591.28 (4.89) | 503.72 (4.02) |
|  | Refugees in donor country | 29.30 (0.36) | 41.88 (0.51) | 46.83 (0.46) | 191.78 (1.88) | 356.32 (3.25) | 567.94 (4.74) | 513.87 (4.25) | 493.10 (3.94) |
|  | Donor administration costs | 429.43 (5.23) | 491.95 (5.97) | 341.94 (3.38) | 369.97 (3.62) | 565.95 (5.16) | 650.56 (5.43) | 740.95 (6.12) | 877.28 (7.00) |
|  | Energy | 241.79 (2.94) | 303.24 (3.68) | 238.64 (2.36) | 155.07 (1.52) | 161.30 (1.47) | 277.66 (2.32) | 159.88 (1.32) | 306.46 (2.45) |
|  | Debt relief | 170.00 (2.07) | 104.75 (1.27) | 77.23 (0.76) | 4.60 (0.04) | 0.00 (0.00) | 3.11 (0.03) | 3.93 (0.03) | 4.71 (0.04) |
|  | Multisector | 561.41 (6.84) | 536.96 (6.52) | 695.01 (6.86) | 728.02 (7.12) | 895.83 (8.17) | 1123.80 (9.38) | 1068.67 (8.83) | 1247.70 (9.96) |
|  | Water and sanitation | 164.60 (2.00) | 159.04 (1.93) | 201.60 (1.99) | 257.30 (2.52) | 261.15 (2.38) | 236.07 (1.97) | 240.62 (1.99) | 277.50 (2.22) |
|  | Financial services and business support | 398.40 (4.85) | 275.38 (3.34) | 304.78 (3.01) | 491.83 (4.81) | 889.17 (8.11) | 668.05 (5.58) | 591.98 (4.89) | 1022.98 (8.17) |
|  | Environmental protection | 185.44 (2.26) | 378.44 (4.59) | 555.54 (5.48) | 509.80 (4.98) | 464.59 (4.23) | 441.49 (3.69) | 440.82 (3.64) | 496.25 (3.96) |
|  | Other social services | 362.03 (4.41) | 310.34 (3.77) | 461.16 (4.55) | 287.18 (2.81) | 416.71 (3.80) | 598.19 (5.00) | 320.83 (2.65) | 287.37 (2.29) |
|  | Conflict, peace and security | 271.30 (3.30) | 286.85 (3.48) | 256.78 (2.53) | 268.38 (2.62) | 421.04 (3.84) | 596.15 (4.98) | 617.98 (5.11) | 610.93 (4.88) |
|  | General budget support | 418.65 (5.10) | 324.81 (3.94) | 195.70 (1.93) | 74.89 (0.73) | 70.31 (0.64) | 83.09 (0.69) | 0.00 (0.00) | 0.00 (0.00) |
|  | Unspecified | 153.72 (1.87) | 32.39 (0.39) | 286.69 (2.83) | 339.66 (3.32) | 125.79 (1.15) | 91.97 (0.77) | 343.83 (2.84) | 113.99 (0.91) |
|  | Industry, construction and mining | 160.99 (1.96) | 75.96 (0.92) | 99.86 (0.99) | 130.35 (1.27) | 123.20 (1.12) | 165.59 (1.38) | 327.95 (2.71) | 458.62 (3.66) |
|  | Food aid and commodity assistance | 142.43 (1.73) | 96.77 (1.17) | 136.28 (1.35) | 33.83 (0.33) | 33.60 (0.31) | 49.01 (0.41) | 55.17 (0.46) | 47.42 (0.38) |
|  | Trade policy | 69.09 (0.84) | 89.01 (1.08) | 109.90 (1.08) | 84.72 (0.83) | 76.03 (0.69) | 63.59 (0.53) | 108.15 (0.89) | 95.09 (0.76) |
|  | Tourism | 2.72 (0.03) | 2.58 (0.03) | 1.45 (0.01) | 0.02 (0.00) | 1.65 (0.02) | 2.18 (0.02) | 2.70 (0.02) | 1.78 (0.01) |
| France | Health | 191.12 (2.21) | 184.81 (2.05) | 278.08 (3.64) | 517.50 (6.80) | 181.77 (2.47) | 309.88 (3.86) | 181.69 (1.99) | 243.16 (2.55) |
|  | Humanitarian aid | 76.67 (0.88) | 59.02 (0.65) | 37.96 (0.50) | 45.96 (0.60) | 39.35 (0.53) | 165.45 (2.06) | 85.21 (0.93) | 108.50 (1.14) |
|  | Government and civil society | 154.24 (1.78) | 115.82 (1.29) | 130.72 (1.71) | 109.25 (1.44) | 121.38 (1.65) | 412.16 (5.14) | 218.21 (2.39) | 612.86 (6.42) |
|  | Education | 1366.57 (15.77) | 1391.72 (15.44) | 1342.72 (17.59) | 1310.92 (17.24) | 1200.01 (16.31) | 1224.46 (15.26) | 1318.42 (14.45) | 1272.13 (13.33) |
|  | Infrastructure | 674.24 (7.78) | 854.59 (9.48) | 722.60 (9.47) | 937.47 (12.33) | 611.05 (8.30) | 632.50 (7.88) | 843.24 (9.24) | 732.89 (7.68) |
|  | Agriculture | 371.51 (4.29) | 381.76 (4.24) | 347.95 (4.56) | 471.03 (6.19) | 385.23 (5.23) | 412.14 (5.14) | 622.93 (6.83) | 629.74 (6.60) |
|  | Refugees in donor country | 488.70 (5.64) | 485.66 (5.39) | 416.76 (5.46) | 444.21 (5.84) | 393.46 (5.35) | 504.20 (6.28) | 657.05 (7.20) | 801.66 (8.40) |
|  | Donor administration costs | 419.46 (4.84) | 472.45 (5.24) | 474.12 (6.21) | 501.97 (6.60) | 508.42 (6.91) | 547.68 (6.82) | 585.75 (6.42) | 629.06 (6.59) |
|  | Energy | 151.93 (1.75) | 409.98 (4.55) | 567.43 (7.43) | 550.98 (7.24) | 562.10 (7.64) | 687.05 (8.56) | 846.75 (9.28) | 675.59 (7.08) |
|  | Debt relief | 1225.24 (14.14) | 1504.45 (16.69) | 1073.24 (14.06) | 99.17 (1.30) | 214.31 (2.91) | 150.30 (1.87) | 157.68 (1.73) | 173.73 (1.82) |
|  | Multisector | 466.03 (5.38) | 298.64 (3.31) | 847.36 (11.10) | 598.22 (7.87) | 902.10 (12.26) | 457.64 (5.70) | 354.18 (3.88) | 389.02 (4.08) |
|  | Water and sanitation | 363.88 (4.20) | 467.37 (5.19) | 325.96 (4.27) | 433.90 (5.70) | 441.09 (5.99) | 656.16 (8.18) | 719.64 (7.89) | 672.96 (7.05) |
|  | Financial services and business support | 166.72 (1.92) | 55.37 (0.61) | 44.28 (0.58) | 38.29 (0.50) | 62.26 (0.85) | 225.28 (2.81) | 161.19 (1.77) | 437.14 (4.58) |
|  | Environmental protection | 1019.01 (11.76) | 549.52 (6.10) | 390.05 (5.11) | 499.03 (6.56) | 610.29 (8.29) | 520.04 (6.48) | 331.72 (3.64) | 465.24 (4.88) |
|  | Other social services | 396.40 (4.57) | 722.67 (8.02) | 165.53 (2.17) | 179.16 (2.36) | 202.38 (2.75) | 161.27 (2.01) | 342.44 (3.75) | 286.58 (3.00) |
|  | Conflict, peace and security | 56.96 (0.66) | 37.05 (0.41) | 57.05 (0.75) | 55.02 (0.72) | 47.87 (0.65) | 34.63 (0.43) | 5.05 (0.06) | 15.44 (0.16) |
|  | General budget support | 600.02 (6.92) | 452.67 (5.02) | 241.94 (3.17) | 198.46 (2.61) | 249.84 (3.39) | 188.21 (2.35) | 861.44 (9.44) | 413.81 (4.34) |
|  | Unspecified | 254.89 (2.94) | 271.73 (3.02) | 70.64 (0.93) | 531.84 (6.99) | 527.67 (7.17) | 569.52 (7.10) | 586.65 (6.43) | 579.17 (6.07) |
|  | Industry, construction and mining | 144.36 (1.67) | 239.65 (2.66) | 36.22 (0.47) | 33.10 (0.44) | 46.50 (0.63) | 112.82 (1.41) | 179.50 (1.97) | 145.57 (1.53) |
|  | Food aid and commodity assistance | 46.21 (0.53) | 48.88 (0.54) | 44.51 (0.58) | 43.44 (0.57) | 46.39 (0.63) | 45.15 (0.56) | 48.39 (0.53) | 44.84 (0.47) |
|  | Trade policy | 3.52 (0.04) | 2.84 (0.03) | 1.90 (0.02) | 3.51 (0.05) | 4.54 (0.06) | 4.54 (0.06) | 3.89 (0.04) | 208.63 (2.19) |
|  | Tourism | 28.08 (0.32) | 5.84 (0.06) | 15.22 (0.20) | 3.52 (0.05) | 1.70 (0.02) | 3.82 (0.05) | 12.55 (0.14) | 5.02 (0.05) |
| Netherlands | Health | 283.37 (6.80) | 263.71 (6.81) | 248.97 (6.97) | 263.96 (6.77) | 264.06 (5.61) | 316.65 (8.59) | 286.20 (7.35) | 379.74 (9.91) |
|  | Humanitarian aid | 162.33 (3.89) | 147.86 (3.82) | 141.02 (3.95) | 279.96 (7.18) | 523.66 (11.12) | 359.87 (9.77) | 306.77 (7.88) | 288.94 (7.54) |
|  | Government and civil society | 335.46 (8.05) | 923.21 (23.84) | 876.48 (24.54) | 827.81 (21.24) | 785.83 (16.69) | 544.41 (14.77) | 536.36 (13.77) | 621.74 (16.22) |
|  | Education | 376.08 (9.02) | 259.56 (6.70) | 208.15 (5.83) | 158.80 (4.08) | 108.04 (2.29) | 121.16 (3.29) | 96.11 (2.47) | 181.80 (4.74) |
|  | Infrastructure | 23.67 (0.57) | 38.25 (0.99) | 25.09 (0.70) | 22.00 (0.56) | 12.79 (0.27) | 5.01 (0.14) | 11.88 (0.31) | 7.38 (0.19) |
|  | Agriculture | 149.13 (3.58) | 243.14 (6.28) | 289.32 (8.10) | 236.28 (6.06) | 215.80 (4.58) | 305.35 (8.29) | 265.46 (6.82) | 296.40 (7.73) |
|  | Refugees in donor country | 441.14 (10.58) | 331.33 (8.55) | 348.09 (9.75) | 871.99 (22.38) | 1466.71 (31.15) | 479.17 (13.00) | 894.03 (22.96) | 629.32 (16.42) |
|  | Donor administration costs | 266.75 (6.40) | 387.90 (10.02) | 240.21 (6.72) | 216.98 (5.57) | 373.31 (7.93) | 291.37 (7.91) | 383.00 (9.84) | 359.89 (9.39) |
|  | Energy | 135.04 (3.24) | 92.94 (2.40) | 91.65 (2.57) | 54.19 (1.39) | 40.37 (0.86) | 57.74 (1.57) | 58.32 (1.50) | 88.43 (2.31) |
|  | Debt relief | 110.83 (2.66) | 117.56 (3.04) | 57.54 (1.61) | 58.77 (1.51) | 50.39 (1.07) | 60.12 (1.63) | 124.22 (3.19) | 0.00 (0.00) |
|  | Multisector | 922.50 (22.12) | 172.92 (4.46) | 174.27 (4.88) | 75.57 (1.94) | 85.87 (1.82) | 275.68 (7.48) | 147.33 (3.78) | 106.38 (2.78) |
|  | Water and sanitation | 223.36 (5.36) | 165.07 (4.26) | 171.51 (4.80) | 177.92 (4.57) | 167.90 (3.57) | 201.38 (5.46) | 209.01 (5.37) | 218.13 (5.69) |
|  | Financial services and business support | 190.02 (4.56) | 226.95 (5.86) | 201.31 (5.64) | 242.69 (6.23) | 235.26 (5.00) | 210.21 (5.70) | 174.46 (4.48) | 251.92 (6.57) |
|  | Environmental protection | 77.89 (1.87) | 93.63 (2.42) | 75.40 (2.11) | 37.82 (0.97) | 39.47 (0.84) | 32.73 (0.89) | 33.66 (0.86) | 28.60 (0.75) |
|  | Other social services | 75.59 (1.81) | 44.30 (1.14) | 46.38 (1.30) | 50.71 (1.30) | 46.68 (0.99) | 71.03 (1.93) | 50.62 (1.30) | 69.08 (1.80) |
|  | Conflict, peace and security | 147.52 (3.54) | 170.05 (4.39) | 194.52 (5.45) | 159.10 (4.08) | 125.55 (2.67) | 181.44 (4.92) | 160.23 (4.12) | 169.98 (4.43) |
|  | General budget support | 83.44 (2.00) | 36.42 (0.94) | 22.32 (0.62) | 0.00 (0.00) | 0.00 (0.00) | 0.00 (0.00) | 0.00 (0.00) | 0.00 (0.00) |
|  | Unspecified | 12.54 (0.30) | 6.49 (0.17) | 1.48 (0.04) | 2.64 (0.07) | 10.26 (0.22) | 9.06 (0.25) | 6.36 (0.16) | 3.81 (0.10) |
|  | Industry, construction and mining | 4.61 (0.11) | 11.35 (0.29) | 9.03 (0.25) | 19.57 (0.50) | 37.91 (0.81) | 34.98 (0.95) | 38.54 (0.99) | 45.84 (1.20) |
|  | Food aid and commodity assistance | 82.29 (1.97) | 60.43 (1.56) | 40.90 (1.15) | 53.65 (1.38) | 50.20 (1.07) | 32.05 (0.87) | 45.02 (1.16) | 28.84 (0.75) |
|  | Trade policy | 64.43 (1.55) | 79.25 (2.05) | 108.26 (3.03) | 86.40 (2.22) | 68.59 (1.46) | 95.78 (2.60) | 66.27 (1.70) | 57.11 (1.49) |
|  | Tourism | 1.57 (0.04) | 0.60 (0.02) | 0.00 (0.00) | 0.00 (0.00) | 0.00 (0.00) | 0.00 (0.00) | 0.00 (0.00) | 0.00 (0.00) |
| Sweden | Health | 159.16 (5.12) | 234.84 (7.39) | 205.43 (6.24) | 197.54 (5.23) | 184.57 (3.66) | 206.20 (5.62) | 248.95 (6.35) | 245.76 (6.22) |
|  | Humanitarian aid | 410.80 (13.22) | 389.14 (12.24) | 372.94 (11.33) | 501.89 (13.28) | 388.11 (7.69) | 475.84 (12.97) | 475.93 (12.14) | 493.81 (12.49) |
|  | Government and civil society | 582.12 (18.74) | 635.00 (19.97) | 655.94 (19.94) | 706.75 (18.70) | 650.41 (12.89) | 687.13 (18.73) | 744.53 (18.99) | 780.80 (19.75) |
|  | Education | 124.10 (3.99) | 75.24 (2.37) | 98.75 (3.00) | 144.67 (3.83) | 91.76 (1.82) | 123.33 (3.36) | 114.36 (2.92) | 163.79 (4.14) |
|  | Infrastructure | 30.73 (0.99) | 36.00 (1.13) | 28.81 (0.88) | 25.60 (0.68) | 19.90 (0.39) | 16.32 (0.44) | 22.98 (0.59) | 25.17 (0.64) |
|  | Agriculture | 116.32 (3.74) | 152.54 (4.80) | 114.70 (3.49) | 119.18 (3.15) | 97.02 (1.92) | 122.38 (3.34) | 154.78 (3.95) | 183.01 (4.63) |
|  | Refugees in donor country | 410.20 (13.20) | 494.79 (15.56) | 582.62 (17.71) | 937.04 (24.79) | 2463.75 (48.83) | 844.25 (23.01) | 832.49 (21.23) | 524.75 (13.27) |
|  | Donor administration costs | 214.41 (6.90) | 203.95 (6.41) | 207.59 (6.31) | 210.63 (5.57) | 221.62 (4.39) | 231.07 (6.30) | 210.66 (5.37) | 222.19 (5.62) |
|  | Energy | 52.62 (1.69) | 62.02 (1.95) | 79.38 (2.41) | 43.45 (1.15) | 92.34 (1.83) | 44.83 (1.22) | 78.89 (2.01) | 102.70 (2.60) |
|  | Debt relief | 156.27 (5.03) | 0.00 (0.00) | 0.00 (0.00) | 0.00 (0.00) | 0.00 (0.00) | 0.00 (0.00) | 0.00 (0.00) | 0.00 (0.00) |
|  | Multisector | 134.01 (4.31) | 98.08 (3.08) | 128.78 (3.91) | 150.27 (3.98) | 171.18 (3.39) | 216.86 (5.91) | 210.11 (5.36) | 213.58 (5.40) |
|  | Water and sanitation | 57.51 (1.85) | 109.49 (3.44) | 115.09 (3.50) | 95.14 (2.52) | 98.68 (1.96) | 94.66 (2.58) | 103.34 (2.64) | 146.28 (3.70) |
|  | Financial services and business support | 92.56 (2.98) | 109.37 (3.44) | 106.05 (3.22) | 110.54 (2.92) | 42.39 (0.84) | 86.98 (2.37) | 116.36 (2.97) | 136.15 (3.44) |
|  | Environmental protection | 123.66 (3.98) | 90.19 (2.84) | 103.64 (3.15) | 104.02 (2.75) | 102.87 (2.04) | 105.22 (2.87) | 159.61 (4.07) | 178.03 (4.50) |
|  | Other social services | 56.45 (1.82) | 62.22 (1.96) | 44.43 (1.35) | 66.72 (1.76) | 61.76 (1.22) | 58.55 (1.60) | 87.63 (2.23) | 126.44 (3.20) |
|  | Conflict, peace and security | 136.63 (4.40) | 155.32 (4.88) | 159.30 (4.84) | 139.99 (3.70) | 129.37 (2.56) | 139.72 (3.81) | 166.40 (4.24) | 185.50 (4.69) |
|  | General budget support | 120.09 (3.87) | 105.38 (3.31) | 93.55 (2.84) | 39.28 (1.04) | 71.96 (1.43) | 0.00 (0.00) | 0.00 (0.00) | 0.00 (0.00) |
|  | Unspecified | 51.39 (1.65) | 81.25 (2.56) | 89.54 (2.72) | 100.60 (2.66) | 56.41 (1.12) | 139.43 (3.80) | 96.99 (2.47) | 116.74 (2.95) |
|  | Industry, construction and mining | 36.88 (1.19) | 39.86 (1.25) | 59.06 (1.80) | 47.23 (1.25) | 53.24 (1.06) | 43.86 (1.20) | 54.63 (1.39) | 69.76 (1.76) |
|  | Food aid and commodity assistance | 0.10 (0.00) | 0.00 (0.00) | 0.00 (0.00) | 0.00 (0.00) | 0.09 (0.00) | 0.00 (0.00) | 0.30 (0.01) | 0.29 (0.01) |
|  | Trade policy | 40.82 (1.31) | 45.21 (1.42) | 44.73 (1.36) | 39.73 (1.05) | 47.82 (0.95) | 32.01 (0.87) | 41.76 (1.06) | 38.56 (0.98) |
|  | Tourism | 0.00 (0.00) | 0.00 (0.00) | 0.00 (0.00) | 0.00 (0.00) | 0.43 (0.01) | 0.84 (0.02) | 0.82 (0.02) | 0.06 (0.00) |
| Canada | Health | 557.90 (16.22) | 551.84 (16.24) | 648.81 (21.63) | 548.09 (18.58) | 441.45 (14.13) | 474.81 (16.47) | 533.30 (16.50) | 566.34 (15.99) |
|  | Humanitarian aid | 353.40 (10.27) | 413.56 (12.17) | 492.04 (16.40) | 583.06 (19.76) | 698.69 (22.36) | 525.19 (18.21) | 655.75 (20.28) | 660.76 (18.65) |
|  | Government and civil society | 325.76 (9.47) | 263.93 (7.77) | 194.39 (6.48) | 215.09 (7.29) | 205.56 (6.58) | 210.42 (7.30) | 205.80 (6.37) | 241.20 (6.81) |
|  | Education | 272.98 (7.94) | 260.56 (7.67) | 226.57 (7.55) | 214.46 (7.27) | 256.21 (8.20) | 221.08 (7.67) | 209.89 (6.49) | 229.14 (6.47) |
|  | Infrastructure | 22.83 (0.66) | 20.63 (0.61) | 16.86 (0.56) | 20.91 (0.71) | 28.08 (0.90) | 14.30 (0.50) | 8.13 (0.25) | 7.57 (0.21) |
|  | Agriculture | 291.43 (8.47) | 224.56 (6.61) | 238.63 (7.96) | 190.03 (6.44) | 184.04 (5.89) | 198.17 (6.87) | 160.65 (4.97) | 204.41 (5.77) |
|  | Refugees in donor country | 282.24 (8.21) | 221.95 (6.53) | 178.19 (5.94) | 192.12 (6.51) | 220.78 (7.07) | 416.36 (14.44) | 475.66 (14.71) | 505.93 (14.28) |
|  | Donor administration costs | 239.90 (6.97) | 235.21 (6.92) | 219.18 (7.31) | 205.94 (6.98) | 206.51 (6.61) | 197.12 (6.84) | 182.26 (5.64) | 248.24 (7.01) |
|  | Energy | 241.02 (7.01) | 138.87 (4.09) | 78.01 (2.60) | 5.21 (0.18) | 14.26 (0.46) | 13.39 (0.46) | 129.93 (4.02) | 197.34 (5.57) |
|  | Debt relief | 3.12 (0.09) | 164.54 (4.84) | 0.00 (0.00) | 0.00 (0.00) | 0.00 (0.00) | 0.00 (0.00) | 6.64 (0.21) | 0.00 (0.00) |
|  | Multisector | 276.70 (8.04) | 242.11 (7.13) | 196.82 (6.56) | 396.86 (13.45) | 451.09 (14.44) | 200.74 (6.96) | 170.44 (5.27) | 221.82 (6.26) |
|  | Water and sanitation | 31.72 (0.92) | 72.42 (2.13) | 47.34 (1.58) | 43.48 (1.47) | 65.54 (2.10) | 33.88 (1.17) | 40.67 (1.26) | 40.87 (1.15) |
|  | Financial services and business support | 68.96 (2.00) | 70.11 (2.06) | 84.07 (2.80) | 68.66 (2.33) | 63.86 (2.04) | 73.76 (2.56) | 86.74 (2.68) | 101.88 (2.88) |
|  | Environmental protection | 40.90 (1.19) | 148.88 (4.38) | 38.20 (1.27) | 21.98 (0.74) | 14.24 (0.46) | 28.49 (0.99) | 39.87 (1.23) | 26.59 (0.75) |
|  | Other social services | 32.85 (0.95) | 42.96 (1.26) | 40.90 (1.36) | 27.78 (0.94) | 28.48 (0.91) | 32.63 (1.13) | 32.46 (1.00) | 63.10 (1.78) |
|  | Conflict, peace and security | 131.47 (3.82) | 88.05 (2.59) | 53.90 (1.80) | 37.02 (1.25) | 101.03 (3.23) | 51.52 (1.79) | 90.96 (2.81) | 94.09 (2.66) |
|  | General budget support | 56.04 (1.63) | 37.23 (1.10) | 30.12 (1.00) | 16.12 (0.55) | 37.75 (1.21) | 8.29 (0.29) | 4.06 (0.13) | 10.81 (0.31) |
|  | Unspecified | 98.36 (2.86) | 79.83 (2.35) | 93.26 (3.11) | 79.71 (2.70) | 18.07 (0.58) | 22.48 (0.78) | 79.08 (2.45) | 31.27 (0.88) |
|  | Industry, construction and mining | 56.43 (1.64) | 44.92 (1.32) | 73.24 (2.44) | 42.46 (1.44) | 52.83 (1.69) | 99.00 (3.43) | 78.70 (2.43) | 65.53 (1.85) |
|  | Food aid and commodity assistance | 24.64 (0.72) | 36.58 (1.08) | 38.94 (1.30) | 17.55 (0.59) | 15.24 (0.49) | 44.12 (1.53) | 25.56 (0.79) | 13.98 (0.39) |
|  | Trade policy | 30.63 (0.89) | 38.32 (1.13) | 6.51 (0.22) | 19.35 (0.66) | 18.91 (0.61) | 16.35 (0.57) | 15.28 (0.47) | 10.03 (0.28) |
|  | Tourism | 0.53 (0.02) | 0.49 (0.01) | 3.63 (0.12) | 4.44 (0.15) | 2.19 (0.07) | 1.49 (0.05) | 0.90 (0.03) | 1.42 (0.04) |
| Norway | Health | 167.25 (6.09) | 165.85 (5.92) | 240.14 (7.15) | 252.64 (7.92) | 251.01 (7.01) | 250.68 (6.36) | 277.05 (8.18) | 270.43 (8.26) |
|  | Humanitarian aid | 289.71 (10.55) | 259.49 (9.27) | 327.56 (9.75) | 367.46 (11.52) | 431.23 (12.04) | 507.95 (12.88) | 578.27 (17.07) | 513.45 (15.68) |
|  | Government and civil society | 364.91 (13.29) | 343.31 (12.26) | 403.34 (12.00) | 374.54 (11.74) | 382.86 (10.69) | 341.46 (8.66) | 337.14 (9.95) | 377.24 (11.52) |
|  | Education | 208.30 (7.59) | 215.00 (7.68) | 224.15 (6.67) | 237.64 (7.45) | 334.25 (9.34) | 430.15 (10.90) | 411.28 (12.14) | 373.52 (11.41) |
|  | Infrastructure | 4.88 (0.18) | 3.83 (0.14) | 1.07 (0.03) | 2.29 (0.07) | 3.99 (0.11) | 4.04 (0.10) | 3.31 (0.10) | 1.69 (0.05) |
|  | Agriculture | 123.07 (4.48) | 136.74 (4.88) | 168.79 (5.02) | 139.19 (4.36) | 136.35 (3.81) | 97.27 (2.47) | 123.41 (3.64) | 109.43 (3.34) |
|  | Refugees in donor country | 202.85 (7.39) | 175.50 (6.27) | 205.88 (6.13) | 227.26 (7.12) | 497.15 (13.89) | 908.31 (23.03) | 161.20 (4.76) | 101.71 (3.11) |
|  | Donor administration costs | 206.70 (7.53) | 208.40 (7.44) | 230.97 (6.87) | 230.02 (7.21) | 260.79 (7.28) | 264.64 (6.71) | 271.37 (8.01) | 261.08 (7.97) |
|  | Energy | 211.36 (7.70) | 280.15 (10.00) | 180.57 (5.37) | 160.65 (5.03) | 125.51 (3.51) | 90.24 (2.29) | 73.06 (2.16) | 82.81 (2.53) |
|  | Debt relief | 16.78 (0.61) | 16.38 (0.58) | 18.08 (0.54) | 14.88 (0.47) | 18.69 (0.52) | 20.30 (0.51) | 26.27 (0.78) | 25.85 (0.79) |
|  | Multisector | 86.73 (3.16) | 108.84 (3.89) | 102.21 (3.04) | 96.75 (3.03) | 84.95 (2.37) | 90.15 (2.29) | 113.03 (3.34) | 151.52 (4.63) |
|  | Water and sanitation | 17.76 (0.65) | 22.24 (0.79) | 25.16 (0.75) | 30.92 (0.97) | 27.59 (0.77) | 29.44 (0.75) | 18.58 (0.55) | 18.01 (0.55) |
|  | Financial services and business support | 131.36 (4.79) | 91.57 (3.27) | 98.95 (2.94) | 179.48 (5.62) | 212.78 (5.94) | 211.70 (5.37) | 225.37 (6.65) | 231.46 (7.07) |
|  | Environmental protection | 231.48 (8.43) | 349.30 (12.47) | 731.23 (21.76) | 494.39 (15.49) | 436.07 (12.18) | 400.80 (10.16) | 416.02 (12.28) | 435.84 (13.31) |
|  | Other social services | 64.08 (2.33) | 54.21 (1.94) | 70.17 (2.09) | 59.66 (1.87) | 51.81 (1.45) | 54.86 (1.39) | 68.51 (2.02) | 45.44 (1.39) |
|  | Conflict, peace and security | 177.12 (6.45) | 173.83 (6.21) | 172.24 (5.13) | 172.07 (5.39) | 169.24 (4.73) | 150.73 (3.82) | 167.14 (4.93) | 192.45 (5.88) |
|  | General budget support | 119.97 (4.37) | 86.84 (3.10) | 63.58 (1.89) | 54.21 (1.70) | 55.32 (1.55) | 31.77 (0.81) | 30.56 (0.90) | 18.44 (0.56) |
|  | Unspecified | 50.52 (1.84) | 40.31 (1.44) | 35.35 (1.05) | 42.59 (1.33) | 45.93 (1.28) | 22.38 (0.57) | 49.50 (1.46) | 25.83 (0.79) |
|  | Industry, construction and mining | 45.95 (1.67) | 48.69 (1.74) | 44.71 (1.33) | 41.63 (1.30) | 41.78 (1.17) | 26.51 (0.67) | 26.61 (0.79) | 25.78 (0.79) |
|  | Food aid and commodity assistance | 0.41 (0.02) | 3.59 (0.13) | 3.24 (0.10) | 0.04 (0.00) | 0.32 (0.01) | 0.01 (0.00) | 0.00 (0.00) | 0.01 (0.00) |
|  | Trade policy | 14.02 (0.51) | 13.82 (0.49) | 11.11 (0.33) | 12.11 (0.38) | 12.47 (0.35) | 10.91 (0.28) | 10.48 (0.31) | 12.64 (0.39) |
|  | Tourism | 9.84 (0.36) | 2.57 (0.09) | 2.26 (0.07) | 0.39 (0.01) | 0.26 (0.01) | 0.34 (0.01) | 0.29 (0.01) | 0.32 (0.01) |
| Spain | Health | 146.18 (6.46) | 74.74 (7.34) | 70.35 (6.53) | 58.67 (9.04) | 42.15 (5.09) | 55.79 (1.79) | 56.14 (4.88) | 54.61 (4.99) |
|  | Humanitarian aid | 217.41 (9.60) | 85.49 (8.39) | 51.13 (4.75) | 69.35 (10.69) | 56.52 (6.83) | 62.06 (1.99) | 64.91 (5.65) | 66.42 (6.06) |
|  | Government and civil society | 236.45 (10.44) | 105.61 (10.37) | 89.82 (8.34) | 81.34 (12.54) | 88.29 (10.67) | 93.81 (3.00) | 110.72 (9.63) | 120.84 (11.03) |
|  | Education | 235.55 (10.40) | 99.54 (9.77) | 71.59 (6.65) | 48.38 (7.46) | 41.20 (4.98) | 54.24 (1.74) | 52.64 (4.58) | 61.08 (5.58) |
|  | Infrastructure | 52.17 (2.30) | 3.28 (0.32) | 10.78 (1.00) | 1.09 (0.17) | 1.11 (0.13) | 1.70 (0.05) | 11.32 (0.98) | 9.47 (0.86) |
|  | Agriculture | 188.56 (8.33) | 96.20 (9.44) | 94.23 (8.75) | 66.66 (10.27) | 59.56 (7.20) | 65.73 (2.10) | 65.45 (5.69) | 64.54 (5.89) |
|  | Refugees in donor country | 29.25 (1.29) | 21.61 (2.12) | 22.49 (2.09) | 16.95 (2.61) | 35.33 (4.27) | 96.86 (3.10) | 229.59 (19.97) | 275.54 (25.16) |
|  | Donor administration costs | 153.54 (6.78) | 161.74 (15.88) | 124.17 (11.53) | 117.31 (18.08) | 113.05 (13.66) | 118.93 (3.81) | 109.62 (9.53) | 109.88 (10.03) |
|  | Energy | 89.92 (3.97) | 1.72 (0.17) | 34.55 (3.21) | 3.32 (0.51) | 10.92 (1.32) | 9.98 (0.32) | 10.30 (0.90) | 6.68 (0.61) |
|  | Debt relief | 33.72 (1.49) | 72.06 (7.07) | 223.45 (20.75) | 0.00 (0.00) | 133.11 (16.08) | 2310.41 (73.97) | 129.97 (11.31) | 5.30 (0.48) |
|  | Multisector | 88.22 (3.90) | 41.02 (4.03) | 34.23 (3.18) | 22.30 (3.44) | 34.69 (4.19) | 86.70 (2.78) | 106.71 (9.28) | 60.81 (5.55) |
|  | Water and sanitation | 124.88 (5.52) | 27.06 (2.66) | 81.71 (7.59) | 37.73 (5.82) | 26.89 (3.25) | 28.92 (0.93) | 20.38 (1.77) | 55.78 (5.09) |
|  | Financial services and business support | 53.58 (2.37) | 47.07 (4.62) | 17.16 (1.59) | 6.05 (0.93) | 3.02 (0.37) | 9.69 (0.31) | 14.07 (1.22) | 6.63 (0.61) |
|  | Environmental protection | 48.14 (2.13) | 14.40 (1.41) | 7.26 (0.67) | 8.14 (1.26) | 7.51 (0.91) | 4.47 (0.14) | 7.84 (0.68) | 9.22 (0.84) |
|  | Other social services | 137.74 (6.08) | 47.93 (4.70) | 45.55 (4.23) | 27.86 (4.29) | 40.52 (4.89) | 31.41 (1.01) | 30.51 (2.65) | 27.95 (2.55) |
|  | Conflict, peace and security | 66.28 (2.93) | 31.28 (3.07) | 27.92 (2.59) | 20.72 (3.19) | 17.08 (2.06) | 15.23 (0.49) | 19.86 (1.73) | 24.28 (2.22) |
|  | General budget support | 21.68 (0.96) | 5.53 (0.54) | 8.89 (0.83) | 0.61 (0.09) | 2.43 (0.29) | 2.44 (0.08) | 1.19 (0.10) | 0.08 (0.01) |
|  | Unspecified | 123.39 (5.45) | 53.74 (5.28) | 45.05 (4.18) | 39.56 (6.10) | 102.05 (12.33) | 62.84 (2.01) | 96.69 (8.41) | 124.87 (11.40) |
|  | Industry, construction and mining | 184.99 (8.17) | 11.88 (1.17) | 6.50 (0.60) | 11.93 (1.84) | 4.46 (0.54) | 4.22 (0.14) | 5.11 (0.44) | 4.95 (0.45) |
|  | Food aid and commodity assistance | 27.38 (1.21) | 15.52 (1.52) | 9.30 (0.86) | 7.92 (1.22) | 6.56 (0.79) | 6.52 (0.21) | 5.44 (0.47) | 4.14 (0.38) |
|  | Trade policy | 1.47 (0.07) | 0.05 (0.00) | 0.15 (0.01) | 0.69 (0.11) | 0.25 (0.03) | 0.08 (0.00) | 0.37 (0.03) | 0.74 (0.07) |
|  | Tourism | 3.61 (0.16) | 1.32 (0.13) | 0.53 (0.05) | 2.22 (0.34) | 1.06 (0.13) | 1.44 (0.05) | 0.83 (0.07) | 1.51 (0.14) |
| Italy | Health | 79.75 (4.40) | 46.98 (6.74) | 51.06 (5.59) | 62.01 (4.61) | 84.26 (4.10) | 71.62 (2.65) | 130.46 (3.85) | 120.46 (5.36) |
|  | Humanitarian aid | 70.28 (3.88) | 86.66 (12.43) | 61.61 (6.74) | 68.43 (5.08) | 120.31 (5.85) | 186.99 (6.92) | 280.91 (8.29) | 234.41 (10.43) |
|  | Government and civil society | 42.75 (2.36) | 19.76 (2.83) | 34.73 (3.80) | 71.21 (5.29) | 130.86 (6.36) | 55.99 (2.07) | 163.67 (4.83) | 163.96 (7.29) |
|  | Education | 74.60 (4.12) | 55.13 (7.90) | 49.95 (5.47) | 65.29 (4.85) | 106.93 (5.20) | 105.77 (3.92) | 105.69 (3.12) | 123.70 (5.50) |
|  | Infrastructure | 49.32 (2.72) | 20.20 (2.90) | 33.35 (3.65) | 10.72 (0.80) | 36.04 (1.75) | 10.22 (0.38) | 19.84 (0.59) | 21.27 (0.95) |
|  | Agriculture | 46.32 (2.56) | 42.02 (6.03) | 50.10 (5.48) | 49.00 (3.64) | 112.49 (5.47) | 91.22 (3.38) | 107.95 (3.19) | 101.07 (4.50) |
|  | Refugees in donor country | 478.48 (26.40) | 239.72 (34.37) | 375.40 (41.08) | 774.69 (57.55) | 1074.45 (52.23) | 1805.24 (66.84) | 1905.29 (56.23) | 1124.71 (50.03) |
|  | Donor administration costs | 48.54 (2.68) | 33.50 (4.80) | 34.02 (3.72) | 36.59 (2.72) | 39.46 (1.92) | 22.30 (0.83) | 40.86 (1.21) | 45.74 (2.03) |
|  | Energy | 20.36 (1.12) | 10.24 (1.47) | 13.01 (1.42) | 10.23 (0.76) | 31.33 (1.52) | 11.16 (0.41) | 151.36 (4.47) | 11.65 (0.52) |
|  | Debt relief | 727.38 (40.14) | 6.93 (0.99) | 35.66 (3.90) | 26.07 (1.94) | 88.35 (4.29) | 156.97 (5.81) | 217.44 (6.42) | 16.06 (0.71) |
|  | Multisector | 16.44 (0.91) | 11.14 (1.60) | 16.70 (1.83) | 10.07 (0.75) | 16.73 (0.81) | 7.56 (0.28) | 27.53 (0.81) | 86.27 (3.84) |
|  | Water and sanitation | 21.57 (1.19) | 15.20 (2.18) | 10.68 (1.17) | 25.63 (1.90) | 19.75 (0.96) | 18.74 (0.69) | 19.26 (0.57) | 21.59 (0.96) |
|  | Financial services and business support | 3.46 (0.19) | 3.83 (0.55) | 2.78 (0.30) | 2.00 (0.15) | 12.83 (0.62) | 7.81 (0.29) | 20.72 (0.61) | 7.07 (0.31) |
|  | Environmental protection | 11.75 (0.65) | 18.09 (2.59) | 27.40 (3.00) | 37.93 (2.82) | 36.67 (1.78) | 16.45 (0.61) | 40.90 (1.21) | 28.72 (1.28) |
|  | Other social services | 28.05 (1.55) | 24.72 (3.54) | 26.63 (2.91) | 34.15 (2.54) | 47.37 (2.30) | 45.20 (1.67) | 57.04 (1.68) | 47.17 (2.10) |
|  | Conflict, peace and security | 10.32 (0.57) | 2.31 (0.33) | 2.91 (0.32) | 5.74 (0.43) | 18.67 (0.91) | 23.87 (0.88) | 30.59 (0.90) | 20.27 (0.90) |
|  | General budget support | 6.00 (0.33) | 5.62 (0.81) | 6.92 (0.76) | 7.05 (0.52) | 6.06 (0.29) | 0.58 (0.02) | 0.15 (0.00) | 0.54 (0.02) |
|  | Unspecified | 17.66 (0.97) | 25.58 (3.67) | 28.77 (3.15) | 23.49 (1.74) | 34.01 (1.65) | 14.87 (0.55) | 16.74 (0.49) | 36.83 (1.64) |
|  | Industry, construction and mining | 39.61 (2.19) | 21.73 (3.12) | 32.88 (3.60) | 14.15 (1.05) | 22.27 (1.08) | 11.37 (0.42) | 21.66 (0.64) | 20.78 (0.92) |
|  | Food aid and commodity assistance | 16.82 (0.93) | 6.30 (0.90) | 18.35 (2.01) | 11.04 (0.82) | 15.54 (0.76) | 36.74 (1.36) | 27.70 (0.82) | 11.27 (0.50) |
|  | Trade policy | 0.20 (0.01) | 0.11 (0.02) | 0.00 (0.00) | 0.11 (0.01) | 0.16 (0.01) | 0.19 (0.01) | 0.25 (0.01) | 0.09 (0.00) |
|  | Tourism | 2.52 (0.14) | 1.65 (0.24) | 0.87 (0.10) | 0.53 (0.04) | 2.60 (0.13) | 0.14 (0.01) | 2.09 (0.06) | 4.63 (0.21) |
| Australia | Health | 368.38 (11.43) | 409.84 (11.32) | 305.89 (8.77) | 300.28 (9.70) | 268.88 (9.17) | 188.96 (7.75) | 189.20 (7.78) | 298.05 (11.67) |
|  | Humanitarian aid | 325.07 (10.09) | 268.32 (7.41) | 218.21 (6.26) | 248.81 (8.03) | 253.86 (8.66) | 157.64 (6.46) | 205.74 (8.46) | 183.49 (7.19) |
|  | Government and civil society | 602.10 (18.69) | 712.22 (19.68) | 616.86 (17.69) | 523.79 (16.91) | 507.83 (17.33) | 479.01 (19.64) | 470.08 (19.33) | 430.65 (16.86) |
|  | Education | 338.39 (10.50) | 438.35 (12.11) | 343.29 (9.85) | 465.31 (15.03) | 354.65 (12.10) | 230.95 (9.47) | 194.82 (8.01) | 216.10 (8.46) |
|  | Infrastructure | 186.49 (5.79) | 136.24 (3.76) | 135.01 (3.87) | 149.88 (4.84) | 169.19 (5.77) | 134.81 (5.53) | 113.99 (4.69) | 131.94 (5.17) |
|  | Agriculture | 215.87 (6.70) | 292.42 (8.08) | 182.28 (5.23) | 209.16 (6.75) | 152.47 (5.20) | 130.55 (5.35) | 166.35 (6.84) | 167.30 (6.55) |
|  | Refugees in donor country | 0.01 (0.00) | 119.65 (3.31) | 282.48 (8.10) | 0.00 (0.00) | 0.00 (0.00) | 0.00 (0.00) | 0.00 (0.00) | 0.00 (0.00) |
|  | Donor administration costs | 216.68 (6.72) | 260.65 (7.20) | 265.33 (7.61) | 239.31 (7.73) | 192.80 (6.58) | 204.75 (8.40) | 194.93 (8.01) | 195.19 (7.64) |
|  | Energy | 8.51 (0.26) | 34.51 (0.95) | 10.97 (0.31) | 12.06 (0.39) | 12.81 (0.44) | 8.10 (0.33) | 19.79 (0.81) | 25.19 (0.99) |
|  | Debt relief | 9.89 (0.31) | 9.30 (0.26) | 8.85 (0.25) | 6.34 (0.20) | 8.30 (0.28) | 8.05 (0.33) | 3.00 (0.12) | 0.00 (0.00) |
|  | Multisector | 506.66 (15.72) | 470.02 (12.99) | 622.97 (17.87) | 581.36 (18.77) | 618.30 (21.10) | 461.20 (18.91) | 495.22 (20.36) | 459.24 (17.98) |
|  | Water and sanitation | 178.52 (5.54) | 129.59 (3.58) | 143.58 (4.12) | 103.74 (3.35) | 98.81 (3.37) | 62.60 (2.57) | 58.86 (2.42) | 78.69 (3.08) |
|  | Financial services and business support | 26.87 (0.83) | 49.55 (1.37) | 29.10 (0.83) | 39.09 (1.26) | 67.57 (2.31) | 86.38 (3.54) | 99.73 (4.10) | 71.43 (2.80) |
|  | Environmental protection | 78.21 (2.43) | 97.54 (2.70) | 83.65 (2.40) | 32.17 (1.04) | 22.76 (0.78) | 10.45 (0.43) | 28.47 (1.17) | 23.95 (0.94) |
|  | Other social services | 74.03 (2.30) | 60.25 (1.66) | 69.82 (2.00) | 71.90 (2.32) | 57.49 (1.96) | 40.56 (1.66) | 41.32 (1.70) | 51.52 (2.02) |
|  | Conflict, peace and security | 84.69 (2.63) | 55.02 (1.52) | 44.71 (1.28) | 44.45 (1.44) | 37.36 (1.27) | 44.67 (1.83) | 23.26 (0.96) | 59.93 (2.35) |
|  | General budget support | 0.00 (0.00) | 8.89 (0.25) | 5.31 (0.15) | 1.59 (0.05) | 0.00 (0.00) | 2.67 (0.11) | 5.30 (0.22) | 2.73 (0.11) |
|  | Unspecified | -73.07 (-2.27) | 2.49 (0.07) | 31.67 (0.91) | 13.75 (0.44) | 14.62 (0.50) | 90.11 (3.70) | 0.21 (0.01) | 20.58 (0.81) |
|  | Industry, construction and mining | 12.81 (0.40) | 15.13 (0.42) | 30.21 (0.87) | 13.86 (0.45) | 30.23 (1.03) | 22.88 (0.94) | 35.16 (1.45) | 50.18 (1.97) |
|  | Food aid and commodity assistance | 44.52 (1.38) | 31.45 (0.87) | 36.28 (1.04) | 22.32 (0.72) | 24.40 (0.83) | 42.59 (1.75) | 57.07 (2.35) | 57.02 (2.23) |
|  | Trade policy | 17.65 (0.55) | 17.84 (0.49) | 20.06 (0.58) | 17.63 (0.57) | 38.59 (1.32) | 31.51 (1.29) | 29.10 (1.20) | 28.35 (1.11) |
|  | Tourism | 0.01 (0.00) | 0.00 (0.00) | 0.00 (0.00) | 0.00 (0.00) | 0.00 (0.00) | 0.00 (0.00) | 0.81 (0.03) | 2.07 (0.08) |
| Switzerland | Health | 70.00 (3.29) | 73.31 (3.12) | 86.73 (3.67) | 128.29 (4.87) | 114.94 (4.18) | 105.93 (3.70) | 132.23 (5.53) | 126.92 (5.35) |
|  | Humanitarian aid | 266.44 (12.54) | 289.57 (12.33) | 322.89 (13.67) | 364.04 (13.82) | 416.11 (15.13) | 363.16 (12.68) | 339.93 (14.22) | 324.93 (13.71) |
|  | Government and civil society | 143.15 (6.73) | 163.94 (6.98) | 196.76 (8.33) | 209.49 (7.95) | 249.08 (9.06) | 257.10 (8.98) | 297.97 (12.46) | 330.76 (13.95) |
|  | Education | 77.01 (3.62) | 74.80 (3.18) | 83.46 (3.53) | 103.80 (3.94) | 111.61 (4.06) | 119.11 (4.16) | 130.58 (5.46) | 133.54 (5.63) |
|  | Infrastructure | 13.34 (0.63) | 18.12 (0.77) | 22.82 (0.97) | 26.42 (1.00) | 25.42 (0.92) | 31.37 (1.10) | 19.07 (0.80) | 12.11 (0.51) |
|  | Agriculture | 130.61 (6.15) | 122.57 (5.22) | 126.20 (5.34) | 156.94 (5.96) | 185.46 (6.74) | 182.53 (6.37) | 175.10 (7.32) | 173.24 (7.31) |
|  | Refugees in donor country | 476.23 (22.41) | 613.99 (26.14) | 417.45 (17.67) | 445.37 (16.91) | 485.10 (17.64) | 693.99 (24.23) | 333.09 (13.93) | 281.76 (11.89) |
|  | Donor administration costs | 205.20 (9.65) | 195.18 (8.31) | 217.95 (9.22) | 144.93 (5.50) | 154.51 (5.62) | 158.02 (5.52) | 209.05 (8.74) | 229.08 (9.66) |
|  | Energy | 17.00 (0.80) | 29.74 (1.27) | 27.37 (1.16) | 20.54 (0.78) | 14.79 (0.54) | 17.03 (0.59) | 25.46 (1.06) | 29.61 (1.25) |
|  | Debt relief | 70.82 (3.33) | 14.21 (0.60) | 0.00 (0.00) | 0.00 (0.00) | 0.00 (0.00) | 0.00 (0.00) | 0.00 (0.00) | 0.00 (0.00) |
|  | Multisector | 106.12 (4.99) | 161.89 (6.89) | 180.63 (7.64) | 205.96 (7.82) | 218.45 (7.94) | 211.92 (7.40) | 81.95 (3.43) | 103.23 (4.35) |
|  | Water and sanitation | 123.70 (5.82) | 143.65 (6.11) | 150.90 (6.39) | 201.63 (7.65) | 199.09 (7.24) | 144.06 (5.03) | 125.29 (5.24) | 103.38 (4.36) |
|  | Financial services and business support | 49.55 (2.33) | 64.08 (2.73) | 83.86 (3.55) | 115.39 (4.38) | 118.25 (4.30) | 127.98 (4.47) | 60.40 (2.53) | 96.44 (4.07) |
|  | Environmental protection | 86.54 (4.07) | 68.11 (2.90) | 76.55 (3.24) | 90.51 (3.44) | 70.41 (2.56) | 87.06 (3.04) | 65.23 (2.73) | 57.08 (2.41) |
|  | Other social services | 17.04 (0.80) | 26.47 (1.13) | 20.51 (0.87) | 25.33 (0.96) | 34.59 (1.26) | 43.25 (1.51) | 48.43 (2.03) | 36.52 (1.54) |
|  | Conflict, peace and security | 91.80 (4.32) | 90.64 (3.86) | 114.60 (4.85) | 117.89 (4.48) | 87.79 (3.19) | 79.21 (2.77) | 71.46 (2.99) | 73.20 (3.09) |
|  | General budget support | 24.27 (1.14) | 24.04 (1.02) | 35.61 (1.51) | 33.53 (1.27) | 23.61 (0.86) | 21.40 (0.75) | 12.82 (0.54) | 10.74 (0.45) |
|  | Unspecified | 98.85 (4.65) | 109.25 (4.65) | 138.61 (5.87) | 143.52 (5.45) | 156.61 (5.69) | 156.42 (5.46) | 166.50 (6.96) | 153.73 (6.49) |
|  | Industry, construction and mining | 33.41 (1.57) | 17.31 (0.74) | 15.73 (0.67) | 58.31 (2.21) | 39.50 (1.44) | 29.43 (1.03) | 53.33 (2.23) | 52.03 (2.20) |
|  | Food aid and commodity assistance | 0.00 (0.00) | 0.00 (0.00) | 6.72 (0.28) | 4.05 (0.15) | 8.10 (0.29) | 0.00 (0.00) | 6.16 (0.26) | 4.09 (0.17) |
|  | Trade policy | 20.50 (0.96) | 48.41 (2.06) | 33.93 (1.44) | 34.41 (1.31) | 32.87 (1.19) | 30.95 (1.08) | 32.91 (1.38) | 31.35 (1.32) |
|  | Tourism | 3.87 (0.18) | 0.00 (0.00) | 3.52 (0.15) | 4.06 (0.15) | 4.28 (0.16) | 4.48 (0.16) | 3.66 (0.15) | 6.70 (0.28) |
| Denmark | Health | 135.06 (6.71) | 109.54 (5.59) | 90.59 (4.25) | 105.05 (5.06) | 96.98 (4.50) | 82.56 (4.25) | 119.74 (6.32) | 132.16 (7.13) |
|  | Humanitarian aid | 167.41 (8.31) | 164.45 (8.39) | 220.49 (10.35) | 248.58 (11.97) | 198.14 (9.19) | 264.10 (13.60) | 367.11 (19.38) | 359.35 (19.39) |
|  | Government and civil society | 334.72 (16.62) | 336.92 (17.19) | 315.90 (14.83) | 272.36 (13.11) | 398.82 (18.50) | 245.52 (12.65) | 260.95 (13.78) | 230.65 (12.45) |
|  | Education | 176.93 (8.78) | 178.10 (9.09) | 126.70 (5.95) | 104.60 (5.04) | 85.97 (3.99) | 61.31 (3.16) | 85.31 (4.50) | 126.63 (6.83) |
|  | Infrastructure | 94.69 (4.70) | 103.01 (5.26) | 42.56 (2.00) | 29.19 (1.41) | 12.72 (0.59) | 14.13 (0.73) | 7.16 (0.38) | 23.02 (1.24) |
|  | Agriculture | 161.46 (8.02) | 158.06 (8.07) | 125.27 (5.88) | 121.42 (5.85) | 106.41 (4.94) | 115.91 (5.97) | 109.37 (5.77) | 111.61 (6.02) |
|  | Refugees in donor country | 110.29 (5.48) | 137.63 (7.02) | 149.47 (7.02) | 234.19 (11.27) | 432.38 (20.05) | 447.49 (23.05) | 123.51 (6.52) | 67.17 (3.63) |
|  | Donor administration costs | 157.46 (7.82) | 155.53 (7.94) | 149.78 (7.03) | 152.65 (7.35) | 153.12 (7.10) | 150.55 (7.75) | 152.10 (8.03) | 151.96 (8.20) |
|  | Energy | 61.65 (3.06) | 23.00 (1.17) | 75.29 (3.53) | 51.30 (2.47) | 33.94 (1.57) | 25.12 (1.29) | 46.43 (2.45) | 79.91 (4.31) |
|  | Debt relief | 1.00 (0.05) | 0.88 (0.04) | 49.01 (2.30) | 0.21 (0.01) | 0.06 (0.00) | 4.67 (0.24) | 2.53 (0.13) | 3.07 (0.17) |
|  | Multisector | 16.37 (0.81) | 13.99 (0.71) | 9.99 (0.47) | 32.37 (1.56) | 37.20 (1.73) | 14.51 (0.75) | 46.36 (2.45) | 36.86 (1.99) |
|  | Water and sanitation | 88.57 (4.40) | 60.06 (3.06) | 76.46 (3.59) | 110.70 (5.33) | 78.22 (3.63) | 75.29 (3.88) | 42.13 (2.22) | 38.06 (2.05) |
|  | Financial services and business support | 73.61 (3.65) | 70.57 (3.60) | 63.58 (2.99) | 54.96 (2.65) | 83.48 (3.87) | 94.44 (4.86) | 56.76 (3.00) | 63.08 (3.40) |
|  | Environmental protection | 137.39 (6.82) | 103.26 (5.27) | 125.03 (5.87) | 117.66 (5.66) | 84.26 (3.91) | 84.95 (4.38) | 58.23 (3.07) | 43.61 (2.35) |
|  | Other social services | 30.83 (1.53) | 48.11 (2.46) | 42.07 (1.98) | 33.79 (1.63) | 30.18 (1.40) | 25.80 (1.33) | 7.94 (0.42) | 54.72 (2.95) |
|  | Conflict, peace and security | 49.09 (2.44) | 50.62 (2.58) | 48.65 (2.28) | 60.69 (2.92) | 51.56 (2.39) | 53.12 (2.74) | 144.84 (7.65) | 104.77 (5.65) |
|  | General budget support | 61.16 (3.04) | 67.06 (3.42) | 52.11 (2.45) | 34.06 (1.64) | 25.61 (1.19) | 11.31 (0.58) | 11.98 (0.63) | 13.48 (0.73) |
|  | Unspecified | 108.70 (5.40) | 106.02 (5.41) | 307.02 (14.42) | 245.89 (11.84) | 196.05 (9.09) | 144.47 (7.44) | 207.55 (10.96) | 159.19 (8.59) |
|  | Industry, construction and mining | 43.11 (2.14) | 54.90 (2.80) | 38.21 (1.79) | 54.45 (2.62) | 37.07 (1.72) | 17.69 (0.91) | 21.87 (1.15) | 25.44 (1.37) |
|  | Food aid and commodity assistance | 0.74 (0.04) | 3.42 (0.17) | 0.53 (0.02) | 6.47 (0.31) | 2.46 (0.11) | 0.01 (0.00) | 7.99 (0.42) | 21.42 (1.16) |
|  | Trade policy | 3.11 (0.15) | 12.27 (0.63) | 21.12 (0.99) | 5.98 (0.29) | 5.12 (0.24) | 5.95 (0.31) | 14.21 (0.75) | 6.71 (0.36) |
|  | Tourism | 0.75 (0.04) | 2.14 (0.11) | 0.00 (0.00) | 0.51 (0.02) | 6.41 (0.30) | 2.53 (0.13) | 0.00 (0.00) | 0.00 (0.00) |
| Belgium | Health | 160.03 (8.53) | 153.22 (10.38) | 158.16 (12.13) | 164.19 (12.69) | 142.18 (10.75) | 134.62 (8.29) | 136.15 (9.61) | 119.94 (8.88) |
|  | Humanitarian aid | 156.86 (8.36) | 74.66 (5.06) | 135.62 (10.40) | 75.52 (5.84) | 154.61 (11.69) | 266.10 (16.39) | 176.89 (12.48) | 190.09 (14.07) |
|  | Government and civil society | 134.00 (7.14) | 94.95 (6.43) | 73.39 (5.63) | 97.22 (7.51) | 80.26 (6.07) | 89.69 (5.53) | 68.93 (4.86) | 74.14 (5.49) |
|  | Education | 205.73 (10.97) | 202.33 (13.70) | 103.76 (7.96) | 95.95 (7.41) | 88.05 (6.66) | 94.94 (5.85) | 112.68 (7.95) | 112.70 (8.34) |
|  | Infrastructure | 43.14 (2.30) | 29.70 (2.01) | 42.87 (3.29) | 27.80 (2.15) | 31.04 (2.35) | 18.40 (1.13) | 20.50 (1.45) | 16.33 (1.21) |
|  | Agriculture | 144.40 (7.70) | 140.59 (9.52) | 147.47 (11.31) | 140.35 (10.85) | 137.64 (10.41) | 130.61 (8.05) | 120.33 (8.49) | 116.50 (8.62) |
|  | Refugees in donor country | 119.37 (6.36) | 125.74 (8.52) | 149.18 (11.44) | 176.86 (13.67) | 255.03 (19.28) | 414.48 (25.53) | 335.99 (23.71) | 244.50 (18.10) |
|  | Donor administration costs | 93.83 (5.00) | 91.43 (6.19) | 88.26 (6.77) | 88.85 (6.87) | 79.94 (6.04) | 78.83 (4.86) | 81.93 (5.78) | 92.11 (6.82) |
|  | Energy | 48.81 (2.60) | 17.02 (1.15) | 21.45 (1.65) | 9.64 (0.74) | 13.03 (0.99) | 14.52 (0.89) | 22.68 (1.60) | 26.45 (1.96) |
|  | Debt relief | 291.76 (15.55) | 277.65 (18.80) | 17.53 (1.34) | 9.10 (0.70) | 1.48 (0.11) | 5.83 (0.36) | 16.68 (1.18) | 7.20 (0.53) |
|  | Multisector | 64.86 (3.46) | 54.44 (3.69) | 161.58 (12.40) | 148.75 (11.50) | 130.33 (9.85) | 139.50 (8.59) | 116.35 (8.21) | 109.37 (8.10) |
|  | Water and sanitation | 55.86 (2.98) | 43.06 (2.92) | 30.82 (2.36) | 46.69 (3.61) | 34.45 (2.60) | 52.98 (3.26) | 42.07 (2.97) | 41.71 (3.09) |
|  | Financial services and business support | 171.83 (9.16) | 19.62 (1.33) | 5.44 (0.42) | 71.88 (5.55) | 52.00 (3.93) | 51.09 (3.15) | 50.80 (3.59) | 54.22 (4.01) |
|  | Environmental protection | 8.57 (0.46) | 9.68 (0.66) | 15.79 (1.21) | 14.98 (1.16) | 16.65 (1.26) | 19.37 (1.19) | 39.85 (2.81) | 57.22 (4.24) |
|  | Other social services | 33.38 (1.78) | 40.32 (2.73) | 37.21 (2.85) | 29.97 (2.32) | 20.75 (1.57) | 21.88 (1.35) | 18.27 (1.29) | 18.88 (1.40) |
|  | Conflict, peace and security | 29.06 (1.55) | 19.28 (1.31) | 8.65 (0.66) | 5.45 (0.42) | 4.09 (0.31) | 11.28 (0.70) | 5.92 (0.42) | 9.03 (0.67) |
|  | General budget support | 3.99 (0.21) | 0.00 (0.00) | 0.00 (0.00) | 0.00 (0.00) | 0.00 (0.00) | 0.00 (0.00) | 0.00 (0.00) | 0.00 (0.00) |
|  | Unspecified | 66.79 (3.56) | 67.85 (4.60) | 91.34 (7.01) | 80.51 (6.22) | 73.27 (5.54) | 67.46 (4.16) | 43.51 (3.07) | 54.79 (4.06) |
|  | Industry, construction and mining | 21.59 (1.15) | 6.61 (0.45) | 6.69 (0.51) | 2.94 (0.23) | 5.93 (0.45) | 5.21 (0.32) | 5.58 (0.39) | 1.83 (0.14) |
|  | Food aid and commodity assistance | 9.93 (0.53) | 0.32 (0.02) | 0.00 (0.00) | 0.01 (0.00) | 0.00 (0.00) | 0.05 (0.00) | 1.20 (0.08) | 0.00 (0.00) |
|  | Trade policy | 11.89 (0.63) | 7.55 (0.51) | 8.00 (0.61) | 7.07 (0.55) | 1.97 (0.15) | 6.46 (0.40) | 0.55 (0.04) | 3.79 (0.28) |
|  | Tourism | 0.45 (0.02) | 0.46 (0.03) | 0.38 (0.03) | 0.29 (0.02) | 0.01 (0.00) | 0.00 (0.00) | 0.00 (0.00) | 0.00 (0.00) |
| South Korea | Health | 115.71 (9.96) | 152.37 (10.97) | 168.76 (11.31) | 175.33 (11.48) | 183.20 (10.98) | 223.48 (12.71) | 215.37 (12.25) | 204.45 (10.70) |
|  | Humanitarian aid | 27.08 (2.33) | 18.77 (1.35) | 29.12 (1.95) | 75.43 (4.94) | 55.48 (3.33) | 70.61 (4.02) | 100.87 (5.74) | 134.29 (7.03) |
|  | Government and civil society | 58.47 (5.03) | 102.24 (7.36) | 121.69 (8.16) | 88.48 (5.79) | 97.08 (5.82) | 99.93 (5.69) | 92.32 (5.25) | 109.54 (5.74) |
|  | Education | 205.88 (17.73) | 236.93 (17.05) | 234.22 (15.70) | 235.28 (15.40) | 251.73 (15.09) | 261.38 (14.87) | 228.60 (13.01) | 228.42 (11.96) |
|  | Infrastructure | 322.11 (27.74) | 309.70 (22.29) | 327.53 (21.95) | 293.51 (19.21) | 288.18 (17.27) | 344.63 (19.61) | 293.10 (16.68) | 393.11 (20.58) |
|  | Agriculture | 78.74 (6.78) | 98.01 (7.05) | 129.93 (8.71) | 141.83 (9.28) | 148.46 (8.90) | 140.26 (7.98) | 136.67 (7.78) | 163.68 (8.57) |
|  | Refugees in donor country | 0.00 (0.00) | 0.00 (0.00) | 0.00 (0.00) | 0.00 (0.00) | 0.00 (0.00) | 0.00 (0.00) | 0.00 (0.00) | 1.83 (0.10) |
|  | Donor administration costs | 60.18 (5.18) | 66.97 (4.82) | 62.06 (4.16) | 58.60 (3.84) | 70.46 (4.22) | 91.80 (5.22) | 83.99 (4.78) | 85.07 (4.45) |
|  | Energy | 45.76 (3.94) | 61.16 (4.40) | 117.60 (7.88) | 103.71 (6.79) | 185.41 (11.11) | 108.50 (6.17) | 124.73 (7.10) | 73.94 (3.87) |
|  | Debt relief | 0.00 (0.00) | 0.00 (0.00) | 0.00 (0.00) | 0.00 (0.00) | 0.00 (0.00) | 0.00 (0.00) | 0.00 (0.00) | 0.00 (0.00) |
|  | Multisector | 48.24 (4.15) | 65.88 (4.74) | 27.18 (1.82) | 32.60 (2.13) | 30.91 (1.85) | 51.22 (2.91) | 48.01 (2.73) | 49.63 (2.60) |
|  | Water and sanitation | 87.43 (7.53) | 142.56 (10.26) | 115.35 (7.73) | 160.19 (10.48) | 147.98 (8.87) | 115.55 (6.57) | 169.80 (9.66) | 160.42 (8.40) |
|  | Financial services and business support | 3.87 (0.33) | 3.50 (0.25) | 5.04 (0.34) | 5.66 (0.37) | 5.58 (0.33) | 8.80 (0.50) | 9.14 (0.52) | 11.29 (0.59) |
|  | Environmental protection | 19.57 (1.69) | 48.29 (3.48) | 29.99 (2.01) | 14.30 (0.94) | 25.30 (1.52) | 26.82 (1.53) | 19.90 (1.13) | 55.19 (2.89) |
|  | Other social services | 20.37 (1.75) | 20.54 (1.48) | 37.31 (2.50) | 36.58 (2.39) | 38.57 (2.31) | 39.03 (2.22) | 41.10 (2.34) | 56.78 (2.97) |
|  | Conflict, peace and security | 1.95 (0.17) | 2.08 (0.15) | 2.02 (0.14) | 7.17 (0.47) | 21.35 (1.28) | 26.71 (1.52) | 37.52 (2.13) | 46.58 (2.44) |
|  | General budget support | 0.00 (0.00) | 0.00 (0.00) | 0.00 (0.00) | 0.00 (0.00) | 0.00 (0.00) | 0.00 (0.00) | 0.00 (0.00) | 0.00 (0.00) |
|  | Unspecified | 39.63 (3.41) | 29.32 (2.11) | 53.01 (3.55) | 62.87 (4.11) | 75.25 (4.51) | 93.21 (5.30) | 96.50 (5.49) | 92.93 (4.87) |
|  | Industry, construction and mining | 15.33 (1.32) | 18.38 (1.32) | 21.20 (1.42) | 23.52 (1.54) | 32.10 (1.92) | 33.44 (1.90) | 37.38 (2.13) | 22.73 (1.19) |
|  | Food aid and commodity assistance | 2.14 (0.18) | 2.23 (0.16) | 0.02 (0.00) | 1.10 (0.07) | 2.14 (0.13) | 14.40 (0.82) | 13.54 (0.77) | 13.98 (0.73) |
|  | Trade policy | 8.27 (0.71) | 9.00 (0.65) | 8.83 (0.59) | 9.03 (0.59) | 7.40 (0.44) | 6.64 (0.38) | 6.48 (0.37) | 3.06 (0.16) |
|  | Tourism | 0.48 (0.04) | 1.37 (0.10) | 1.17 (0.08) | 2.70 (0.18) | 1.72 (0.10) | 1.38 (0.08) | 2.57 (0.15) | 3.08 (0.16) |
| Austria | Health | 14.42 (3.04) | 10.48 (1.89) | 8.67 (1.63) | 15.08 (2.47) | 7.22 (0.82) | 13.05 (1.20) | 7.27 (1.13) | 6.87 (1.40) |
|  | Humanitarian aid | 13.59 (2.86) | 18.50 (3.34) | 16.38 (3.08) | 19.29 (3.16) | 24.22 (2.76) | 33.06 (3.03) | 62.23 (9.67) | 26.56 (5.40) |
|  | Government and civil society | 29.74 (6.27) | 24.36 (4.40) | 27.36 (5.14) | 16.70 (2.74) | 22.63 (2.58) | 23.62 (2.17) | 22.55 (3.50) | 24.69 (5.02) |
|  | Education | 128.52 (27.08) | 156.08 (28.17) | 138.17 (25.95) | 139.18 (22.81) | 149.32 (17.01) | 158.21 (14.52) | 167.62 (26.04) | 160.05 (32.54) |
|  | Infrastructure | 0.82 (0.17) | 3.41 (0.61) | 1.72 (0.32) | 0.93 (0.15) | 2.16 (0.25) | 4.20 (0.39) | 1.84 (0.29) | 3.44 (0.70) |
|  | Agriculture | 25.22 (5.31) | 15.60 (2.81) | 36.91 (6.93) | 21.72 (3.56) | 21.58 (2.46) | 18.97 (1.74) | 26.65 (4.14) | 22.30 (4.53) |
|  | Refugees in donor country | 40.41 (8.52) | 58.73 (10.60) | 61.44 (11.54) | 104.14 (17.07) | 488.38 (55.64) | 654.32 (60.06) | 163.20 (25.35) | 61.77 (12.56) |
|  | Donor administration costs | 35.28 (7.43) | 32.64 (5.89) | 33.08 (6.21) | 35.36 (5.80) | 35.34 (4.03) | 33.89 (3.11) | 35.07 (5.45) | 33.80 (6.87) |
|  | Energy | 20.37 (4.29) | 12.19 (2.20) | 16.14 (3.03) | 12.90 (2.11) | 17.49 (1.99) | 17.02 (1.56) | 24.05 (3.73) | 19.38 (3.94) |
|  | Debt relief | 41.63 (8.77) | 107.87 (19.47) | 43.60 (8.19) | 103.60 (16.98) | 0.69 (0.08) | 8.57 (0.79) | 10.81 (1.68) | 12.95 (2.63) |
|  | Multisector | 15.43 (3.25) | 6.75 (1.22) | 20.74 (3.90) | 22.45 (3.68) | 17.22 (1.96) | 22.94 (2.11) | 16.04 (2.49) | 19.70 (4.01) |
|  | Water and sanitation | 18.47 (3.89) | 20.78 (3.75) | 18.69 (3.51) | 21.66 (3.55) | 19.45 (2.22) | 17.91 (1.64) | 11.73 (1.82) | 18.17 (3.70) |
|  | Financial services and business support | 18.98 (4.00) | 20.16 (3.64) | 8.77 (1.65) | 17.09 (2.80) | 7.09 (0.81) | 12.47 (1.14) | 6.47 (1.00) | 5.38 (1.09) |
|  | Environmental protection | 7.69 (1.62) | 3.71 (0.67) | 4.72 (0.89) | 9.25 (1.52) | 4.56 (0.52) | 5.10 (0.47) | 5.59 (0.87) | 4.42 (0.90) |
|  | Other social services | 9.07 (1.91) | 9.18 (1.66) | 10.39 (1.95) | 14.51 (2.38) | 11.98 (1.36) | 19.61 (1.80) | 27.97 (4.34) | 21.79 (4.43) |
|  | Conflict, peace and security | 9.42 (1.99) | 10.51 (1.90) | 12.68 (2.38) | 8.98 (1.47) | 3.21 (0.37) | 3.29 (0.30) | 7.49 (1.16) | 3.68 (0.75) |
|  | General budget support | 4.28 (0.90) | 3.28 (0.59) | 1.93 (0.36) | 1.89 (0.31) | 1.85 (0.21) | 0.00 (0.00) | 0.00 (0.00) | 0.00 (0.00) |
|  | Unspecified | 33.50 (7.06) | 34.37 (6.20) | 22.80 (4.28) | 34.25 (5.61) | 37.56 (4.28) | 36.20 (3.32) | 30.12 (4.68) | 29.48 (5.99) |
|  | Industry, construction and mining | 5.07 (1.07) | 3.62 (0.65) | 22.18 (4.17) | 9.22 (1.51) | 4.45 (0.51) | 5.37 (0.49) | 15.48 (2.40) | 15.58 (3.17) |
|  | Food aid and commodity assistance | 0.40 (0.08) | 0.39 (0.07) | 24.11 (4.53) | 0.90 (0.15) | 0.51 (0.06) | 0.24 (0.02) | 0.13 (0.02) | 0.66 (0.13) |
|  | Trade policy | 0.15 (0.03) | 0.03 (0.01) | 0.04 (0.01) | 0.01 (0.00) | 0.01 (0.00) | 0.06 (0.01) | 0.04 (0.01) | 0.07 (0.01) |
|  | Tourism | 2.09 (0.44) | 1.52 (0.27) | 1.89 (0.36) | 1.04 (0.17) | 0.80 (0.09) | 1.33 (0.12) | 1.45 (0.23) | 1.06 (0.22) |
| Finland | Health | 31.97 (3.89) | 34.81 (4.33) | 21.89 (2.81) | 50.76 (5.81) | 26.60 (3.38) | 19.13 (2.73) | 20.23 (3.11) | 21.22 (4.29) |
|  | Humanitarian aid | 112.68 (13.71) | 103.06 (12.81) | 102.48 (13.16) | 117.33 (13.42) | 96.83 (12.29) | 88.75 (12.65) | 71.77 (11.03) | 50.31 (10.18) |
|  | Government and civil society | 83.12 (10.12) | 65.33 (8.12) | 73.71 (9.46) | 85.21 (9.75) | 79.92 (10.14) | 55.92 (7.97) | 64.00 (9.84) | 52.68 (10.66) |
|  | Education | 50.98 (6.20) | 53.58 (6.66) | 48.98 (6.29) | 69.24 (7.92) | 68.10 (8.64) | 50.20 (7.16) | 45.87 (7.05) | 46.20 (9.35) |
|  | Infrastructure | 17.35 (2.11) | 20.33 (2.53) | 8.54 (1.10) | 9.67 (1.11) | 8.15 (1.03) | 5.04 (0.72) | 8.80 (1.35) | 19.10 (3.86) |
|  | Agriculture | 93.74 (11.41) | 85.43 (10.61) | 80.57 (10.34) | 95.60 (10.93) | 86.40 (10.97) | 69.64 (9.93) | 53.68 (8.25) | 27.91 (5.64) |
|  | Refugees in donor country | 33.61 (4.09) | 22.92 (2.85) | 19.76 (2.54) | 14.99 (1.71) | 42.83 (5.44) | 142.98 (20.38) | 82.80 (12.73) | 56.96 (11.52) |
|  | Donor administration costs | 87.35 (10.63) | 81.36 (10.11) | 84.20 (10.81) | 76.30 (8.73) | 58.82 (7.47) | 50.23 (7.16) | 48.78 (7.50) | 48.61 (9.83) |
|  | Energy | 25.92 (3.15) | 28.27 (3.51) | 25.01 (3.21) | 54.75 (6.26) | 32.82 (4.17) | 18.28 (2.61) | 93.48 (14.37) | 20.05 (4.06) |
|  | Debt relief | 0.00 (0.00) | 0.00 (0.00) | 0.00 (0.00) | 0.00 (0.00) | 0.00 (0.00) | 0.00 (0.00) | 0.00 (0.00) | 0.00 (0.00) |
|  | Multisector | 37.99 (4.62) | 40.00 (4.97) | 32.24 (4.14) | 32.14 (3.68) | 25.90 (3.29) | 22.82 (3.25) | 16.65 (2.56) | 13.35 (2.70) |
|  | Water and sanitation | 33.37 (4.06) | 54.53 (6.78) | 55.47 (7.12) | 46.60 (5.33) | 45.75 (5.81) | 36.53 (5.21) | 25.88 (3.98) | 15.89 (3.21) |
|  | Financial services and business support | 28.84 (3.51) | 23.43 (2.91) | 23.57 (3.03) | 24.00 (2.75) | 23.73 (3.01) | 18.59 (2.65) | 16.86 (2.59) | 12.43 (2.51) |
|  | Environmental protection | 30.61 (3.73) | 34.57 (4.29) | 31.52 (4.05) | 18.50 (2.12) | 25.71 (3.26) | 8.06 (1.15) | 2.94 (0.45) | 4.33 (0.88) |
|  | Other social services | 25.84 (3.15) | 24.93 (3.10) | 29.63 (3.80) | 24.97 (2.86) | 23.01 (2.92) | 19.46 (2.77) | 22.80 (3.50) | 20.46 (4.14) |
|  | Conflict, peace and security | 42.67 (5.19) | 37.89 (4.71) | 41.59 (5.34) | 46.07 (5.27) | 49.32 (6.26) | 33.18 (4.73) | 28.58 (4.39) | 25.56 (5.17) |
|  | General budget support | 36.56 (4.45) | 22.59 (2.81) | 30.82 (3.96) | 27.61 (3.16) | 7.30 (0.93) | 0.00 (0.00) | 0.00 (0.00) | 0.00 (0.00) |
|  | Unspecified | 30.77 (3.74) | 58.39 (7.26) | 51.05 (6.55) | 57.95 (6.63) | 56.71 (7.20) | 34.10 (4.86) | 36.14 (5.55) | 40.79 (8.25) |
|  | Industry, construction and mining | 6.16 (0.75) | 6.99 (0.87) | 11.77 (1.51) | 7.28 (0.83) | 10.58 (1.34) | 11.91 (1.70) | 4.77 (0.73) | 5.41 (1.09) |
|  | Food aid and commodity assistance | 0.02 (0.00) | 0.05 (0.01) | 0.05 (0.01) | 1.31 (0.15) | 3.71 (0.47) | 0.09 (0.01) | 0.05 (0.01) | 0.01 (0.00) |
|  | Trade policy | 9.65 (1.17) | 4.37 (0.54) | 5.91 (0.76) | 13.28 (1.52) | 9.15 (1.16) | 13.94 (1.99) | 4.49 (0.69) | 7.24 (1.46) |
|  | Tourism | 2.53 (0.31) | 1.97 (0.24) | 0.21 (0.03) | 0.68 (0.08) | 6.54 (0.83) | 2.68 (0.38) | 2.00 (0.31) | 5.85 (1.18) |
| Ireland | Health | 92.53 (15.86) | 86.07 (15.78) | 92.19 (17.44) | 97.34 (19.04) | 76.35 (16.52) | 74.58 (16.06) | 76.22 (14.66) | 69.09 (13.03) |
|  | Humanitarian aid | 85.53 (14.66) | 106.84 (19.59) | 98.02 (18.55) | 98.24 (19.21) | 96.99 (20.98) | 121.64 (26.19) | 121.55 (23.39) | 125.16 (23.60) |
|  | Government and civil society | 77.38 (13.26) | 64.54 (11.83) | 72.34 (13.69) | 66.44 (12.99) | 59.16 (12.80) | 58.68 (12.63) | 64.23 (12.36) | 59.50 (11.22) |
|  | Education | 63.50 (10.88) | 44.58 (8.17) | 46.69 (8.83) | 44.56 (8.72) | 43.05 (9.31) | 36.88 (7.94) | 39.29 (7.56) | 43.26 (8.16) |
|  | Infrastructure | 1.16 (0.20) | 0.03 (0.00) | 0.38 (0.07) | 0.03 (0.01) | 0.03 (0.01) | 0.03 (0.01) | 0.03 (0.01) | 0.09 (0.02) |
|  | Agriculture | 60.52 (10.37) | 64.54 (11.83) | 62.38 (11.80) | 58.84 (11.51) | 43.83 (9.48) | 51.35 (11.05) | 48.06 (9.25) | 38.63 (7.28) |
|  | Refugees in donor country | 0.28 (0.05) | 0.13 (0.02) | 0.00 (0.00) | 0.33 (0.06) | 0.68 (0.15) | 1.16 (0.25) | 42.94 (8.26) | 56.37 (10.63) |
|  | Donor administration costs | 38.73 (6.64) | 35.72 (6.55) | 33.43 (6.33) | 32.17 (6.29) | 32.42 (7.01) | 33.76 (7.27) | 33.17 (6.38) | 33.10 (6.24) |
|  | Energy | 0.67 (0.11) | 0.10 (0.02) | 0.05 (0.01) | 0.53 (0.10) | 0.00 (0.00) | 0.14 (0.03) | 0.15 (0.03) | 0.08 (0.01) |
|  | Debt relief | 0.00 (0.00) | 0.00 (0.00) | 0.13 (0.02) | 0.13 (0.03) | 0.12 (0.03) | 0.12 (0.03) | 0.00 (0.00) | 0.00 (0.00) |
|  | Multisector | 12.44 (2.13) | 5.52 (1.01) | 5.16 (0.98) | 2.29 (0.45) | 2.25 (0.49) | 3.26 (0.70) | 7.27 (1.40) | 9.74 (1.84) |
|  | Water and sanitation | 10.95 (1.88) | 6.42 (1.18) | 5.81 (1.10) | 6.05 (1.18) | 7.14 (1.54) | 6.51 (1.40) | 6.81 (1.31) | 4.80 (0.91) |
|  | Financial services and business support | 5.81 (1.00) | 6.89 (1.26) | 3.44 (0.65) | 3.80 (0.74) | 3.67 (0.79) | 3.29 (0.71) | 2.87 (0.55) | 2.10 (0.40) |
|  | Environmental protection | 2.88 (0.49) | 3.86 (0.71) | 3.69 (0.70) | 4.16 (0.81) | 3.63 (0.78) | 5.60 (1.21) | 7.42 (1.43) | 7.28 (1.37) |
|  | Other social services | 50.67 (8.68) | 46.06 (8.45) | 28.58 (5.41) | 24.52 (4.80) | 18.70 (4.05) | 30.26 (6.51) | 29.03 (5.59) | 24.36 (4.59) |
|  | Conflict, peace and security | 9.27 (1.59) | 8.73 (1.60) | 8.74 (1.65) | 8.91 (1.74) | 10.04 (2.17) | 9.15 (1.97) | 8.89 (1.71) | 11.78 (2.22) |
|  | General budget support | 28.09 (4.81) | 23.53 (4.32) | 21.97 (4.16) | 11.64 (2.28) | 17.39 (3.76) | 0.00 (0.00) | 0.00 (0.00) | 0.00 (0.00) |
|  | Unspecified | 20.54 (3.52) | 22.18 (4.07) | 22.14 (4.19) | 31.12 (6.09) | 26.58 (5.75) | 24.51 (5.28) | 28.78 (5.54) | 41.77 (7.88) |
|  | Industry, construction and mining | 0.88 (0.15) | 0.75 (0.14) | 0.57 (0.11) | 0.18 (0.04) | 0.80 (0.17) | 1.50 (0.32) | 1.62 (0.31) | 1.06 (0.20) |
|  | Food aid and commodity assistance | 21.16 (3.63) | 17.74 (3.25) | 21.60 (4.09) | 19.16 (3.75) | 18.74 (4.05) | 1.41 (0.30) | 0.77 (0.15) | 0.01 (0.00) |
|  | Trade policy | 0.55 (0.09) | 1.17 (0.21) | 1.25 (0.24) | 0.90 (0.18) | 0.67 (0.14) | 0.64 (0.14) | 0.63 (0.12) | 2.13 (0.40) |
|  | Tourism | 0.01 (0.00) | 0.00 (0.00) | 0.00 (0.00) | 0.00 (0.00) | 0.00 (0.00) | 0.01 (0.00) | 0.01 (0.00) | 0.01 (0.00) |
| Portugal | Health | 16.18 (3.37) | 14.75 (3.35) | 16.23 (4.94) | 18.74 (6.78) | 25.54 (11.93) | 13.49 (7.04) | 5.19 (2.93) | 8.22 (4.38) |
|  | Humanitarian aid | 0.51 (0.11) | 0.09 (0.02) | 0.25 (0.07) | 2.82 (1.02) | 1.13 (0.53) | 9.14 (4.77) | 13.33 (7.52) | 6.88 (3.67) |
|  | Government and civil society | 9.68 (2.02) | 5.14 (1.17) | 5.58 (1.70) | 3.71 (1.34) | 3.50 (1.63) | 2.99 (1.56) | 4.41 (2.49) | 5.17 (2.76) |
|  | Education | 56.59 (11.79) | 52.81 (12.00) | 50.70 (15.44) | 53.71 (19.42) | 46.43 (21.68) | 52.29 (27.30) | 51.83 (29.25) | 57.23 (30.51) |
|  | Infrastructure | 29.68 (6.19) | 33.84 (7.69) | 9.77 (2.97) | 34.42 (12.44) | 5.23 (2.44) | 0.92 (0.48) | 0.41 (0.23) | 2.31 (1.23) |
|  | Agriculture | 3.88 (0.81) | 2.02 (0.46) | 2.38 (0.72) | 1.63 (0.59) | 2.11 (0.99) | 0.88 (0.46) | 1.51 (0.85) | 1.22 (0.65) |
|  | Refugees in donor country | 0.51 (0.11) | 0.83 (0.19) | 1.50 (0.46) | 0.88 (0.32) | 3.13 (1.46) | 4.77 (2.49) | 6.11 (3.45) | 9.54 (5.08) |
|  | Donor administration costs | 13.20 (2.75) | 11.20 (2.55) | 11.30 (3.44) | 7.65 (2.77) | 7.70 (3.60) | 8.27 (4.32) | 9.53 (5.38) | 9.01 (4.81) |
|  | Energy | 19.90 (4.15) | 18.71 (4.25) | 19.50 (5.94) | 8.72 (3.15) | 16.50 (7.70) | 2.22 (1.16) | 0.90 (0.51) | 0.08 (0.04) |
|  | Debt relief | 4.76 (0.99) | 7.01 (1.59) | 8.71 (2.65) | 10.95 (3.96) | 15.97 (7.46) | 19.20 (10.03) | 22.17 (12.51) | 24.81 (13.23) |
|  | Multisector | 4.81 (1.00) | 2.05 (0.47) | 2.79 (0.85) | 3.25 (1.18) | 4.23 (1.97) | 3.73 (1.95) | 4.74 (2.68) | 7.77 (4.14) |
|  | Water and sanitation | 0.59 (0.12) | 0.22 (0.05) | 0.24 (0.07) | 1.57 (0.57) | 0.47 (0.22) | 4.47 (2.33) | 8.90 (5.02) | 9.22 (4.92) |
|  | Financial services and business support | 0.82 (0.17) | 1.27 (0.29) | 1.13 (0.35) | 1.44 (0.52) | 1.22 (0.57) | 1.50 (0.78) | 1.24 (0.70) | 10.25 (5.46) |
|  | Environmental protection | 0.82 (0.17) | 0.34 (0.08) | 0.84 (0.26) | 1.35 (0.49) | 4.22 (1.97) | 1.01 (0.53) | 0.55 (0.31) | 0.51 (0.27) |
|  | Other social services | 24.75 (5.16) | 37.84 (8.60) | 63.44 (19.32) | 63.60 (22.99) | 37.31 (17.42) | 27.77 (14.50) | 23.23 (13.11) | 10.90 (5.81) |
|  | Conflict, peace and security | 12.06 (2.51) | 10.33 (2.35) | 2.98 (0.91) | 3.78 (1.37) | 4.36 (2.04) | 4.61 (2.41) | 6.13 (3.46) | 6.50 (3.47) |
|  | General budget support | 4.53 (0.94) | 3.51 (0.80) | 2.54 (0.77) | 1.14 (0.41) | 1.11 (0.52) | 0.61 (0.32) | 0.60 (0.34) | 0.59 (0.31) |
|  | Unspecified | 1.61 (0.33) | 0.92 (0.21) | 0.68 (0.21) | 0.83 (0.30) | 0.73 (0.34) | 1.24 (0.65) | 1.21 (0.68) | 1.03 (0.55) |
|  | Industry, construction and mining | 0.09 (0.02) | 0.20 (0.04) | 0.13 (0.04) | 0.07 (0.03) | 0.94 (0.44) | 0.06 (0.03) | 0.05 (0.03) | 0.21 (0.11) |
|  | Food aid and commodity assistance | 274.89 (57.28) | 237.10 (53.86) | 127.62 (38.87) | 56.36 (20.37) | 31.74 (14.82) | 32.24 (16.83) | 14.80 (8.35) | 12.62 (6.73) |
|  | Trade policy | 0.01 (0.00) | 0.00 (0.00) | 0.00 (0.00) | 0.00 (0.00) | 0.00 (0.00) | 0.02 (0.01) | 0.07 (0.04) | 0.08 (0.04) |
|  | Tourism | 0.05 (0.01) | 0.01 (0.00) | 0.00 (0.00) | 0.00 (0.00) | 0.56 (0.26) | 0.09 (0.05) | 0.31 (0.17) | 3.41 (1.82) |
| New Zealand | Health | 26.56 (8.10) | 26.80 (7.60) | 23.25 (7.16) | 21.42 (5.86) | 15.92 (4.19) | 13.34 (3.54) | 14.47 (3.94) | 19.14 (4.14) |
|  | Humanitarian aid | 25.61 (7.81) | 21.45 (6.09) | 22.32 (6.87) | 28.02 (7.67) | 23.15 (6.10) | 27.11 (7.20) | 36.22 (9.87) | 38.60 (8.34) |
|  | Government and civil society | 27.98 (8.54) | 30.99 (8.79) | 22.80 (7.02) | 26.10 (7.14) | 27.68 (7.29) | 32.19 (8.55) | 25.63 (6.98) | 38.00 (8.21) |
|  | Education | 54.71 (16.69) | 67.61 (19.18) | 70.70 (21.77) | 65.52 (17.93) | 78.15 (20.59) | 69.68 (18.51) | 72.20 (19.67) | 70.39 (15.21) |
|  | Infrastructure | 19.45 (5.93) | 27.58 (7.83) | 28.82 (8.87) | 26.37 (7.22) | 12.57 (3.31) | 7.68 (2.04) | 13.01 (3.54) | 32.09 (6.94) |
|  | Agriculture | 25.17 (7.68) | 22.15 (6.28) | 23.20 (7.14) | 32.04 (8.77) | 41.65 (10.97) | 43.71 (11.61) | 43.64 (11.89) | 49.61 (10.72) |
|  | Refugees in donor country | 13.59 (4.15) | 18.53 (5.26) | 17.94 (5.52) | 17.63 (4.83) | 17.60 (4.64) | 17.44 (4.63) | 17.13 (4.67) | 16.95 (3.66) |
|  | Donor administration costs | 44.36 (13.53) | 41.41 (11.75) | 40.56 (12.49) | 39.86 (10.91) | 42.31 (11.15) | 43.07 (11.44) | 43.65 (11.89) | 47.04 (10.17) |
|  | Energy | 11.80 (3.60) | 19.51 (5.53) | 12.49 (3.85) | 34.91 (9.56) | 26.43 (6.96) | 22.21 (5.90) | 21.70 (5.91) | 30.08 (6.50) |
|  | Debt relief | 0.00 (0.00) | 0.00 (0.00) | 0.00 (0.00) | 0.00 (0.00) | 0.00 (0.00) | 0.00 (0.00) | 0.00 (0.00) | 0.00 (0.00) |
|  | Multisector | 8.87 (2.71) | 13.21 (3.75) | 7.97 (2.45) | 9.31 (2.55) | 11.75 (3.09) | 14.74 (3.92) | 17.68 (4.81) | 32.86 (7.10) |
|  | Water and sanitation | 5.73 (1.75) | 6.94 (1.97) | 6.88 (2.12) | 8.18 (2.24) | 9.30 (2.45) | 8.30 (2.21) | 8.18 (2.23) | 12.13 (2.62) |
|  | Financial services and business support | 6.35 (1.94) | 7.59 (2.15) | 10.35 (3.19) | 10.13 (2.77) | 7.65 (2.02) | 13.26 (3.52) | 8.39 (2.28) | 24.44 (5.28) |
|  | Environmental protection | 0.92 (0.28) | 1.39 (0.39) | 1.42 (0.44) | 1.84 (0.50) | 8.79 (2.32) | 1.80 (0.48) | 3.29 (0.90) | 2.35 (0.51) |
|  | Other social services | 4.67 (1.42) | 2.55 (0.72) | 3.42 (1.05) | 5.15 (1.41) | 6.55 (1.72) | 5.69 (1.51) | 4.94 (1.35) | 5.30 (1.15) |
|  | Conflict, peace and security | 11.45 (3.49) | 10.88 (3.09) | 6.58 (2.03) | 9.83 (2.69) | 9.97 (2.63) | 11.11 (2.95) | 11.26 (3.07) | 2.53 (0.55) |
|  | General budget support | 15.85 (4.84) | 17.23 (4.89) | 14.30 (4.40) | 14.73 (4.03) | 14.92 (3.93) | 27.50 (7.31) | 15.19 (4.14) | 27.86 (6.02) |
|  | Unspecified | 3.96 (1.21) | 0.35 (0.10) | 0.34 (0.10) | 0.33 (0.09) | 0.35 (0.09) | 0.38 (0.10) | 0.38 (0.10) | 0.69 (0.15) |
|  | Industry, construction and mining | 3.41 (1.04) | 1.93 (0.55) | 1.05 (0.32) | 2.73 (0.75) | 3.02 (0.80) | 0.92 (0.25) | 0.01 (0.00) | 0.58 (0.12) |
|  | Food aid and commodity assistance | 0.50 (0.15) | 0.51 (0.14) | 0.00 (0.00) | 0.00 (0.00) | 0.00 (0.00) | 0.00 (0.00) | 0.00 (0.00) | 0.00 (0.00) |
|  | Trade policy | 3.25 (0.99) | 4.17 (1.18) | 2.86 (0.88) | 4.96 (1.36) | 4.49 (1.18) | 2.38 (0.63) | 2.57 (0.70) | 3.64 (0.79) |
|  | Tourism | 13.62 (4.16) | 9.72 (2.76) | 7.57 (2.33) | 6.26 (1.71) | 17.31 (4.56) | 13.84 (3.68) | 7.57 (2.06) | 8.39 (1.81) |
| Luxembourg | Health | 39.16 (14.46) | 39.95 (14.15) | 48.37 (16.66) | 45.24 (15.86) | 39.87 (13.57) | 41.02 (13.36) | 37.35 (11.36) | 37.61 (10.70) |
|  | Humanitarian aid | 50.49 (18.65) | 46.77 (16.57) | 47.41 (16.33) | 45.26 (15.87) | 47.65 (16.22) | 54.54 (17.77) | 58.48 (17.78) | 60.79 (17.29) |
|  | Government and civil society | 9.80 (3.62) | 13.49 (4.78) | 16.41 (5.65) | 13.08 (4.59) | 23.85 (8.12) | 26.60 (8.67) | 26.53 (8.07) | 25.09 (7.14) |
|  | Education | 29.81 (11.01) | 41.99 (14.88) | 45.45 (15.65) | 49.80 (17.46) | 49.50 (16.85) | 56.73 (18.48) | 43.71 (13.29) | 51.53 (14.66) |
|  | Infrastructure | 0.27 (0.10) | 0.24 (0.09) | 1.49 (0.51) | 3.58 (1.26) | 2.50 (0.85) | 0.47 (0.15) | 11.26 (3.42) | 5.04 (1.43) |
|  | Agriculture | 21.15 (7.81) | 23.24 (8.23) | 23.47 (8.08) | 21.33 (7.48) | 24.06 (8.19) | 26.68 (8.69) | 35.85 (10.90) | 38.75 (11.02) |
|  | Refugees in donor country | 0.00 (0.00) | 0.03 (0.01) | 0.33 (0.11) | 0.00 (0.00) | 0.00 (0.00) | 0.00 (0.00) | 0.00 (0.00) | 0.00 (0.00) |
|  | Donor administration costs | 19.22 (7.10) | 21.00 (7.44) | 21.29 (7.33) | 22.30 (7.82) | 21.48 (7.31) | 20.01 (6.52) | 21.63 (6.58) | 21.43 (6.10) |
|  | Energy | 4.27 (1.58) | 6.46 (2.29) | 3.75 (1.29) | 3.82 (1.34) | 0.33 (0.11) | 0.35 (0.11) | 1.12 (0.34) | 2.07 (0.59) |
|  | Debt relief | 0.00 (0.00) | 0.00 (0.00) | 0.00 (0.00) | 0.00 (0.00) | 0.00 (0.00) | 0.00 (0.00) | 0.00 (0.00) | 0.00 (0.00) |
|  | Multisector | 10.99 (4.06) | 15.42 (5.46) | 9.41 (3.24) | 11.63 (4.08) | 4.19 (1.43) | 6.81 (2.22) | 6.32 (1.92) | 8.31 (2.36) |
|  | Water and sanitation | 20.62 (7.61) | 15.26 (5.41) | 10.15 (3.50) | 12.27 (4.30) | 12.53 (4.27) | 5.79 (1.89) | 16.78 (5.10) | 22.93 (6.52) |
|  | Financial services and business support | 17.06 (6.30) | 17.23 (6.11) | 19.75 (6.80) | 17.28 (6.06) | 15.06 (5.13) | 21.92 (7.14) | 17.93 (5.45) | 22.63 (6.44) |
|  | Environmental protection | 2.04 (0.75) | 1.63 (0.58) | 1.94 (0.67) | 2.35 (0.82) | 2.28 (0.78) | 1.10 (0.36) | 1.41 (0.43) | 0.30 (0.09) |
|  | Other social services | 10.90 (4.03) | 8.95 (3.17) | 7.33 (2.52) | 7.84 (2.75) | 8.54 (2.91) | 9.06 (2.95) | 14.17 (4.31) | 12.45 (3.54) |
|  | Conflict, peace and security | 1.42 (0.53) | 1.35 (0.48) | 2.06 (0.71) | 1.39 (0.49) | 2.45 (0.83) | 2.05 (0.67) | 2.88 (0.87) | 4.00 (1.14) |
|  | General budget support | 0.67 (0.25) | 0.00 (0.00) | 0.00 (0.00) | 0.00 (0.00) | 0.00 (0.00) | 0.00 (0.00) | 0.00 (0.00) | 0.00 (0.00) |
|  | Unspecified | 25.85 (9.55) | 25.83 (9.15) | 26.62 (9.17) | 20.95 (7.35) | 31.86 (10.85) | 26.66 (8.68) | 26.84 (8.16) | 33.14 (9.43) |
|  | Industry, construction and mining | 0.47 (0.18) | 0.25 (0.09) | 0.16 (0.06) | 2.49 (0.87) | 0.48 (0.16) | 0.36 (0.12) | 1.29 (0.39) | 2.07 (0.59) |
|  | Food aid and commodity assistance | 3.08 (1.14) | 1.04 (0.37) | 3.69 (1.27) | 2.80 (0.98) | 3.89 (1.33) | 4.12 (1.34) | 4.72 (1.44) | 0.95 (0.27) |
|  | Trade policy | 1.59 (0.59) | 0.00 (0.00) | 0.00 (0.00) | 0.00 (0.00) | 0.00 (0.00) | 2.46 (0.80) | 0.08 (0.03) | 1.85 (0.53) |
|  | Tourism | 1.94 (0.72) | 2.10 (0.74) | 1.31 (0.45) | 1.76 (0.62) | 3.21 (1.09) | 0.25 (0.08) | 0.60 (0.18) | 0.55 (0.16) |
| Poland | Health | NA | NA | 1.48 (1.11) | 0.76 (0.81) | 1.21 (0.90) | 1.18 (0.60) | 1.08 (0.41) | 1.49 (0.56) |
|  | Humanitarian aid | NA | NA | 4.83 (3.61) | 4.80 (5.10) | 6.98 (5.18) | 35.25 (17.95) | 48.39 (18.42) | 35.61 (13.45) |
|  | Government and civil society | NA | NA | 19.39 (14.48) | 12.36 (13.12) | 19.11 (14.19) | 14.49 (7.38) | 18.16 (6.91) | 75.14 (28.38) |
|  | Education | NA | NA | 63.87 (47.71) | 41.67 (44.25) | 53.06 (39.39) | 43.23 (22.02) | 87.83 (33.44) | 92.87 (35.07) |
|  | Infrastructure | NA | NA | 0.00 (0.00) | 0.30 (0.32) | 0.00 (0.00) | 0.04 (0.02) | 0.03 (0.01) | 0.03 (0.01) |
|  | Agriculture | NA | NA | 1.76 (1.32) | 22.48 (23.87) | 30.20 (22.42) | 81.88 (41.71) | 14.85 (5.65) | 35.39 (13.36) |
|  | Refugees in donor country | NA | NA | 0.00 (0.00) | 0.00 (0.00) | 10.02 (7.44) | 6.50 (3.31) | 6.29 (2.39) | 6.25 (2.36) |
|  | Donor administration costs | NA | NA | 0.34 (0.26) | 0.50 (0.53) | 1.70 (1.26) | 1.87 (0.95) | 2.34 (0.89) | 1.92 (0.72) |
|  | Energy | NA | NA | 0.98 (0.73) | 0.55 (0.58) | 2.25 (1.67) | 0.41 (0.21) | 0.33 (0.13) | 0.26 (0.10) |
|  | Debt relief | NA | NA | 0.00 (0.00) | 0.00 (0.00) | 0.00 (0.00) | 1.10 (0.56) | 0.00 (0.00) | 0.00 (0.00) |
|  | Multisector | NA | NA | 1.16 (0.87) | 1.10 (1.17) | 0.42 (0.31) | 0.51 (0.26) | 60.41 (23.00) | 3.78 (1.43) |
|  | Water and sanitation | NA | NA | 1.40 (1.04) | 1.10 (1.17) | 0.94 (0.70) | 0.36 (0.18) | 0.23 (0.09) | 0.58 (0.22) |
|  | Financial services and business support | NA | NA | 1.07 (0.80) | 0.77 (0.82) | 0.89 (0.66) | 1.26 (0.64) | 1.33 (0.51) | 1.74 (0.66) |
|  | Environmental protection | NA | NA | 4.20 (3.14) | 0.31 (0.33) | 0.46 (0.34) | 0.24 (0.12) | 0.22 (0.08) | 0.22 (0.08) |
|  | Other social services | NA | NA | 3.02 (2.25) | 4.14 (4.40) | 4.52 (3.36) | 4.69 (2.39) | 15.42 (5.87) | 5.53 (2.09) |
|  | Conflict, peace and security | NA | NA | 1.48 (1.11) | 1.22 (1.30) | 1.42 (1.05) | 1.23 (0.63) | 4.26 (1.62) | 2.05 (0.78) |
|  | General budget support | NA | NA | 0.00 (0.00) | 0.00 (0.00) | 0.00 (0.00) | 0.00 (0.00) | 0.00 (0.00) | 0.00 (0.00) |
|  | Unspecified | NA | NA | 28.61 (21.38) | 1.46 (1.56) | 1.11 (0.83) | 0.73 (0.37) | 1.14 (0.44) | 0.68 (0.26) |
|  | Industry, construction and mining | NA | NA | 0.28 (0.21) | 0.48 (0.51) | 0.20 (0.15) | 1.16 (0.59) | 0.02 (0.01) | 0.35 (0.13) |
|  | Food aid and commodity assistance | NA | NA | 0.00 (0.00) | 0.00 (0.00) | 0.00 (0.00) | 0.00 (0.00) | 0.00 (0.00) | 0.00 (0.00) |
|  | Trade policy | NA | NA | 0.00 (0.00) | 0.00 (0.00) | 0.00 (0.00) | 0.01 (0.01) | 0.00 (0.00) | 0.00 (0.00) |
|  | Tourism | NA | NA | 0.00 (0.00) | 0.16 (0.17) | 0.21 (0.16) | 0.19 (0.10) | 0.32 (0.12) | 0.91 (0.34) |
| Greece | Health | 2.19 (1.75) | 1.96 (2.07) | 0.38 (1.01) | 0.41 (1.00) | 0.23 (0.29) | 0.10 (0.06) | 0.10 (0.11) | 0.02 (0.04) |
|  | Humanitarian aid | 1.15 (0.92) | 0.21 (0.22) | 0.20 (0.51) | 1.03 (2.50) | 0.07 (0.09) | 8.58 (5.00) | 13.43 (15.07) | 6.54 (16.92) |
|  | Government and civil society | 0.00 (0.00) | 0.02 (0.02) | 0.02 (0.06) | 0.14 (0.35) | 0.00 (0.00) | 0.00 (0.00) | 0.00 (0.00) | 0.02 (0.04) |
|  | Education | 61.44 (49.15) | 59.45 (62.79) | 9.47 (24.77) | 9.76 (23.68) | 9.83 (12.75) | 1.22 (0.71) | 1.31 (1.47) | 2.24 (5.79) |
|  | Infrastructure | 16.85 (13.48) | 0.10 (0.10) | 0.06 (0.17) | 0.01 (0.04) | 0.01 (0.01) | 0.00 (0.00) | 0.00 (0.00) | 0.00 (0.00) |
|  | Agriculture | 0.29 (0.23) | 0.00 (0.00) | 0.00 (0.00) | 0.00 (0.00) | 0.00 (0.00) | 0.00 (0.00) | 0.00 (0.00) | 0.00 (0.00) |
|  | Refugees in donor country | 20.63 (16.50) | 17.65 (18.64) | 18.72 (48.97) | 19.04 (46.19) | 63.77 (82.66) | 157.99 (92.12) | 71.58 (80.37) | 27.92 (72.24) |
|  | Donor administration costs | 10.39 (8.31) | 9.04 (9.54) | 6.21 (16.24) | 6.50 (15.77) | 0.08 (0.11) | 1.40 (0.82) | 0.00 (0.00) | 0.02 (0.06) |
|  | Energy | 0.00 (0.00) | 0.00 (0.00) | 0.00 (0.00) | 0.00 (0.00) | 0.00 (0.00) | 0.00 (0.00) | 0.00 (0.00) | 0.00 (0.00) |
|  | Debt relief | 0.00 (0.00) | 0.00 (0.00) | 0.00 (0.00) | 0.00 (0.00) | 0.00 (0.00) | 0.00 (0.00) | 0.00 (0.00) | 0.00 (0.00) |
|  | Multisector | 6.88 (5.50) | 4.97 (5.25) | 1.71 (4.47) | 3.17 (7.68) | 2.01 (2.60) | 1.87 (1.09) | 2.32 (2.60) | 1.82 (4.70) |
|  | Water and sanitation | 0.00 (0.00) | 0.10 (0.11) | 0.03 (0.09) | 0.04 (0.10) | 0.00 (0.00) | 0.00 (0.00) | 0.00 (0.00) | 0.00 (0.00) |
|  | Financial services and business support | 0.07 (0.05) | 0.00 (0.00) | 0.00 (0.00) | 0.00 (0.00) | 0.00 (0.00) | 0.00 (0.00) | 0.00 (0.00) | 0.00 (0.00) |
|  | Environmental protection | 0.00 (0.00) | 0.38 (0.40) | 0.08 (0.21) | 0.18 (0.45) | 0.21 (0.27) | 0.28 (0.16) | 0.24 (0.27) | 0.00 (0.01) |
|  | Other social services | 4.55 (3.64) | 0.00 (0.00) | 0.00 (0.00) | 0.40 (0.97) | 0.16 (0.20) | 0.06 (0.03) | 0.01 (0.01) | 0.00 (0.00) |
|  | Conflict, peace and security | 0.56 (0.45) | 0.79 (0.83) | 0.41 (1.08) | 0.44 (1.07) | 0.77 (1.00) | 0.00 (0.00) | 0.00 (0.00) | 0.00 (0.00) |
|  | General budget support | 0.00 (0.00) | 0.00 (0.00) | 0.00 (0.00) | 0.00 (0.00) | 0.00 (0.00) | 0.00 (0.00) | 0.00 (0.00) | 0.00 (0.00) |
|  | Unspecified | 0.00 (0.00) | 0.00 (0.00) | 0.93 (2.43) | 0.09 (0.21) | 0.01 (0.01) | 0.01 (0.00) | 0.08 (0.09) | 0.08 (0.20) |
|  | Industry, construction and mining | 0.00 (0.00) | 0.00 (0.00) | 0.00 (0.00) | 0.00 (0.00) | 0.00 (0.00) | 0.00 (0.00) | 0.00 (0.00) | 0.00 (0.00) |
|  | Food aid and commodity assistance | 0.00 (0.00) | 0.01 (0.01) | 0.00 (0.00) | 0.00 (0.00) | 0.00 (0.00) | 0.00 (0.00) | 0.00 (0.00) | 0.00 (0.00) |
|  | Trade policy | 0.00 (0.00) | 0.00 (0.00) | 0.00 (0.00) | 0.00 (0.00) | 0.00 (0.00) | 0.00 (0.00) | 0.00 (0.00) | 0.00 (0.00) |
|  | Tourism | 0.00 (0.00) | 0.00 (0.00) | 0.00 (0.00) | 0.00 (0.00) | 0.00 (0.00) | 0.00 (0.00) | 0.00 (0.00) | 0.00 (0.00) |
| Czech | Health | 2.90 (4.12) | 2.29 (3.45) | 2.47 (4.40) | 3.63 (5.70) | 3.07 (3.67) | 3.24 (3.88) | 5.42 (6.10) | 1.69 (1.69) |
|  | Humanitarian aid | 3.63 (5.16) | 4.93 (7.45) | 4.71 (8.39) | 7.30 (11.46) | 10.00 (11.97) | 14.37 (17.20) | 8.40 (9.46) | 16.91 (16.88) |
|  | Government and civil society | 7.66 (10.90) | 3.48 (5.26) | 5.60 (10.00) | 4.80 (7.54) | 7.02 (8.41) | 6.18 (7.40) | 9.35 (10.52) | 12.75 (12.73) |
|  | Education | 7.00 (9.95) | 8.07 (12.20) | 8.55 (15.25) | 10.34 (16.24) | 11.44 (13.69) | 9.10 (10.90) | 8.51 (9.57) | 8.50 (8.49) |
|  | Infrastructure | 1.16 (1.64) | 0.27 (0.41) | 0.00 (0.00) | 0.00 (0.00) | 0.03 (0.04) | 0.09 (0.11) | 0.21 (0.23) | 0.09 (0.09) |
|  | Agriculture | 5.28 (7.50) | 4.62 (6.99) | 3.91 (6.98) | 5.03 (7.89) | 5.67 (6.78) | 4.77 (5.71) | 7.10 (7.99) | 6.67 (6.66) |
|  | Refugees in donor country | 10.71 (15.23) | 9.49 (14.34) | 8.78 (15.67) | 11.76 (18.47) | 16.82 (20.13) | 21.07 (25.23) | 23.98 (26.98) | 24.99 (24.95) |
|  | Donor administration costs | 5.82 (8.27) | 4.96 (7.50) | 4.48 (7.99) | 4.51 (7.08) | 4.76 (5.70) | 4.91 (5.88) | 3.63 (4.08) | 4.97 (4.96) |
|  | Energy | 3.43 (4.87) | 3.48 (5.27) | 3.05 (5.45) | 1.52 (2.39) | 5.48 (6.56) | 1.68 (2.02) | 1.82 (2.05) | 1.22 (1.22) |
|  | Debt relief | 0.00 (0.00) | 0.00 (0.00) | 0.00 (0.00) | 0.00 (0.00) | 0.00 (0.00) | 0.00 (0.00) | 0.00 (0.00) | 0.00 (0.00) |
|  | Multisector | 1.86 (2.65) | 0.32 (0.49) | 0.00 (0.00) | 1.37 (2.15) | 5.08 (6.08) | 4.76 (5.70) | 1.80 (2.02) | 1.02 (1.02) |
|  | Water and sanitation | 7.40 (10.52) | 7.07 (10.68) | 5.01 (8.94) | 4.03 (6.33) | 4.03 (4.83) | 3.87 (4.64) | 3.14 (3.53) | 3.84 (3.83) |
|  | Financial services and business support | 0.58 (0.82) | 0.62 (0.94) | 0.51 (0.91) | 0.28 (0.43) | 0.76 (0.91) | 0.48 (0.57) | 0.91 (1.02) | 1.74 (1.74) |
|  | Environmental protection | 0.48 (0.69) | 1.01 (1.52) | 0.97 (1.72) | 1.27 (1.99) | 0.62 (0.74) | 1.00 (1.19) | 0.60 (0.67) | 0.41 (0.41) |
|  | Other social services | 3.10 (4.41) | 2.21 (3.34) | 2.23 (3.97) | 1.72 (2.70) | 2.66 (3.18) | 2.45 (2.93) | 8.40 (9.45) | 1.45 (1.45) |
|  | Conflict, peace and security | 6.91 (9.82) | 9.72 (14.69) | 1.78 (3.17) | 3.09 (4.86) | 3.08 (3.68) | 2.72 (3.26) | 1.44 (1.62) | 1.23 (1.23) |
|  | General budget support | 0.00 (0.00) | 0.00 (0.00) | 0.00 (0.00) | 0.00 (0.00) | 0.00 (0.00) | 0.00 (0.00) | 0.00 (0.00) | 0.00 (0.00) |
|  | Unspecified | 1.70 (2.42) | 2.58 (3.90) | 2.62 (4.68) | 2.31 (3.63) | 2.64 (3.16) | 2.50 (3.00) | 3.57 (4.01) | 12.48 (12.46) |
|  | Industry, construction and mining | 0.65 (0.92) | 0.82 (1.24) | 1.32 (2.36) | 0.69 (1.08) | 0.30 (0.36) | 0.07 (0.08) | 0.36 (0.41) | 0.10 (0.10) |
|  | Food aid and commodity assistance | 0.00 (0.00) | 0.04 (0.06) | 0.00 (0.00) | 0.00 (0.00) | 0.01 (0.01) | 0.19 (0.23) | 0.23 (0.25) | 0.07 (0.07) |
|  | Trade policy | 0.00 (0.00) | 0.15 (0.23) | 0.03 (0.06) | 0.00 (0.00) | 0.05 (0.06) | 0.04 (0.05) | 0.01 (0.01) | 0.00 (0.00) |
|  | Tourism | 0.07 (0.11) | 0.05 (0.07) | 0.04 (0.06) | 0.04 (0.07) | 0.03 (0.03) | 0.01 (0.01) | 0.02 (0.02) | 0.03 (0.03) |
| Hungary | Health | NA | NA | NA | 0.74 (2.57) | 1.44 (2.69) | 0.33 (0.53) | 0.33 (0.80) | 7.81 (6.05) |
|  | Humanitarian aid | NA | NA | NA | 0.55 (1.90) | 1.44 (2.70) | 0.43 (0.70) | 0.18 (0.43) | 7.23 (5.60) |
|  | Government and civil society | NA | NA | NA | 0.18 (0.62) | 0.70 (1.31) | 0.34 (0.56) | 0.32 (0.77) | 1.85 (1.43) |
|  | Education | NA | NA | NA | 7.25 (24.98) | 20.89 (39.08) | 26.99 (43.74) | 26.70 (63.88) | 62.91 (48.72) |
|  | Infrastructure | NA | NA | NA | 0.00 (0.00) | 0.01 (0.02) | 0.00 (0.00) | 0.01 (0.03) | 0.02 (0.02) |
|  | Agriculture | NA | NA | NA | 0.53 (1.83) | 0.43 (0.80) | 4.03 (6.53) | 2.36 (5.63) | 11.23 (8.70) |
|  | Refugees in donor country | NA | NA | NA | 10.04 (34.60) | 10.94 (20.46) | 11.06 (17.92) | 3.54 (8.48) | 3.65 (2.83) |
|  | Donor administration costs | NA | NA | NA | 1.90 (6.54) | 1.46 (2.73) | 0.51 (0.83) | 0.69 (1.65) | 0.00 (0.00) |
|  | Energy | NA | NA | NA | 0.01 (0.05) | 0.00 (0.00) | 0.00 (0.00) | 0.00 (0.00) | 1.12 (0.87) |
|  | Debt relief | NA | NA | NA | 0.00 (0.00) | 0.00 (0.00) | 0.00 (0.00) | 0.00 (0.00) | 0.00 (0.00) |
|  | Multisector | NA | NA | NA | 0.12 (0.41) | 1.65 (3.10) | 0.60 (0.98) | 0.43 (1.03) | 0.45 (0.35) |
|  | Water and sanitation | NA | NA | NA | 1.29 (4.43) | 5.52 (10.33) | 3.07 (4.97) | 2.31 (5.53) | 17.16 (13.29) |
|  | Financial services and business support | NA | NA | NA | 6.10 (21.02) | 0.00 (0.00) | 0.14 (0.23) | 0.20 (0.47) | 0.05 (0.04) |
|  | Environmental protection | NA | NA | NA | 0.06 (0.22) | 0.00 (0.00) | 0.00 (0.00) | 0.00 (0.00) | 0.32 (0.25) |
|  | Other social services | NA | NA | NA | 0.08 (0.28) | 0.05 (0.10) | 0.02 (0.04) | 0.16 (0.39) | 12.40 (9.60) |
|  | Conflict, peace and security | NA | NA | NA | 0.14 (0.50) | 0.00 (0.00) | 0.12 (0.20) | 0.23 (0.56) | 0.06 (0.05) |
|  | General budget support | NA | NA | NA | 0.00 (0.00) | 0.00 (0.00) | 0.00 (0.00) | 0.00 (0.00) | 0.00 (0.00) |
|  | Unspecified | NA | NA | NA | 0.02 (0.06) | 8.91 (16.66) | 14.05 (22.77) | 3.13 (7.50) | 2.84 (2.20) |
|  | Industry, construction and mining | NA | NA | NA | 0.00 (0.00) | 0.00 (0.00) | 0.00 (0.00) | 0.00 (0.00) | 0.00 (0.00) |
|  | Food aid and commodity assistance | NA | NA | NA | 0.00 (0.00) | 0.00 (0.00) | 0.00 (0.00) | 1.20 (2.86) | 0.00 (0.00) |
|  | Trade policy | NA | NA | NA | 0.00 (0.00) | 0.00 (0.01) | 0.00 (0.00) | 0.00 (0.00) | 0.00 (0.00) |
|  | Tourism | NA | NA | NA | 0.00 (0.00) | 0.00 (0.00) | 0.00 (0.00) | 0.00 (0.00) | 0.00 (0.00) |
| Slovak Republic | Health | NA | NA | 0.69 (4.67) | 0.46 (3.06) | 0.30 (1.62) | 0.74 (2.62) | 2.26 (5.96) | 1.63 (5.04) |
|  | Humanitarian aid | NA | NA | 0.46 (3.10) | 0.84 (5.65) | 2.69 (14.27) | 1.28 (4.53) | 1.03 (2.71) | 0.34 (1.05) |
|  | Government and civil society | NA | NA | 1.33 (9.01) | 1.44 (9.61) | 2.70 (14.34) | 1.81 (6.38) | 3.41 (9.01) | 4.05 (12.53) |
|  | Education | NA | NA | 4.45 (30.25) | 4.01 (26.85) | 4.21 (22.33) | 5.24 (18.48) | 4.23 (11.18) | 4.03 (12.46) |
|  | Infrastructure | NA | NA | 0.01 (0.06) | 0.02 (0.16) | 0.03 (0.17) | 0.01 (0.02) | 0.00 (0.00) | 0.00 (0.00) |
|  | Agriculture | NA | NA | 2.05 (13.90) | 0.56 (3.76) | 0.68 (3.61) | 0.67 (2.36) | 0.50 (1.31) | 0.13 (0.39) |
|  | Refugees in donor country | NA | NA | 0.88 (5.96) | 0.90 (6.02) | 1.90 (10.08) | 1.74 (6.12) | 1.42 (3.74) | 0.79 (2.45) |
|  | Donor administration costs | NA | NA | 2.12 (14.41) | 2.01 (13.44) | 1.90 (10.08) | 1.96 (6.92) | 3.20 (8.45) | 2.14 (6.61) |
|  | Energy | NA | NA | 0.13 (0.91) | 0.02 (0.13) | 0.10 (0.53) | 0.29 (1.04) | 0.19 (0.51) | 0.16 (0.51) |
|  | Debt relief | NA | NA | 0.00 (0.00) | 0.00 (0.00) | 0.00 (0.00) | 0.00 (0.00) | 0.00 (0.00) | 0.00 (0.00) |
|  | Multisector | NA | NA | 0.02 (0.12) | 0.09 (0.63) | 1.23 (6.50) | 1.27 (4.46) | 0.00 (0.00) | 0.03 (0.09) |
|  | Water and sanitation | NA | NA | 0.07 (0.51) | 0.07 (0.45) | 0.61 (3.25) | 0.51 (1.80) | 1.56 (4.13) | 0.98 (3.02) |
|  | Financial services and business support | NA | NA | 0.11 (0.73) | 0.03 (0.21) | 0.01 (0.04) | 0.09 (0.31) | 0.04 (0.11) | 0.18 (0.55) |
|  | Environmental protection | NA | NA | 0.18 (1.25) | 0.11 (0.70) | 0.02 (0.12) | 0.04 (0.13) | 0.06 (0.16) | 0.00 (0.00) |
|  | Other social services | NA | NA | 0.28 (1.94) | 0.14 (0.91) | 0.26 (1.36) | 0.09 (0.32) | 0.30 (0.81) | 0.12 (0.38) |
|  | Conflict, peace and security | NA | NA | 1.39 (9.42) | 1.29 (8.61) | 1.75 (9.27) | 1.46 (5.15) | 1.76 (4.64) | 1.24 (3.83) |
|  | General budget support | NA | NA | 0.00 (0.00) | 0.00 (0.00) | 0.00 (0.00) | 0.00 (0.00) | 0.00 (0.00) | 0.00 (0.00) |
|  | Unspecified | NA | NA | 0.54 (3.68) | 2.94 (19.70) | 0.39 (2.07) | 11.05 (38.97) | 17.83 (47.09) | 16.31 (50.42) |
|  | Industry, construction and mining | NA | NA | 0.00 (0.00) | 0.00 (0.00) | 0.06 (0.32) | 0.11 (0.37) | 0.00 (0.00) | 0.08 (0.24) |
|  | Food aid and commodity assistance | NA | NA | 0.00 (0.00) | 0.02 (0.11) | 0.00 (0.01) | 0.00 (0.00) | 0.05 (0.14) | 0.12 (0.36) |
|  | Trade policy | NA | NA | 0.01 (0.07) | 0.00 (0.00) | 0.01 (0.04) | 0.00 (0.01) | 0.01 (0.01) | 0.02 (0.05) |
|  | Tourism | NA | NA | 0.00 (0.00) | 0.00 (0.00) | 0.00 (0.00) | 0.00 (0.00) | 0.01 (0.02) | 0.00 (0.01) |
| Slovenia | Health | 0.45 (2.62) | 1.44 (7.59) | 0.90 (4.62) | 0.15 (0.77) | 0.21 (0.75) | 0.27 (0.86) | 0.26 (0.96) | 0.20 (0.67) |
|  | Humanitarian aid | 0.68 (3.92) | 0.65 (3.42) | 0.45 (2.31) | 0.99 (5.24) | 2.05 (7.33) | 1.95 (6.32) | 2.10 (7.87) | 2.28 (7.78) |
|  | Government and civil society | 2.60 (15.05) | 2.24 (11.83) | 2.09 (10.70) | 1.99 (10.47) | 2.03 (7.26) | 1.20 (3.87) | 1.39 (5.20) | 1.37 (4.69) |
|  | Education | 2.39 (13.83) | 3.82 (20.20) | 4.61 (23.67) | 6.16 (32.44) | 6.74 (24.14) | 7.84 (25.38) | 10.68 (40.03) | 11.63 (39.73) |
|  | Infrastructure | 0.26 (1.48) | 0.31 (1.63) | 0.13 (0.64) | 0.00 (0.00) | 0.00 (0.00) | 0.01 (0.03) | 0.09 (0.33) | 0.06 (0.20) |
|  | Agriculture | 0.23 (1.34) | 0.12 (0.61) | 0.15 (0.77) | 0.06 (0.29) | 0.00 (0.00) | 0.38 (1.23) | 0.03 (0.10) | 0.19 (0.64) |
|  | Refugees in donor country | 0.46 (2.63) | 0.26 (1.37) | 0.09 (0.47) | 0.07 (0.39) | 7.86 (28.14) | 8.02 (25.96) | 1.00 (3.75) | 2.45 (8.36) |
|  | Donor administration costs | 3.50 (20.23) | 4.48 (23.70) | 5.88 (30.17) | 5.34 (28.08) | 3.72 (13.30) | 4.12 (13.33) | 3.54 (13.25) | 3.71 (12.66) |
|  | Energy | 0.66 (3.84) | 0.15 (0.80) | 0.37 (1.91) | 0.27 (1.43) | 0.03 (0.12) | 0.16 (0.51) | 0.38 (1.42) | 2.65 (9.06) |
|  | Debt relief | 0.00 (0.00) | 0.00 (0.00) | 0.00 (0.00) | 0.00 (0.00) | 0.00 (0.00) | 0.00 (0.00) | 0.00 (0.00) | 0.00 (0.00) |
|  | Multisector | 0.03 (0.20) | 0.22 (1.18) | 0.17 (0.87) | 0.17 (0.91) | 0.14 (0.50) | 0.10 (0.32) | 0.10 (0.36) | 0.09 (0.32) |
|  | Water and sanitation | 0.76 (4.40) | 1.63 (8.61) | 0.70 (3.58) | 1.30 (6.85) | 1.27 (4.53) | 2.10 (6.80) | 2.43 (9.10) | 1.57 (5.38) |
|  | Financial services and business support | 0.20 (1.18) | 0.14 (0.72) | 0.20 (1.04) | 0.14 (0.73) | 0.10 (0.37) | 0.80 (2.59) | 0.45 (1.69) | 0.38 (1.31) |
|  | Environmental protection | 0.28 (1.64) | 0.65 (3.46) | 1.01 (5.19) | 0.13 (0.66) | 0.73 (2.62) | 0.25 (0.82) | 0.21 (0.79) | 0.19 (0.65) |
|  | Other social services | 0.45 (2.61) | 0.14 (0.76) | 0.12 (0.60) | 0.08 (0.41) | 0.00 (0.00) | 1.33 (4.31) | 1.61 (6.04) | 0.84 (2.87) |
|  | Conflict, peace and security | 2.09 (12.07) | 1.76 (9.32) | 1.75 (8.98) | 1.51 (7.97) | 2.02 (7.24) | 1.59 (5.16) | 1.73 (6.49) | 1.26 (4.29) |
|  | General budget support | 0.00 (0.00) | 0.00 (0.00) | 0.00 (0.00) | 0.00 (0.00) | 0.00 (0.00) | 0.00 (0.00) | 0.00 (0.00) | 0.00 (0.00) |
|  | Unspecified | 0.61 (3.52) | 0.67 (3.53) | 0.64 (3.29) | 0.49 (2.59) | 0.56 (2.02) | 0.33 (1.06) | 0.38 (1.43) | 0.34 (1.17) |
|  | Industry, construction and mining | 0.43 (2.51) | 0.06 (0.31) | 0.11 (0.57) | 0.15 (0.77) | 0.38 (1.35) | 0.45 (1.45) | 0.31 (1.17) | 0.06 (0.19) |
|  | Food aid and commodity assistance | 0.00 (0.00) | 0.00 (0.00) | 0.02 (0.13) | 0.00 (0.00) | 0.00 (0.00) | 0.00 (0.00) | 0.00 (0.00) | 0.00 (0.00) |
|  | Trade policy | 1.17 (6.76) | 0.04 (0.22) | 0.00 (0.00) | 0.00 (0.00) | 0.00 (0.00) | 0.00 (0.00) | 0.00 (0.01) | 0.00 (0.02) |
|  | Tourism | 0.03 (0.17) | 0.14 (0.74) | 0.09 (0.48) | 0.00 (0.00) | 0.09 (0.33) | 0.00 (0.00) | 0.00 (0.00) | 0.00 (0.00) |
| Iceland | Health | 2.21 (8.48) | 2.60 (9.03) | 4.00 (10.48) | 2.98 (8.06) | 1.66 (4.19) | 2.06 (3.76) | 0.61 (1.11) | 2.45 (4.01) |
|  | Humanitarian aid | 1.73 (6.61) | 1.20 (4.16) | 2.17 (5.69) | 2.30 (6.22) | 4.66 (11.74) | 7.33 (13.36) | 4.25 (7.76) | 6.81 (11.14) |
|  | Government and civil society | 2.26 (8.65) | 2.56 (8.89) | 3.67 (9.61) | 3.38 (9.15) | 2.75 (6.93) | 1.86 (3.40) | 2.71 (4.94) | 4.31 (7.05) |
|  | Education | 1.72 (6.60) | 2.04 (7.09) | 4.19 (10.97) | 2.58 (6.99) | 1.95 (4.90) | 1.68 (3.06) | 0.94 (1.72) | 0.36 (0.59) |
|  | Infrastructure | 0.00 (0.00) | 0.00 (0.00) | 0.00 (0.00) | 0.00 (0.00) | 0.00 (0.00) | 0.07 (0.13) | 0.00 (0.00) | 0.00 (0.00) |
|  | Agriculture | 6.35 (24.34) | 6.37 (22.13) | 8.43 (22.09) | 6.12 (16.56) | 4.40 (11.09) | 4.22 (7.70) | 4.50 (8.21) | 6.73 (11.02) |
|  | Refugees in donor country | 0.22 (0.86) | 0.27 (0.94) | 0.40 (1.06) | 3.04 (8.23) | 6.04 (15.22) | 18.05 (32.91) | 22.43 (40.93) | 11.20 (18.32) |
|  | Donor administration costs | 2.42 (9.26) | 2.35 (8.18) | 2.90 (7.61) | 2.97 (8.04) | 3.07 (7.73) | 3.46 (6.30) | 3.17 (5.78) | 3.47 (5.67) |
|  | Energy | 3.58 (13.72) | 4.70 (16.35) | 4.91 (12.87) | 5.11 (13.81) | 3.99 (10.05) | 4.41 (8.04) | 4.04 (7.37) | 4.75 (7.78) |
|  | Debt relief | 0.21 (0.82) | 0.00 (0.00) | 0.00 (0.00) | 0.00 (0.00) | 0.00 (0.00) | 0.00 (0.00) | 0.00 (0.00) | 0.41 (0.68) |
|  | Multisector | 0.06 (0.23) | 0.41 (1.43) | 1.00 (2.61) | 0.55 (1.48) | 0.09 (0.23) | 0.23 (0.41) | 0.47 (0.86) | 6.99 (11.43) |
|  | Water and sanitation | 0.51 (1.96) | 0.85 (2.97) | 1.27 (3.34) | 1.94 (5.25) | 3.48 (8.76) | 2.95 (5.37) | 1.68 (3.07) | 4.34 (7.09) |
|  | Financial services and business support | 0.18 (0.69) | 0.00 (0.00) | 0.25 (0.66) | 0.12 (0.32) | 0.11 (0.27) | 0.13 (0.24) | 0.00 (0.00) | 0.00 (0.00) |
|  | Environmental protection | 0.26 (1.00) | 0.17 (0.61) | 0.00 (0.00) | 0.00 (0.00) | 0.10 (0.25) | 0.00 (0.00) | 0.08 (0.14) | 0.00 (0.00) |
|  | Other social services | 2.63 (10.08) | 3.47 (12.05) | 3.57 (9.37) | 4.01 (10.86) | 5.95 (14.98) | 6.75 (12.30) | 7.96 (14.53) | 7.63 (12.49) |
|  | Conflict, peace and security | 1.19 (4.56) | 1.14 (3.95) | 0.92 (2.41) | 1.37 (3.72) | 0.98 (2.46) | 0.89 (1.62) | 0.92 (1.68) | 1.07 (1.75) |
|  | General budget support | 0.00 (0.00) | 0.00 (0.00) | 0.00 (0.00) | 0.00 (0.00) | 0.00 (0.00) | 0.00 (0.00) | 0.00 (0.00) | 0.00 (0.00) |
|  | Unspecified | 0.56 (2.13) | 0.39 (1.35) | 0.46 (1.21) | 0.48 (1.31) | 0.48 (1.20) | 0.77 (1.41) | 1.05 (1.92) | 0.60 (0.99) |
|  | Industry, construction and mining | 0.00 (0.00) | 0.25 (0.87) | 0.00 (0.00) | 0.00 (0.00) | 0.00 (0.00) | 0.00 (0.00) | 0.00 (0.00) | 0.00 (0.00) |
|  | Food aid and commodity assistance | 0.00 (0.00) | 0.00 (0.00) | 0.00 (0.00) | 0.00 (0.00) | 0.00 (0.00) | 0.00 (0.00) | 0.00 (0.00) | 0.00 (0.00) |
|  | Trade policy | 0.00 (0.00) | 0.00 (0.00) | 0.00 (0.00) | 0.00 (0.00) | 0.00 (0.00) | 0.00 (0.00) | 0.00 (0.00) | 0.00 (0.00) |
|  | Tourism | 0.00 (0.00) | 0.00 (0.00) | 0.00 (0.00) | 0.00 (0.00) | 0.00 (0.00) | 0.00 (0.00) | 0.00 (0.00) | 0.00 (0.00) |

**Supplementary table s3: Estimated multilateral ODA by sector in million USD at constant price of 2018 and sectoral shares (%) in the 29 DAC member countries, 2011–2018.** The sum of the percentages of bilateral and multilateral breakdown may not necessarily total to 100 given that data on core funding to multilateral agencies were not considered if the sectoral share data were not available in the OECD iLibrary. ODA: official development assistance; DAC: Development Assistance Committee.

| Country | Sector | 2011 | 2012 | 2013 | 2014 | 2015 | 2016 | 2017 | 2018 |
| --- | --- | --- | --- | --- | --- | --- | --- | --- | --- |
| United States | Health | 1514.80 (36.58) | 1834.79 (31.74) | 1830.30 (34.35) | 2223.58 (37.33) | 1099.42 (24.06) | 2400.98 (39.11) | 1599.29 (33.02) | 1486.99 (38.59) |
|  | Humanitarian aid | 85.61 (2.07) | 51.47 (0.89) | 50.62 (0.95) | 69.13 (1.16) | 88.54 (1.94) | 83.49 (1.36) | 89.30 (1.84) | 102.52 (2.66) |
|  | Government and civil society | 293.54 (7.09) | 276.65 (4.79) | 318.68 (5.98) | 299.34 (5.03) | 274.76 (6.01) | 235.08 (3.83) | 193.10 (3.99) | 179.35 (4.65) |
|  | Education | 229.57 (5.54) | 252.26 (4.36) | 191.97 (3.60) | 244.45 (4.10) | 201.66 (4.41) | 208.39 (3.39) | 141.05 (2.91) | 119.42 (3.10) |
|  | Infrastructure | 235.21 (5.68) | 465.30 (8.05) | 364.39 (6.84) | 376.77 (6.33) | 305.25 (6.68) | 244.44 (3.98) | 228.71 (4.72) | 189.76 (4.92) |
|  | Agriculture | 232.25 (5.61) | 330.76 (5.72) | 341.23 (6.40) | 300.64 (5.05) | 241.89 (5.29) | 278.39 (4.54) | 214.66 (4.43) | 216.40 (5.62) |
|  | Refugees in donor country | 0.00 (0.00) | 0.00 (0.00) | 0.00 (0.00) | 0.00 (0.00) | 0.00 (0.00) | 0.00 (0.00) | 0.00 (0.00) | 0.00 (0.00) |
|  | Donor administration costs | 68.41 (1.65) | 117.32 (2.03) | 112.95 (2.12) | 93.90 (1.58) | 95.57 (2.09) | 102.57 (1.67) | 55.04 (1.14) | 62.86 (1.63) |
|  | Energy | 125.69 (3.03) | 263.04 (4.55) | 239.28 (4.49) | 348.51 (5.85) | 262.11 (5.74) | 184.85 (3.01) | 208.74 (4.31) | 170.59 (4.43) |
|  | Debt relief | 0.14 (0.00) | 94.68 (1.64) | 45.28 (0.85) | 33.09 (0.56) | 93.69 (2.05) | 33.39 (0.54) | 28.17 (0.58) | 26.25 (0.68) |
|  | Multisector | 97.96 (2.37) | 127.85 (2.21) | 114.07 (2.14) | 96.62 (1.62) | 105.20 (2.30) | 111.37 (1.81) | 104.55 (2.16) | 94.67 (2.46) |
|  | Water and sanitation | 119.28 (2.88) | 231.15 (4.00) | 180.14 (3.38) | 206.44 (3.47) | 171.10 (3.74) | 190.23 (3.10) | 121.27 (2.50) | 133.03 (3.45) |
|  | Financial services and business support | 81.30 (1.96) | 91.23 (1.58) | 124.41 (2.33) | 124.91 (2.10) | 128.52 (2.81) | 59.97 (0.98) | 79.21 (1.64) | 76.99 (2.00) |
|  | Environmental protection | 137.06 (3.31) | 172.05 (2.98) | 164.00 (3.08) | 180.43 (3.03) | 204.60 (4.48) | 202.11 (3.29) | 144.34 (2.98) | 116.11 (3.01) |
|  | Other social services | 148.96 (3.60) | 217.92 (3.77) | 210.11 (3.94) | 207.31 (3.48) | 211.29 (4.62) | 201.31 (3.28) | 162.05 (3.35) | 150.83 (3.91) |
|  | Conflict, peace and security | 9.66 (0.23) | 9.01 (0.16) | 7.94 (0.15) | 10.09 (0.17) | 8.18 (0.18) | 5.55 (0.09) | 6.17 (0.13) | 5.31 (0.14) |
|  | General budget support | 9.11 (0.22) | 38.01 (0.66) | 34.62 (0.65) | 24.68 (0.41) | 6.96 (0.15) | 14.34 (0.23) | 12.40 (0.26) | 2.35 (0.06) |
|  | Unspecified | 0.04 (0.00) | 20.32 (0.35) | 19.06 (0.36) | 20.16 (0.34) | 110.60 (2.42) | 91.85 (1.50) | 211.01 (4.36) | 121.27 (3.15) |
|  | Industry, construction and mining | 59.29 (1.43) | 56.98 (0.99) | 46.32 (0.87) | 40.67 (0.68) | 45.92 (1.01) | 30.27 (0.49) | 39.58 (0.82) | 29.51 (0.77) |
|  | Food aid and commodity assistance | 4.04 (0.10) | 2.94 (0.05) | 5.60 (0.11) | 4.48 (0.08) | 1.90 (0.04) | 1.92 (0.03) | 3.82 (0.08) | 2.87 (0.07) |
|  | Trade policy | 11.49 (0.28) | 21.95 (0.38) | 26.25 (0.49) | 14.96 (0.25) | 16.59 (0.36) | 43.40 (0.71) | 8.10 (0.17) | 14.39 (0.37) |
|  | Tourism | 1.62 (0.04) | 3.30 (0.06) | 4.16 (0.08) | 4.14 (0.07) | 5.76 (0.13) | 1.94 (0.03) | 1.92 (0.04) | 4.11 (0.11) |
| Germany | Health | 565.27 (11.17) | 485.33 (11.07) | 496.06 (10.86) | 536.05 (11.47) | 466.86 (11.05) | 547.68 (9.81) | 610.73 (11.08) | 584.21 (9.40) |
|  | Humanitarian aid | 345.46 (6.83) | 270.59 (6.17) | 319.99 (7.01) | 385.66 (8.25) | 348.15 (8.24) | 450.92 (8.08) | 529.97 (9.61) | 589.48 (9.49) |
|  | Government and civil society | 445.62 (8.81) | 326.67 (7.45) | 357.03 (7.82) | 353.20 (7.56) | 297.11 (7.03) | 374.63 (6.71) | 485.91 (8.81) | 637.91 (10.27) |
|  | Education | 333.21 (6.58) | 233.68 (5.33) | 229.61 (5.03) | 219.57 (4.70) | 163.07 (3.86) | 264.48 (4.74) | 281.44 (5.11) | 322.80 (5.20) |
|  | Infrastructure | 537.92 (10.63) | 549.42 (12.53) | 587.13 (12.85) | 480.74 (10.28) | 386.06 (9.13) | 484.42 (8.68) | 480.82 (8.72) | 676.32 (10.89) |
|  | Agriculture | 395.98 (7.82) | 251.28 (5.73) | 350.33 (7.67) | 355.99 (7.62) | 250.73 (5.93) | 439.87 (7.88) | 381.50 (6.92) | 447.98 (7.21) |
|  | Refugees in donor country | 0.00 (0.00) | 0.00 (0.00) | 2.67 (0.06) | 4.12 (0.09) | 0.00 (0.00) | 0.00 (0.00) | 0.00 (0.00) | 0.00 (0.00) |
|  | Donor administration costs | 117.22 (2.32) | 112.89 (2.57) | 121.78 (2.67) | 133.41 (2.85) | 126.68 (3.00) | 122.89 (2.20) | 140.18 (2.54) | 157.95 (2.54) |
|  | Energy | 366.91 (7.25) | 341.69 (7.79) | 325.49 (7.13) | 337.91 (7.23) | 221.77 (5.25) | 344.32 (6.17) | 403.13 (7.31) | 426.97 (6.87) |
|  | Debt relief | 10.89 (0.22) | 62.53 (1.43) | 7.39 (0.16) | 0.87 (0.02) | 1.48 (0.03) | 24.26 (0.43) | 16.40 (0.30) | 33.94 (0.55) |
|  | Multisector | 139.47 (2.76) | 145.35 (3.31) | 166.41 (3.64) | 166.04 (3.55) | 139.37 (3.30) | 208.13 (3.73) | 263.37 (4.78) | 276.11 (4.44) |
|  | Water and sanitation | 207.48 (4.10) | 190.15 (4.34) | 189.43 (4.15) | 217.57 (4.65) | 181.77 (4.30) | 200.81 (3.60) | 175.96 (3.19) | 303.66 (4.89) |
|  | Financial services and business support | 282.94 (5.59) | 290.43 (6.62) | 329.30 (7.21) | 377.27 (8.07) | 389.05 (9.21) | 358.76 (6.43) | 278.67 (5.06) | 351.77 (5.66) |
|  | Environmental protection | 223.14 (4.41) | 188.27 (4.29) | 195.01 (4.27) | 206.54 (4.42) | 182.79 (4.33) | 228.35 (4.09) | 200.23 (3.63) | 186.19 (3.00) |
|  | Other social services | 194.07 (3.83) | 159.37 (3.63) | 179.44 (3.93) | 176.44 (3.77) | 152.69 (3.61) | 180.67 (3.24) | 158.55 (2.88) | 197.97 (3.19) |
|  | Conflict, peace and security | 102.65 (2.03) | 56.04 (1.28) | 84.14 (1.84) | 101.42 (2.17) | 102.16 (2.42) | 115.22 (2.06) | 127.10 (2.31) | 174.91 (2.82) |
|  | General budget support | 189.43 (3.74) | 173.29 (3.95) | 180.56 (3.95) | 203.17 (4.35) | 149.94 (3.55) | 119.51 (2.14) | 118.76 (2.15) | 97.85 (1.58) |
|  | Unspecified | 79.28 (1.57) | 37.24 (0.85) | 35.15 (0.77) | 24.31 (0.52) | 51.67 (1.22) | 90.25 (1.62) | 44.49 (0.81) | 75.36 (1.21) |
|  | Industry, construction and mining | 193.53 (3.82) | 205.62 (4.69) | 79.14 (1.73) | 71.02 (1.52) | 85.89 (2.03) | 69.00 (1.24) | 153.45 (2.78) | 145.43 (2.34) |
|  | Food aid and commodity assistance | 45.96 (0.91) | 43.23 (0.99) | 38.59 (0.84) | 39.35 (0.84) | 57.52 (1.36) | 45.73 (0.82) | 35.58 (0.65) | 70.37 (1.13) |
|  | Trade policy | 48.88 (0.97) | 32.65 (0.74) | 45.87 (1.00) | 44.73 (0.96) | 34.46 (0.82) | 48.33 (0.87) | 39.75 (0.72) | 44.71 (0.72) |
|  | Tourism | 3.06 (0.06) | 2.70 (0.06) | 4.98 (0.11) | 3.33 (0.07) | 2.19 (0.05) | 5.91 (0.11) | 7.60 (0.14) | 6.77 (0.11) |
| Japan | Health | 360.67 (11.17) | 459.73 (14.62) | 315.53 (11.57) | 465.33 (14.18) | 424.92 (12.78) | 414.47 (12.53) | 545.23 (15.88) | 552.36 (13.93) |
|  | Humanitarian aid | 120.69 (3.74) | 49.40 (1.57) | 51.44 (1.89) | 58.82 (1.79) | 87.75 (2.64) | 114.33 (3.46) | 134.59 (3.92) | 159.88 (4.03) |
|  | Government and civil society | 339.47 (10.52) | 187.69 (5.97) | 301.12 (11.04) | 203.39 (6.20) | 264.69 (7.96) | 249.24 (7.53) | 244.54 (7.12) | 282.39 (7.12) |
|  | Education | 301.98 (9.35) | 198.70 (6.32) | 158.45 (5.81) | 191.08 (5.82) | 204.28 (6.14) | 237.89 (7.19) | 189.85 (5.53) | 243.53 (6.14) |
|  | Infrastructure | 372.44 (11.54) | 390.14 (12.41) | 333.51 (12.23) | 336.37 (10.25) | 310.03 (9.32) | 277.30 (8.38) | 296.22 (8.63) | 446.29 (11.25) |
|  | Agriculture | 333.78 (10.34) | 249.69 (7.94) | 234.58 (8.60) | 236.36 (7.20) | 241.48 (7.26) | 297.80 (9.00) | 281.70 (8.21) | 334.47 (8.43) |
|  | Refugees in donor country | 0.00 (0.00) | 0.00 (0.00) | 0.00 (0.00) | 0.00 (0.00) | 0.00 (0.00) | 0.00 (0.00) | 0.00 (0.00) | 0.00 (0.00) |
|  | Donor administration costs | 21.11 (0.65) | 23.55 (0.75) | 27.36 (1.00) | 22.39 (0.68) | 21.49 (0.65) | 11.44 (0.35) | 7.71 (0.22) | 8.14 (0.21) |
|  | Energy | 166.55 (5.16) | 186.23 (5.92) | 195.55 (7.17) | 312.63 (9.52) | 269.93 (8.12) | 219.52 (6.64) | 265.54 (7.74) | 334.50 (8.44) |
|  | Debt relief | 22.53 (0.70) | 46.59 (1.48) | 32.26 (1.18) | 35.96 (1.10) | 89.90 (2.70) | 27.22 (0.82) | 17.14 (0.50) | 20.40 (0.51) |
|  | Multisector | 83.31 (2.58) | 89.21 (2.84) | 79.29 (2.91) | 59.56 (1.81) | 84.21 (2.53) | 90.64 (2.74) | 81.35 (2.37) | 192.31 (4.85) |
|  | Water and sanitation | 177.44 (5.50) | 168.34 (5.35) | 156.52 (5.74) | 182.50 (5.56) | 172.46 (5.19) | 205.30 (6.21) | 161.69 (4.71) | 278.75 (7.03) |
|  | Financial services and business support | 107.87 (3.34) | 70.49 (2.24) | 102.20 (3.75) | 118.25 (3.60) | 133.80 (4.02) | 84.26 (2.55) | 113.33 (3.30) | 140.52 (3.54) |
|  | Environmental protection | 214.81 (6.65) | 117.87 (3.75) | 108.24 (3.97) | 133.48 (4.07) | 170.04 (5.11) | 150.72 (4.56) | 148.77 (4.33) | 30.60 (0.77) |
|  | Other social services | 148.29 (4.59) | 117.95 (3.75) | 115.41 (4.23) | 137.31 (4.18) | 146.90 (4.42) | 190.04 (5.75) | 187.99 (5.48) | 191.81 (4.84) |
|  | Conflict, peace and security | 8.92 (0.28) | 5.35 (0.17) | 5.67 (0.21) | 6.82 (0.21) | 6.51 (0.20) | 3.73 (0.11) | 6.66 (0.19) | 7.45 (0.19) |
|  | General budget support | 40.51 (1.26) | 17.00 (0.54) | 93.14 (3.42) | 192.47 (5.86) | 6.25 (0.19) | 10.77 (0.33) | 9.85 (0.29) | 0.76 (0.02) |
|  | Unspecified | 0.03 (0.00) | 3.11 (0.10) | 6.28 (0.23) | 6.89 (0.21) | 60.70 (1.83) | 43.51 (1.32) | 44.26 (1.29) | 61.82 (1.56) |
|  | Industry, construction and mining | 80.62 (2.50) | 43.82 (1.39) | 37.28 (1.37) | 32.51 (0.99) | 46.38 (1.39) | 32.38 (0.98) | 56.32 (1.64) | 50.98 (1.29) |
|  | Food aid and commodity assistance | 4.90 (0.15) | 2.46 (0.08) | 3.95 (0.14) | 3.41 (0.10) | 2.11 (0.06) | 2.43 (0.07) | 5.17 (0.15) | 5.35 (0.14) |
|  | Trade policy | 13.85 (0.43) | 16.26 (0.52) | 17.45 (0.64) | 10.18 (0.31) | 16.36 (0.49) | 47.27 (1.43) | 11.65 (0.34) | 24.07 (0.61) |
|  | Tourism | 2.72 (0.08) | 2.26 (0.07) | 4.31 (0.16) | 4.23 (0.13) | 5.66 (0.17) | 2.04 (0.06) | 3.07 (0.09) | 7.80 (0.20) |
| United Kingdom | Health | 682.38 (13.50) | 641.69 (12.71) | 1488.91 (21.93) | 1105.19 (15.91) | 834.68 (13.19) | 785.46 (11.71) | 1063.35 (14.88) | 1131.23 (15.86) |
|  | Humanitarian aid | 343.00 (6.78) | 227.85 (4.51) | 295.15 (4.35) | 360.56 (5.19) | 368.88 (5.83) | 429.80 (6.41) | 472.00 (6.61) | 596.63 (8.37) |
|  | Government and civil society | 462.82 (9.15) | 351.94 (6.97) | 429.09 (6.32) | 492.84 (7.09) | 461.36 (7.29) | 425.33 (6.34) | 457.71 (6.41) | 578.88 (8.12) |
|  | Education | 361.46 (7.15) | 275.01 (5.45) | 298.93 (4.40) | 407.28 (5.86) | 329.64 (5.21) | 362.99 (5.41) | 334.55 (4.68) | 396.30 (5.56) |
|  | Infrastructure | 501.09 (9.91) | 517.67 (10.25) | 628.96 (9.26) | 613.43 (8.83) | 521.27 (8.24) | 510.69 (7.61) | 474.59 (6.64) | 722.23 (10.13) |
|  | Agriculture | 390.46 (7.72) | 276.21 (5.47) | 396.61 (5.84) | 477.88 (6.88) | 365.48 (5.78) | 482.16 (7.19) | 428.85 (6.00) | 482.78 (6.77) |
|  | Refugees in donor country | 0.00 (0.00) | 0.00 (0.00) | 1.88 (0.03) | 2.48 (0.04) | 0.00 (0.00) | 0.00 (0.00) | 0.00 (0.00) | 0.00 (0.00) |
|  | Donor administration costs | 127.95 (2.53) | 128.63 (2.55) | 144.72 (2.13) | 139.80 (2.01) | 132.98 (2.10) | 123.47 (1.84) | 121.03 (1.69) | 122.89 (1.72) |
|  | Energy | 317.90 (6.29) | 323.89 (6.42) | 370.68 (5.46) | 503.34 (7.25) | 362.07 (5.72) | 370.50 (5.52) | 420.42 (5.88) | 482.07 (6.76) |
|  | Debt relief | 15.15 (0.30) | 80.90 (1.60) | 14.75 (0.22) | 9.07 (0.13) | 91.14 (1.44) | 9.03 (0.13) | 8.44 (0.12) | 9.61 (0.13) |
|  | Multisector | 138.77 (2.74) | 153.70 (3.04) | 183.74 (2.71) | 171.74 (2.47) | 175.96 (2.78) | 223.19 (3.33) | 221.40 (3.10) | 245.92 (3.45) |
|  | Water and sanitation | 198.42 (3.92) | 200.70 (3.98) | 237.31 (3.50) | 299.81 (4.32) | 241.08 (3.81) | 262.10 (3.91) | 208.66 (2.92) | 387.78 (5.44) |
|  | Financial services and business support | 233.68 (4.62) | 229.87 (4.55) | 308.25 (4.54) | 325.59 (4.69) | 391.76 (6.19) | 288.46 (4.30) | 243.62 (3.41) | 326.42 (4.58) |
|  | Environmental protection | 175.69 (3.48) | 162.82 (3.22) | 177.49 (2.61) | 127.74 (1.84) | 95.11 (1.50) | 201.36 (3.00) | 165.22 (2.31) | 129.47 (1.82) |
|  | Other social services | 205.44 (4.06) | 171.07 (3.39) | 231.41 (3.41) | 254.77 (3.67) | 241.37 (3.81) | 246.12 (3.67) | 244.66 (3.42) | 283.96 (3.98) |
|  | Conflict, peace and security | 85.89 (1.70) | 45.27 (0.90) | 75.12 (1.11) | 75.73 (1.09) | 87.73 (1.39) | 77.77 (1.16) | 75.36 (1.05) | 99.95 (1.40) |
|  | General budget support | 160.16 (3.17) | 139.72 (2.77) | 196.45 (2.89) | 144.73 (2.08) | 266.37 (4.21) | 712.81 (10.63) | 1062.06 (14.86) | 166.97 (2.34) |
|  | Unspecified | 55.15 (1.09) | 36.00 (0.71) | 34.18 (0.50) | 28.93 (0.42) | 65.51 (1.04) | 102.93 (1.53) | 78.20 (1.09) | 73.18 (1.03) |
|  | Industry, construction and mining | 159.18 (3.15) | 161.00 (3.19) | 82.91 (1.22) | 80.51 (1.16) | 103.94 (1.64) | 69.30 (1.03) | 131.40 (1.84) | 127.54 (1.79) |
|  | Food aid and commodity assistance | 34.88 (0.69) | 30.85 (0.61) | 31.31 (0.46) | 24.02 (0.35) | 35.61 (0.56) | 30.09 (0.45) | 26.19 (0.37) | 40.63 (0.57) |
|  | Trade policy | 39.48 (0.78) | 29.95 (0.59) | 49.83 (0.73) | 43.10 (0.62) | 41.19 (0.65) | 67.65 (1.01) | 31.78 (0.44) | 47.58 (0.67) |
|  | Tourism | 2.39 (0.05) | 2.36 (0.05) | 5.27 (0.08) | 4.35 (0.06) | 6.52 (0.10) | 4.67 (0.07) | 6.45 (0.09) | 10.97 (0.15) |
| France | Health | 599.11 (14.04) | 600.06 (15.10) | 597.93 (14.15) | 598.70 (15.45) | 592.45 (13.20) | 572.74 (12.28) | 674.53 (12.76) | 666.43 (11.42) |
|  | Humanitarian aid | 261.57 (6.13) | 211.27 (5.32) | 253.27 (5.99) | 296.89 (7.66) | 322.33 (7.18) | 414.20 (8.88) | 360.00 (6.81) | 395.82 (6.78) |
|  | Government and civil society | 300.47 (7.04) | 251.66 (6.33) | 275.76 (6.53) | 276.35 (7.13) | 342.47 (7.63) | 301.53 (6.47) | 386.09 (7.31) | 551.60 (9.45) |
|  | Education | 215.64 (5.05) | 175.11 (4.41) | 173.23 (4.10) | 169.96 (4.39) | 223.44 (4.98) | 220.10 (4.72) | 231.29 (4.38) | 304.88 (5.22) |
|  | Infrastructure | 397.71 (9.32) | 427.56 (10.76) | 460.57 (10.90) | 367.17 (9.48) | 416.60 (9.28) | 413.31 (8.86) | 452.03 (8.55) | 601.30 (10.30) |
|  | Agriculture | 263.03 (6.16) | 184.42 (4.64) | 236.72 (5.60) | 248.60 (6.42) | 286.47 (6.38) | 338.41 (7.26) | 305.76 (5.79) | 406.11 (6.96) |
|  | Refugees in donor country | 0.00 (0.00) | 0.00 (0.00) | 2.23 (0.05) | 3.28 (0.08) | 0.00 (0.00) | 0.00 (0.00) | 0.00 (0.00) | 0.00 (0.00) |
|  | Donor administration costs | 106.20 (2.49) | 95.21 (2.40) | 106.04 (2.51) | 107.32 (2.77) | 103.40 (2.30) | 106.59 (2.29) | 110.20 (2.09) | 114.55 (1.96) |
|  | Energy | 271.91 (6.37) | 265.55 (6.68) | 247.60 (5.86) | 258.06 (6.66) | 270.34 (6.02) | 290.26 (6.22) | 352.55 (6.67) | 401.74 (6.88) |
|  | Debt relief | 25.45 (0.60) | 75.34 (1.90) | 22.42 (0.53) | 16.30 (0.42) | 54.85 (1.22) | 16.02 (0.34) | 14.00 (0.26) | 15.92 (0.27) |
|  | Multisector | 109.37 (2.56) | 114.56 (2.88) | 132.22 (3.13) | 128.15 (3.31) | 137.22 (3.06) | 181.92 (3.90) | 256.61 (4.86) | 241.52 (4.14) |
|  | Water and sanitation | 141.90 (3.32) | 142.31 (3.58) | 137.96 (3.27) | 158.79 (4.10) | 183.95 (4.10) | 156.29 (3.35) | 148.51 (2.81) | 279.95 (4.80) |
|  | Financial services and business support | 217.83 (5.10) | 234.20 (5.89) | 265.29 (6.28) | 298.95 (7.72) | 365.26 (8.14) | 313.58 (6.72) | 225.78 (4.27) | 319.44 (5.47) |
|  | Environmental protection | 104.25 (2.44) | 116.86 (2.94) | 86.69 (2.05) | 78.64 (2.03) | 124.17 (2.77) | 147.27 (3.16) | 125.33 (2.37) | 115.05 (1.97) |
|  | Other social services | 128.74 (3.02) | 122.27 (3.08) | 137.46 (3.25) | 138.59 (3.58) | 180.09 (4.01) | 144.26 (3.09) | 122.65 (2.32) | 207.00 (3.55) |
|  | Conflict, peace and security | 85.67 (2.01) | 43.56 (1.10) | 69.95 (1.66) | 78.42 (2.02) | 80.48 (1.79) | 90.79 (1.95) | 91.90 (1.74) | 114.77 (1.97) |
|  | General budget support | 386.92 (9.07) | 305.90 (7.70) | 455.27 (10.78) | 157.29 (4.06) | 117.42 (2.62) | 105.02 (2.25) | 107.67 (2.04) | 77.56 (1.33) |
|  | Unspecified | 67.30 (1.58) | 30.20 (0.76) | 27.43 (0.65) | 15.88 (0.41) | 40.43 (0.90) | 45.94 (0.98) | 36.72 (0.69) | 169.67 (2.91) |
|  | Industry, construction and mining | 147.59 (3.46) | 165.68 (4.17) | 61.68 (1.46) | 55.36 (1.43) | 90.41 (2.01) | 58.69 (1.26) | 123.66 (2.34) | 129.78 (2.22) |
|  | Food aid and commodity assistance | 36.71 (0.86) | 32.85 (0.83) | 31.07 (0.74) | 18.75 (0.48) | 36.02 (0.80) | 32.18 (0.69) | 27.95 (0.53) | 50.23 (0.86) |
|  | Trade policy | 37.93 (0.89) | 25.65 (0.65) | 36.23 (0.86) | 35.72 (0.92) | 36.11 (0.80) | 39.65 (0.85) | 31.65 (0.60) | 42.21 (0.72) |
|  | Tourism | 2.38 (0.06) | 2.11 (0.05) | 3.76 (0.09) | 2.47 (0.06) | 4.31 (0.10) | 5.14 (0.11) | 5.74 (0.11) | 7.27 (0.12) |
| Netherlands | Health | 287.28 (15.62) | 236.95 (14.56) | 299.76 (17.95) | 253.34 (17.58) | 237.46 (13.74) | 267.32 (13.38) | 251.43 (16.49) | 247.47 (13.23) |
|  | Humanitarian aid | 165.26 (8.98) | 133.87 (8.23) | 148.28 (8.88) | 144.17 (10.00) | 158.18 (9.15) | 174.39 (8.73) | 272.99 (17.91) | 202.62 (10.83) |
|  | Government and civil society | 158.65 (8.62) | 119.55 (7.35) | 120.98 (7.24) | 95.45 (6.62) | 133.29 (7.71) | 158.64 (7.94) | 106.03 (6.95) | 180.13 (9.63) |
|  | Education | 97.92 (5.32) | 76.77 (4.72) | 74.48 (4.46) | 51.76 (3.59) | 93.06 (5.38) | 123.50 (6.18) | 63.03 (4.13) | 97.78 (5.23) |
|  | Infrastructure | 123.96 (6.74) | 121.39 (7.46) | 129.24 (7.74) | 78.60 (5.45) | 119.61 (6.92) | 143.52 (7.18) | 81.82 (5.37) | 153.14 (8.19) |
|  | Agriculture | 100.22 (5.45) | 63.85 (3.92) | 75.23 (4.50) | 57.67 (4.00) | 94.01 (5.44) | 154.39 (7.73) | 51.07 (3.35) | 106.71 (5.70) |
|  | Refugees in donor country | 0.00 (0.00) | 0.00 (0.00) | 0.64 (0.04) | 0.92 (0.06) | 0.00 (0.00) | 0.00 (0.00) | 0.00 (0.00) | 0.00 (0.00) |
|  | Donor administration costs | 55.41 (3.01) | 62.14 (3.82) | 68.10 (4.08) | 60.17 (4.18) | 47.01 (2.72) | 46.23 (2.31) | 49.59 (3.25) | 52.84 (2.82) |
|  | Energy | 94.51 (5.14) | 86.60 (5.32) | 72.40 (4.33) | 54.65 (3.79) | 81.36 (4.71) | 111.78 (5.60) | 60.45 (3.96) | 108.87 (5.82) |
|  | Debt relief | 8.94 (0.49) | 8.25 (0.51) | 141.16 (8.45) | 145.59 (10.10) | 143.61 (8.31) | 92.69 (4.64) | 84.95 (5.57) | 50.34 (2.69) |
|  | Multisector | 58.92 (3.20) | 57.01 (3.50) | 67.84 (4.06) | 47.71 (3.31) | 48.19 (2.79) | 60.26 (3.02) | 55.77 (3.66) | 60.35 (3.23) |
|  | Water and sanitation | 51.85 (2.82) | 48.11 (2.96) | 43.29 (2.59) | 38.16 (2.65) | 55.41 (3.21) | 78.49 (3.93) | 22.54 (1.48) | 75.09 (4.01) |
|  | Financial services and business support | 74.37 (4.04) | 72.67 (4.47) | 83.52 (5.00) | 79.43 (5.51) | 114.22 (6.61) | 88.24 (4.42) | 49.90 (3.27) | 82.55 (4.41) |
|  | Environmental protection | 59.93 (3.26) | 61.25 (3.76) | 39.50 (2.36) | 32.97 (2.29) | 31.08 (1.80) | 60.69 (3.04) | 38.17 (2.50) | 61.26 (3.27) |
|  | Other social services | 70.29 (3.82) | 61.62 (3.79) | 66.42 (3.98) | 40.76 (2.83) | 79.95 (4.62) | 99.63 (4.99) | 34.93 (2.29) | 72.16 (3.86) |
|  | Conflict, peace and security | 28.56 (1.55) | 16.48 (1.01) | 23.74 (1.42) | 23.24 (1.61) | 23.49 (1.36) | 22.04 (1.10) | 22.37 (1.47) | 29.76 (1.59) |
|  | General budget support | 36.28 (1.97) | 34.06 (2.09) | 32.84 (1.97) | 39.83 (2.76) | 35.97 (2.08) | 27.71 (1.39) | 28.16 (1.85) | 25.69 (1.37) |
|  | Unspecified | 19.49 (1.06) | 18.25 (1.12) | 22.54 (1.35) | 16.92 (1.17) | 43.48 (2.51) | 54.72 (2.74) | 79.20 (5.19) | 61.09 (3.27) |
|  | Industry, construction and mining | 50.50 (2.75) | 51.01 (3.14) | 20.15 (1.21) | 13.85 (0.96) | 30.19 (1.75) | 23.05 (1.15) | 27.99 (1.84) | 33.56 (1.79) |
|  | Food aid and commodity assistance | 13.17 (0.72) | 14.32 (0.88) | 10.65 (0.64) | 7.98 (0.55) | 12.84 (0.74) | 8.63 (0.43) | 8.08 (0.53) | 14.01 (0.75) |
|  | Trade policy | 13.02 (0.71) | 8.62 (0.53) | 11.80 (0.71) | 9.56 (0.66) | 11.83 (0.68) | 24.45 (1.22) | 7.73 (0.51) | 11.44 (0.61) |
|  | Tourism | 0.83 (0.05) | 0.76 (0.05) | 1.28 (0.08) | 0.64 (0.04) | 1.67 (0.10) | 1.41 (0.07) | 1.27 (0.08) | 2.10 (0.11) |
| Sweden | Health | 305.01 (18.66) | 259.15 (18.35) | 301.26 (19.10) | 260.40 (16.11) | 301.38 (12.96) | 285.47 (19.26) | 289.97 (16.61) | 294.33 (13.61) |
|  | Humanitarian aid | 170.54 (10.44) | 150.07 (10.63) | 148.98 (9.44) | 168.44 (10.42) | 282.55 (12.15) | 140.49 (9.48) | 314.63 (18.03) | 446.79 (20.66) |
|  | Government and civil society | 127.82 (7.82) | 99.85 (7.07) | 122.46 (7.76) | 121.91 (7.54) | 150.26 (6.46) | 96.25 (6.49) | 137.02 (7.85) | 167.90 (7.76) |
|  | Education | 96.22 (5.89) | 74.92 (5.31) | 87.33 (5.54) | 89.15 (5.51) | 114.82 (4.94) | 81.50 (5.50) | 102.71 (5.89) | 113.39 (5.24) |
|  | Infrastructure | 79.85 (4.89) | 84.91 (6.01) | 117.66 (7.46) | 116.28 (7.19) | 116.49 (5.01) | 99.55 (6.72) | 109.29 (6.26) | 135.16 (6.25) |
|  | Agriculture | 71.48 (4.37) | 56.65 (4.01) | 86.73 (5.50) | 102.94 (6.37) | 78.93 (3.39) | 102.49 (6.92) | 98.30 (5.63) | 97.89 (4.53) |
|  | Refugees in donor country | 0.00 (0.00) | 0.00 (0.00) | 0.33 (0.02) | 0.53 (0.03) | 0.00 (0.00) | 0.00 (0.00) | 0.00 (0.00) | 0.00 (0.00) |
|  | Donor administration costs | 65.71 (4.02) | 59.32 (4.20) | 59.77 (3.79) | 64.59 (3.99) | 71.26 (3.06) | 44.63 (3.01) | 50.15 (2.87) | 55.48 (2.57) |
|  | Energy | 57.02 (3.49) | 60.19 (4.26) | 69.59 (4.41) | 93.23 (5.77) | 81.98 (3.53) | 67.81 (4.58) | 94.05 (5.39) | 114.11 (5.28) |
|  | Debt relief | 156.63 (9.58) | 91.03 (6.45) | 9.29 (0.59) | 6.21 (0.38) | 20.36 (0.88) | 7.84 (0.53) | 6.00 (0.34) | 6.07 (0.28) |
|  | Multisector | 59.61 (3.65) | 52.75 (3.73) | 55.85 (3.54) | 52.80 (3.27) | 57.72 (2.48) | 68.77 (4.64) | 75.89 (4.35) | 71.93 (3.33) |
|  | Water and sanitation | 41.95 (2.57) | 36.65 (2.59) | 45.15 (2.86) | 65.31 (4.04) | 55.64 (2.39) | 59.40 (4.01) | 50.19 (2.88) | 74.03 (3.42) |
|  | Financial services and business support | 45.58 (2.79) | 42.74 (3.03) | 60.25 (3.82) | 61.12 (3.78) | 86.04 (3.70) | 44.97 (3.03) | 50.58 (2.90) | 60.46 (2.80) |
|  | Environmental protection | 40.52 (2.48) | 34.17 (2.42) | 29.75 (1.89) | 141.11 (8.73) | 29.87 (1.28) | 40.46 (2.73) | 41.05 (2.35) | 178.17 (8.24) |
|  | Other social services | 56.32 (3.45) | 48.65 (3.44) | 57.79 (3.66) | 48.00 (2.97) | 74.12 (3.19) | 68.50 (4.62) | 68.86 (3.95) | 59.96 (2.77) |
|  | Conflict, peace and security | 22.34 (1.37) | 15.80 (1.12) | 18.88 (1.20) | 20.39 (1.26) | 25.86 (1.11) | 15.96 (1.08) | 23.86 (1.37) | 43.46 (2.01) |
|  | General budget support | 21.27 (1.30) | 19.68 (1.39) | 19.98 (1.27) | 33.31 (2.06) | 25.15 (1.08) | 17.20 (1.16) | 19.96 (1.14) | 15.24 (0.70) |
|  | Unspecified | 9.81 (0.60) | 13.91 (0.98) | 16.63 (1.05) | 18.90 (1.17) | 32.65 (1.40) | 62.49 (4.22) | 41.36 (2.37) | 44.06 (2.04) |
|  | Industry, construction and mining | 29.41 (1.80) | 29.17 (2.07) | 16.48 (1.04) | 15.67 (0.97) | 22.29 (0.96) | 12.18 (0.82) | 27.35 (1.57) | 24.14 (1.12) |
|  | Food aid and commodity assistance | 11.28 (0.69) | 14.83 (1.05) | 7.28 (0.46) | 8.33 (0.52) | 11.48 (0.49) | 13.16 (0.89) | 7.20 (0.41) | 16.26 (0.75) |
|  | Trade policy | 7.49 (0.46) | 5.66 (0.40) | 9.35 (0.59) | 7.81 (0.48) | 8.70 (0.37) | 12.94 (0.87) | 6.62 (0.38) | 8.18 (0.38) |
|  | Tourism | 0.57 (0.04) | 0.53 (0.04) | 1.32 (0.08) | 1.02 (0.06) | 1.46 (0.06) | 0.73 (0.05) | 1.33 (0.08) | 1.59 (0.07) |
| Canada | Health | 256.94 (22.51) | 256.00 (18.95) | 221.07 (18.24) | 61.95 (7.25) | 425.82 (31.47) | 312.81 (23.10) | 347.65 (28.96) | 332.94 (29.02) |
|  | Humanitarian aid | 52.47 (4.60) | 33.31 (2.47) | 38.58 (3.18) | 32.14 (3.76) | 47.71 (3.53) | 47.06 (3.47) | 77.34 (6.44) | 81.45 (7.10) |
|  | Government and civil society | 89.76 (7.86) | 61.61 (4.56) | 108.76 (8.98) | 58.42 (6.84) | 92.46 (6.83) | 69.50 (5.13) | 66.97 (5.58) | 64.84 (5.65) |
|  | Education | 69.98 (6.13) | 63.65 (4.71) | 54.78 (4.52) | 59.41 (6.96) | 54.56 (4.03) | 68.47 (5.06) | 53.55 (4.46) | 52.90 (4.61) |
|  | Infrastructure | 93.86 (8.22) | 112.90 (8.36) | 108.82 (8.98) | 98.68 (11.56) | 90.73 (6.70) | 73.45 (5.42) | 80.75 (6.73) | 101.17 (8.82) |
|  | Agriculture | 82.19 (7.20) | 84.04 (6.22) | 88.10 (7.27) | 71.23 (8.34) | 69.61 (5.14) | 77.73 (5.74) | 70.38 (5.86) | 59.04 (5.15) |
|  | Refugees in donor country | 0.00 (0.00) | 0.00 (0.00) | 0.00 (0.00) | 0.00 (0.00) | 0.00 (0.00) | 0.00 (0.00) | 0.00 (0.00) | 0.00 (0.00) |
|  | Donor administration costs | 10.47 (0.92) | 18.90 (1.40) | 9.37 (0.77) | 11.77 (1.38) | 11.59 (0.86) | 20.11 (1.48) | 13.02 (1.08) | 12.66 (1.10) |
|  | Energy | 40.26 (3.53) | 67.55 (5.00) | 70.08 (5.78) | 82.97 (9.72) | 78.38 (5.79) | 53.87 (3.98) | 72.25 (6.02) | 62.05 (5.41) |
|  | Debt relief | 21.33 (1.87) | 64.24 (4.75) | 26.26 (2.17) | 0.44 (0.05) | 41.52 (3.07) | 3.23 (0.24) | 0.44 (0.04) | 0.70 (0.06) |
|  | Multisector | 25.68 (2.25) | 25.62 (1.90) | 36.45 (3.01) | 22.58 (2.64) | 29.98 (2.22) | 35.17 (2.60) | 31.42 (2.62) | 36.75 (3.20) |
|  | Water and sanitation | 42.88 (3.76) | 57.80 (4.28) | 53.52 (4.42) | 51.04 (5.98) | 50.90 (3.76) | 54.03 (3.99) | 39.62 (3.30) | 59.26 (5.17) |
|  | Financial services and business support | 24.39 (2.14) | 21.09 (1.56) | 35.51 (2.93) | 27.89 (3.27) | 34.35 (2.54) | 16.74 (1.24) | 26.24 (2.19) | 27.40 (2.39) |
|  | Environmental protection | 58.42 (5.12) | 43.38 (3.21) | 46.62 (3.85) | 24.39 (2.86) | 70.83 (5.23) | 51.77 (3.82) | 59.08 (4.92) | 21.20 (1.85) |
|  | Other social services | 38.28 (3.35) | 49.27 (3.65) | 45.50 (3.75) | 39.36 (4.61) | 43.35 (3.20) | 49.21 (3.63) | 53.37 (4.45) | 42.54 (3.71) |
|  | Conflict, peace and security | 3.32 (0.29) | 2.19 (0.16) | 6.26 (0.52) | 2.41 (0.28) | 4.08 (0.30) | 2.79 (0.21) | 3.49 (0.29) | 2.38 (0.21) |
|  | General budget support | 17.69 (1.55) | 12.59 (0.93) | 17.12 (1.41) | 9.97 (1.17) | 2.91 (0.21) | 6.18 (0.46) | 4.92 (0.41) | 0.94 (0.08) |
|  | Unspecified | 0.01 (0.00) | 1.91 (0.14) | 5.44 (0.45) | 3.90 (0.46) | 46.09 (3.41) | 66.51 (4.91) | 45.79 (3.81) | 59.68 (5.20) |
|  | Industry, construction and mining | 17.09 (1.50) | 13.77 (1.02) | 12.45 (1.03) | 9.30 (1.09) | 12.19 (0.90) | 8.08 (0.60) | 12.58 (1.05) | 9.75 (0.85) |
|  | Food aid and commodity assistance | 1.84 (0.16) | 2.86 (0.21) | 2.10 (0.17) | 2.45 (0.29) | 1.75 (0.13) | 1.96 (0.14) | 1.70 (0.14) | 2.13 (0.19) |
|  | Trade policy | 3.15 (0.28) | 5.46 (0.40) | 6.97 (0.57) | 3.45 (0.40) | 4.16 (0.31) | 11.62 (0.86) | 2.58 (0.21) | 4.59 (0.40) |
|  | Tourism | 0.56 (0.05) | 0.56 (0.04) | 1.10 (0.09) | 0.83 (0.10) | 1.52 (0.11) | 0.45 (0.03) | 0.66 (0.05) | 1.50 (0.13) |
| Norway | Health | 245.35 (26.66) | 260.16 (27.33) | 276.67 (28.66) | 298.48 (30.59) | 317.41 (30.44) | 302.71 (28.70) | 309.14 (28.80) | 289.99 (28.20) |
|  | Humanitarian aid | 65.08 (7.07) | 56.26 (5.91) | 55.61 (5.76) | 59.81 (6.13) | 75.90 (7.28) | 92.62 (8.78) | 159.17 (14.83) | 168.46 (16.38) |
|  | Government and civil society | 77.88 (8.46) | 68.41 (7.19) | 69.27 (7.18) | 74.93 (7.68) | 68.71 (6.59) | 61.23 (5.81) | 63.64 (5.93) | 59.64 (5.80) |
|  | Education | 40.72 (4.42) | 36.19 (3.80) | 35.44 (3.67) | 36.00 (3.69) | 36.88 (3.54) | 38.06 (3.61) | 31.50 (2.93) | 33.53 (3.26) |
|  | Infrastructure | 19.47 (2.12) | 42.20 (4.43) | 42.37 (4.39) | 46.87 (4.80) | 44.07 (4.23) | 38.34 (3.63) | 37.07 (3.45) | 52.93 (5.15) |
|  | Agriculture | 24.45 (2.66) | 30.40 (3.19) | 32.93 (3.41) | 30.84 (3.16) | 26.92 (2.58) | 31.10 (2.95) | 28.36 (2.64) | 26.92 (2.62) |
|  | Refugees in donor country | 0.00 (0.00) | 0.00 (0.00) | 0.00 (0.00) | 0.00 (0.00) | 0.00 (0.00) | 0.00 (0.00) | 0.00 (0.00) | 0.00 (0.00) |
|  | Donor administration costs | 35.77 (3.89) | 37.32 (3.92) | 36.18 (3.75) | 36.38 (3.73) | 32.27 (3.10) | 31.93 (3.03) | 28.56 (2.66) | 27.60 (2.68) |
|  | Energy | 13.97 (1.52) | 23.64 (2.48) | 29.26 (3.03) | 32.63 (3.34) | 35.39 (3.39) | 24.25 (2.30) | 31.33 (2.92) | 27.36 (2.66) |
|  | Debt relief | 67.39 (7.32) | 19.42 (2.04) | 5.27 (0.55) | 4.42 (0.45) | 10.72 (1.03) | 6.24 (0.59) | 4.88 (0.45) | 4.51 (0.44) |
|  | Multisector | 45.73 (4.97) | 37.43 (3.93) | 35.82 (3.71) | 34.94 (3.58) | 32.64 (3.13) | 34.59 (3.28) | 32.05 (2.99) | 31.29 (3.04) |
|  | Water and sanitation | 13.93 (1.51) | 20.60 (2.16) | 21.81 (2.26) | 26.01 (2.67) | 24.86 (2.38) | 26.13 (2.48) | 17.50 (1.63) | 29.26 (2.85) |
|  | Financial services and business support | 10.20 (1.11) | 8.24 (0.87) | 13.92 (1.44) | 10.68 (1.09) | 12.90 (1.24) | 5.60 (0.53) | 9.07 (0.85) | 9.78 (0.95) |
|  | Environmental protection | 30.57 (3.32) | 27.80 (2.92) | 25.26 (2.62) | 28.24 (2.89) | 35.47 (3.40) | 28.48 (2.70) | 22.19 (2.07) | 21.85 (2.12) |
|  | Other social services | 27.19 (2.95) | 26.01 (2.73) | 28.17 (2.92) | 20.03 (2.05) | 37.53 (3.60) | 33.06 (3.13) | 33.21 (3.09) | 24.91 (2.42) |
|  | Conflict, peace and security | 7.87 (0.86) | 8.09 (0.85) | 8.33 (0.86) | 8.57 (0.88) | 7.28 (0.70) | 3.61 (0.34) | 11.75 (1.09) | 11.68 (1.14) |
|  | General budget support | 0.06 (0.01) | 9.14 (0.96) | 12.38 (1.28) | 8.52 (0.87) | 2.57 (0.25) | 6.81 (0.65) | 4.89 (0.46) | 2.15 (0.21) |
|  | Unspecified | 0.00 (0.00) | 9.70 (1.02) | 10.80 (1.12) | 11.45 (1.17) | 31.58 (3.03) | 44.51 (4.22) | 43.91 (4.09) | 39.38 (3.83) |
|  | Industry, construction and mining | 5.73 (0.62) | 4.18 (0.44) | 4.24 (0.44) | 3.74 (0.38) | 4.15 (0.40) | 2.88 (0.27) | 4.52 (0.42) | 3.44 (0.33) |
|  | Food aid and commodity assistance | 1.15 (0.12) | 2.17 (0.23) | 1.03 (0.11) | 1.70 (0.17) | 1.38 (0.13) | 2.47 (0.23) | 1.33 (0.12) | 2.39 (0.23) |
|  | Trade policy | 1.51 (0.16) | 1.79 (0.19) | 2.36 (0.24) | 1.12 (0.11) | 1.36 (0.13) | 3.86 (0.37) | 0.86 (0.08) | 1.56 (0.15) |
|  | Tourism | 0.27 (0.03) | 0.27 (0.03) | 0.45 (0.05) | 0.47 (0.05) | 0.56 (0.05) | 0.12 (0.01) | 0.21 (0.02) | 0.51 (0.05) |
| Spain | Health | 98.65 (5.89) | 36.02 (3.60) | 60.05 (4.67) | 49.74 (3.83) | 49.69 (4.36) | 91.22 (4.96) | 108.32 (5.45) | 75.21 (4.00) |
|  | Humanitarian aid | 133.62 (7.98) | 83.33 (8.33) | 104.28 (8.11) | 117.53 (9.05) | 112.23 (9.84) | 172.08 (9.36) | 151.15 (7.61) | 149.47 (7.94) |
|  | Government and civil society | 137.40 (8.20) | 79.68 (7.97) | 97.13 (7.55) | 92.99 (7.16) | 98.90 (8.67) | 154.54 (8.41) | 181.05 (9.12) | 218.50 (11.61) |
|  | Education | 100.58 (6.01) | 46.48 (4.65) | 53.13 (4.13) | 47.82 (3.68) | 52.86 (4.64) | 114.95 (6.25) | 109.94 (5.53) | 114.76 (6.10) |
|  | Infrastructure | 162.68 (9.71) | 127.72 (12.77) | 151.56 (11.78) | 144.79 (11.14) | 112.57 (9.87) | 208.83 (11.36) | 179.14 (9.02) | 238.72 (12.68) |
|  | Agriculture | 103.00 (6.15) | 35.64 (3.56) | 67.87 (5.27) | 83.66 (6.44) | 71.93 (6.31) | 172.91 (9.41) | 139.70 (7.03) | 140.67 (7.47) |
|  | Refugees in donor country | 0.00 (0.00) | 0.00 (0.00) | 1.01 (0.08) | 1.44 (0.11) | 0.00 (0.00) | 0.00 (0.00) | 0.00 (0.00) | 0.00 (0.00) |
|  | Donor administration costs | 60.59 (3.62) | 38.26 (3.83) | 48.20 (3.75) | 44.47 (3.42) | 44.59 (3.91) | 54.79 (2.98) | 50.91 (2.56) | 54.38 (2.89) |
|  | Energy | 118.62 (7.08) | 83.65 (8.36) | 76.16 (5.92) | 86.75 (6.68) | 60.00 (5.26) | 149.17 (8.11) | 147.93 (7.45) | 141.47 (7.52) |
|  | Debt relief | 37.36 (2.23) | 3.21 (0.32) | 0.52 (0.04) | 0.15 (0.01) | 1.07 (0.09) | 0.13 (0.01) | 0.22 (0.01) | 0.18 (0.01) |
|  | Multisector | 58.49 (3.49) | 38.63 (3.86) | 51.17 (3.98) | 53.05 (4.08) | 42.46 (3.72) | 82.34 (4.48) | 89.71 (4.52) | 89.79 (4.77) |
|  | Water and sanitation | 57.53 (3.44) | 32.39 (3.24) | 35.13 (2.73) | 58.88 (4.53) | 42.37 (3.72) | 83.82 (4.56) | 61.29 (3.09) | 93.45 (4.96) |
|  | Financial services and business support | 99.65 (5.95) | 95.58 (9.56) | 105.45 (8.20) | 121.26 (9.33) | 140.05 (12.28) | 155.03 (8.43) | 112.72 (5.67) | 127.08 (6.75) |
|  | Environmental protection | 19.96 (1.19) | 20.40 (2.04) | 22.19 (1.73) | 32.47 (2.50) | 28.50 (2.50) | 53.15 (2.89) | 47.09 (2.37) | 41.07 (2.18) |
|  | Other social services | 60.23 (3.60) | 34.46 (3.44) | 50.53 (3.93) | 45.89 (3.53) | 53.77 (4.72) | 80.64 (4.39) | 71.58 (3.60) | 61.06 (3.24) |
|  | Conflict, peace and security | 40.05 (2.39) | 18.47 (1.85) | 32.36 (2.51) | 33.41 (2.57) | 36.27 (3.18) | 43.13 (2.35) | 44.15 (2.22) | 53.90 (2.86) |
|  | General budget support | 62.31 (3.72) | 52.32 (5.23) | 51.43 (4.00) | 72.46 (5.58) | 52.10 (4.57) | 47.51 (2.58) | 42.62 (2.15) | 37.29 (1.98) |
|  | Unspecified | 31.49 (1.88) | 13.22 (1.32) | 12.03 (0.93) | 6.52 (0.50) | 12.58 (1.10) | 10.96 (0.60) | 11.71 (0.59) | 8.59 (0.46) |
|  | Industry, construction and mining | 67.44 (4.03) | 68.16 (6.81) | 22.84 (1.77) | 20.33 (1.56) | 30.96 (2.72) | 29.79 (1.62) | 61.51 (3.10) | 53.14 (2.82) |
|  | Food aid and commodity assistance | 17.76 (1.06) | 14.50 (1.45) | 13.38 (1.04) | 7.70 (0.59) | 15.86 (1.39) | 14.82 (0.81) | 13.81 (0.70) | 22.10 (1.17) |
|  | Trade policy | 17.42 (1.04) | 8.82 (0.88) | 13.34 (1.04) | 13.79 (1.06) | 12.70 (1.11) | 21.74 (1.18) | 15.99 (0.80) | 15.85 (0.84) |
|  | Tourism | 1.14 (0.07) | 0.72 (0.07) | 1.36 (0.11) | 0.87 (0.07) | 0.77 (0.07) | 5.43 (0.30) | 5.78 (0.29) | 4.74 (0.25) |
| Italy | Health | 99.48 (4.16) | 90.86 (4.43) | 115.50 (4.85) | 142.89 (5.88) | 144.24 (6.07) | 179.13 (6.20) | 158.49 (5.21) | 189.78 (6.41) |
|  | Humanitarian aid | 197.30 (8.25) | 149.63 (7.30) | 185.49 (7.78) | 208.91 (8.59) | 195.11 (8.21) | 276.26 (9.55) | 250.58 (8.24) | 245.62 (8.30) |
|  | Government and civil society | 180.82 (7.56) | 161.99 (7.90) | 198.73 (8.34) | 199.03 (8.18) | 197.04 (8.29) | 215.77 (7.46) | 268.88 (8.84) | 330.84 (11.18) |
|  | Education | 132.10 (5.53) | 106.04 (5.17) | 126.61 (5.31) | 125.41 (5.16) | 118.56 (4.99) | 152.34 (5.27) | 159.61 (5.25) | 164.62 (5.56) |
|  | Infrastructure | 280.44 (11.73) | 265.53 (12.95) | 333.27 (13.98) | 263.95 (10.85) | 236.41 (9.95) | 280.98 (9.72) | 284.48 (9.35) | 365.88 (12.37) |
|  | Agriculture | 164.49 (6.88) | 97.57 (4.76) | 160.85 (6.75) | 179.23 (7.37) | 153.27 (6.45) | 233.57 (8.08) | 201.29 (6.62) | 217.68 (7.36) |
|  | Refugees in donor country | 0.00 (0.00) | 0.00 (0.00) | 1.59 (0.07) | 2.33 (0.10) | 0.00 (0.00) | 0.00 (0.00) | 0.00 (0.00) | 0.00 (0.00) |
|  | Donor administration costs | 79.08 (3.31) | 67.00 (3.27) | 75.48 (3.17) | 75.95 (3.12) | 72.03 (3.03) | 76.33 (2.64) | 80.80 (2.66) | 83.33 (2.82) |
|  | Energy | 192.38 (8.05) | 167.18 (8.15) | 177.11 (7.43) | 189.27 (7.78) | 142.07 (5.98) | 201.11 (6.96) | 228.14 (7.50) | 213.40 (7.21) |
|  | Debt relief | 18.12 (0.76) | 25.24 (1.23) | 14.04 (0.59) | 10.69 (0.44) | 23.01 (0.97) | 10.50 (0.36) | 10.49 (0.34) | 8.80 (0.30) |
|  | Multisector | 77.64 (3.25) | 77.24 (3.77) | 91.05 (3.82) | 92.21 (3.79) | 84.04 (3.54) | 117.89 (4.08) | 155.93 (5.12) | 148.30 (5.01) |
|  | Water and sanitation | 93.19 (3.90) | 79.48 (3.88) | 99.35 (4.17) | 115.06 (4.73) | 98.13 (4.13) | 102.85 (3.56) | 92.75 (3.05) | 144.50 (4.88) |
|  | Financial services and business support | 160.13 (6.70) | 165.59 (8.07) | 190.53 (7.99) | 216.83 (8.92) | 241.02 (10.14) | 227.00 (7.85) | 161.47 (5.31) | 188.41 (6.37) |
|  | Environmental protection | 47.38 (1.98) | 41.93 (2.04) | 52.91 (2.22) | 55.90 (2.30) | 53.61 (2.26) | 97.77 (3.38) | 98.93 (3.25) | 57.58 (1.95) |
|  | Other social services | 78.24 (3.27) | 87.03 (4.24) | 109.45 (4.59) | 114.20 (4.70) | 110.16 (4.64) | 110.07 (3.81) | 89.77 (2.95) | 86.78 (2.93) |
|  | Conflict, peace and security | 65.38 (2.73) | 31.76 (1.55) | 47.54 (1.99) | 56.08 (2.31) | 58.29 (2.45) | 66.37 (2.30) | 67.45 (2.22) | 80.43 (2.72) |
|  | General budget support | 125.40 (5.25) | 90.12 (4.39) | 105.71 (4.43) | 110.57 (4.55) | 84.07 (3.54) | 72.00 (2.49) | 70.57 (2.32) | 56.08 (1.90) |
|  | Unspecified | 54.44 (2.28) | 21.62 (1.05) | 20.91 (0.88) | 12.43 (0.51) | 59.01 (2.48) | 31.28 (1.08) | 110.81 (3.64) | 23.09 (0.78) |
|  | Industry, construction and mining | 108.08 (4.52) | 116.76 (5.69) | 43.83 (1.84) | 39.94 (1.64) | 55.68 (2.34) | 41.74 (1.44) | 88.29 (2.90) | 78.68 (2.66) |
|  | Food aid and commodity assistance | 29.25 (1.22) | 25.27 (1.23) | 22.61 (0.95) | 14.03 (0.58) | 25.99 (1.09) | 23.30 (0.81) | 20.62 (0.68) | 34.85 (1.18) |
|  | Trade policy | 28.13 (1.18) | 16.90 (0.82) | 25.51 (1.07) | 25.57 (1.05) | 22.44 (0.94) | 27.71 (0.96) | 22.99 (0.76) | 23.15 (0.78) |
|  | Tourism | 1.80 (0.08) | 1.41 (0.07) | 2.77 (0.12) | 1.86 (0.08) | 2.01 (0.08) | 3.87 (0.13) | 4.54 (0.15) | 2.83 (0.10) |
| Australia | Health | 131.87 (24.12) | 171.82 (25.59) | 174.81 (30.45) | 158.41 (20.37) | 113.88 (14.44) | 170.18 (16.22) | 69.96 (11.26) | 40.71 (6.80) |
|  | Humanitarian aid | 38.45 (7.03) | 46.68 (6.95) | 32.89 (5.73) | 43.56 (5.60) | 54.70 (6.94) | 52.74 (5.03) | 39.02 (6.28) | 41.07 (6.86) |
|  | Government and civil society | 44.80 (8.20) | 39.41 (5.87) | 45.35 (7.90) | 44.98 (5.78) | 40.31 (5.11) | 50.66 (4.83) | 38.86 (6.25) | 32.41 (5.41) |
|  | Education | 41.30 (7.55) | 46.38 (6.91) | 38.48 (6.70) | 34.35 (4.42) | 34.22 (4.34) | 48.59 (4.63) | 26.48 (4.26) | 29.53 (4.93) |
|  | Infrastructure | 39.39 (7.21) | 56.50 (8.41) | 47.62 (8.29) | 47.13 (6.06) | 46.59 (5.91) | 55.17 (5.26) | 43.28 (6.96) | 135.08 (22.56) |
|  | Agriculture | 34.67 (6.34) | 38.94 (5.80) | 35.23 (6.14) | 41.92 (5.39) | 32.82 (4.16) | 49.45 (4.71) | 28.94 (4.66) | 31.96 (5.34) |
|  | Refugees in donor country | 0.00 (0.00) | 0.00 (0.00) | 0.00 (0.00) | 0.00 (0.00) | 0.00 (0.00) | 0.00 (0.00) | 0.00 (0.00) | 0.00 (0.00) |
|  | Donor administration costs | 18.11 (3.31) | 20.15 (3.00) | 5.04 (0.88) | 22.62 (2.91) | 6.08 (0.77) | 8.63 (0.82) | 5.85 (0.94) | 4.67 (0.78) |
|  | Energy | 17.37 (3.18) | 29.76 (4.43) | 27.97 (4.87) | 52.00 (6.69) | 41.82 (5.30) | 44.57 (4.25) | 31.27 (5.03) | 39.62 (6.62) |
|  | Debt relief | 0.04 (0.01) | 0.00 (0.00) | 1.56 (0.27) | 0.35 (0.05) | 7.95 (1.01) | 0.32 (0.03) | 0.07 (0.01) | 0.29 (0.05) |
|  | Multisector | 19.37 (3.54) | 21.49 (3.20) | 10.53 (1.83) | 22.16 (2.85) | 19.03 (2.41) | 17.23 (1.64) | 10.92 (1.76) | 11.60 (1.94) |
|  | Water and sanitation | 19.31 (3.53) | 26.15 (3.90) | 23.01 (4.01) | 32.06 (4.12) | 26.57 (3.37) | 39.52 (3.77) | 22.66 (3.65) | 66.27 (11.07) |
|  | Financial services and business support | 12.48 (2.28) | 11.41 (1.70) | 15.38 (2.68) | 20.62 (2.65) | 19.50 (2.47) | 21.52 (2.05) | 16.64 (2.68) | 12.16 (2.03) |
|  | Environmental protection | 12.32 (2.25) | 19.41 (2.89) | 23.32 (4.06) | 73.30 (9.43) | 29.79 (3.78) | 24.73 (2.36) | 17.33 (2.79) | 14.85 (2.48) |
|  | Other social services | 20.88 (3.82) | 25.49 (3.80) | 19.12 (3.33) | 26.52 (3.41) | 21.69 (2.75) | 38.67 (3.68) | 21.97 (3.54) | 21.59 (3.61) |
|  | Conflict, peace and security | 1.57 (0.29) | 1.79 (0.27) | 0.58 (0.10) | 2.66 (0.34) | 1.16 (0.15) | 0.66 (0.06) | 2.06 (0.33) | 3.11 (0.52) |
|  | General budget support | 0.08 (0.01) | 0.11 (0.02) | 0.10 (0.02) | 30.53 (3.93) | 0.15 (0.02) | 0.61 (0.06) | 0.23 (0.04) | 0.30 (0.05) |
|  | Unspecified | 0.00 (0.00) | 3.95 (0.59) | 2.45 (0.43) | 10.84 (1.39) | 7.41 (0.94) | 13.27 (1.26) | 8.42 (1.35) | 9.57 (1.60) |
|  | Industry, construction and mining | 9.46 (1.73) | 7.12 (1.06) | 5.85 (1.02) | 5.69 (0.73) | 6.29 (0.80) | 5.50 (0.52) | 7.68 (1.24) | 5.16 (0.86) |
|  | Food aid and commodity assistance | 0.65 (0.12) | 4.02 (0.60) | 1.60 (0.28) | 2.53 (0.33) | 2.62 (0.33) | 2.75 (0.26) | 0.33 (0.05) | 0.40 (0.07) |
|  | Trade policy | 1.61 (0.29) | 2.72 (0.41) | 2.58 (0.45) | 1.36 (0.17) | 2.08 (0.26) | 7.26 (0.69) | 1.64 (0.26) | 2.46 (0.41) |
|  | Tourism | 0.32 (0.06) | 0.32 (0.05) | 0.69 (0.12) | 0.89 (0.11) | 0.84 (0.11) | 0.56 (0.05) | 0.16 (0.03) | 0.58 (0.10) |
| Switzerland | Health | 73.75 (11.82) | 72.48 (12.91) | 62.25 (9.67) | 78.88 (11.52) | 85.49 (11.15) | 80.77 (9.94) | 83.82 (10.28) | 77.98 (10.19) |
|  | Humanitarian aid | 32.53 (5.21) | 23.38 (4.16) | 25.67 (3.99) | 31.11 (4.54) | 39.73 (5.18) | 43.54 (5.36) | 55.25 (6.77) | 61.75 (8.07) |
|  | Government and civil society | 75.04 (12.03) | 51.69 (9.20) | 65.10 (10.11) | 66.56 (9.72) | 71.72 (9.35) | 66.10 (8.13) | 64.75 (7.94) | 61.37 (8.02) |
|  | Education | 51.31 (8.22) | 32.65 (5.81) | 37.49 (5.82) | 45.53 (6.65) | 49.74 (6.49) | 55.71 (6.85) | 45.43 (5.57) | 47.93 (6.26) |
|  | Infrastructure | 52.67 (8.44) | 48.86 (8.70) | 58.48 (9.08) | 56.49 (8.25) | 59.26 (7.73) | 52.81 (6.50) | 58.96 (7.23) | 75.38 (9.85) |
|  | Agriculture | 50.70 (8.13) | 38.97 (6.94) | 59.73 (9.28) | 45.43 (6.63) | 46.71 (6.09) | 56.29 (6.92) | 52.57 (6.45) | 47.98 (6.27) |
|  | Refugees in donor country | 0.00 (0.00) | 0.00 (0.00) | 0.00 (0.00) | 0.00 (0.00) | 0.00 (0.00) | 0.00 (0.00) | 0.00 (0.00) | 0.00 (0.00) |
|  | Donor administration costs | 12.41 (1.99) | 11.43 (2.04) | 11.53 (1.79) | 14.32 (2.09) | 12.26 (1.60) | 9.90 (1.22) | 9.99 (1.22) | 10.28 (1.34) |
|  | Energy | 27.06 (4.34) | 30.17 (5.37) | 39.43 (6.12) | 47.04 (6.87) | 50.05 (6.53) | 38.55 (4.74) | 53.20 (6.52) | 48.10 (6.28) |
|  | Debt relief | 5.52 (0.88) | 15.58 (2.77) | 7.85 (1.22) | 21.58 (3.15) | 18.47 (2.41) | 15.22 (1.87) | 5.39 (0.66) | 5.21 (0.68) |
|  | Multisector | 23.65 (3.79) | 22.19 (3.95) | 25.28 (3.93) | 22.94 (3.35) | 24.82 (3.24) | 29.59 (3.64) | 29.07 (3.56) | 32.41 (4.23) |
|  | Water and sanitation | 24.56 (3.94) | 25.80 (4.59) | 29.71 (4.62) | 31.04 (4.53) | 32.94 (4.30) | 38.69 (4.76) | 29.36 (3.60) | 44.90 (5.87) |
|  | Financial services and business support | 16.15 (2.59) | 9.57 (1.70) | 20.00 (3.11) | 15.25 (2.23) | 23.29 (3.04) | 10.57 (1.30) | 18.12 (2.22) | 20.30 (2.65) |
|  | Environmental protection | 32.56 (5.22) | 32.04 (5.71) | 31.59 (4.91) | 32.94 (4.81) | 40.73 (5.31) | 41.01 (5.05) | 37.67 (4.62) | 30.81 (4.02) |
|  | Other social services | 27.93 (4.48) | 22.34 (3.98) | 29.56 (4.59) | 24.31 (3.55) | 32.91 (4.29) | 37.88 (4.66) | 40.19 (4.93) | 32.46 (4.24) |
|  | Conflict, peace and security | 4.33 (0.69) | 4.00 (0.71) | 4.53 (0.70) | 5.25 (0.77) | 4.40 (0.57) | 2.86 (0.35) | 3.94 (0.48) | 4.24 (0.55) |
|  | General budget support | 14.14 (2.27) | 9.85 (1.75) | 14.15 (2.20) | 21.16 (3.09) | 11.68 (1.52) | 16.23 (2.00) | 15.42 (1.89) | 11.71 (1.53) |
|  | Unspecified | 0.00 (0.00) | 3.71 (0.66) | 4.69 (0.73) | 5.40 (0.79) | 21.82 (2.85) | 36.04 (4.43) | 37.30 (4.57) | 34.35 (4.49) |
|  | Industry, construction and mining | 10.72 (1.72) | 6.00 (1.07) | 7.01 (1.09) | 5.70 (0.83) | 8.45 (1.10) | 5.75 (0.71) | 9.06 (1.11) | 7.22 (0.94) |
|  | Food aid and commodity assistance | 0.91 (0.15) | 0.83 (0.15) | 1.05 (0.16) | 1.11 (0.16) | 0.75 (0.10) | 0.85 (0.10) | 1.08 (0.13) | 1.22 (0.16) |
|  | Trade policy | 2.37 (0.38) | 2.50 (0.44) | 4.14 (0.64) | 2.10 (0.31) | 3.01 (0.39) | 8.43 (1.04) | 1.82 (0.22) | 3.43 (0.45) |
|  | Tourism | 0.25 (0.04) | 0.24 (0.04) | 0.57 (0.09) | 0.54 (0.08) | 1.00 (0.13) | 0.42 (0.05) | 0.68 (0.08) | 1.34 (0.17) |
| Denmark | Health | 111.29 (15.53) | 110.07 (14.74) | 104.68 (14.28) | 124.63 (15.52) | 71.55 (9.58) | 69.35 (8.68) | 64.17 (8.35) | 76.19 (9.76) |
|  | Humanitarian aid | 76.08 (10.62) | 70.17 (9.40) | 74.56 (10.17) | 103.05 (12.83) | 90.64 (12.13) | 124.87 (15.62) | 142.88 (18.59) | 130.50 (16.73) |
|  | Government and civil society | 67.00 (9.35) | 65.85 (8.82) | 61.58 (8.40) | 66.39 (8.27) | 69.96 (9.36) | 62.06 (7.76) | 70.69 (9.20) | 74.96 (9.61) |
|  | Education | 40.16 (5.60) | 39.66 (5.31) | 37.10 (5.06) | 36.76 (4.58) | 39.85 (5.33) | 45.14 (5.65) | 41.46 (5.39) | 39.20 (5.02) |
|  | Infrastructure | 49.77 (6.94) | 60.40 (8.09) | 63.38 (8.65) | 45.34 (5.65) | 54.05 (7.24) | 64.12 (8.02) | 53.88 (7.01) | 62.58 (8.02) |
|  | Agriculture | 35.30 (4.93) | 31.40 (4.20) | 35.59 (4.86) | 34.58 (4.31) | 38.21 (5.11) | 54.38 (6.80) | 42.87 (5.58) | 40.73 (5.22) |
|  | Refugees in donor country | 0.00 (0.00) | 0.00 (0.00) | 0.26 (0.04) | 0.38 (0.05) | 0.00 (0.00) | 0.00 (0.00) | 0.00 (0.00) | 0.00 (0.00) |
|  | Donor administration costs | 27.86 (3.89) | 29.54 (3.96) | 28.23 (3.85) | 42.04 (5.24) | 21.28 (2.85) | 16.53 (2.07) | 16.14 (2.10) | 16.88 (2.16) |
|  | Energy | 34.79 (4.85) | 39.83 (5.33) | 38.05 (5.19) | 35.56 (4.43) | 37.88 (5.07) | 43.98 (5.50) | 47.98 (6.24) | 48.31 (6.19) |
|  | Debt relief | 14.14 (1.97) | 23.21 (3.11) | 15.97 (2.18) | 0.20 (0.02) | 7.14 (0.96) | 0.19 (0.02) | 0.16 (0.02) | 0.15 (0.02) |
|  | Multisector | 39.10 (5.46) | 32.76 (4.39) | 34.57 (4.72) | 35.04 (4.36) | 26.51 (3.55) | 35.54 (4.45) | 29.31 (3.81) | 27.53 (3.53) |
|  | Water and sanitation | 20.08 (2.80) | 23.28 (3.12) | 22.94 (3.13) | 23.50 (2.93) | 25.62 (3.43) | 30.55 (3.82) | 20.60 (2.68) | 27.77 (3.56) |
|  | Financial services and business support | 27.15 (3.79) | 30.71 (4.11) | 34.68 (4.73) | 37.50 (4.67) | 46.79 (6.26) | 35.98 (4.50) | 30.20 (3.93) | 33.52 (4.30) |
|  | Environmental protection | 25.57 (3.57) | 27.22 (3.65) | 32.42 (4.42) | 28.84 (3.59) | 30.65 (4.10) | 36.81 (4.61) | 33.59 (4.37) | 63.01 (8.08) |
|  | Other social services | 23.06 (3.22) | 26.17 (3.50) | 25.06 (3.42) | 26.27 (3.27) | 34.14 (4.57) | 31.26 (3.91) | 30.49 (3.97) | 24.09 (3.09) |
|  | Conflict, peace and security | 12.82 (1.79) | 12.23 (1.64) | 11.46 (1.56) | 13.02 (1.62) | 12.54 (1.68) | 10.57 (1.32) | 12.11 (1.58) | 18.40 (2.36) |
|  | General budget support | 19.64 (2.74) | 17.88 (2.39) | 29.47 (4.02) | 37.38 (4.66) | 14.61 (1.96) | 15.04 (1.88) | 11.70 (1.52) | 9.61 (1.23) |
|  | Unspecified | 7.70 (1.07) | 8.09 (1.08) | 9.81 (1.34) | 12.92 (1.61) | 19.37 (2.59) | 15.83 (1.98) | 13.98 (1.82) | 10.98 (1.41) |
|  | Industry, construction and mining | 17.66 (2.46) | 20.82 (2.79) | 8.30 (1.13) | 7.30 (0.91) | 11.43 (1.53) | 7.86 (0.98) | 16.40 (2.13) | 13.76 (1.76) |
|  | Food aid and commodity assistance | 5.40 (0.75) | 6.98 (0.93) | 4.51 (0.62) | 4.52 (0.56) | 6.39 (0.86) | 5.88 (0.74) | 4.31 (0.56) | 7.33 (0.94) |
|  | Trade policy | 4.70 (0.66) | 3.63 (0.49) | 4.56 (0.62) | 4.31 (0.54) | 4.42 (0.59) | 6.39 (0.80) | 4.15 (0.54) | 4.23 (0.54) |
|  | Tourism | 0.38 (0.05) | 0.38 (0.05) | 0.59 (0.08) | 0.48 (0.06) | 0.63 (0.08) | 0.55 (0.07) | 0.77 (0.10) | 0.64 (0.08) |
| Belgium | Health | 94.84 (9.43) | 83.06 (9.42) | 75.27 (7.94) | 85.96 (8.05) | 75.82 (8.56) | 76.65 (7.95) | 83.35 (8.70) | 72.20 (7.13) |
|  | Humanitarian aid | 69.46 (6.90) | 57.88 (6.56) | 64.45 (6.80) | 84.78 (7.94) | 73.47 (8.29) | 95.72 (9.92) | 109.93 (11.48) | 97.06 (9.59) |
|  | Government and civil society | 85.26 (8.47) | 74.08 (8.40) | 75.84 (8.00) | 83.74 (7.84) | 69.61 (7.86) | 72.95 (7.56) | 96.53 (10.08) | 118.64 (11.72) |
|  | Education | 56.30 (5.60) | 48.78 (5.53) | 49.82 (5.25) | 52.33 (4.90) | 36.96 (4.17) | 47.32 (4.91) | 54.85 (5.73) | 63.00 (6.22) |
|  | Infrastructure | 103.93 (10.33) | 93.59 (10.61) | 114.10 (12.03) | 95.23 (8.92) | 75.43 (8.51) | 89.22 (9.25) | 83.29 (8.70) | 97.11 (9.59) |
|  | Agriculture | 71.99 (7.16) | 48.45 (5.49) | 66.36 (7.00) | 69.80 (6.54) | 46.44 (5.24) | 75.01 (7.78) | 60.33 (6.30) | 73.16 (7.23) |
|  | Refugees in donor country | 0.00 (0.00) | 0.00 (0.00) | 0.50 (0.05) | 0.74 (0.07) | 0.00 (0.00) | 0.00 (0.00) | 0.00 (0.00) | 0.00 (0.00) |
|  | Donor administration costs | 30.84 (3.07) | 30.69 (3.48) | 29.49 (3.11) | 32.20 (3.02) | 30.37 (3.43) | 29.27 (3.03) | 33.45 (3.49) | 32.33 (3.19) |
|  | Energy | 67.29 (6.69) | 65.64 (7.44) | 65.05 (6.86) | 69.70 (6.53) | 43.83 (4.95) | 62.37 (6.47) | 70.81 (7.39) | 74.82 (7.39) |
|  | Debt relief | 2.11 (0.21) | 5.23 (0.59) | 4.51 (0.48) | 2.96 (0.28) | 7.16 (0.81) | 2.73 (0.28) | 2.36 (0.25) | 2.35 (0.23) |
|  | Multisector | 36.41 (3.62) | 35.39 (4.01) | 37.15 (3.92) | 38.28 (3.59) | 33.97 (3.83) | 44.93 (4.66) | 51.61 (5.39) | 48.04 (4.75) |
|  | Water and sanitation | 38.58 (3.84) | 36.36 (4.12) | 38.91 (4.10) | 44.41 (4.16) | 31.74 (3.58) | 33.78 (3.50) | 25.99 (2.71) | 40.60 (4.01) |
|  | Financial services and business support | 51.28 (5.10) | 54.40 (6.17) | 64.37 (6.79) | 70.92 (6.64) | 75.60 (8.53) | 70.74 (7.33) | 54.79 (5.72) | 64.64 (6.39) |
|  | Environmental protection | 49.58 (4.93) | 45.93 (5.21) | 52.09 (5.49) | 42.35 (3.97) | 34.52 (3.90) | 57.64 (5.98) | 41.95 (4.38) | 32.84 (3.24) |
|  | Other social services | 36.05 (3.58) | 33.78 (3.83) | 42.71 (4.50) | 41.54 (3.89) | 37.90 (4.28) | 33.95 (3.52) | 31.70 (3.31) | 40.41 (3.99) |
|  | Conflict, peace and security | 21.42 (2.13) | 11.47 (1.30) | 16.87 (1.78) | 19.42 (1.82) | 20.68 (2.33) | 21.51 (2.23) | 24.44 (2.55) | 28.67 (2.83) |
|  | General budget support | 44.20 (4.39) | 26.40 (2.99) | 33.75 (3.56) | 51.56 (4.83) | 65.77 (7.42) | 24.87 (2.58) | 23.55 (2.46) | 18.38 (1.82) |
|  | Unspecified | 15.33 (1.52) | 10.15 (1.15) | 8.00 (0.84) | 7.92 (0.74) | 21.45 (2.42) | 14.00 (1.45) | 10.75 (1.12) | 10.19 (1.01) |
|  | Industry, construction and mining | 34.25 (3.40) | 38.76 (4.39) | 15.86 (1.67) | 14.01 (1.31) | 16.85 (1.90) | 12.51 (1.30) | 30.26 (3.16) | 26.82 (2.65) |
|  | Food aid and commodity assistance | 8.44 (0.84) | 7.47 (0.85) | 7.24 (0.76) | 5.51 (0.52) | 8.30 (0.94) | 8.18 (0.85) | 7.17 (0.75) | 11.12 (1.10) |
|  | Trade policy | 8.88 (0.88) | 6.46 (0.73) | 9.12 (0.96) | 8.53 (0.80) | 6.75 (0.76) | 7.09 (0.74) | 8.01 (0.84) | 8.16 (0.81) |
|  | Tourism | 0.60 (0.06) | 0.53 (0.06) | 1.01 (0.11) | 0.71 (0.07) | 0.49 (0.05) | 1.06 (0.11) | 1.43 (0.15) | 1.15 (0.11) |
| South Korea | Health | 32.97 (8.77) | 40.24 (8.62) | 44.89 (9.28) | 35.07 (7.35) | 40.13 (8.34) | 39.32 (5.20) | 42.91 (7.09) | 39.13 (6.27) |
|  | Humanitarian aid | 13.65 (3.63) | 7.86 (1.68) | 7.34 (1.52) | 9.14 (1.92) | 12.49 (2.60) | 13.59 (1.80) | 18.05 (2.98) | 20.50 (3.29) |
|  | Government and civil society | 32.45 (8.63) | 29.58 (6.33) | 47.55 (9.83) | 32.47 (6.81) | 37.27 (7.75) | 34.64 (4.58) | 31.73 (5.24) | 31.56 (5.06) |
|  | Education | 29.95 (7.96) | 31.66 (6.78) | 25.93 (5.36) | 31.25 (6.55) | 29.48 (6.13) | 33.41 (4.42) | 25.03 (4.13) | 25.36 (4.07) |
|  | Infrastructure | 43.50 (11.57) | 66.35 (14.20) | 57.69 (11.92) | 55.07 (11.55) | 50.85 (10.57) | 43.47 (5.75) | 52.11 (8.61) | 165.37 (26.51) |
|  | Agriculture | 32.69 (8.69) | 38.33 (8.21) | 46.31 (9.57) | 36.67 (7.69) | 32.63 (6.78) | 39.10 (5.17) | 37.26 (6.16) | 39.22 (6.29) |
|  | Refugees in donor country | 0.00 (0.00) | 0.00 (0.00) | 0.00 (0.00) | 0.00 (0.00) | 0.00 (0.00) | 0.00 (0.00) | 0.00 (0.00) | 0.00 (0.00) |
|  | Donor administration costs | 5.63 (1.50) | 11.04 (2.36) | 6.21 (1.28) | 5.75 (1.21) | 6.01 (1.25) | 6.78 (0.90) | 2.27 (0.37) | 2.32 (0.37) |
|  | Energy | 16.01 (4.26) | 29.56 (6.33) | 33.13 (6.85) | 48.93 (10.26) | 42.41 (8.82) | 32.17 (4.26) | 37.19 (6.14) | 38.37 (6.15) |
|  | Debt relief | 0.04 (0.01) | 7.66 (1.64) | 9.79 (2.02) | 6.86 (1.44) | 15.57 (3.24) | 8.12 (1.07) | 9.31 (1.54) | 8.88 (1.42) |
|  | Multisector | 9.72 (2.58) | 14.08 (3.01) | 17.20 (3.56) | 13.59 (2.85) | 16.34 (3.40) | 17.64 (2.33) | 12.83 (2.12) | 16.89 (2.71) |
|  | Water and sanitation | 18.13 (4.82) | 27.00 (5.78) | 26.47 (5.47) | 28.95 (6.07) | 26.37 (5.48) | 29.62 (3.92) | 22.04 (3.64) | 76.19 (12.21) |
|  | Financial services and business support | 10.87 (2.89) | 12.20 (2.61) | 16.98 (3.51) | 19.27 (4.04) | 19.43 (4.04) | 12.87 (1.70) | 16.81 (2.78) | 15.44 (2.48) |
|  | Environmental protection | 5.25 (1.40) | 4.57 (0.98) | 8.29 (1.71) | 11.33 (2.38) | 14.55 (3.02) | 16.51 (2.18) | 17.36 (2.87) | 14.27 (2.29) |
|  | Other social services | 14.39 (3.83) | 25.31 (5.42) | 20.70 (4.28) | 24.47 (5.13) | 23.72 (4.93) | 29.50 (3.90) | 30.96 (5.11) | 27.37 (4.39) |
|  | Conflict, peace and security | 1.02 (0.27) | 0.90 (0.19) | 0.90 (0.19) | 1.13 (0.24) | 0.94 (0.19) | 0.97 (0.13) | 1.64 (0.27) | 1.99 (0.32) |
|  | General budget support | 10.24 (2.72) | 9.08 (1.94) | 13.21 (2.73) | 7.13 (1.50) | 5.16 (1.07) | 5.63 (0.74) | 4.89 (0.81) | 3.25 (0.52) |
|  | Unspecified | 0.01 (0.00) | 0.64 (0.14) | 0.81 (0.17) | 1.25 (0.26) | 9.27 (1.93) | 9.99 (1.32) | 11.31 (1.87) | 11.61 (1.86) |
|  | Industry, construction and mining | 7.97 (2.12) | 6.99 (1.50) | 6.41 (1.33) | 5.29 (1.11) | 6.26 (1.30) | 4.33 (0.57) | 7.43 (1.23) | 5.84 (0.94) |
|  | Food aid and commodity assistance | 0.41 (0.11) | 0.30 (0.06) | 0.62 (0.13) | 0.53 (0.11) | 0.26 (0.05) | 0.27 (0.04) | 0.59 (0.10) | 0.54 (0.09) |
|  | Trade policy | 1.25 (0.33) | 2.54 (0.54) | 2.86 (0.59) | 1.72 (0.36) | 2.22 (0.46) | 6.05 (0.80) | 1.67 (0.28) | 2.84 (0.46) |
|  | Tourism | 0.33 (0.09) | 0.48 (0.10) | 0.67 (0.14) | 0.61 (0.13) | 0.82 (0.17) | 0.35 (0.05) | 0.39 (0.06) | 1.15 (0.18) |
| Austria | Health | 34.03 (5.69) | 30.07 (5.17) | 31.34 (5.14) | 28.69 (5.04) | 32.86 (5.46) | 33.34 (4.68) | 42.38 (6.12) | 32.56 (4.75) |
|  | Humanitarian aid | 39.67 (6.64) | 30.32 (5.21) | 35.87 (5.88) | 42.25 (7.43) | 39.79 (6.61) | 53.43 (7.50) | 49.82 (7.19) | 49.44 (7.22) |
|  | Government and civil society | 55.00 (9.20) | 47.91 (8.24) | 52.24 (8.56) | 51.20 (9.00) | 54.02 (8.97) | 54.62 (7.66) | 67.18 (9.69) | 79.52 (11.61) |
|  | Education | 40.63 (6.80) | 37.36 (6.43) | 35.02 (5.74) | 36.48 (6.41) | 35.68 (5.93) | 43.25 (6.07) | 43.93 (6.34) | 43.55 (6.36) |
|  | Infrastructure | 71.93 (12.03) | 84.29 (14.49) | 87.66 (14.36) | 74.56 (13.11) | 69.38 (11.53) | 74.93 (10.51) | 76.44 (11.03) | 92.68 (13.53) |
|  | Agriculture | 51.62 (8.64) | 43.40 (7.46) | 50.02 (8.20) | 50.30 (8.84) | 49.68 (8.25) | 65.67 (9.21) | 61.20 (8.83) | 58.56 (8.55) |
|  | Refugees in donor country | 0.00 (0.00) | 0.00 (0.00) | 0.31 (0.05) | 0.45 (0.08) | 0.00 (0.00) | 0.00 (0.00) | 0.00 (0.00) | 0.00 (0.00) |
|  | Donor administration costs | 14.30 (2.39) | 12.22 (2.10) | 13.90 (2.28) | 14.15 (2.49) | 13.23 (2.20) | 14.56 (2.04) | 14.86 (2.14) | 16.09 (2.35) |
|  | Energy | 45.25 (7.57) | 51.69 (8.89) | 49.77 (8.16) | 53.61 (9.42) | 47.10 (7.83) | 52.90 (7.42) | 64.14 (9.25) | 57.95 (8.46) |
|  | Debt relief | 3.37 (0.56) | 14.28 (2.45) | 3.59 (0.59) | 2.11 (0.37) | 8.83 (1.47) | 2.49 (0.35) | 1.74 (0.25) | 1.68 (0.25) |
|  | Multisector | 18.26 (3.05) | 20.45 (3.52) | 23.75 (3.89) | 22.72 (3.99) | 21.86 (3.63) | 32.79 (4.60) | 37.25 (5.37) | 38.95 (5.69) |
|  | Water and sanitation | 27.14 (4.54) | 31.60 (5.43) | 30.71 (5.03) | 33.46 (5.88) | 32.50 (5.40) | 34.52 (4.84) | 29.68 (4.28) | 41.74 (6.09) |
|  | Financial services and business support | 34.07 (5.70) | 36.88 (6.34) | 43.85 (7.19) | 46.83 (8.23) | 54.62 (9.07) | 46.72 (6.56) | 39.01 (5.63) | 45.24 (6.61) |
|  | Environmental protection | 29.27 (4.90) | 20.32 (3.49) | 20.71 (3.39) | 8.57 (1.51) | 33.40 (5.55) | 26.67 (3.74) | 25.63 (3.70) | 10.47 (1.53) |
|  | Other social services | 23.72 (3.97) | 25.04 (4.31) | 27.39 (4.49) | 26.65 (4.68) | 28.07 (4.66) | 29.72 (4.17) | 29.69 (4.28) | 29.09 (4.25) |
|  | Conflict, peace and security | 12.55 (2.10) | 6.50 (1.12) | 10.19 (1.67) | 11.24 (1.98) | 11.48 (1.91) | 12.88 (1.81) | 13.41 (1.93) | 16.34 (2.39) |
|  | General budget support | 27.16 (4.54) | 22.96 (3.95) | 25.17 (4.12) | 25.02 (4.40) | 17.21 (2.86) | 15.84 (2.22) | 14.63 (2.11) | 11.08 (1.62) |
|  | Unspecified | 9.39 (1.57) | 4.13 (0.71) | 3.93 (0.64) | 2.32 (0.41) | 4.71 (0.78) | 23.25 (3.26) | 3.78 (0.55) | 3.51 (0.51) |
|  | Industry, construction and mining | 23.09 (3.86) | 25.97 (4.47) | 11.05 (1.81) | 9.67 (1.70) | 13.96 (2.32) | 10.06 (1.41) | 21.15 (3.05) | 18.37 (2.68) |
|  | Food aid and commodity assistance | 5.32 (0.89) | 4.80 (0.82) | 4.65 (0.76) | 2.89 (0.51) | 4.96 (0.82) | 4.40 (0.62) | 4.32 (0.62) | 6.81 (0.99) |
|  | Trade policy | 5.80 (0.97) | 4.76 (0.82) | 6.57 (1.08) | 5.76 (1.01) | 5.47 (0.91) | 8.46 (1.19) | 5.26 (0.76) | 5.91 (0.86) |
|  | Tourism | 0.39 (0.06) | 0.34 (0.06) | 0.69 (0.11) | 0.46 (0.08) | 0.72 (0.12) | 0.75 (0.11) | 1.24 (0.18) | 1.22 (0.18) |
| Finland | Health | 77.73 (14.34) | 74.90 (14.21) | 87.25 (15.02) | 94.99 (14.62) | 69.25 (10.70) | 35.26 (7.62) | 37.66 (7.25) | 29.96 (5.89) |
|  | Humanitarian aid | 41.01 (7.57) | 33.94 (6.44) | 42.64 (7.34) | 44.60 (6.86) | 54.89 (8.48) | 54.71 (11.83) | 66.85 (12.87) | 71.00 (13.97) |
|  | Government and civil society | 44.76 (8.26) | 40.06 (7.60) | 45.58 (7.85) | 51.29 (7.89) | 51.57 (7.97) | 33.81 (7.31) | 46.72 (8.99) | 51.90 (10.21) |
|  | Education | 29.53 (5.45) | 26.54 (5.04) | 27.22 (4.69) | 28.64 (4.41) | 33.30 (5.14) | 26.75 (5.78) | 29.96 (5.77) | 29.68 (5.84) |
|  | Infrastructure | 44.15 (8.15) | 53.70 (10.19) | 57.35 (9.87) | 61.96 (9.53) | 52.10 (8.05) | 43.27 (9.35) | 48.07 (9.25) | 55.02 (10.83) |
|  | Agriculture | 31.41 (5.80) | 27.90 (5.29) | 33.71 (5.80) | 43.14 (6.64) | 37.03 (5.72) | 34.19 (7.39) | 34.53 (6.65) | 33.41 (6.57) |
|  | Refugees in donor country | 0.00 (0.00) | 0.00 (0.00) | 0.20 (0.03) | 0.28 (0.04) | 0.00 (0.00) | 0.00 (0.00) | 0.00 (0.00) | 0.00 (0.00) |
|  | Donor administration costs | 18.68 (3.45) | 18.18 (3.45) | 20.92 (3.60) | 18.73 (2.88) | 17.99 (2.78) | 16.33 (3.53) | 13.82 (2.66) | 12.84 (2.53) |
|  | Energy | 29.37 (5.42) | 32.47 (6.16) | 34.55 (5.95) | 40.65 (6.26) | 36.55 (5.65) | 29.30 (6.34) | 38.81 (7.47) | 34.42 (6.77) |
|  | Debt relief | 3.38 (0.62) | 12.97 (2.46) | 3.19 (0.55) | 2.27 (0.35) | 7.87 (1.22) | 2.23 (0.48) | 1.93 (0.37) | 1.82 (0.36) |
|  | Multisector | 26.82 (4.95) | 23.82 (4.52) | 26.09 (4.49) | 26.60 (4.09) | 22.51 (3.48) | 21.10 (4.56) | 28.63 (5.51) | 24.99 (4.92) |
|  | Water and sanitation | 17.85 (3.29) | 20.48 (3.89) | 21.57 (3.71) | 30.79 (4.74) | 25.85 (3.99) | 18.30 (3.96) | 16.74 (3.22) | 23.99 (4.72) |
|  | Financial services and business support | 22.22 (4.10) | 24.30 (4.61) | 28.71 (4.94) | 29.77 (4.58) | 37.62 (5.81) | 29.01 (6.27) | 24.81 (4.77) | 26.47 (5.21) |
|  | Environmental protection | 26.39 (4.87) | 34.38 (6.52) | 28.45 (4.90) | 38.29 (5.89) | 30.43 (4.70) | 17.12 (3.70) | 16.32 (3.14) | 16.48 (3.24) |
|  | Other social services | 20.47 (3.78) | 19.57 (3.71) | 23.28 (4.01) | 20.10 (3.09) | 33.90 (5.24) | 18.27 (3.95) | 17.86 (3.44) | 17.05 (3.35) |
|  | Conflict, peace and security | 9.54 (1.76) | 5.75 (1.09) | 8.22 (1.41) | 8.80 (1.35) | 8.31 (1.28) | 8.58 (1.85) | 9.82 (1.89) | 10.33 (2.03) |
|  | General budget support | 26.47 (4.88) | 16.81 (3.19) | 18.35 (3.16) | 20.44 (3.15) | 11.28 (1.74) | 10.19 (2.20) | 10.97 (2.11) | 7.08 (1.39) |
|  | Unspecified | 6.34 (1.17) | 6.02 (1.14) | 6.73 (1.16) | 5.57 (0.86) | 12.44 (1.92) | 9.12 (1.97) | 9.75 (1.88) | 7.31 (1.44) |
|  | Industry, construction and mining | 14.82 (2.74) | 16.58 (3.15) | 7.10 (1.22) | 6.56 (1.01) | 9.78 (1.51) | 5.69 (1.23) | 13.59 (2.62) | 10.84 (2.13) |
|  | Food aid and commodity assistance | 3.80 (0.70) | 3.90 (0.74) | 3.17 (0.55) | 3.68 (0.57) | 3.70 (0.57) | 3.50 (0.76) | 3.23 (0.62) | 4.78 (0.94) |
|  | Trade policy | 3.81 (0.70) | 2.81 (0.53) | 4.02 (0.69) | 3.49 (0.54) | 3.77 (0.58) | 3.86 (0.83) | 3.47 (0.67) | 3.36 (0.66) |
|  | Tourism | 0.31 (0.06) | 0.30 (0.06) | 0.51 (0.09) | 0.42 (0.06) | 0.58 (0.09) | 0.52 (0.11) | 0.64 (0.12) | 0.50 (0.10) |
| Ireland | Health | 38.84 (13.18) | 39.86 (14.39) | 44.27 (15.00) | 36.72 (12.93) | 35.68 (11.35) | 38.60 (9.45) | 37.23 (10.22) | 32.99 (8.17) |
|  | Humanitarian aid | 31.74 (10.78) | 28.69 (10.36) | 31.15 (10.56) | 34.19 (12.03) | 43.27 (13.76) | 72.59 (17.77) | 63.44 (17.41) | 72.21 (17.88) |
|  | Government and civil society | 22.72 (7.71) | 20.75 (7.50) | 22.20 (7.52) | 23.57 (8.30) | 23.74 (7.55) | 29.16 (7.14) | 34.18 (9.38) | 41.52 (10.28) |
|  | Education | 14.70 (4.99) | 14.53 (5.25) | 16.00 (5.42) | 14.39 (5.07) | 16.71 (5.31) | 23.24 (5.69) | 20.30 (5.57) | 22.46 (5.56) |
|  | Infrastructure | 23.32 (7.92) | 23.38 (8.44) | 27.44 (9.30) | 20.82 (7.33) | 22.21 (7.06) | 30.85 (7.55) | 27.70 (7.60) | 34.42 (8.52) |
|  | Agriculture | 14.34 (4.87) | 9.88 (3.57) | 14.70 (4.98) | 14.03 (4.94) | 14.85 (4.72) | 25.27 (6.19) | 20.80 (5.71) | 21.67 (5.37) |
|  | Refugees in donor country | 0.00 (0.00) | 0.00 (0.00) | 0.14 (0.05) | 0.21 (0.07) | 0.00 (0.00) | 0.00 (0.00) | 0.00 (0.00) | 0.00 (0.00) |
|  | Donor administration costs | 12.07 (4.10) | 11.92 (4.31) | 11.63 (3.94) | 12.02 (4.23) | 10.34 (3.29) | 10.81 (2.65) | 11.57 (3.18) | 13.43 (3.33) |
|  | Energy | 16.48 (5.59) | 15.98 (5.77) | 15.01 (5.09) | 15.59 (5.49) | 13.72 (4.36) | 22.48 (5.50) | 22.94 (6.30) | 22.65 (5.61) |
|  | Debt relief | 0.63 (0.21) | 0.46 (0.17) | 2.51 (0.85) | 0.05 (0.02) | 1.27 (0.40) | 0.09 (0.02) | 0.02 (0.01) | 0.06 (0.01) |
|  | Multisector | 12.28 (4.17) | 10.32 (3.73) | 11.63 (3.94) | 10.78 (3.79) | 10.33 (3.28) | 13.59 (3.33) | 16.86 (4.63) | 15.74 (3.90) |
|  | Water and sanitation | 8.53 (2.90) | 7.87 (2.84) | 8.40 (2.85) | 9.31 (3.28) | 9.56 (3.04) | 11.02 (2.70) | 9.18 (2.52) | 12.73 (3.15) |
|  | Financial services and business support | 14.22 (4.83) | 15.02 (5.42) | 16.78 (5.69) | 19.61 (6.90) | 23.86 (7.59) | 26.03 (6.37) | 18.20 (4.99) | 21.41 (5.30) |
|  | Environmental protection | 4.96 (1.68) | 5.80 (2.09) | 5.86 (1.98) | 5.18 (1.82) | 7.04 (2.24) | 9.35 (2.29) | 8.77 (2.41) | 7.86 (1.95) |
|  | Other social services | 9.82 (3.33) | 10.00 (3.61) | 11.55 (3.91) | 9.91 (3.49) | 12.64 (4.02) | 13.60 (3.33) | 10.81 (2.97) | 10.83 (2.68) |
|  | Conflict, peace and security | 6.03 (2.05) | 3.26 (1.18) | 4.76 (1.61) | 5.84 (2.06) | 6.07 (1.93) | 7.93 (1.94) | 8.47 (2.32) | 13.45 (3.33) |
|  | General budget support | 8.43 (2.86) | 7.48 (2.70) | 8.19 (2.77) | 9.22 (3.24) | 8.03 (2.55) | 7.98 (1.95) | 7.48 (2.05) | 7.01 (1.73) |
|  | Unspecified | 4.54 (1.54) | 3.47 (1.25) | 3.91 (1.32) | 3.80 (1.34) | 8.43 (2.68) | 12.64 (3.09) | 6.29 (1.73) | 6.92 (1.71) |
|  | Industry, construction and mining | 9.68 (3.29) | 10.59 (3.82) | 3.94 (1.33) | 3.44 (1.21) | 5.53 (1.76) | 4.67 (1.14) | 10.04 (2.75) | 9.07 (2.25) |
|  | Food aid and commodity assistance | 2.83 (0.96) | 3.42 (1.23) | 2.29 (0.78) | 2.04 (0.72) | 3.95 (1.26) | 6.03 (1.47) | 2.70 (0.74) | 5.22 (1.29) |
|  | Trade policy | 2.47 (0.84) | 1.63 (0.59) | 2.34 (0.79) | 2.21 (0.78) | 2.21 (0.70) | 3.08 (0.75) | 2.64 (0.72) | 2.60 (0.64) |
|  | Tourism | 0.20 (0.07) | 0.14 (0.05) | 0.25 (0.08) | 0.20 (0.07) | 0.22 (0.07) | 0.40 (0.10) | 0.47 (0.13) | 0.29 (0.07) |
| Portugal | Health | 11.74 (5.46) | 5.31 (2.86) | 10.44 (5.87) | 5.69 (3.25) | 7.96 (4.40) | 10.28 (4.28) | 14.98 (5.29) | 9.15 (3.58) |
|  | Humanitarian aid | 17.66 (8.22) | 14.60 (7.86) | 16.45 (9.26) | 19.62 (11.22) | 18.32 (10.13) | 26.12 (10.87) | 23.33 (8.24) | 21.93 (8.57) |
|  | Government and civil society | 17.20 (8.00) | 14.30 (7.70) | 13.77 (7.75) | 14.53 (8.30) | 15.62 (8.64) | 18.45 (7.68) | 27.89 (9.85) | 32.78 (12.81) |
|  | Education | 11.92 (5.55) | 8.89 (4.79) | 7.80 (4.39) | 6.98 (3.99) | 8.30 (4.59) | 11.97 (4.98) | 16.84 (5.95) | 15.80 (6.17) |
|  | Infrastructure | 23.85 (11.10) | 24.54 (13.22) | 23.38 (13.16) | 18.10 (10.35) | 18.27 (10.10) | 27.10 (11.28) | 33.01 (11.66) | 32.74 (12.80) |
|  | Agriculture | 13.82 (6.43) | 7.29 (3.92) | 9.27 (5.21) | 11.44 (6.54) | 11.36 (6.28) | 19.91 (8.29) | 22.40 (7.91) | 19.11 (7.47) |
|  | Refugees in donor country | 0.00 (0.00) | 0.00 (0.00) | 0.16 (0.09) | 0.24 (0.14) | 0.00 (0.00) | 0.00 (0.00) | 0.00 (0.00) | 0.00 (0.00) |
|  | Donor administration costs | 7.50 (3.49) | 7.07 (3.81) | 6.93 (3.90) | 7.27 (4.16) | 6.96 (3.85) | 7.83 (3.26) | 7.96 (2.81) | 8.22 (3.21) |
|  | Energy | 17.69 (8.23) | 15.03 (8.10) | 11.58 (6.52) | 11.68 (6.68) | 9.57 (5.29) | 18.45 (7.68) | 25.37 (8.96) | 20.73 (8.10) |
|  | Debt relief | 1.31 (0.61) | 1.92 (1.04) | 1.12 (0.63) | 1.05 (0.60) | 1.08 (0.60) | 1.01 (0.42) | 0.89 (0.31) | 0.86 (0.34) |
|  | Multisector | 7.45 (3.47) | 6.98 (3.76) | 7.11 (4.00) | 7.15 (4.08) | 6.51 (3.60) | 11.72 (4.88) | 17.69 (6.25) | 14.51 (5.67) |
|  | Water and sanitation | 7.99 (3.72) | 6.41 (3.45) | 5.27 (2.97) | 7.05 (4.03) | 6.79 (3.76) | 8.32 (3.46) | 10.63 (3.75) | 11.73 (4.59) |
|  | Financial services and business support | 15.24 (7.09) | 16.75 (9.02) | 16.37 (9.21) | 19.80 (11.32) | 22.65 (12.53) | 22.83 (9.50) | 17.06 (6.03) | 18.42 (7.20) |
|  | Environmental protection | 2.75 (1.28) | 3.63 (1.95) | 3.27 (1.84) | 2.69 (1.54) | 2.81 (1.55) | 6.09 (2.54) | 6.43 (2.27) | 4.22 (1.65) |
|  | Other social services | 7.26 (3.38) | 6.53 (3.52) | 7.36 (4.14) | 7.29 (4.17) | 8.54 (4.72) | 8.48 (3.53) | 8.56 (3.02) | 6.89 (2.69) |
|  | Conflict, peace and security | 6.29 (2.93) | 3.07 (1.65) | 4.72 (2.66) | 5.40 (3.08) | 5.68 (3.14) | 6.66 (2.77) | 6.69 (2.36) | 8.43 (3.30) |
|  | General budget support | 9.92 (4.61) | 9.10 (4.90) | 8.43 (4.74) | 10.51 (6.01) | 8.52 (4.71) | 7.84 (3.26) | 8.03 (2.84) | 5.93 (2.32) |
|  | Unspecified | 5.22 (2.43) | 2.30 (1.24) | 1.90 (1.07) | 1.07 (0.61) | 2.08 (1.15) | 2.36 (0.98) | 1.27 (0.45) | 0.71 (0.28) |
|  | Industry, construction and mining | 10.38 (4.83) | 11.85 (6.38) | 3.46 (1.95) | 3.22 (1.84) | 4.97 (2.75) | 3.86 (1.61) | 9.37 (3.31) | 7.82 (3.05) |
|  | Food aid and commodity assistance | 2.75 (1.28) | 2.49 (1.34) | 2.11 (1.19) | 1.27 (0.73) | 2.58 (1.43) | 2.35 (0.98) | 2.13 (0.75) | 3.43 (1.34) |
|  | Trade policy | 2.70 (1.25) | 1.57 (0.85) | 2.02 (1.14) | 2.30 (1.32) | 2.04 (1.13) | 2.05 (0.85) | 2.41 (0.85) | 2.19 (0.85) |
|  | Tourism | 0.18 (0.08) | 0.15 (0.08) | 0.20 (0.11) | 0.12 (0.07) | 0.11 (0.06) | 0.57 (0.24) | 0.64 (0.23) | 0.40 (0.16) |
| New Zealand | Health | 11.13 (12.45) | 9.61 (11.40) | 10.26 (10.44) | 8.12 (9.40) | 6.91 (7.81) | 6.92 (7.74) | 6.55 (8.41) | 6.69 (7.16) |
|  | Humanitarian aid | 9.11 (10.19) | 7.47 (8.86) | 7.70 (7.83) | 7.42 (8.59) | 8.73 (9.88) | 9.23 (10.32) | 11.83 (15.19) | 13.69 (14.66) |
|  | Government and civil society | 6.89 (7.70) | 4.19 (4.97) | 8.27 (8.42) | 5.52 (6.39) | 5.73 (6.48) | 5.60 (6.27) | 4.65 (5.97) | 5.14 (5.50) |
|  | Education | 5.16 (5.78) | 3.48 (4.13) | 4.62 (4.70) | 3.43 (3.97) | 3.56 (4.03) | 3.73 (4.17) | 2.29 (2.94) | 4.11 (4.40) |
|  | Infrastructure | 5.58 (6.24) | 5.34 (6.34) | 6.26 (6.37) | 3.67 (4.25) | 3.68 (4.16) | 3.39 (3.79) | 1.66 (2.13) | 3.06 (3.28) |
|  | Agriculture | 4.37 (4.89) | 2.36 (2.80) | 5.85 (5.95) | 2.70 (3.12) | 2.58 (2.92) | 2.78 (3.11) | 2.12 (2.72) | 3.17 (3.40) |
|  | Refugees in donor country | 0.00 (0.00) | 0.00 (0.00) | 0.00 (0.00) | 0.00 (0.00) | 0.00 (0.00) | 0.00 (0.00) | 0.00 (0.00) | 0.00 (0.00) |
|  | Donor administration costs | 2.53 (2.83) | 2.88 (3.42) | 2.70 (2.75) | 2.52 (2.92) | 1.83 (2.07) | 1.22 (1.37) | 1.16 (1.49) | 1.62 (1.74) |
|  | Energy | 1.97 (2.20) | 1.35 (1.61) | 4.00 (4.07) | 3.86 (4.47) | 3.38 (3.82) | 2.74 (3.06) | 1.95 (2.50) | 3.01 (3.23) |
|  | Debt relief | 0.01 (0.01) | 0.00 (0.00) | 0.25 (0.25) | 0.03 (0.03) | 0.67 (0.75) | 0.05 (0.06) | 0.03 (0.04) | 0.03 (0.03) |
|  | Multisector | 3.85 (4.30) | 3.13 (3.72) | 3.19 (3.24) | 1.99 (2.31) | 2.34 (2.64) | 1.73 (1.93) | 1.54 (1.98) | 1.25 (1.34) |
|  | Water and sanitation | 2.72 (3.05) | 1.64 (1.94) | 3.21 (3.26) | 2.21 (2.56) | 2.20 (2.49) | 2.46 (2.75) | 1.12 (1.43) | 2.10 (2.25) |
|  | Financial services and business support | 1.58 (1.77) | 0.93 (1.11) | 2.36 (2.40) | 1.65 (1.91) | 1.62 (1.83) | 1.35 (1.50) | 0.81 (1.04) | 0.90 (0.97) |
|  | Environmental protection | 2.35 (2.63) | 0.81 (0.96) | 3.68 (3.74) | 1.80 (2.09) | 2.58 (2.92) | 2.27 (2.54) | 1.80 (2.31) | 3.48 (3.73) |
|  | Other social services | 3.13 (3.50) | 2.18 (2.58) | 3.57 (3.63) | 2.43 (2.81) | 3.26 (3.69) | 3.47 (3.88) | 3.01 (3.87) | 2.68 (2.87) |
|  | Conflict, peace and security | 0.35 (0.39) | 0.35 (0.42) | 0.40 (0.40) | 0.42 (0.49) | 0.31 (0.35) | 0.11 (0.12) | 0.18 (0.23) | 0.18 (0.19) |
|  | General budget support | 0.01 (0.01) | 0.00 (0.00) | 0.02 (0.02) | 0.04 (0.04) | 0.01 (0.02) | 0.15 (0.17) | 0.12 (0.16) | 0.18 (0.19) |
|  | Unspecified | 0.00 (0.00) | 0.72 (0.85) | 0.98 (1.00) | 1.19 (1.38) | 2.65 (3.00) | 4.15 (4.64) | 3.76 (4.83) | 3.19 (3.41) |
|  | Industry, construction and mining | 1.20 (1.35) | 0.45 (0.53) | 0.88 (0.90) | 0.41 (0.48) | 0.51 (0.57) | 0.31 (0.34) | 0.41 (0.53) | 0.41 (0.43) |
|  | Food aid and commodity assistance | 0.22 (0.24) | 0.47 (0.56) | 0.23 (0.23) | 0.33 (0.39) | 0.29 (0.33) | 0.33 (0.37) | 0.15 (0.20) | 0.27 (0.29) |
|  | Trade policy | 0.18 (0.20) | 0.14 (0.17) | 0.45 (0.46) | 0.12 (0.14) | 0.16 (0.18) | 0.38 (0.43) | 0.08 (0.11) | 0.20 (0.21) |
|  | Tourism | 0.07 (0.08) | 0.05 (0.06) | 0.09 (0.09) | 0.07 (0.08) | 0.07 (0.08) | 0.04 (0.04) | 0.02 (0.03) | 0.05 (0.05) |
| Luxembourg | Health | 25.46 (20.38) | 24.57 (19.88) | 24.67 (19.66) | 23.55 (20.48) | 22.01 (19.65) | 24.02 (18.57) | 25.65 (19.88) | 25.51 (19.64) |
|  | Humanitarian aid | 8.09 (6.48) | 6.52 (5.27) | 8.19 (6.53) | 8.38 (7.29) | 8.57 (7.65) | 10.55 (8.16) | 11.42 (8.86) | 11.32 (8.72) |
|  | Government and civil society | 11.90 (9.53) | 9.98 (8.08) | 12.03 (9.59) | 12.30 (10.69) | 9.53 (8.51) | 10.62 (8.21) | 12.61 (9.77) | 13.40 (10.32) |
|  | Education | 6.35 (5.08) | 5.53 (4.47) | 8.06 (6.42) | 6.65 (5.78) | 4.61 (4.11) | 6.03 (4.66) | 5.72 (4.44) | 5.86 (4.51) |
|  | Infrastructure | 7.92 (6.34) | 9.76 (7.90) | 9.87 (7.87) | 7.23 (6.29) | 7.29 (6.51) | 8.71 (6.74) | 8.69 (6.74) | 9.32 (7.18) |
|  | Agriculture | 6.23 (4.98) | 5.73 (4.64) | 6.07 (4.84) | 5.21 (4.53) | 5.02 (4.48) | 7.78 (6.01) | 6.76 (5.24) | 6.39 (4.92) |
|  | Refugees in donor country | 0.00 (0.00) | 0.00 (0.00) | 0.04 (0.03) | 0.05 (0.04) | 0.00 (0.00) | 0.00 (0.00) | 0.00 (0.00) | 0.00 (0.00) |
|  | Donor administration costs | 3.94 (3.15) | 4.01 (3.25) | 4.60 (3.66) | 4.42 (3.85) | 3.10 (2.77) | 3.16 (2.44) | 3.30 (2.55) | 3.72 (2.86) |
|  | Energy | 5.57 (4.46) | 6.64 (5.37) | 5.75 (4.58) | 5.63 (4.90) | 5.11 (4.56) | 6.40 (4.95) | 7.25 (5.62) | 6.88 (5.30) |
|  | Debt relief | 0.15 (0.12) | 0.74 (0.60) | 0.22 (0.18) | 0.02 (0.02) | 2.09 (1.86) | 1.39 (1.08) | 0.03 (0.03) | 0.08 (0.06) |
|  | Multisector | 5.62 (4.50) | 4.92 (3.98) | 5.59 (4.45) | 4.28 (3.72) | 4.12 (3.68) | 4.68 (3.62) | 5.90 (4.57) | 5.31 (4.09) |
|  | Water and sanitation | 3.36 (2.69) | 4.16 (3.36) | 3.51 (2.80) | 3.51 (3.06) | 3.54 (3.16) | 4.31 (3.33) | 3.53 (2.73) | 4.43 (3.41) |
|  | Financial services and business support | 4.48 (3.59) | 4.65 (3.76) | 5.99 (4.77) | 5.28 (4.59) | 6.04 (5.39) | 5.58 (4.31) | 4.58 (3.55) | 4.97 (3.83) |
|  | Environmental protection | 2.62 (2.09) | 2.93 (2.37) | 2.97 (2.36) | 2.38 (2.07) | 2.66 (2.38) | 3.38 (2.62) | 3.16 (2.45) | 2.67 (2.06) |
|  | Other social services | 6.06 (4.85) | 6.53 (5.28) | 7.14 (5.69) | 4.56 (3.97) | 7.23 (6.45) | 7.28 (5.63) | 7.09 (5.49) | 6.29 (4.84) |
|  | Conflict, peace and security | 1.81 (1.45) | 1.10 (0.89) | 1.81 (1.44) | 1.85 (1.61) | 1.76 (1.57) | 1.68 (1.30) | 1.84 (1.43) | 2.23 (1.72) |
|  | General budget support | 3.00 (2.40) | 2.45 (1.98) | 2.00 (1.59) | 2.44 (2.12) | 1.75 (1.56) | 1.74 (1.35) | 1.73 (1.34) | 1.44 (1.11) |
|  | Unspecified | 1.07 (0.86) | 1.45 (1.17) | 1.60 (1.28) | 1.25 (1.08) | 2.45 (2.19) | 3.40 (2.63) | 4.12 (3.19) | 7.01 (5.40) |
|  | Industry, construction and mining | 3.02 (2.42) | 3.18 (2.58) | 1.53 (1.22) | 1.06 (0.92) | 1.51 (1.35) | 1.20 (0.93) | 2.44 (1.90) | 2.05 (1.58) |
|  | Food aid and commodity assistance | 0.67 (0.53) | 0.64 (0.52) | 0.63 (0.50) | 0.37 (0.32) | 0.58 (0.52) | 0.56 (0.43) | 0.51 (0.39) | 0.77 (0.59) |
|  | Trade policy | 0.77 (0.61) | 0.65 (0.53) | 0.90 (0.72) | 0.61 (0.53) | 0.58 (0.52) | 1.07 (0.83) | 0.61 (0.47) | 0.65 (0.50) |
|  | Tourism | 0.06 (0.05) | 0.06 (0.05) | 0.11 (0.09) | 0.07 (0.07) | 0.09 (0.08) | 0.09 (0.07) | 0.11 (0.09) | 0.11 (0.08) |
| Poland | Health | NA | NA | 14.25 (4.32) | 13.63 (4.06) | 17.12 (4.66) | 24.03 (4.15) | 24.31 (5.02) | 17.53 (3.36) |
|  | Humanitarian aid | NA | NA | 32.85 (9.97) | 38.37 (11.42) | 36.45 (9.92) | 58.89 (10.18) | 46.55 (9.62) | 48.08 (9.23) |
|  | Government and civil society | NA | NA | 27.66 (8.39) | 28.39 (8.45) | 33.41 (9.09) | 43.82 (7.58) | 50.08 (10.35) | 66.99 (12.85) |
|  | Education | NA | NA | 15.47 (4.69) | 13.36 (3.98) | 18.31 (4.98) | 28.27 (4.89) | 27.56 (5.69) | 28.59 (5.49) |
|  | Infrastructure | NA | NA | 46.26 (14.04) | 34.43 (10.25) | 37.54 (10.22) | 56.68 (9.80) | 45.75 (9.45) | 73.11 (14.03) |
|  | Agriculture | NA | NA | 18.14 (5.50) | 21.93 (6.53) | 24.49 (6.66) | 45.75 (7.91) | 31.28 (6.46) | 36.42 (6.99) |
|  | Refugees in donor country | NA | NA | 0.32 (0.10) | 0.47 (0.14) | 0.00 (0.00) | 0.00 (0.00) | 0.00 (0.00) | 0.00 (0.00) |
|  | Donor administration costs | NA | NA | 13.58 (4.12) | 13.91 (4.14) | 13.46 (3.66) | 16.20 (2.80) | 17.03 (3.52) | 19.01 (3.65) |
|  | Energy | NA | NA | 22.85 (6.93) | 22.32 (6.64) | 20.39 (5.55) | 40.74 (7.04) | 36.42 (7.52) | 36.28 (6.96) |
|  | Debt relief | NA | NA | 0.07 (0.02) | 0.02 (0.01) | 0.98 (0.27) | 0.00 (0.00) | 0.00 (0.00) | 0.00 (0.00) |
|  | Multisector | NA | NA | 14.87 (4.51) | 14.38 (4.28) | 13.74 (3.74) | 22.35 (3.86) | 26.86 (5.55) | 25.64 (4.92) |
|  | Water and sanitation | NA | NA | 10.23 (3.10) | 13.35 (3.97) | 14.30 (3.89) | 17.37 (3.00) | 12.64 (2.61) | 24.12 (4.63) |
|  | Financial services and business support | NA | NA | 32.74 (9.93) | 38.34 (11.41) | 46.02 (12.52) | 51.24 (8.86) | 31.78 (6.57) | 39.10 (7.50) |
|  | Environmental protection | NA | NA | 6.59 (2.00) | 5.24 (1.56) | 7.71 (2.10) | 14.01 (2.42) | 11.37 (2.35) | 9.29 (1.78) |
|  | Other social services | NA | NA | 13.28 (4.03) | 14.00 (4.16) | 18.00 (4.90) | 19.62 (3.39) | 11.85 (2.45) | 14.06 (2.70) |
|  | Conflict, peace and security | NA | NA | 9.84 (2.99) | 10.81 (3.22) | 11.56 (3.15) | 15.18 (2.63) | 14.57 (3.01) | 19.18 (3.68) |
|  | General budget support | NA | NA | 16.44 (4.99) | 20.27 (6.03) | 16.75 (4.56) | 15.95 (2.76) | 14.11 (2.91) | 13.48 (2.59) |
|  | Unspecified | NA | NA | 3.81 (1.16) | 2.07 (0.62) | 3.42 (0.93) | 4.42 (0.76) | 3.38 (0.70) | 3.22 (0.62) |
|  | Industry, construction and mining | NA | NA | 6.95 (2.11) | 6.23 (1.85) | 10.31 (2.80) | 8.93 (1.54) | 17.68 (3.65) | 16.78 (3.22) |
|  | Food aid and commodity assistance | NA | NA | 4.24 (1.29) | 2.47 (0.74) | 5.12 (1.39) | 5.25 (0.91) | 4.32 (0.89) | 7.72 (1.48) |
|  | Trade policy | NA | NA | 4.06 (1.23) | 4.46 (1.33) | 4.22 (1.15) | 5.39 (0.93) | 4.74 (0.98) | 4.53 (0.87) |
|  | Tourism | NA | NA | 0.40 (0.12) | 0.23 (0.07) | 0.28 (0.08) | 1.00 (0.17) | 1.05 (0.22) | 0.56 (0.11) |
| Greece | Health | 9.56 (4.34) | 6.23 (3.21) | 9.15 (5.34) | 7.42 (4.12) | 8.39 (4.69) | 10.31 (4.57) | 13.41 (5.57) | 8.66 (3.44) |
|  | Humanitarian aid | 20.13 (9.15) | 16.39 (8.44) | 17.10 (9.98) | 20.11 (11.17) | 18.99 (10.61) | 26.40 (11.70) | 23.07 (9.58) | 22.62 (8.98) |
|  | Government and civil society | 16.30 (7.41) | 15.35 (7.91) | 13.85 (8.08) | 14.33 (7.96) | 15.81 (8.83) | 18.35 (8.13) | 25.64 (10.65) | 32.26 (12.81) |
|  | Education | 10.44 (4.75) | 8.88 (4.57) | 7.86 (4.59) | 6.79 (3.77) | 8.36 (4.67) | 11.11 (4.92) | 14.31 (5.95) | 14.07 (5.59) |
|  | Infrastructure | 24.44 (11.11) | 25.22 (12.99) | 23.79 (13.89) | 17.85 (9.92) | 18.41 (10.29) | 24.30 (10.77) | 23.64 (9.82) | 25.83 (10.26) |
|  | Agriculture | 12.76 (5.80) | 7.04 (3.62) | 9.51 (5.55) | 11.29 (6.27) | 11.50 (6.43) | 18.65 (8.27) | 17.06 (7.09) | 18.26 (7.25) |
|  | Refugees in donor country | 0.00 (0.00) | 0.00 (0.00) | 0.17 (0.10) | 0.25 (0.14) | 0.00 (0.00) | 0.00 (0.00) | 0.00 (0.00) | 0.00 (0.00) |
|  | Donor administration costs | 8.98 (4.08) | 7.32 (3.77) | 6.92 (4.04) | 7.15 (3.97) | 6.98 (3.90) | 7.39 (3.28) | 8.17 (3.39) | 8.71 (3.46) |
|  | Energy | 19.65 (8.93) | 16.52 (8.51) | 11.70 (6.83) | 11.47 (6.37) | 9.48 (5.30) | 17.24 (7.64) | 19.15 (7.96) | 18.12 (7.20) |
|  | Debt relief | 0.88 (0.40) | 0.63 (0.33) | 0.02 (0.01) | 0.01 (0.01) | 0.00 (0.00) | 0.00 (0.00) | 0.01 (0.00) | 0.01 (0.00) |
|  | Multisector | 8.07 (3.67) | 7.28 (3.75) | 7.32 (4.27) | 7.16 (3.98) | 6.64 (3.71) | 10.17 (4.51) | 13.54 (5.62) | 12.78 (5.08) |
|  | Water and sanitation | 7.61 (3.46) | 6.40 (3.30) | 5.20 (3.03) | 6.88 (3.82) | 6.76 (3.78) | 6.66 (2.95) | 7.06 (2.93) | 8.36 (3.32) |
|  | Financial services and business support | 17.30 (7.86) | 18.82 (9.69) | 16.87 (9.85) | 20.00 (11.11) | 23.50 (13.13) | 22.91 (10.15) | 16.11 (6.69) | 18.78 (7.46) |
|  | Environmental protection | 2.93 (1.33) | 4.01 (2.07) | 3.40 (1.98) | 2.70 (1.50) | 3.06 (1.71) | 6.64 (2.94) | 5.86 (2.44) | 4.46 (1.77) |
|  | Other social services | 6.79 (3.09) | 6.17 (3.18) | 6.18 (3.61) | 6.46 (3.59) | 7.82 (4.37) | 6.89 (3.05) | 7.12 (2.96) | 7.61 (3.02) |
|  | Conflict, peace and security | 7.65 (3.48) | 3.51 (1.81) | 4.94 (2.88) | 5.50 (3.06) | 5.95 (3.32) | 7.07 (3.13) | 7.16 (2.97) | 8.89 (3.53) |
|  | General budget support | 11.97 (5.44) | 10.33 (5.32) | 8.55 (4.99) | 10.65 (5.91) | 8.89 (4.97) | 7.28 (3.23) | 6.83 (2.84) | 6.20 (2.46) |
|  | Unspecified | 6.46 (2.94) | 2.61 (1.34) | 1.98 (1.16) | 1.09 (0.60) | 1.43 (0.80) | 1.06 (0.47) | 0.67 (0.28) | 0.90 (0.36) |
|  | Industry, construction and mining | 11.70 (5.32) | 13.46 (6.93) | 3.58 (2.09) | 3.24 (1.80) | 5.14 (2.87) | 3.82 (1.69) | 8.93 (3.71) | 8.00 (3.18) |
|  | Food aid and commodity assistance | 3.33 (1.51) | 2.86 (1.47) | 2.20 (1.28) | 1.29 (0.72) | 2.70 (1.51) | 2.38 (1.05) | 2.13 (0.89) | 3.58 (1.42) |
|  | Trade policy | 3.13 (1.42) | 1.74 (0.90) | 2.09 (1.22) | 2.33 (1.29) | 2.11 (1.18) | 2.03 (0.90) | 2.37 (0.99) | 2.23 (0.88) |
|  | Tourism | 0.19 (0.08) | 0.14 (0.07) | 0.20 (0.12) | 0.12 (0.06) | 0.11 (0.06) | 0.34 (0.15) | 0.64 (0.27) | 0.63 (0.25) |
| Czech | Health | 7.32 (4.62) | 6.23 (4.08) | 7.56 (5.00) | 5.06 (3.32) | 6.96 (4.53) | 9.72 (4.40) | 11.72 (4.75) | 6.79 (3.31) |
|  | Humanitarian aid | 13.23 (8.34) | 11.03 (7.23) | 12.79 (8.46) | 16.18 (10.63) | 14.55 (9.47) | 24.69 (11.18) | 21.86 (8.86) | 21.39 (10.43) |
|  | Government and civil society | 12.50 (7.89) | 12.13 (7.95) | 11.42 (7.55) | 12.76 (8.38) | 13.22 (8.61) | 17.90 (8.10) | 23.45 (9.51) | 27.19 (13.25) |
|  | Education | 8.45 (5.33) | 7.57 (4.96) | 6.56 (4.34) | 6.39 (4.20) | 7.16 (4.66) | 11.28 (5.10) | 12.58 (5.10) | 11.64 (5.67) |
|  | Infrastructure | 17.00 (10.72) | 18.93 (12.41) | 18.74 (12.39) | 15.35 (10.08) | 14.82 (9.65) | 23.40 (10.59) | 21.16 (8.57) | 21.60 (10.53) |
|  | Agriculture | 9.84 (6.20) | 6.79 (4.45) | 7.54 (4.99) | 10.28 (6.75) | 9.80 (6.38) | 18.70 (8.46) | 14.68 (5.95) | 15.32 (7.47) |
|  | Refugees in donor country | 0.00 (0.00) | 0.00 (0.00) | 0.12 (0.08) | 0.20 (0.13) | 0.00 (0.00) | 0.00 (0.00) | 0.00 (0.00) | 0.00 (0.00) |
|  | Donor administration costs | 5.64 (3.55) | 4.87 (3.19) | 5.25 (3.47) | 5.85 (3.84) | 5.45 (3.55) | 6.96 (3.15) | 7.74 (3.14) | 7.45 (3.63) |
|  | Energy | 13.41 (8.46) | 12.74 (8.35) | 9.44 (6.24) | 10.37 (6.81) | 8.06 (5.24) | 16.78 (7.59) | 17.11 (6.93) | 15.13 (7.37) |
|  | Debt relief | 0.55 (0.35) | 0.62 (0.41) | 0.08 (0.05) | 0.01 (0.01) | 0.33 (0.22) | 0.00 (0.00) | 0.00 (0.00) | 0.00 (0.00) |
|  | Multisector | 5.30 (3.34) | 5.52 (3.62) | 5.95 (3.93) | 6.24 (4.10) | 5.51 (3.58) | 9.31 (4.22) | 11.81 (4.79) | 10.49 (5.11) |
|  | Water and sanitation | 5.59 (3.52) | 5.62 (3.68) | 4.41 (2.92) | 6.18 (4.06) | 5.71 (3.71) | 6.97 (3.16) | 5.97 (2.42) | 6.77 (3.30) |
|  | Financial services and business support | 11.55 (7.28) | 12.87 (8.44) | 12.98 (8.58) | 16.41 (10.78) | 18.26 (11.89) | 21.51 (9.74) | 14.66 (5.94) | 15.78 (7.69) |
|  | Environmental protection | 2.00 (1.26) | 2.85 (1.87) | 2.60 (1.72) | 3.40 (2.23) | 4.70 (3.06) | 7.32 (3.31) | 6.30 (2.55) | 4.64 (2.26) |
|  | Other social services | 5.35 (3.38) | 5.76 (3.77) | 5.81 (3.84) | 6.43 (4.22) | 7.19 (4.68) | 7.77 (3.52) | 6.02 (2.44) | 6.08 (2.96) |
|  | Conflict, peace and security | 4.86 (3.07) | 2.43 (1.59) | 3.79 (2.50) | 4.58 (3.01) | 4.64 (3.02) | 6.41 (2.90) | 6.44 (2.61) | 7.57 (3.69) |
|  | General budget support | 7.52 (4.74) | 11.12 (7.29) | 17.24 (11.40) | 8.53 (5.60) | 6.68 (4.35) | 6.75 (3.05) | 6.46 (2.62) | 5.32 (2.59) |
|  | Unspecified | 4.06 (2.56) | 1.70 (1.11) | 1.46 (0.97) | 0.87 (0.57) | 1.72 (1.12) | 1.66 (0.75) | 3.26 (1.32) | 0.77 (0.38) |
|  | Industry, construction and mining | 7.84 (4.94) | 9.18 (6.02) | 2.81 (1.86) | 2.76 (1.81) | 4.09 (2.66) | 3.69 (1.67) | 8.14 (3.30) | 6.76 (3.29) |
|  | Food aid and commodity assistance | 2.14 (1.35) | 1.89 (1.24) | 1.65 (1.09) | 1.05 (0.69) | 2.04 (1.33) | 2.21 (1.00) | 1.98 (0.80) | 3.06 (1.49) |
|  | Trade policy | 2.08 (1.31) | 1.31 (0.86) | 1.66 (1.10) | 1.93 (1.27) | 1.67 (1.09) | 2.12 (0.96) | 2.18 (0.88) | 1.86 (0.91) |
|  | Tourism | 0.12 (0.07) | 0.10 (0.06) | 0.16 (0.11) | 0.10 (0.07) | 0.11 (0.07) | 0.50 (0.23) | 0.38 (0.16) | 0.33 (0.16) |
| Hungary | Health | NA | NA | NA | 4.80 (4.37) | 6.13 (5.01) | 7.36 (4.52) | 6.06 (5.22) | 6.28 (4.03) |
|  | Humanitarian aid | NA | NA | NA | 11.40 (10.37) | 11.52 (9.43) | 17.16 (10.56) | 10.32 (8.89) | 14.75 (9.47) |
|  | Government and civil society | NA | NA | NA | 9.43 (8.59) | 11.20 (9.17) | 13.26 (8.15) | 11.22 (9.67) | 18.42 (11.82) |
|  | Education | NA | NA | NA | 5.25 (4.78) | 6.66 (5.45) | 8.66 (5.32) | 6.16 (5.31) | 8.19 (5.26) |
|  | Infrastructure | NA | NA | NA | 11.53 (10.49) | 12.50 (10.23) | 16.77 (10.32) | 10.31 (8.89) | 17.30 (11.10) |
|  | Agriculture | NA | NA | NA | 7.90 (7.19) | 8.59 (7.03) | 13.86 (8.52) | 7.28 (6.28) | 10.78 (6.92) |
|  | Refugees in donor country | NA | NA | NA | 0.14 (0.12) | 0.00 (0.00) | 0.00 (0.00) | 0.00 (0.00) | 0.00 (0.00) |
|  | Donor administration costs | NA | NA | NA | 4.03 (3.66) | 4.02 (3.29) | 4.62 (2.84) | 3.59 (3.09) | 5.81 (3.73) |
|  | Energy | NA | NA | NA | 8.10 (7.37) | 7.24 (5.92) | 12.13 (7.46) | 8.33 (7.18) | 10.28 (6.60) |
|  | Debt relief | NA | NA | NA | 0.02 (0.01) | 0.74 (0.61) | 0.00 (0.00) | 0.00 (0.00) | 0.00 (0.00) |
|  | Multisector | NA | NA | NA | 4.21 (3.84) | 4.19 (3.43) | 6.73 (4.14) | 6.08 (5.24) | 7.43 (4.77) |
|  | Water and sanitation | NA | NA | NA | 4.72 (4.29) | 4.98 (4.08) | 5.40 (3.32) | 2.98 (2.57) | 5.83 (3.74) |
|  | Financial services and business support | NA | NA | NA | 11.63 (10.58) | 14.48 (11.85) | 14.81 (9.11) | 7.04 (6.07) | 10.72 (6.88) |
|  | Environmental protection | NA | NA | NA | 1.67 (1.52) | 2.58 (2.11) | 4.09 (2.51) | 2.79 (2.41) | 2.59 (1.66) |
|  | Other social services | NA | NA | NA | 4.87 (4.43) | 6.11 (5.00) | 6.10 (3.75) | 2.61 (2.25) | 4.11 (2.64) |
|  | Conflict, peace and security | NA | NA | NA | 3.05 (2.78) | 3.37 (2.76) | 4.44 (2.73) | 3.23 (2.79) | 5.08 (3.26) |
|  | General budget support | NA | NA | NA | 5.95 (5.41) | 5.00 (4.09) | 4.64 (2.86) | 3.18 (2.74) | 3.58 (2.30) |
|  | Unspecified | NA | NA | NA | 0.60 (0.55) | 1.11 (0.91) | 1.29 (0.80) | 0.74 (0.64) | 1.26 (0.81) |
|  | Industry, construction and mining | NA | NA | NA | 2.02 (1.84) | 3.36 (2.75) | 2.65 (1.63) | 3.92 (3.38) | 4.59 (2.95) |
|  | Food aid and commodity assistance | NA | NA | NA | 0.74 (0.68) | 1.56 (1.28) | 1.51 (0.93) | 0.95 (0.82) | 2.07 (1.33) |
|  | Trade policy | NA | NA | NA | 1.39 (1.26) | 1.37 (1.12) | 1.69 (1.04) | 1.05 (0.90) | 1.28 (0.82) |
|  | Tourism | NA | NA | NA | 0.08 (0.07) | 0.11 (0.09) | 0.37 (0.23) | 0.32 (0.28) | 0.11 (0.07) |
| Slovak Republic | Health | NA | NA | 2.55 (4.01) | 2.54 (4.17) | 3.68 (4.97) | 3.77 (4.26) | 4.50 (5.03) | 3.45 (3.28) |
|  | Humanitarian aid | NA | NA | 5.89 (9.26) | 6.95 (11.41) | 7.04 (9.51) | 11.44 (12.92) | 9.44 (10.55) | 8.81 (8.35) |
|  | Government and civil society | NA | NA | 5.06 (7.97) | 5.12 (8.40) | 6.79 (9.18) | 7.04 (7.95) | 9.46 (10.56) | 12.47 (11.83) |
|  | Education | NA | NA | 2.81 (4.42) | 2.50 (4.09) | 3.98 (5.37) | 4.25 (4.80) | 5.06 (5.65) | 5.42 (5.14) |
|  | Infrastructure | NA | NA | 8.33 (13.10) | 6.31 (10.35) | 7.52 (10.16) | 9.15 (10.33) | 8.53 (9.53) | 9.91 (9.40) |
|  | Agriculture | NA | NA | 3.28 (5.15) | 4.05 (6.65) | 5.14 (6.94) | 7.02 (7.93) | 5.75 (6.42) | 7.11 (6.75) |
|  | Refugees in donor country | NA | NA | 0.06 (0.09) | 0.08 (0.14) | 0.00 (0.00) | 0.00 (0.00) | 0.00 (0.00) | 0.00 (0.00) |
|  | Donor administration costs | NA | NA | 2.42 (3.81) | 2.51 (4.13) | 2.45 (3.31) | 2.88 (3.25) | 3.23 (3.61) | 3.38 (3.21) |
|  | Energy | NA | NA | 4.12 (6.49) | 4.13 (6.77) | 4.32 (5.84) | 6.49 (7.33) | 6.76 (7.56) | 6.96 (6.60) |
|  | Debt relief | NA | NA | 0.02 (0.03) | 0.00 (0.01) | 0.42 (0.57) | 0.00 (0.00) | 0.00 (0.00) | 0.00 (0.00) |
|  | Multisector | NA | NA | 2.78 (4.37) | 2.49 (4.09) | 2.64 (3.57) | 4.01 (4.53) | 5.06 (5.65) | 4.80 (4.56) |
|  | Water and sanitation | NA | NA | 1.86 (2.93) | 2.46 (4.04) | 2.98 (4.03) | 2.53 (2.85) | 2.31 (2.58) | 3.21 (3.04) |
|  | Financial services and business support | NA | NA | 5.87 (9.23) | 6.96 (11.41) | 8.76 (11.83) | 8.64 (9.75) | 5.96 (6.65) | 7.22 (6.85) |
|  | Environmental protection | NA | NA | 1.19 (1.87) | 0.94 (1.55) | 1.58 (2.13) | 2.29 (2.59) | 2.14 (2.39) | 1.75 (1.66) |
|  | Other social services | NA | NA | 2.40 (3.78) | 2.58 (4.23) | 3.69 (4.98) | 2.92 (3.30) | 2.15 (2.40) | 3.10 (2.94) |
|  | Conflict, peace and security | NA | NA | 1.82 (2.86) | 1.89 (3.11) | 2.10 (2.84) | 2.70 (3.04) | 2.74 (3.06) | 3.44 (3.27) |
|  | General budget support | NA | NA | 6.80 (10.70) | 3.66 (6.01) | 3.04 (4.11) | 2.74 (3.09) | 2.68 (2.99) | 2.39 (2.27) |
|  | Unspecified | NA | NA | 0.68 (1.07) | 0.38 (0.62) | 0.86 (1.17) | 0.71 (0.80) | 0.76 (0.85) | 1.08 (1.03) |
|  | Industry, construction and mining | NA | NA | 1.25 (1.97) | 1.14 (1.87) | 2.02 (2.73) | 1.44 (1.62) | 3.31 (3.70) | 3.08 (2.92) |
|  | Food aid and commodity assistance | NA | NA | 0.76 (1.19) | 0.45 (0.74) | 0.96 (1.29) | 0.92 (1.04) | 0.83 (0.92) | 1.38 (1.31) |
|  | Trade policy | NA | NA | 0.73 (1.15) | 0.81 (1.33) | 0.83 (1.12) | 0.76 (0.86) | 0.89 (1.00) | 0.86 (0.81) |
|  | Tourism | NA | NA | 0.07 (0.11) | 0.04 (0.07) | 0.07 (0.09) | 0.13 (0.15) | 0.16 (0.17) | 0.08 (0.07) |
| Slovenia | Health | 1.87 (4.63) | 1.80 (4.62) | 1.81 (4.68) | 1.57 (4.04) | 1.95 (4.58) | 2.51 (4.24) | 2.84 (5.22) | 1.77 (3.26) |
|  | Humanitarian aid | 3.41 (8.45) | 2.94 (7.54) | 3.46 (8.95) | 4.05 (10.44) | 4.00 (9.41) | 6.05 (10.21) | 5.19 (9.52) | 4.98 (9.19) |
|  | Government and civil society | 3.25 (8.06) | 3.24 (8.32) | 3.29 (8.52) | 3.22 (8.29) | 3.75 (8.82) | 5.12 (8.65) | 6.21 (11.40) | 7.62 (14.05) |
|  | Education | 2.18 (5.40) | 2.01 (5.16) | 1.92 (4.95) | 1.63 (4.20) | 2.07 (4.86) | 2.97 (5.01) | 3.11 (5.71) | 3.00 (5.53) |
|  | Infrastructure | 4.34 (10.76) | 5.02 (12.89) | 5.29 (13.68) | 3.88 (9.99) | 4.19 (9.84) | 5.88 (9.93) | 5.17 (9.50) | 5.54 (10.21) |
|  | Agriculture | 2.59 (6.41) | 1.90 (4.87) | 2.33 (6.02) | 2.67 (6.87) | 2.86 (6.73) | 4.95 (8.35) | 3.70 (6.80) | 3.92 (7.23) |
|  | Refugees in donor country | 0.00 (0.00) | 0.00 (0.00) | 0.03 (0.09) | 0.05 (0.13) | 0.00 (0.00) | 0.00 (0.00) | 0.00 (0.00) | 0.00 (0.00) |
|  | Donor administration costs | 1.47 (3.65) | 1.30 (3.33) | 1.49 (3.84) | 1.50 (3.86) | 1.53 (3.59) | 2.41 (4.07) | 2.50 (4.59) | 2.64 (4.87) |
|  | Energy | 3.46 (8.57) | 3.40 (8.73) | 2.75 (7.12) | 2.66 (6.86) | 2.34 (5.51) | 4.28 (7.22) | 4.30 (7.89) | 3.97 (7.32) |
|  | Debt relief | 0.14 (0.35) | 0.11 (0.28) | 0.03 (0.09) | 0.00 (0.01) | 0.13 (0.32) | 0.00 (0.00) | 0.00 (0.00) | 0.00 (0.00) |
|  | Multisector | 1.48 (3.67) | 1.50 (3.85) | 1.69 (4.38) | 1.60 (4.11) | 1.56 (3.66) | 2.35 (3.97) | 2.96 (5.44) | 2.72 (5.02) |
|  | Water and sanitation | 1.47 (3.65) | 1.55 (3.97) | 1.36 (3.51) | 1.59 (4.09) | 1.65 (3.89) | 1.93 (3.25) | 1.53 (2.81) | 1.78 (3.29) |
|  | Financial services and business support | 2.95 (7.32) | 3.41 (8.74) | 3.59 (9.27) | 4.10 (10.56) | 5.06 (11.90) | 5.26 (8.89) | 3.54 (6.50) | 4.03 (7.43) |
|  | Environmental protection | 1.17 (2.89) | 1.35 (3.46) | 1.34 (3.47) | 1.21 (3.11) | 1.58 (3.71) | 2.50 (4.22) | 1.98 (3.64) | 1.58 (2.91) |
|  | Other social services | 1.37 (3.38) | 1.55 (3.97) | 1.67 (4.32) | 1.62 (4.18) | 2.01 (4.73) | 2.09 (3.54) | 1.51 (2.77) | 1.59 (2.93) |
|  | Conflict, peace and security | 1.29 (3.20) | 0.66 (1.69) | 1.04 (2.69) | 1.15 (2.97) | 1.28 (3.01) | 1.57 (2.65) | 1.57 (2.89) | 1.92 (3.54) |
|  | General budget support | 1.93 (4.78) | 1.78 (4.57) | 1.70 (4.39) | 2.12 (5.46) | 1.82 (4.29) | 1.63 (2.76) | 1.52 (2.79) | 1.34 (2.47) |
|  | Unspecified | 1.04 (2.58) | 0.45 (1.15) | 0.40 (1.03) | 0.22 (0.57) | 0.65 (1.52) | 0.48 (0.80) | 0.53 (0.97) | 0.38 (0.69) |
|  | Industry, construction and mining | 2.01 (4.97) | 2.43 (6.24) | 0.80 (2.07) | 0.70 (1.79) | 1.15 (2.70) | 0.93 (1.58) | 1.96 (3.61) | 1.72 (3.17) |
|  | Food aid and commodity assistance | 0.55 (1.36) | 0.50 (1.28) | 0.45 (1.16) | 0.26 (0.68) | 0.56 (1.31) | 0.54 (0.91) | 0.47 (0.87) | 0.77 (1.43) |
|  | Trade policy | 0.53 (1.32) | 0.35 (0.89) | 0.47 (1.21) | 0.48 (1.24) | 0.47 (1.10) | 0.57 (0.97) | 0.52 (0.96) | 0.48 (0.88) |
|  | Tourism | 0.03 (0.08) | 0.03 (0.07) | 0.05 (0.12) | 0.03 (0.07) | 0.03 (0.08) | 0.08 (0.13) | 0.09 (0.17) | 0.04 (0.08) |
| Iceland | Health | 0.84 (11.69) | 0.58 (8.66) | 0.61 (8.55) | 0.49 (6.39) | 0.84 (7.48) | 0.84 (6.60) | 1.21 (8.44) | 0.98 (7.52) |
|  | Humanitarian aid | 0.23 (3.18) | 0.09 (1.42) | 0.11 (1.57) | 0.15 (1.96) | 0.32 (2.84) | 0.33 (2.63) | 2.19 (15.33) | 2.02 (15.46) |
|  | Government and civil society | 0.56 (7.85) | 0.52 (7.73) | 0.50 (7.07) | 0.57 (7.41) | 0.99 (8.82) | 1.01 (7.96) | 0.81 (5.69) | 0.82 (6.26) |
|  | Education | 0.57 (7.97) | 0.38 (5.69) | 0.39 (5.45) | 0.50 (6.53) | 0.77 (6.83) | 0.79 (6.21) | 0.79 (5.55) | 0.81 (6.19) |
|  | Infrastructure | 0.31 (4.27) | 0.38 (5.68) | 0.38 (5.28) | 0.53 (6.95) | 0.81 (7.17) | 0.70 (5.52) | 0.69 (4.83) | 1.28 (9.76) |
|  | Agriculture | 0.34 (4.71) | 0.35 (5.31) | 0.47 (6.53) | 0.53 (6.90) | 0.83 (7.33) | 0.95 (7.47) | 0.88 (6.15) | 0.82 (6.29) |
|  | Refugees in donor country | 0.00 (0.00) | 0.00 (0.00) | 0.00 (0.00) | 0.00 (0.00) | 0.00 (0.00) | 0.00 (0.00) | 0.00 (0.00) | 0.00 (0.00) |
|  | Donor administration costs | 0.30 (4.18) | 0.27 (4.05) | 0.23 (3.29) | 0.20 (2.58) | 0.24 (2.14) | 0.25 (1.94) | 0.33 (2.34) | 0.34 (2.61) |
|  | Energy | 0.26 (3.59) | 0.40 (5.94) | 0.35 (4.88) | 0.57 (7.43) | 0.73 (6.43) | 0.58 (4.58) | 0.71 (4.94) | 0.77 (5.88) |
|  | Debt relief | 0.02 (0.31) | 0.01 (0.21) | 0.03 (0.44) | 0.01 (0.11) | 0.26 (2.31) | 0.00 (0.00) | 0.00 (0.03) | 0.00 (0.03) |
|  | Multisector | 0.72 (10.07) | 0.58 (8.68) | 0.49 (6.93) | 0.45 (5.93) | 0.54 (4.81) | 0.48 (3.76) | 0.62 (4.35) | 0.49 (3.75) |
|  | Water and sanitation | 0.22 (3.03) | 0.24 (3.65) | 0.21 (2.90) | 0.35 (4.62) | 0.51 (4.53) | 0.58 (4.53) | 0.45 (3.16) | 0.83 (6.35) |
|  | Financial services and business support | 0.13 (1.80) | 0.13 (1.92) | 0.19 (2.69) | 0.17 (2.25) | 0.49 (4.33) | 0.19 (1.50) | 0.33 (2.33) | 0.43 (3.25) |
|  | Environmental protection | 0.23 (3.15) | 0.35 (5.28) | 0.32 (4.43) | 0.22 (2.86) | 0.30 (2.66) | 0.24 (1.93) | 0.23 (1.57) | 0.10 (0.79) |
|  | Other social services | 0.28 (3.88) | 0.34 (5.08) | 0.34 (4.77) | 0.37 (4.83) | 0.65 (5.72) | 0.75 (5.90) | 0.77 (5.41) | 0.66 (5.08) |
|  | Conflict, peace and security | 0.02 (0.31) | 0.07 (1.05) | 0.07 (1.04) | 0.07 (0.87) | 0.09 (0.80) | 0.06 (0.50) | 0.09 (0.61) | 0.04 (0.27) |
|  | General budget support | 0.00 (0.02) | 0.00 (0.02) | 0.00 (0.02) | 0.01 (0.12) | 0.01 (0.04) | 0.01 (0.11) | 0.00 (0.01) | 0.00 (0.03) |
|  | Unspecified | 0.00 (0.00) | 0.10 (1.48) | 0.10 (1.34) | 0.08 (1.03) | 0.24 (2.14) | 0.30 (2.37) | 0.32 (2.24) | 0.29 (2.21) |
|  | Industry, construction and mining | 0.09 (1.26) | 0.06 (0.95) | 0.06 (0.83) | 0.07 (0.89) | 0.17 (1.55) | 0.11 (0.86) | 0.17 (1.20) | 0.15 (1.17) |
|  | Food aid and commodity assistance | 0.01 (0.11) | 0.00 (0.06) | 0.01 (0.11) | 0.01 (0.11) | 0.01 (0.07) | 0.01 (0.06) | 0.04 (0.28) | 0.04 (0.34) |
|  | Trade policy | 0.02 (0.29) | 0.03 (0.40) | 0.04 (0.52) | 0.03 (0.38) | 0.07 (0.58) | 0.18 (1.43) | 0.04 (0.25) | 0.08 (0.58) |
|  | Tourism | 0.00 (0.01) | 0.00 (0.02) | 0.00 (0.05) | 0.00 (0.05) | 0.02 (0.16) | 0.00 (0.03) | 0.01 (0.07) | 0.02 (0.19) |
